# What We Do Not Know About Stretching in Healthy Athletes: A Scoping Review with Evidence Gap Map from 300 Trials

**DOI:** 10.1007/s40279-024-02002-7

**Published:** 2024-03-08

**Authors:** José Afonso, Renato Andrade, Sílvia Rocha-Rodrigues, Fábio Yuzo Nakamura, Hugo Sarmento, Sandro R. Freitas, Ana Filipa Silva, Lorenzo Laporta, Maryam Abarghoueinejad, Zeki Akyildiz, Rongzhi Chen, Andreia Pizarro, Rodrigo Ramirez-Campillo, Filipe Manuel Clemente

**Affiliations:** 1 Faculty of Sport, Centre of Research, Education, Innovation, and Intervention in Sport (CIFI2D), University of Porto, Porto, Portugal; 2 Clínica Espregueira-FIFA Medical Centre of Excellence, Porto, Portugal; 3 Dom Henrique Research Centre, Porto, Portugal; 4 Porto Biomechanics Laboratory (LABIOMEP), University of Porto, Porto, Portugal; 5 Escola Superior de Desporto e Lazer, Instituto Politécnico de Viana do Castelo, Rua Escola Industrial e Comercial de Nun’Alvares, 4900-347 Viana do Castelo, Portugal; 6 Tumour and Microenvironment Interactions Group, INEB-Institute of Biomedical Engineering, i3S-Instituto de Investigação e Inovação em Saúde, Universidade do Porto, Rua Alfredo Allen, 4200-153 Porto, Portugal; 7 Research Center in Sports Sciences, Health Sciences and Human Development (CIDESD), University of Maia, Maia, Portugal; 8 University of Coimbra, Research Unit for Sport and Physical Activity (CIDAF), Faculty of Sport Sciences and Physical Education, Coimbra, Portugal; 9 Laboratório de Função Neuromuscular, Faculdade de Motricidade Humana, Universidade de Lisboa, Cruz Quebrada, Portugal; 10 Sport Physical Activity and Health Research & Innovation Center, 4900-347 Viana do Castelo, Portugal; 11 Núcleo de Estudos em Performance Analysis Esportiva (NEPAE/UFSM), Universidade Federal de Santa Maria, Avenida Roraima, nº 1000, Cidade Universitária, Bairro Camobi, Santa Maria, RS CEP: 97105-900 Brazil; 12 Independent Researcher, Porto, Portugal; 13 Sports Science Faculty, Department of Coaching Education, Afyon Kocatepe University, Afyonkarahisar, Turkey; 14 Faculty of Sport, Research Center in Physical Activity, Health and Leisure (CIAFEL), University of Porto, Porto, Portugal; 15 Laboratory for Integrative and Translational Research in Population Health (ITR), Rua das Taipas, 135, 4050-600 Porto, Portugal; 16 Exercise and Rehabilitation Sciences Institute, School of Physical Therapy. Faculty of Rehabilitation Sciences, Universidad Andres Bello, 7591538 Santiago, Chile; 17 Gdańsk University of Physical Education and Sport, 80-336 Gdańsk, Poland

## Abstract

**Background:**

Stretching has garnered significant attention in sports sciences, resulting in numerous studies. However, there is no comprehensive overview on investigation of stretching in healthy athletes.

**Objectives:**

To perform a systematic scoping review with an evidence gap map of stretching studies in healthy athletes, identify current gaps in the literature, and provide stakeholders with priorities for future research.

**Methods:**

Preferred Reporting Items for Systematic Reviews and Meta-Analyses (PRISMA) 2020 and PRISMA-ScR guidelines were followed. We included studies comprising healthy athletes exposed to acute and/or chronic stretching interventions. Six databases were searched (CINAHL, EMBASE, PubMed, Scopus, SPORTDiscus, and Web of Science) until 1 January 2023. The relevant data were narratively synthesized; quantitative data summaries were provided for key data items. An evidence gap map was developed to offer an overview of the existing research and relevant gaps.

**Results:**

Of ~ 220,000 screened records, we included 300 trials involving 7080 athletes [mostly males (~ 65% versus ~ 20% female, and ~ 15% unreported) under 36 years of age; tiers 2 and 3 of the Participant Classification Framework] across 43 sports. Sports requiring extreme range of motion (e.g., gymnastics) were underrepresented. Most trials assessed the acute effects of stretching, with chronic effects being scrutinized in less than 20% of trials. Chronic interventions averaged 7.4 ± 5.1 weeks and never exceeded 6 months. Most trials (~ 85%) implemented stretching within the warm-up, with other application timings (e.g., post-exercise) being under-researched. Most trials examined static active stretching (62.3%), followed by dynamic stretching (38.3%) and proprioceptive neuromuscular facilitation (PNF) stretching (12.0%), with scarce research on alternative methods (e.g., ballistic stretching). Comparators were mostly limited to passive controls, with ~ 25% of trials including active controls (e.g., strength training). The lower limbs were primarily targeted by interventions (~ 75%). Reporting of dose was heterogeneous in style (e.g., 10 repetitions versus 10 s for dynamic stretching) and completeness of information (i.e., with disparities in the comprehensiveness of the provided information). Most trials (~ 90%) reported performance-related outcomes (mainly strength/power and range of motion); sport-specific outcomes were collected in less than 15% of trials. Biomechanical, physiological, and neural/psychological outcomes were assessed sparsely and heterogeneously; only five trials investigated injury-related outcomes.

**Conclusions:**

There is room for improvement, with many areas of research on stretching being underexplored and others currently too heterogeneous for reliable comparisons between studies. There is limited representation of elite-level athletes (~ 5% tier 4 and no tier 5) and underpowered sample sizes (≤ 20 participants). Research was biased toward adult male athletes of sports not requiring extreme ranges of motion, and mostly assessed the acute effects of static active stretching and dynamic stretching during the warm-up. Dose–response relationships remain largely underexplored. Outcomes were mostly limited to general performance testing. Injury prevention and other effects of stretching remain poorly investigated. These relevant research gaps should be prioritized by funding policies.

**Registration:**

OSF project (https://osf.io/6auyj/) and registration (https://osf.io/gu8ya).

**Supplementary Information:**

The online version contains supplementary material available at 10.1007/s40279-024-02002-7.

## Key Points

**Table:** 

Research investigating stretching in healthy athletes is mostly limited to small-scale trials of adult, nonelite male athletes, assessing acute effects of static active stretching or dynamic stretching applied to the lower limbs during the warm-up, commonly compared with passive controls.
Outcomes have mostly been limited to general performance tests, with scarce information on the underlying mechanisms and on sport-specific performance data. Dose–response relationships were seldom explored.
Surprisingly, only five trials assessed injury data. Their results do not support a role for stretching in injury prevention, but further research is required on the topic.
Future research and funding policies should devote more effort toward investigating the gaps identified in this scoping review.

## Introduction

In the context of sports and physical exercise, stretching refers to a set of interventions focused primarily on improving joint flexibility or range of motion (ROM) [1–3]. The benefits of stretching on flexibility and ROM seem consensual in the scientific milieu [4–10] (i.e., large and mostly homogeneous body of research supporting this effect) and are integrated into internationally recognized guidelines for exercise prescription [1, 2]. The mechanisms mediating stretching effects on flexibility include structural (e.g., increased fascicle length), mechanical (e.g., decreased muscle stiffness), and sensorial/neural changes (e.g., improved stretch tolerance) [8, 10–14]. The most commonly used stretching methods are static (passive or active), dynamic, ballistic (a form of dynamic stretching where the velocity of limb motion is very high), and passive stretching coupled with isometric muscle actions, commonly termed proprioceptive neuromuscular facilitation (PNF) [1, 3]. These stretching modalities may operate through partially overlapping mechanisms and produce differentiated effects [3, 4, 8, 9, 15]. Stretching volume, intensity, and weekly frequency may have far-reaching impacts on the dose–response relationships [15].

Although stretching interventions conducted in athletes are commonly focused on improving ROM, arguments in favor of stretching also revolve around its value for injury prevention [4, 16–18], warm-up [4, 19, 20], and cool-down/recovery [21–23]. Nonetheless, the evidence for the overall effectivity of stretching in the aforementioned contexts is unclear and heterogeneous at best [5]. Furthermore, answering the question “Can I stretch?” does not answer the question “Must I stretch?” [5]: for the purposes of ROM gains, injury risk, warm-up, and cool-down, stretching can be performed but possibly does not need to be mandatory. Conceivable exceptions are a few selected sports with very specific demands (i.e., gymnastics), although targeted research on this topic is required. Aside from the lack of robust evidence favoring stretching, recent evidence suggests that alternative interventions, such as strength training or foam rolling, may offer similar ROM gains [5, 24–29]. However, such findings should not be used as an argument against stretching, as its applications are not limited to improving ROM (e.g., it may improve strength and muscle hypertrophy [30–32]), and other effects of stretching warrant greater research efforts.

Focusing overly on the acute effects of stretching when applied during the warm-up and/or the cool-down, as well as on the acute and/or chronic effects on ROM and injury risk, may systematically allocate more and more resources (human, financial, and time based) to the same areas of research, while risking neglecting or overlooking other opportunities for implementing stretching interventions focused on alternative outcome measures. For example, the acute nonlocal effects of stretching on ROM and strength [13, 14] are based on generalized mechanisms that may be harnessed when considering injury rehabilitation, as stretching the noninjured areas may generate effects on the injured areas. Stretching has also been shown to generate acute changes in the autonomic nervous system [33–35] and in the cardiovascular system [34, 36, 37]. Moreover, despite the existing research on the chronic effects of stretching focusing on muscle architecture, scarce research is available assessing the effects on nerves and other structures [38]. Athletic preparation may potentially benefit from extending the scope of research on stretching.

Within the traditionally analyzed topics, much research is focused on static stretching (active and passive), PNF (albeit only in a few select types of PNF), and dynamic stretching [4]. How, when, and why athletes could benefit from lesser-known stretching modalities such as global active stretching (SGA, from the original French expression) [39] and Gyrotonic stretching [40], among others, is largely unknown and warrants further exploration. How these interventions may be substantially changed by manipulating the set of provided instructions constitutes another emerging field of research [41]. It is also troublesome that a few purported applications of stretching have remained for decades despite the absence of research to sustain them, as is the case with stretching for the recovery from groin pain or injury in athletes [18]. Overall, valuable research opportunities and potentially relevant applications of stretching in sporting and athletic environments are possibly being wasted due to overemphasis on specific domains (e.g., ROM) and poor investment in relevant others (e.g., nonlocal neural effects).

Scoping reviews perform a systematic mapping of existing evidence and identify relevant gaps in the literature [42, 43]; their aim is not to provide pooled results or analytical comparisons, but to map the existing evidence [43]. Future research would benefit from clear guidance based on an evidence gap map (EGM) [44, 45], and scoping reviews provide a suitable and systematic approach to building such maps [43]. Fitting into the broad approach of most scoping reviews, EGMs graphically represent the body of evidence, conveying an intuitive visual interpretation of research efforts allocation (i.e., where the evidence is rich versus where it is scarce) [44–46]. Such data assist in developing policies and guidelines and exposes areas requiring further research [44–46]. Sports medicine-related reviews with EGMs have been published in recent years [47–49]. Therefore, a scoping review with EGM will provide a clearer picture of what are the research trends, as well as what is known and unknown (i.e., gaps in research) about stretching in athletes, which can inform future policies and funding.

A quick search in PubMed (using “stretching [Ti/Ab] AND sport* [Ti/Ab] OR exercise* [Ti/Ab]”) yielded 1611 records from inception to 2012 and 2177 records from 2013 to 2022, showing that more than half of all studies on the subject of stretching in healthy athletes have been published in the last ~ 10 years and highlighting the fast-growing pace of research on stretching. Therefore, our goal was to perform a systematic scoping review with EGM of stretching-related studies in healthy athletes to identify trends and gaps in the literature and inform stakeholders in priorities for future research.

## Methods

This systematic scoping review with EGM followed the Preferred Reporting Items for Systematic Reviews and Meta-Analyses (PRISMA) 2020 [50], the PRISMA extension for Scoping Reviews (PRISMA-ScR) [42], and the Cochrane guidelines [51] (e.g., search for errata before closing the final list of included studies).

### Eligibility Criteria

Research articles published in peer-reviewed journals were considered, with no limitations imposed on publication date or language. Eligibility criteria were set based on the Participants, Intervention, Comparators, Outcomes and Study Design (PICOS) framework:

(P) Healthy athletes of any age, sex, or sport, with a competitive level corresponding to tier 2 (trained/developmental) or higher of the Participant Classification Framework (PCF; tiers 0 and 1 are not athletes, falling outside the scope of this review) [52], regardless of how the original studies have classified them. Studies with injured (e.g., studies on rehabilitation or return to sports) or disabled athletes (e.g., cerebral palsy) were excluded. Since the goal was to provide an overview of the research field and not provide meta-analytical summaries of data, no minimum number of participants per study was stipulated.

(I) Acute (single session or multiple sessions but with assessments of acute responses up to 72 h postintervention) or chronic (multiple sessions with assessment of pre- to post- differences) interventions exclusively using any form of stretching (e.g., static active or passive stretching, dynamic stretching, PNF, other), either single mode (e.g., static passive stretching only) or combined mode (e.g., static passive stretching combined with static active stretching). Multimodal interventions (e.g., stretching + foam rolling or stretching + strength training) were not considered. We chose not to predefine a minimum length for an intervention to be considered chronic (e.g., 4 weeks or 8 weeks), since these thresholds may vary depending on the specific outcome (i.e., some outcomes may experience faster adaptations than others) and on the characteristics and doses of the interventions. Moreover, these thresholds are largely arbitrary.

(C) Comparators were not compulsory (because we were not directly comparing the effectiveness or efficacy of stretching interventions). However, if available, these were considered and could include stretching interventions with different modalities, intensities and/or durations, nonstretching-based interventions, multimodal interventions (e.g., stretching + strength training), or passive controls.

(O) At least one of the following: acute or chronic physiological, biomechanical, psychological, performance-related outcomes/adaptations, and/or data on injury risk (from prevention-focused studies).

(S) All types of experimental and observational studies [single- or multi-arm, randomized (parallel, crossover, cluster, other) or nonrandomized], including case series and case studies.

### Information Sources and Search Strategy

CINAHL, EMBASE, PubMed, Scopus, SPORTDiscus (via EBSCO), and Web of Science were searched on 15 July 2022, and again on 1 January 2023. As per the preregistered protocol, additional procedures (e.g., snowballing citation tracking, expert consultation) were not performed, due to the large number of included studies (> 300). A comprehensive reporting of information sources and of the search strategy is provided in the Electronic Supplementary Material (ESM Sects. 1.1 and 1.2.).

### Selection Process

Three authors (JA, SRR, and AP) independently screened all retrieved records. A third author (RA) arbitrated in case of disagreements. Automated removal of duplicates was performed using EndNote 20.3 for Mac (Clarivate), but further manual removal of duplicates was required.

### Data Collection Process

Eight authors (JA, SRR, FYN, AFS, LL, ZA, RC, AP) independently extracted data from the included studies. The coordinator author (JA) double-checked all assessments. After completion of data collection, four authors (RA, HS, RRC, FMC) reanalyzed 40 randomly selected studies (~ 13%) to further ensure proper data quality and completeness of data extraction. Data on competitive level were recoded by three authors (JA, SSR, and AP) using the PCF [52], but excluding tiers 0 and 1 participants: (i) tier 2: Trained/developmental; (ii) tier 3: Highly trained/national level; (iii) tier 4: Elite/international level; (iv) tier 5: World class. A fourth author (RA) arbitrated in case of disagreements.

### Data Items and Management

Data were extracted within six domains: (i) participant-related information, (ii) intervention-related information, (iii) comparator-related information, (iv) outcome-related information, (v) study design, and (vi) context of interventions. Full details and explanations can be found in the ESM (Sect. 1.3). Given that stretching interventions were at the core of this work, we followed a mainstream, reader-friendly approach to the classification of stretching modalities [3]: (i) static stretching involving the lengthening of a muscle until a feeling of stretch or point of discomfort are reached, and keeping that position, with (passive) or without (active) assistance from an external force (e.g., a person or a machine); (ii) dynamic stretching involving controlled movements through the joint ROM; (iii) ballistic stretching as an extreme form of dynamic stretching performed at high speeds and with bouncing actions near or at the end-ROM; (iv) PNF stretching combining static stretching and isometric contractions in a cyclical pattern; and (v) other forms of stretching (e.g., SGA). A complete description of data management procedures, including further details regarding how stretching interventions were classified, is provided in the ESM (Sect. 1.4).

### Data Synthesis Methods

A narrative synthesis was performed, accompanied by data summaries (number, percentage) for the previously defined data items. To provide an overview of the existing body and the corresponding gaps in research, an EGM was constructed to graphically represent the body of evidence and intuitively convey an overview of the existing evidence and the current research gaps [44–46]. In the EGM, the different circles have proportional sizes, reflecting the number of trials; however, this proportionality is only applied within each cell, and not between cells.

## Results

### Study Selection

**Fig. 1:**
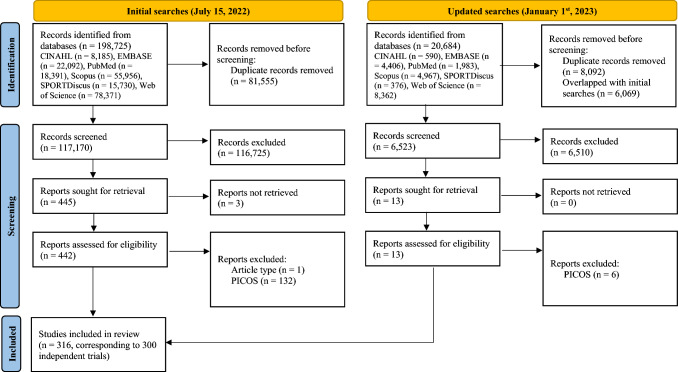
PRISMA 2020 flow diagram

The initial and updated database searches resulted in ~ 220,000 records, of which 316 studies (corresponding to 300 independent trials) [39, 53–367] complied with eligibility criteria and were included in this scoping review (Fig. 1). This means that 300 independent trials gave origin to 316 publications, as some trial authors chose to report different outcomes in different publications (suggesting a high risk of bias for selective reporting, especially in the absence of a preregistered protocol). More detailed information on study selection is provided in the ESM (Sect. 2.1).

### Publication-Level Information

#### Publication Date and Study Design

**Fig. 2:**
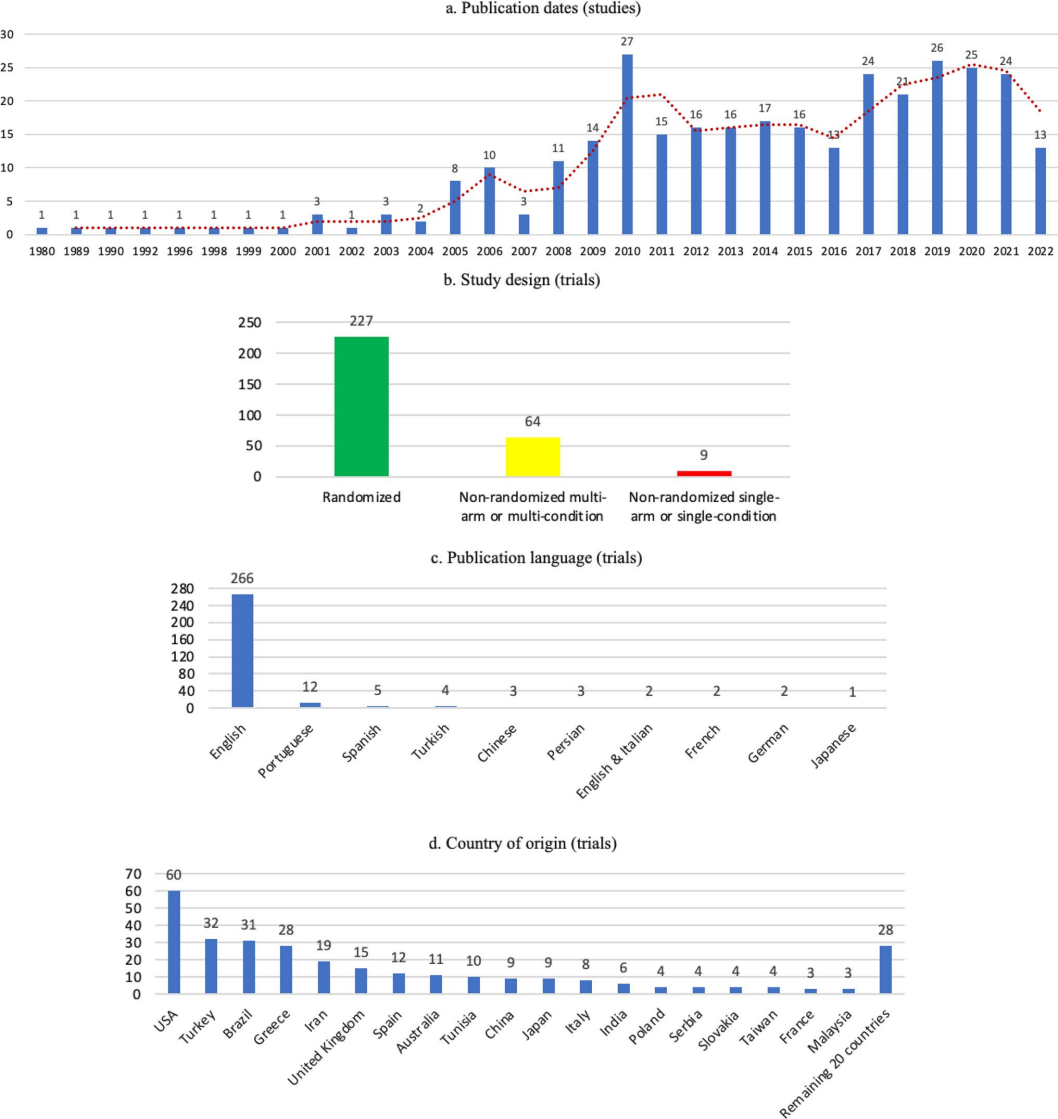

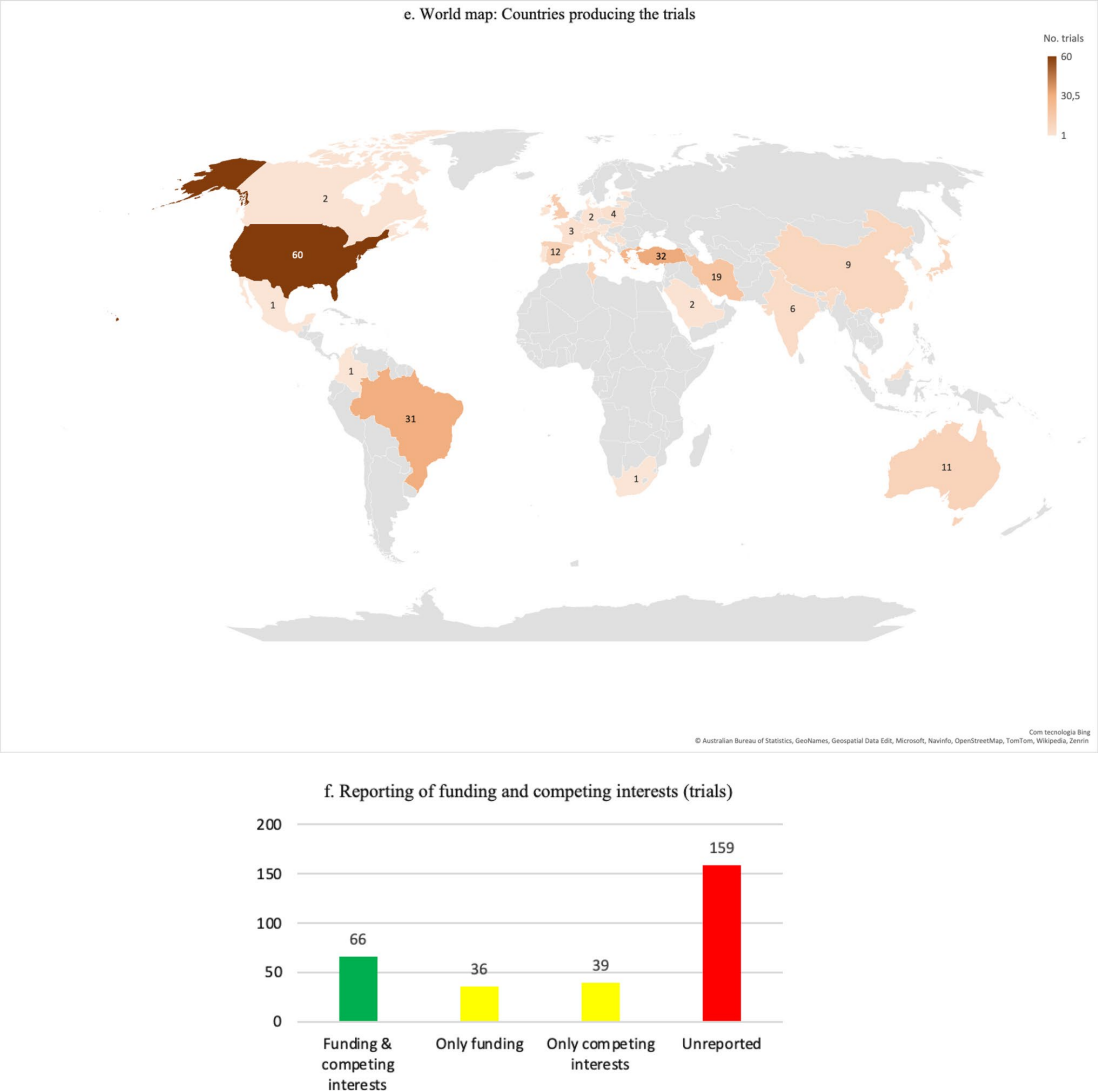
Publication-level distributions

The 300 trials were published between 1980 and 2022 (ESM Sect. 2.2), resulting in 316 publications (i.e., some trial authors chose to report different outcomes from a single experiment in different publications). Date of publication was evenly distributed before or after 2015 (48.7% before 2015 and 51.3% from 2015 onwards; Fig. 2a), meaning that more than half of research was performed in the last 7 years (last search on 1 January 2023). Since 2008, research on the topic has steadily generated over ten publications each year. Most trials (*n* = 227, 75.7%) were randomized (Fig. 2b), followed by nonrandomized multi-arm/condition trials (*k* = 64, 21.3%), and a minority of nonrandomized single-arm/condition trials (*k* = 9, 3.0%).

#### Publication Language and Geographical Location

English was the predominant language of publication (266 trials, 88.7%). Other languages each represented less than 5% of trials, and when combined represented 11.3% (Fig. 2c). Most trials were performed in Europe (*k* = 94, 31.3%), followed by North America (*k* = 63, 21.0%), Asia (*k* = 55, 18.3%), South America (*k* = 33, 11.0%), and Turkey (technically, belonging to both Europe and Asia; *k* = 32, 10.7%). Africa contributed with 11 trials (3.7%, of which 10 were from Tunisia), Oceania also delivered 11 trials (3.7%, all from Australia), while one research had unclear origin (either Europe or South America). Out of nearly 40 countries contributing with research, the USA produced the largest number of trials (*k* = 60, 20.0%), followed by Turkey (*k* = 32, 10.7%), Brazil (*k* = 31, 10.3%), Greece (*k* = 28, 9.3%), Iran (*k* = 19, 6.3%), and UK (*k* = 15, 5.0%) (Fig. 2d and e). More detailed information is available in the ESM (Sect. 2.2).

#### Funding and Competing Interests

A total of 102 studies included a funding statement, with 48 trials (16.0%) reporting not having funding and 54 trials (18.0%) reporting their funding sources; however, the majority of trials (*k* = 198, 66.0%) did not provide any (published) funding statement. A single trial (0.3%) reported having a conflict of interest, while 102 trials (34.0%) declared having no competing interests; again, the majority of trials (*k* = 197, 65.6%) failed to provide a published competing interests statement. Overall, 159 trials (53.0%) failed to report both funding and competing interests (Fig. 2f). Considering the 153 trials published from 2015 onward, the percentage of nonreported information on funding (51.6%) and competing interests (41.8%) is lower in comparison with the 147 trials published before 2015 (81.0% and 89.1%, respectively).

### Participant-Related Characteristics

#### Sample Size and Sex

Across the 300 trials, a combined aggregate of 7080 athletes were involved, of which 6005 performed stretching and 1075 athletes only participated in nonstretching-related control groups. Trials averaged 23.3 ± 20.8 athletes per trial (median: 18), ranging from 5 [173] to 220 athletes [177]. A total of 282 trials (94.0%) had fewer than 51 athletes (more detailed information in ESM Sect. 2.2).

A total of 168 trials (56.0%) only included male athletes (*n* = 4035), 54 trials (18.0%) only included female athletes (*n* = 1079), and 36 trials (12.0%) included both male (*n* = 589) and female athletes (*n* = 378). In one study, there was a nonanalyzed participant, but it was unclear whether this individual was male or female, and this participant was therefore not considered here. In total, there were 4624 male (65.3%) and 1457 female athletes (20.6%). Two trials [124, 135] included male and female athletes (*n* = 48) but failed to report the number or percentage of each. Forty trials (13.3%) including 950 athletes did not report on sex. A summary of sample size and sex can be found in Sect. 3.7 (EGM).

#### Age

Most trials (*k* = 290, 96.7%) reported age, but not in an easily comparable manner, which precluded a simplified cross-study synthesis. The reasons for that and a more complete reporting can be found in the ESM (Sect. 2.2). Considering the 25 trials (8.3%) that provided ranges, age varied from to 8 [149, 338] to 36 [177] years. The narrowest range was 14–15 years of age [265] and the broadest range was 17–36 years [177]. Only four trials included athletes ≤ 12 years of age [86, 141, 149, 338], seven trials included athletes ≥ 30 years of age [105, 177, 218, 232, 311, 329, 358], and a single trial included athletes up to 36 years [177]. Age was reported in the form of mean ± SD in 264 trials (88.0%): at the lower end, a mean age of 9.6 ± 1.5 years was reported [55], while at the upper end the mean age was 35.7 ± 6.1 years [137].

#### Sports and Competitive Level

Most trials (*k* = 260 trials, 86.7%) were conducted within a single sport. Thirty-six trials (12.0%) included athletes from multiple sports, and four trials (1.3%) [81, 82, 108, 261] provided insufficient information to assess this item. The available information showed that at least 43 sports were represented (possibly more). Soccer was represented in 98 trials (26.2%), track and field in 41 (11.0%), volleyball in 32 (8.6%), basketball in 29 (7.8%), and artistic gymnastics in 19 (5.1%). All other sports were represented in less than 4% of trials each. More details are provided in the ESM (Sect. 2.2).

Regarding the competitive level (PCF), no trial was found including tier 5 athletes. Most trials (*k* = 175, 46.8%) included tier 2 athletes, followed by tier 3 (*k* = 95, 25.4%). Tier 4 (*k* = 18, 4.8%), mixed tiers 2 and 3 (*k* = 8, 2.1%), and mixed tiers 3 and 4 (*k* = 4, 1.1%) were less common. The few tier 4 trials were spread across several different sports (artistic gymnastics, Brazilian jiu-jitsu, fencing, handball, judo, kickboxing, rhythmic gymnastics, rowing, soccer, synchronized swimming, taekwondo, tennis, track and field, volleyball, wrestling), while the mixed tiers 3 and 4 trials included field hockey, futsal, and rhythmic gymnastics (one study reported multiple individual and team sports, but it was unclear which [117]). A visual summary of sport and competitive level can be found in Sect. 3.7 (EGM).

### Context of Intervention

Fully detailed, context-level reporting of stretching interventions is provided in the ESM (Sect. 2.2), while summary data for key features (e.g., length of interventions) are provided in Sect. 3.7 (EGM).

#### Length of Interventions

Most trials focused on acute stretching effects (*k* = 244, 81.3%), while 51 trials (17.0%) assessed chronic effects (see ESM Sect. 2.2 for the remaining cases). Trials assessing chronic effects lasted between 1 and 21 weeks [90, 285, 312, 351], with an average of 7.4 ± 5.1 and a median of 6.0 [interquartile range (IQR) 4.0–10.5] weeks. Eleven trials, from 12 studies, (3.7%) failed to reach a minimum length of 4 weeks [78, 90, 154, 161, 211, 237, 285, 299, 300, 304, 329, 351] that would suffice to generate adaptations to stretching interventions in humans [368]. Trials lasting up to 8 weeks represented 13.0% of the publications (*k* = 39), and only six trials, from 10 studies, (2.0%) lasted ≥ 16 weeks [99, 188, 259, 265, 305–309, 312]. Additional information is available in the ESM (Sect. 2.2).

#### Number of Weekly Sessions and Total Number of Sessions in Chronic Trials

The number of weekly stretching sessions in chronic interventions varied from < 1 per week [188] to 14 weekly sessions [329], with an average of 3.4 ± 2.0 and a median of 3 (IQR 3.0–4.0). Thirty-three trials (67.3%) had less than 4 weekly sessions, 15 trials (27.2%) had between 4 and 7 sessions, and a single trial exceeded 7 weekly sessions [329]. Six of the 55 relevant trials (10.9%) did not report the number of weekly sessions [78, 105, 177, 211, 258, 304].

The total number of stretching sessions in trials assessing chronic effects ranged from 3 to 150 [90, 154, 237, 312, 351], with an average of 24.6 ± 24.1, a median of 19 (IQR 10.3–31.5), and was unreported and impossible to assess in three trials [105, 211, 304].

#### Within-Season Timing

Most trials (*k* = 180, 60.0%) failed to report the within-season timing and provided insufficient information for the reviewers to infer this (e.g., by providing specific date ranges for data collection). Of the trials that reported this information, 70 (23.3%) were performed during the competitive season, 24 (8.0%) in the off-season and 22 (7.3%) in the pre-season. The remaining trials (*k* = 4, 1.2%) were either mixed (e.g., pre-season and competitive season) [125, 177, 365] or the authors reported the specific weeks of the season, but it was unclear whether that still represented the pre-season or was already in the competitive season (i.e., coded as unclear) [182].

#### Within-Session Timing

**Fig. 3:**
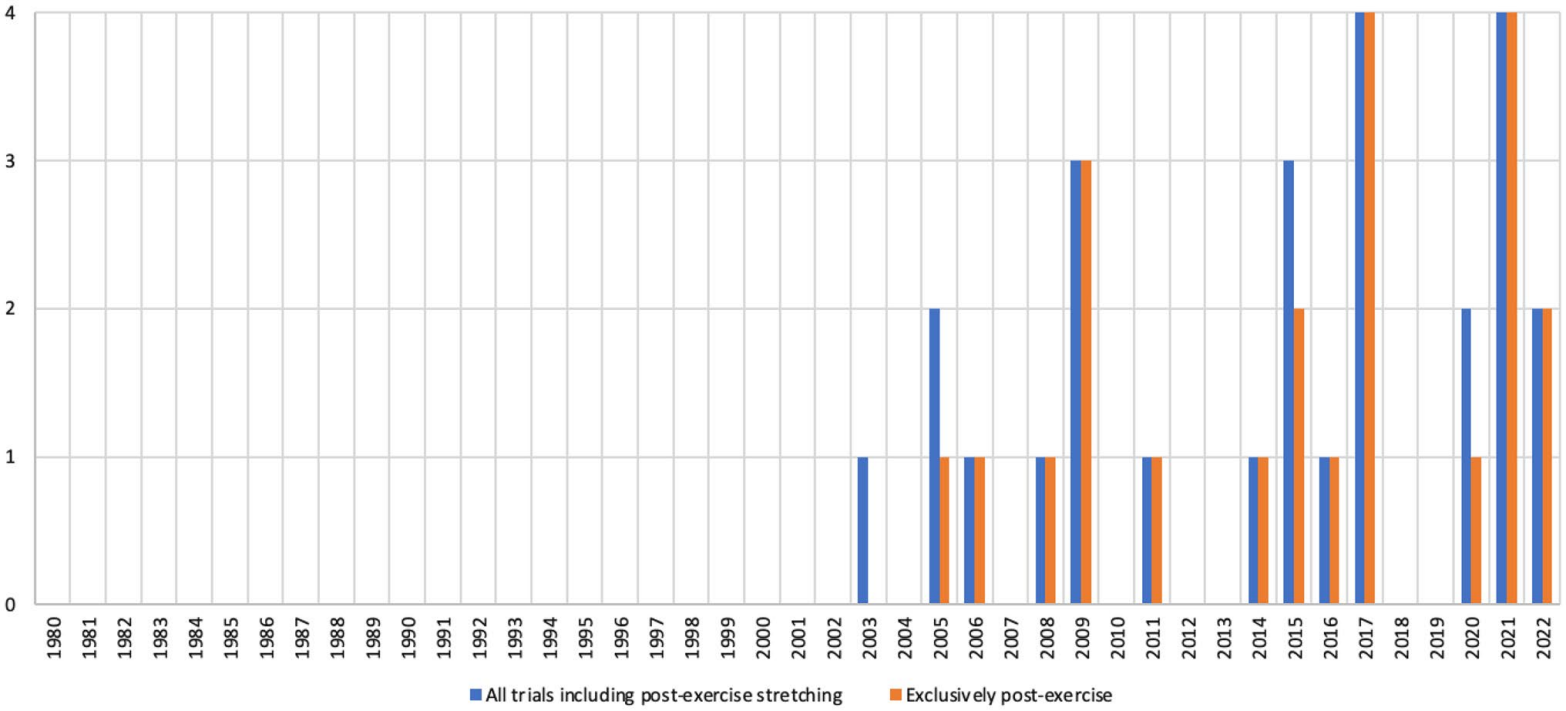
Time map of research on postexercise stretching

Most trials (*k* = 252, 84.0%) implemented stretching as warm-up or within the context of a warm-up. Postexercise stretching was analyzed in isolation in 22 trials published in 26 studies (7.3%) [57, 99, 105, 111, 113, 115, 123, 139, 160, 177, 245, 259, 277, 278, 285–287, 294, 305–309, 336, 347, 357], in conjunction with warm-up (i.e*.*, stretching in the warm-up and also postexercise) in two trials [124, 175], optionally in the warm-up or postexercise in one trial [150], and combined with independent sessions in another trial [87]. Trials studying the effects of postexercise stretching in athletes began to be published in 2003 and seem to be growing, albeit there is fluctuating interest in the topic (Fig. 3).

The remaining categories (e.g., inter-set, independent sessions, at night) combined were represented in only 13 trials (4.2%) (see ESM Sect. 2.2 for full details) [87, 95, 124, 150, 154, 175, 191, 247, 262, 268, 273, 312, 352]. Of note, all inter-set research with athletes was performed between 2009 and 2015 and limited to males [95, 247, 262, 352]. Additionally, the within-session timing of the stretching intervention was unclear or unreported in 13 trials (4.3%) [85, 90, 92, 106, 131, 141, 171, 188, 208, 235, 265, 274, 311].

### Intervention-Level Information

The full details of intervention-level related features can be found in the ESM (Sect. 2.3) and a summary of key features is presented in Sect. 3.7 (EGM).

#### Stretching Interventions

Static active stretching was the most common modality (*k* = 187, 62.3%), followed by dynamic stretching (*k* = 115, 38.3%), and static passive stretching (*k* = 77, 25.7%). PNF was implemented in 36 trials [12.0%, mostly contract–relax (*k* = 22), with the remaining methods being represented in less than 4 trials each], ballistic stretching in 13 trials (4.3%), and static stretching (unclear if active or passive) in 6 trials (2.0%). The remaining stretching modalities (e.g., SGA) were applied in two or less studies each (see ESM Sect. 2.3 for further details).

Overall, 154 trials (51.3%) applied a single stretching modality (e.g., ballistic stretching [205], PNF [142]), 133 trials (44.3%) compared two or more stretching modalities (e.g., dynamic stretching versus static active stretching [61]), and 11 trials (3.7%) implemented a single combination of stretching modalities (e.g., dynamic stretching + static active stretching within the same intervention group [314]). Twelve trials (4.0%) had at least one group performing some stretching modality with superimposed vibration (e.g., static active stretching + vibration [235]) (*k* = 10, 3.3%), heat (*k* = 2, 0.6% [106, 176]), or ice (*k* = 1, 0.3% [106]); considering the eligibility criteria, these were classified as being comparators.

Twenty-two trials (7.3%) compared different doses within a given stretching modality (e.g., 1 versus 2 versus 3 sets of ballistic stretching [205]; 6 versus 12 versus 18 repetitions of dynamic stretching [121]; and 35 s repetitions versus 65 s repetitions of PNF contract–relax [142]). There were specific comparisons within dynamic stretching: three trials (1.0%) compared stationary dynamic stretching versus dynamic stretching while moving [97, 167, 168], and one trial (0.3%) compared dynamic stretching performed at self-paced versus self-paced with additional forces versus maximal speed [343]. Five trials (1.7%) compared continuous with intermittent static active or passive stretching [82, 103, 114, 147, 149]. A single trial (0.3%) included a comparison of static active stretching to differing intensity thresholds: less than to the point of discomfort versus to the point of discomfort [117]. Occasionally (*k* = 7, 2.3%), the order of the interventions (e.g., dynamic stretching + static active stretching versus static active stretching + dynamic stretching [322]) was compared [70, 77, 91, 117, 216, 315, 322]. Additional information is available in the ESM (Sect. 2.3).

#### Nonstretching Comparators

A total of 169 trials (56.3%) included a no-stretching control group (i.e., passive controls or no-stretching on contralateral limb), while 76 trials (25.3%) included ≥ 1 comparator groups involving nonstretching interventions (e.g., parallel squat [202], FIFA 11 + [79]), or stretching combined with additional interventions (e.g., dynamic stretching followed by vibration foam rolling [229]). Nonstretching-related comparators were highly heterogeneous, and most were underrepresented. Strength-based training (e.g., resistance training [255], plyometrics [283]; 25 trials, 8.3%) and multimodal exercise and/or warm-up programs (e.g., FIFA 11 + [79]; 20 trials, 6.7%) were the most common comparisons, followed by aerobic-based activities (e.g., cycling [247]; 13 trials, 4.3%). A more detailed explanation is available in the ESM (Sect. 2.3).

#### Anatomical Regions Stretched

**Fig. 4:**
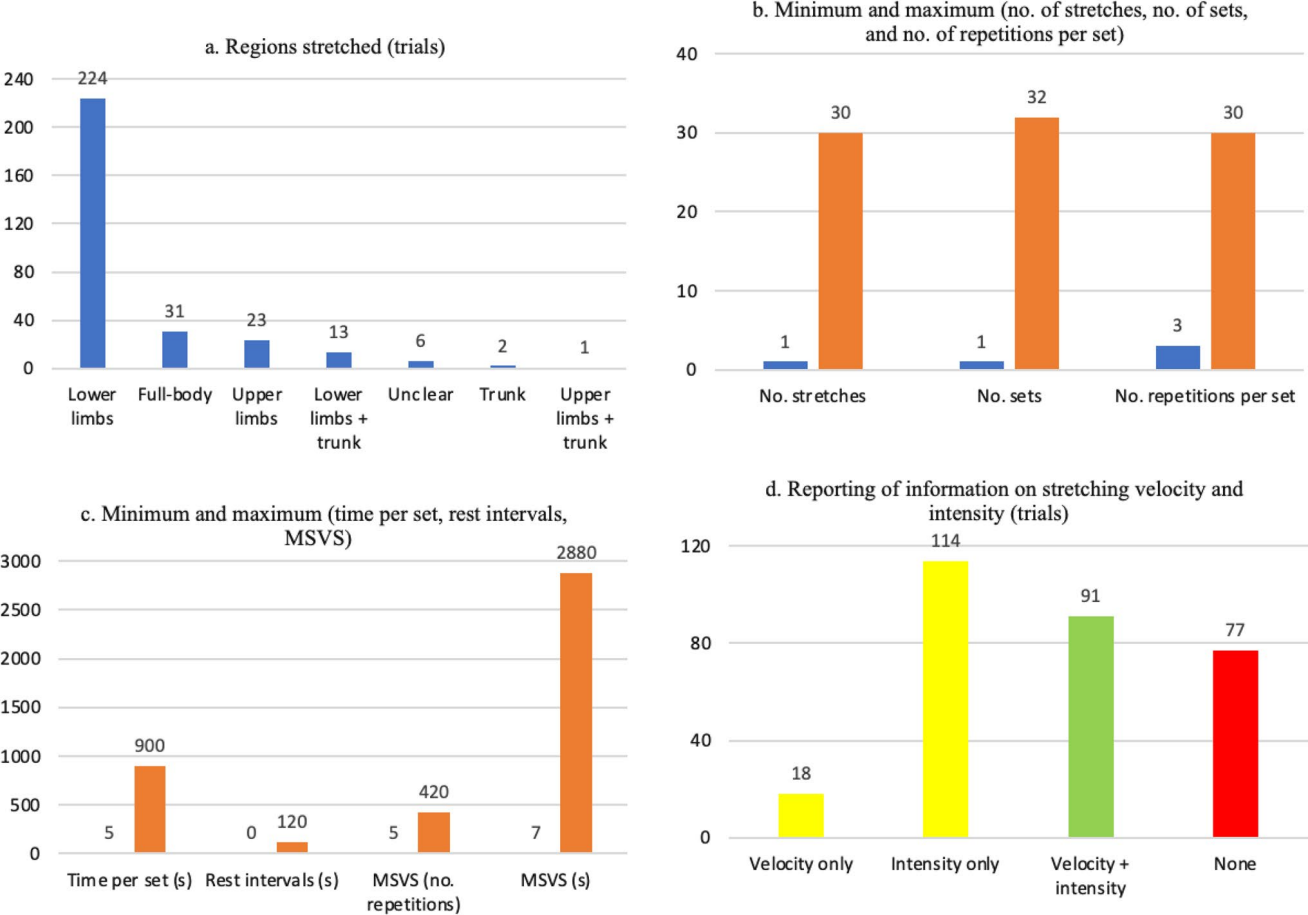
Summary of prescription of stretching interventions. For the stretching modalities, refer to Sect. 3.7 (EGM). *Only considering the trials that reported the relevant values. MSVS, minimum stretching volume per session

Most trials (*k* = 224, 74.7%) focused on stretching the lower limbs (Fig. 4a). An additional 13 trials (4.3%) stretched the lower limbs and the trunk. The upper limbs were stretched in 23 trials (7.7%), while 31 trials (10.3%) stretched the full body. Full details are provided in the ESM (Sect. 2.3).

#### Number of Stretches Per Intervention

When reported, the number of stretching exercises per intervention (Fig. 4b) ranged from one in 54 trials (18.0%; e.g., [60, 89]) to 22 (single stretching modality [172]) or 30 (combined stretching modalities, e.g., dynamic stretching + static active stretching group [250]). We refrained from calculating pooled means and standard deviations, as there was considerable complexity that could result in miscalculations (a detailed explanation is provided in ESM Sect. 2.3).

#### Number of Sets

The number of sets was not always explicitly stated but, in most cases, it could be inferred from the description that a single set was performed (for exceptions, see ESM Sect. 2.3). When reported, the minimum number of sets was one (83 trials, 27.7%), while the maximum was 32 (high-volume group in [363]) (Fig. 4b). For purposes of making terminology more uniform across different stretching modalities, in cases such as static stretching, each repetition was deemed a set and we then reported time per repetition.

Four chronic trials [55, 287, 327, 336] (1.3%) had a progressive number of sets, i.e., the number of sets increased throughout the weeks (e.g., 2 sets in week 1 versus 5 sets in week 4 [336]). Fifteen trials from 16 studies (5.0%) compared different numbers of sets [75, 82, 98, 110, 178, 203–205, 249, 332, 340, 359–363] (e.g., 4 versus 8 versus 12 sets [98]), with difference between the minimum and the maximum number of sets ranging from twofold (e.g., 2 versus 4 sets [75]) to 16-fold (e.g., 1 versus 16 sets [362]). Trials comparing continuous versus intermittent static active or passive stretching [103, 114, 147, 149] also had groups performing a different number of sets (e.g., single, longer set for continuous stretching versus six smaller sets for intermittent stretching [147]). For other less common cases, see the ESM (Sect. 2.3).

Trials of multiple stretching interventions also presented relevant specificities regarding the number of sets. For example, in 30 trials (10.0%) the number of sets was not equated between the interventions (e.g., one set for static active stretching versus three sets for dynamic stretching [66]). The difference in number of sets ranged from a 133.3% increase (three sets for Mulligan stretching versus four sets for static passive stretching [90]) to a 500% increase (two sets for static passive stretching versus ten sets for dynamic stretching [146]). In a subset of trials, the difference in the number of sets was mostly between combined versus noncombined interventions, but the implementation could be the reverse. For example, one trial applied two sets for the static passive stretching and dynamic stretching interventions, but only one set for the combined dynamic + static passive stretching groups [77], possibly in an attempt to match training volume. Conversely, another trial implemented two sets of dynamic stretching, but a combined group performed three sets of static passive stretching in addition to the two sets of dynamic stretching [167], effectively increasing training volume.

#### Number of Repetitions or Time Per Set

For interventions reporting time, values ranged from 5 s (e.g., [178, 343]) to 900 s [39, 60] (Fig. 4c). In nine trials (3.0%) [81, 82, 142, 151, 180, 224, 340, 359, 360], different durations were compared (e.g., 5 s for the five-set group versus 30 s for the single-set group [359, 360] or 35 s for the low-volume group versus 65 s for the high-volume group [142]). This was obviously extended to the trials comparing continuous versus intermittent stretching [103, 114, 147, 149]. In some experiments, the duration could vary [237, 252, 259, 279, 280, 336, 367] (e.g., 19–30 s [336]), but without a rationale being provided and without an analysis of whether that varying duration had an impact on the results. Two trials [270, 327] implemented a progressively increasing duration (e.g., 45 s in the first 3 weeks versus 75 s in the last 6 weeks [270]).

For interventions reporting repetitions per set, values ranged from 3 (for the ballistic stretching group in [331]) to 30 (for the dynamic stretching groups in [330]) (Fig. 4b). One trial compared different volumes (6 versus 12 versus 18 repetitions) [121]. Five trials from seven studies (1.7%) presented a variable number of repetitions [237, 243, 279–281, 321, 337] (e.g., 3–10 repetitions for the dynamic stretching group [243]), but without a rationale behind it or any monitoring being implemented to assess the impact of such variation on the results. One trial implemented a progressive increase in the number of repetitions, starting with 15 and adding 5 repetitions every three sessions [258].

Trials of multiple stretching modalities could either report all modalities in time (e.g., 30 s for both dynamic and static active stretching [220, 221]) or report some modalities in time and others in repetitions (e.g., 10 repetitions for dynamic stretching, 90 s for static active stretching [314]). Accordingly, combined interventions could be reported in uniform units (e.g., 120 s of dynamic stretching + 300 s of static passive stretching [244]) or in nonuniform units (e.g., 30 s of ballistic stretching + 15 repetitions of dynamic stretching [219]). However, multiple reporting problems were identified and are detailed in the ESM (Sect. 2.3).

#### Rest Intervals

When reported (*k* = 187, 62.3%), it was not always clear whether the rest intervals were between exercises or between sets. Regardless, rest intervals ranged from 0 s (e.g., [105, 276]) to 120 s [57] (Fig. 4c). While most trials reporting rest intervals provided fixed values, some provided a narrow (e.g., 5–8 s [281]) or large (e.g., 45–60 s [93]) range of possible values. Some dynamic stretching trials reported recovery in terms of a walked distance (e.g., 20 m walking recovery [167]). It should be highlighted that some trials provided rest intervals that were longer than the duration of each set [56, 57, 184, 242, 249, 335, 342, 343, 351]. For example, one trial requested the athletes to perform 3 sets of 10 s repetitions of combined static active and passive stretching, but the rest between exercises lasted 30 s [184], i.e., the work to rest ratio was 1:3. Additional information is available in the ESM (Sect. 2.3).

#### Minimum Stretching Volume Per Session

There was insufficient information to assess minimum stretching volume per session in 32 trials (10.7%). For interventions reported in seconds, the minimum stretching volume per session ranged from 7 s of static passive stretching [218] to 2880 s of PNF (contract–relax) in the last 2 weeks of the intervention [327] (Fig. 4c). For those reported in number of repetitions, the minimum stretching volume per session was 5 repetitions [267] and the maximum was 420 [330] (Fig. 4c). As for other variables (e.g., age, number of stretches per intervention), we refrained from providing pooled means and standard deviations due to the mixed character of several trials, such as: (i) having different volume groups (e.g., low versus intermediate versus high volume groups, ranging from 100 to 300 repetitions [205], or 200 s in the smaller duration group versus 1200 s in the larger duration group [82]); (ii) presenting a dynamic, evolving volume across the trial (e.g., 60 repetitions during the first week sessions versus 160 repetitions in the last week sessions [258]); (iii) having mixed reporting (e.g., a combined group performing 70 repetitions of dynamic stretching + 810 s of static active stretching [314]); or (iv) having groups with sufficient versus insufficient information to assess this variable (e.g., 360 s for static active stretching but insufficient information regarding ballistic and dynamic stretching [174]).

#### Stretching Velocity and Intensity

Stretching velocity (e.g., 1 repetition every 2 s for dynamic stretching [79], slowly for static active stretching [114]) and intensity (e.g., to point of discomfort [81], maximum ROM while avoiding pain [141]) were unreported in 77 trials (23.3%), 114 trials (48.0%) reported stretching intensity but not stretching velocity, and 18 trials (6.0%) reported stretching velocity but not intensity. Overall, 91 trials (30.3%) reported both stretching velocity and intensity (Fig. 4d).

Reporting of stretching intensity presented considerable variation, making intertrial comparisons challenging; however, some common trends emerged. For the few cases where intensity was prescribed (or at least reported) for ballistic stretching, it varied from reaching the point of light discomfort (e.g., [181]), to maximum ROM while avoiding pain (e.g., [238]), to extreme ROM (e.g., [174]). Reporting of intensity for dynamic stretching commonly ranged from “through active ROM” (e.g., [97]) to maximum ROM (e.g., [122]), but other descriptions were provided as well (e.g., with slight pain [281], to point of discomfort [247], or from low to high intensity [255]). Static active and passive stretching, as well as PNF, were commonly reported as being performed to certain degrees of discomfort (e.g., to point of mild discomfort [75, 167, 182], to point of discomfort [61, 142, 216]), feeling a stretch (e.g., [154, 222, 285]), or to maximum ROM (e.g., [76, 90, 201]). Often, the request to achieve maximum ROM in static active and passive stretching was followed by qualifiers such as “while avoiding pain” (e.g., [141, 327, 364]). One trial assessed static active stretching to point of discomfort versus to ~ 90% of point of discomfort [117].

Reporting of stretching intensity was not always the same for different stretching modalities within a given trial. Three scenarios occurred: (i) some trials had comparable descriptions of intensity for all included stretching modalities (e.g., maximum ROM while avoiding pain for ballistic stretching, PNF, and static active stretching [238]); (ii) other trials had different descriptions for different stretching modalities (e.g., progressing from moderate to high intensity in dynamic stretching versus maximum ROM for static active stretching [76]); (iii) still other trials specified intensity for one stretching modality, but not for the others (e.g., to point before discomfort for static active stretching but unreported for dynamic stretching [232]). Thus, the requested intensity levels were not always equated (or even reported) between groups or conditions.

The reporting of stretching velocity varied depending on the stretching modality. For example, ballistic stretching was commonly prescribed at a rate of one repetition per second (e.g., [174, 181, 202]), but some descriptions were considerably vaguer (e.g., “in rapid fashion” [331] or “with velocity” [339]). Reporting of dynamic stretching velocity ranged from highly specific information (e.g., 1 repetition every 2 s [79]) to vaguer qualitative descriptions such as “slowly” [96] or “gently” [203], sometimes specifically stating the movements had to be performed without ballistic or abrupt movements [97] or without bouncing [121]. In some cases, there was a progression in velocity (e.g., five repetitions slowly, then ten repetitions quickly [129]). One trial compared dynamic stretching at self-selected speed versus at maximal speed [343].

Stretching velocity was rarely reported for PNF, static active stretching, and static passive stretching, presumably because these modalities tend to be performed at slow velocities by default (e.g., slow progression until reaching maximum ROM, followed by even slower progression to even greater ROM during the stretch). When it was reported, it was usually using the term “slowly” (e.g., [115, 267, 360]) or similar expressions such as “gently” [349] or “smoothly” [318]. A single trial, using static passive stretching, provided an objective measure of stretching velocity, set at 20 degrees per second [187].

#### Within-Trial Inconsistencies in Intervention Volume

There were considerable within-trial inconsistencies that may have compromised the interpretation of results (see ESM Sect. 2.3 for more details). An example emerges from trials that aimed to compare different interventions that were not volume equated (within reasonable limits). As an example, one trial compared 90 s of static active stretching to 20 min of moist heat pack application [176]. Another trial compared 150 s of static active stretching with 750 s of combined static active and dynamic stretching [232].

### Outcome-Level Information

#### Overview of Outcome-Level Information

Complete details of outcome-level features can be found in the ESM (Sect. 2.4) and a summary of the most salient features in Sect. 3.7 of the manuscript (EGM). In summary, five outcome domains were considered: physiological, biomechanical, neural/psychological, performance related, and injury related. No trial assessed outcomes across more than three domains, e.g., physiological, biomechanical, and performance [169, 170]. Biomechanical outcomes were assessed in 54 trials (18.0%), physiological outcomes in 31 (10.3%), and neural/psychological in 26 trials (8.7%). Only five trials (1.7%) assessed injury-related outcomes [e.g., injury incidence, risk ratios (RR)] [87, 99, 105, 177, 312], none of which supported the purported preventive role of stretching. Considering the disparity between the widespread interest on the topic of stretching for injury prevention and the scarcity of studies on the subject (at least with athletes), we provide more in-depth information in the ESM (Sect. 2.5).

**Fig. 5:**
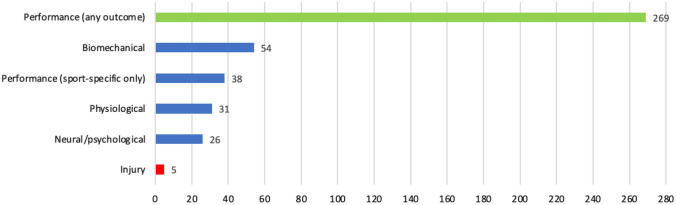
Number of trials assessing each outcome domain

Most trials (*k* = 269, 89.7%) reported performance-related outcomes, mainly focusing on strength/power and ROM (49.7% and 41.0% of trials, respectively), followed by speed (20.3% of trials), and change of direction (COD; 12.3% of trials). All other performance-related outcomes (e.g., balance, endurance, proprioception) were assessed in fewer than 5% of trials. Importantly, sport-specific performance tests were applied in only 38 trials published in 43 studies (12.7%) across 17 sports (most commonly soccer, swimming, artistic gymnastics, and volleyball, in order). Figure 5 synthesizes the research trends for outcome domains.

#### Main Outcomes Assessed Per Domain

Regarding physiological outcomes, blood lactate [115, 203–205, 247, 262, 271, 347, 367] and heart rate [169, 170, 174, 203–205, 208, 247, 248, 287, 315, 347, 349, 353, 354, 357] were the most commonly reported, but a diverse range of other outcomes were reported, such as core temperature [169, 170] and inflammation [60].

Biomechanical outcomes ranged from kinetic and/or kinematic analysis of sport-specific actions (e.g., [64, 206, 253]) to measures of muscle properties such as fascicle length and muscle thickness, among others (e.g., [122, 148, 270]). In this context, 22 trials (7.3%) assessed electromyographic activity, but only two focused on the upper limb [200, 215].

Neural/psychological outcomes including perceived pain [57, 99, 187, 251, 292, 304], soreness [139, 245, 277, 278, 286, 320, 324], and exertion [174, 203–205, 245, 248, 264, 287, 315, 349, 353] were the most commonly reported outcomes in this context, but other outcomes were considered as well (e.g., mood state [245, 347]).

Among performance outcomes, the strength/power-related outcomes (e.g., isokinetic knee flexion and extension [322], medicine ball throw [39]) dominated the research, having been assessed in 149 trials (49.7%). ROM (e.g., sit and reach [331], trunk lateral flexion [343]) was assessed in 123 trials (41.0%), followed by speed (e.g., 15 m sprint [275], curved 55 m sprint [200]) in 61 trials (20.3%). COD (e.g., Illinois Agility Test [63, 326]) was reported in 37 trials (12.3%), and balance (e.g., Star Excursion Balance Test [131, 154]) in 12 trials (4.0%) [53, 55, 65, 98, 120, 131, 154, 200, 252, 276, 289, 301]. Speed endurance (e.g., 6 × 20 m sprints [95]) was assessed in 11 trials published in 12 studies (3.7%) [75, 95, 97, 191, 203–205, 223, 231, 315, 351, 365]. Endurance (e.g., time to exhaustion in supramaximal cycling [247]) was reported in ten trials (3.3%) [137, 233, 247, 248, 342, 349, 352–354, 366]. Proprioception (e.g., knee joint position sense [266, 345]) was reported in six trials (2.0%) [119, 160, 266, 289, 296, 345]. Other outcomes (e.g., global coordination testing [174], functional independence measure score [304], strength endurance [187]) were usually assessed in only one to three trials and had no overall expression.

Sport-specific performance tests were applied in only 38 trials (12.7%) across 17 sports: archery [334], artistic gymnastics [53, 166, 209, 243, 313], badminton [229], baseball [190], Brazilian jiu-jitsu [115], handball [239, 258], fencing [331], ice hockey [288], judo [39], rhythmic gymnastics [145], rowing [286], soccer [67, 69, 174, 182, 189, 210, 252, 305–309], swimming [54, 134, 212, 228, 254, 271], table tennis [199], tennis [184, 238], track and field [283], and volleyball [114, 132, 279–281].

## Evidence Gap Map

**Fig. 6:**
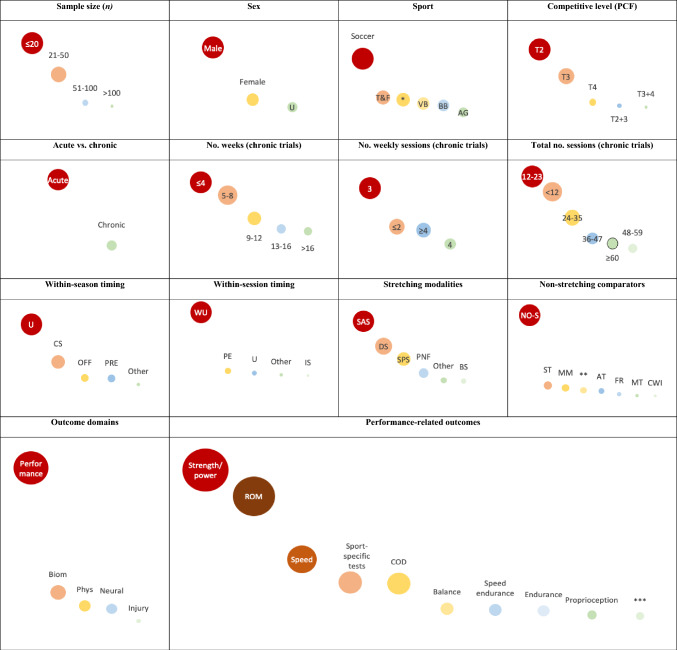
Evidence gap map of stretching research with healthy athletes

Figure 6 shows the EGM that synthesizes the patterns and gaps that were previously identified. Beyond visually conveying the information in a user-friendly manner, some data are shown with a slightly different perspective to avoid redundancy with the previously presented figures.

Inspection of the EGM reveals some key findings: (1) there was an over-abundance of trials with up to 20 participants, but very few large-scale (> 100 participants) trials; (2) most research was performed with male participants, and several trials failed to report on sex; (3) soccer dominated the research efforts, while sports such as artistic gymnastics and martial arts were severely underrepresented; (4) most athletes were tier 2, with scarce research with tier 4 athletes and none with tier 5 athletes; (5) most information derived from acute trials, while chronic trials were mostly ≤ 8 weeks in length and rarely surpassed 16 weeks; (6) trials mostly explored stretching interventions with 3 weekly sessions, with 12–23 total sessions, performed in the context of the warm-up part of the training session; (7) most trials did not report season timing; (8) static active stretching predominated the body of research and was mostly compared with nonstretching passive controls; (9) performance outcomes were very commonly assessed, while biomechanical, physiological, and neural/psychological assessments were less often performed; (10) within performance-related outcomes, trials mostly focused on strength/power and ROM; and (11) only scarce data were available regarding injuries.

## Discussion

Stretching is implemented widespread in multiple sports and different contextual settings. Due to the high number of studies investigating the effects of stretching using different methodologies and protocols, an updated and reliable summary from direct comparisons between studies becomes cumbersome and often challenging. Aiming to guide and inform future research and funding policies, we systematically reviewed the existing literature to map the existing research and identify the trends and current gaps relating to stretching interventions in healthy athletes with a minimum competitive level of tier 2 or higher [52].

### Are All Athletes the Same? Who is Being Studied?

Roughly 7000 athletes were included across the 300 trials, but the median of athletes per trial was 18, and 94.0% of trials had less than 50 athletes. This means that most trials were likely underpowered, and their results lack generalizability, a common problem within the sports science field [369–372]. Such a pattern would be expected if there was a predominance of trials of the most high-level athletes (i.e., tiers 4 and 5), as these populations are statistically small and challenging to enrol in research [52, 372] (being possibly concerned that experimental interventions may disrupt their training routines and, eventually, impair performance). Notwithstanding, there was a complete lack of stretching-related research with tier 5 athletes (a noticeable gap in itself), and research including tier 4 athletes (*k* = 22) represented only 7.3% of all trials. The field of elite sport research faces significant challenges arising from the limited availability of elite athletes as study participants. Longitudinal case studies, ensuring a large number of data points while upholding the fundamental principles that underlie successful clinical trials, could potentially be devised to investigate such high-level athletes [372]. Currently, most knowledge on stretching in athletes is likely limited to underpowered trials and involving nonelite athletes whose results should not be lightly transferred to elite athletes [372]. The results from our scoping review pertain only to the healthy athletic populations (i.e., tier 2 or higher [52]) and should not be extrapolated to other populations, including athletes in rehabilitation contexts.

#### Sex

Typical of sex imbalances in sports sciences publications [373–379], female athletes are underrepresented in the stretching literature (~ 20% versus ~ 65% of male athletes). The missing percentage refers to trials that failed to report sex, potentially meaning they were also male, considering societal biases. The observed disparity in sex representation also aligns with the broader imbalance in samples within the field of sport and exercise psychology [379] and extends to research authorship as well [380]. A discussion of the societal biases that may underlie this phenomenon is beyond the scope of our review, but we strongly support increasing research efforts in female athletes. To rectify this sex-based imbalance, it is crucial for funders, researchers, and journal editors to collaborate actively and diligently toward making significant advancements in addressing this issue.

#### Age

Age was reported very differently across trials, but a rough simplification highlights an age range from 8 to ~ 40 years, and mostly limited to athletes under 30 years of age. Therefore, it can be concluded that research on stretching is mostly focused on adolescents and young adults in their most physically active years and when organized sports participation and engagement in physical activity in general are most common [381, 382]. Notwithstanding, there has been increased participation in sports by older adults, with rising awareness of the specificity of the master athlete in the sports science literature [383–388]. Our scoping review showed that research on stretching in master athletes is largely lacking, and so how these older athletes respond to stretching interventions is currently unclear.

#### Sports

While at least 43 sports were represented (soccer being the most studied), there was a noticeable scarcity of trials performed in sports such as artistic or rhythmic gymnastics, or in martial arts. Stretching, as the most popular exercise modality for improving ROM [3, 4], might be more determinant for performance in some of these sports (e.g., gymnastics, martial arts), where extreme ROM is required [265, 314, 389]. As these types of sports are greatly underrepresented in the stretching literature (as our scoping review has shown), no strong conclusions can be made as to the role of stretching in these sports, and it is unclear whether findings from other sports (e.g., soccer, volleyball) can be extrapolated to gymnastics or martial arts [5]. Even in sports not requiring extreme ROM, there may be important differences in the typical ROMs presented [390], i.e., different sports will pose specific necessities and therefore stretching may play different roles.

### Context Matters! In What Circumstances are Athletes Being Studied?

Over 80% of stretching research in athletic populations focused only on the acute effects. Knowledge about chronic effects of stretching in athletes derives from a much narrower body of research, and no trial lasted more than 21 weeks. Therefore, all knowledge concerning the chronic effects of stretching in athletes derives from trials lasting < 6 months. However, this is an overly optimistic scenario because trials assessing chronic effects lasted a median of 6 weeks, and only 2.0% of trials lasted ≥ 16 weeks. This seems a common limitation within research dealing with other training methods and concepts (e.g., plyometric training [374], periodization [391]). Furthermore, almost 70% of chronic trials implemented ≤ 3 weekly stretching sessions, which may be inferior to common practice in many sports (e.g., artistic gymnastics [392]), especially at higher levels of practice. Possibly, future terminological revisions should consider a category between acute and chronic (e.g., delayed effects?). As explained in Sect. 2, we avoided stipulating an arbitrary temporal threshold for what should be considered a chronic intervention. Regardless, we also feel that trials lasting 1 week or having only three sessions in total should probably not be considered chronic. In summary, there is still a huge knowledge gap about the chronic effects of stretching interventions in athletes, which may result from the extensive resources required and the challenges inherent in performing such longitudinal studies [393].

Season timing may influence the athletes’ fitness status at the time of testing [52, 394]. It may also impact the willingness of athletes to engage in experimental interventions; for example, weekly matches may result in time constraints and concerns about recovery [395, 396], which may influence the weekly contents and workload. Overall, 40% of stretching research with athletes reported the within-season timing. When reported, the competitive season was more common than the off-season and preseason combined. This seems similar to other research training methods (e.g., [397]). Future studies should more consistently report within-season timing.

Most trials (~ 85%) implemented stretching in the context of a warm-up (either in isolation or as a part of the warm-up), denoting a considerable imbalance in the literature and providing a very limited account of stretching effects when applied in other settings. For example, postexercise stretching represented only ~ 7% of all trials. Despite widespread use of [398–401], and support for post-exercise stretching [402, 403], it seems largely ineffective as a recovery method [21, 404], and there is little scientific scrutiny of its effects in athletes. Even less is known about other applications of stretching (e.g., inter-set, at night before falling asleep), representing a major gap in research.

### All Stretching is Not the Same: What Stretching Modalities are Being Implemented?

According to the literature, static active, static passive, dynamic, ballistic, and PNF are the most commonly used stretching methods [1, 3]. Stretching research with healthy athletes is dominated by static active stretching, represented in > 60% of trials, followed by dynamic stretching (< 40% of trials). Of note, trials could implement multiple stretching modalities and doses. Static passive stretching and PNF (mostly limited to the contract–relax method) were analyzed in only ~ 25% and 12% of trials, respectively, and ballistic stretching represented less than 5% of trials. This means that further research is required to better understand the effects of static passive stretching, PNF, and ballistic stretching in athletes. It is possible that static passive stretching and PNF may play a more determinant role in performance in sports requiring extreme ROM (e.g., gymnastics, martial arts), and this literature gap may therefore differentially affect distinct sports.

Alternative stretching methods (e.g., SGA) are being largely neglected by research, which does not benefit scientific advances and fails to either support or recommend against their application. The effects of combining stretching with heat, cold, or vibration superimposed on the stretches are also largely unexplored. Finally, ~ 75% of the trials applied stretching to the lower limbs, with much less information being available concerning the trunk and the upper limbs. This is problematic, as the upper limbs and the trunk play a major role in several sports (e.g., handball, throwing-based track and field events, volleyball, and weightlifting, among others). The existing imbalance in the body of knowledge concerning the primary areas of intervention raises concerns about the potential generalization of evidence that is specifically researched. To mitigate this issue, it is crucial to foster a greater proportion of research in less explored domains and to incorporate an analysis of anatomical variability. Such an approach would shed light on the diverse mechanisms of adaptation and underscore the significance of considering case studies that reflect this variability.

### Everything May be Superior to Nothing: What Comparisons are Being Performed?

Beyond comparisons between different stretching modalities and doses, more than half of the trials included a no-stretching control group that otherwise was subjected to the same procedures as the stretching groups. This is highly relevant to ascertain the effects of stretching compared with individuals who were maintained at rest (i.e., passive controls), and can be used to justify stretching. However, alternative interventions may show equal or superior efficacy or effectiveness (e.g., strength training versus stretching for small ROM improvements [24, 405]). Moreover, such relative effectiveness is likely to vary depending on the specific outcome being assessed and with potential varying adherence. Unfortunately, only ~ 25% of all trials included active, nonstretching-related comparators (e.g., FIFA 11 +), or stretching added to some other intervention not included in the stretching-only groups (e.g., stretching followed by vibration foam rolling). These trials were spread across more than 15 classes of comparators (e.g., foam rolling, aerobic training), and within each class there were considerable differences in the interventions. Moreover, these interventions were often not equated for volume. Therefore, systematic comparisons between stretching and alternative interventions are lacking, with some exceptions regarding strength training and multimodal exercise programs.

### How Much? Is Dose-Response Being Scrutinized?

Volume, intensity, and weekly frequency may strongly influence the effects of stretching [15]. However, less than 8% of all trials compared different doses of stretching (usually, through manipulation of duration, number of repetitions and/or number of sets), and only one trial compared different intensity thresholds [117]. Overall, the number of stretches varied widely (1–22), occasionally even between two stretching groups within the same trial (e.g., [232]). The same was true for the number of sets (1–32), number of repetitions (3–30), time per set (5–900 s), rest intervals (0–120 s), and, consequently, in the minimum stretching volume per session (5–420 repetitions; 7–2880 s). Information concerning stretching velocity and intensity was reported very inconsistently across and within trials, and only less than ~ 33% of all trials provided sufficient information to assess both velocity and intensity. Although some protocol variability is required to search for the most suitable stretching protocol(s), there is also the need for replicability and reproducibility. Likewise, equalizing the volume between stretching methods is challenging but needed. Perhaps time under tension could be applied, but it is difficult to assess this variable in dynamic or ballistic stretching as the tension is not uniform during the course of the movement. More research is required to better understand how to properly equate training volume when comparing different stretching interventions.

As a result, there is a paucity of information pertaining to dose–response relationships in the context of implementing stretching protocols for athletes, despite the importance of understanding such relationships to more appropriately design and prescribe exercise interventions [406–410]. Relatedly, it is further imperative to incorporate the concept of individualization into training practices and consider the impact of human variability when addressing dose–response relationships [411–414]. However, our understanding of this individualized training approach remains limited, necessitating the establishment of new research avenues to explore this direction comprehensively.

### Are We Looking for the Most Relevant Outcomes? What Has the Literature Assessed?

Physiological outcomes were assessed in only ~ 10% of trials, (most commonly, blood lactate and heart rate), and the same applies to neural and/or psychological outcomes (commonly perceived pain, perceived soreness, and perceived exertion). More information is available on how stretching affects biomechanical outcomes, but less than 20% of trials reported such outcomes. The limited reporting on physiological, neural/psychological, and biomechanical outcomes precludes a robust understanding of the mechanisms underlying changes in the commonly assessed performance outcomes, which affects knowledge on causal relationships and thus provides limited information regarding the optimization of training prescription [415].

Performance outcomes were reported in ~ 90% of trials. However, most reporting referred to strength/power and ROM, with less information available concerning speed and COD (~ 10 to 20% of trials). Outcomes reported in less than 5% of trials included balance, speed endurance, endurance, and proprioception, among others. The predominance of strength/power outcomes was expected, as these constructs are strongly associated with performance in athletes (e.g., [416–419]), while ROM is perhaps the most obvious outcome to check when implementing stretching interventions. What is surprising is the very limited exploration of how stretching affects other parameters such as balance, endurance, or proprioception. Furthermore, sport-specific performance tests were applied in < 15% of trials. Currently, most knowledge on performance-related effects of stretching derives from general tests (e.g., 20 m sprint, 1 RM strength tests, sit and reach) that are transversal but lack specificity, with much less being known about the effects of stretching on sport-specific performance.

A glaring gap is the lack of trials investigating the effects of stretching on injury prevention/injury risk reduction. We identified a lacklustre total of five trials (mostly chronic postexercise stretching interventions with male athletes) that assessed injury (i.e., that provided data on injury incidence, prevalence, or risk, instead of relying exclusively on surrogate measures of injury risk, such as strength). These trials failed to support the notion that stretching reduces injury risk and, despite being limited to athlete tiers 2 or higher, align well with the results of several reviews on the topic [4, 16, 17, 420–425]. Of note, not all reviews on the topic reach the same conclusions [426], and these reviews were not necessarily limited by the minimum PCF tier 2. This is perhaps the most striking gap in our knowledge, and probably should be the focus of ample research investment in the near future.

Additional opportunities for research with athletes (some of which have started being scrutinized in different populations) would include exploration of the effects of stretching on venous and lymphatic circulation [427, 428], nonlocal effects of stretching [13, 14], and the effect of previous knowledge/expectations on the efficacy of stretching interventions [429].

### What Lies Ahead? Priorities for Future Research

**Fig. 7:**
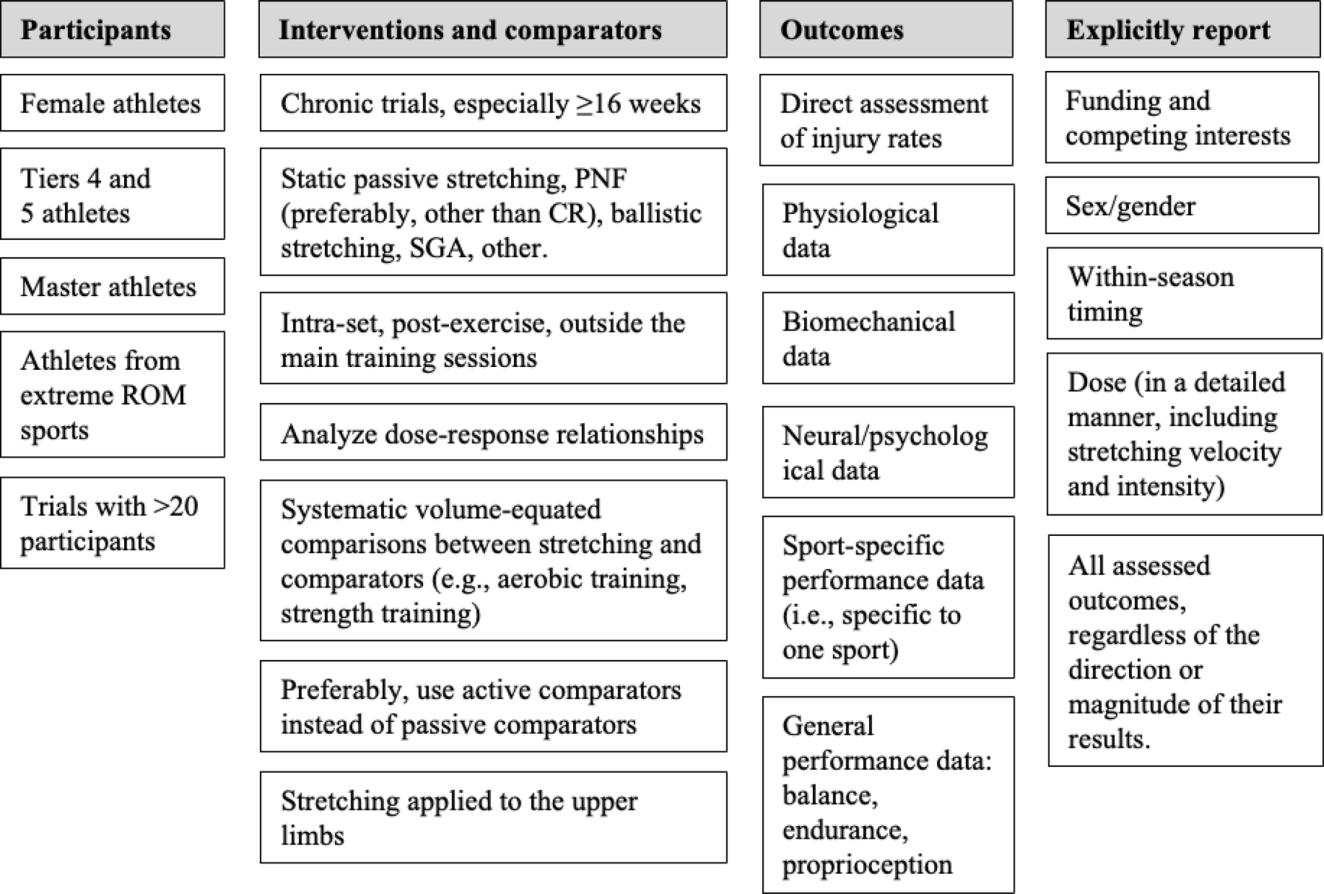
Research priorities regarding stretching interventions with athletes. *CR* contract–relax, *PNF* proprioceptive neuromuscular facilitation, *ROM* range of motion, *SGA* global active stretching (from the French original *Stretching Global Actif*)

Based on the most relevant gaps that were identified, Fig. 7 shows suggested research priorities. This summary provides opportunities for funders and researchers to focus on less researched areas of stretching, while potentially eliminating wasteful research on further investigating topics that are already well researched. We also propose that more funding is provided for the development of stretching-based research in African countries, as research on the topic performed in this continent is mostly limited to Tunisia. Additionally, any information concerning funding and competing interests should be mandatory, and all journals should define them as a prerequisite for publication. Finally, we highlight the need to implement efforts to avoid selective reporting of outcomes, which may bias not only the original research findings, but also future reviews on selected topics. Preregistration of experimental studies is highly advisable.

### Limitations

By focusing solely on athletes and establishing the PCF’s tier 2 as the minimum for inclusion, it is possible that other well-trained populations have been left out (e.g., regular gym goers engaging in noncompetitive CrossFit or bodybuilding, dancers). However, a threshold had to be established, and participation in competitions was deemed necessary to use the term “athlete.” Regardless, the sample of 316 published studies showed trends that would likely remain robust even if some extra trials had been considered. The exclusion of injured athletes precludes us from making any statements regarding the role of stretching in injury rehabilitation, while the exclusion of athletes with disabilities inhibits any statements concerning the status of stretching research in these populations. However, these specific populations were outside the scope we intended for our scoping review. Interpreting the results of the included trials should be moderated by knowledge that 66.0% did not provide a published funding statement (i.e., whether there was funding or not) and 65.6% did not provide a published statement pertaining competing interests.

### Should We Reconsider the Terminology and Description of Stretching Exercises?

An additional limitation that may impact the findings deserves to be highlighted. Commonly, static stretching is deemed passive if an external agent (e.g., coach, teammate) applies the stretch to the athlete, and our scoping review followed that logic and terminology to facilitate an intuitive understanding for most readers. However, future discussions on the terminology should be considered, as in many of the so-called active stretching exercises the athletes used a part of their body to stretch another part, or they used external surfaces (e.g., walls). Therefore, those exercises would technically have a passive component. Moreover, in such cases, it was unclear whether the “self-passive” assistance was used from the beginning of the stretching until reaching the desired endpoint, or if it was used exclusively near or at the endpoint. It was further unclear how long each phase lasted, i.e., whether the stretching was mostly active with a slight passive component or the reverse. We encourage the scientific community to engage in a debate surrounding the terminology, and perhaps less commonly used taxonomy such as self-stretching [91] is appropriate. Within the concept of self-stretching, authors should carefully describe the details of each exercise, including an estimate of the role played by the active and passive phases (when applicable). The fact that so-called static active stretching may, in fact, include a considerable passive phase could impact the interpretation of findings from stretching studies.

## Conclusions

There are noticeable gaps in stretching research in athletic populations, precluding a thorough knowledge of its effects. Some problems are common to most research in sports sciences (e.g., small samples; poor representation of females, master athletes, and tiers 4 and 5 athletes; lack of long-term chronic trials; scarce exploration of dose–response relationships), but there are additional relevant gaps. Most evident is the negligible number of trials assessing the effects of stretching on injury rates (and those that exist do not support a preventive effect). This refers specifically to trials containing explicit information to allow considering the participants as tier 2 or higher, and so may not be directly comparable to most reviews on the topic, which usually have broader eligibility criteria, including participants below tier 2. Therefore, the possibility of stretching reducing overall or specific (e.g., musculotendinous) injury risk in athletes (tier 2 or higher) requires more extensive research.

Furthermore, the outcomes assessed in the included trials have been largely limited to general performance tests, with reduced exploration of sport-specific performance tests and mechanistic assessments (e.g., biomechanical, physiological). Also relevant is the scarcity of trials with participants from sports demanding extreme ROM (e.g., gymnastics), and the underrepresentation of static passive stretching, PNF, and especially ballistic stretching.

Currently, most knowledge regarding applications of stretching with athletes derives from underpowered trials of nonelite athletes, assessing the acute effects of static active or dynamic stretching applied to the lower limbs compared with passive controls, and mostly performed in the context of a warm-up. This field of research seems to be limiting itself, focusing on a very narrow range of possibilities and therefore providing only a limited window for stretching and its potential effects in athletes. We advise a change in research priorities, policies, and funding, focusing future research on fulfiling the extensive existing gaps.

### Supplementary Information

Below is the link to the electronic supplementary material.

Supplementary file1 (DOCX 727 KB)

## Declarations

### Registration and protocol

The protocol was created (https://osf.io/6auyj/) and preregistered (https://osf.io/gu8ya) as an Open Science Framework (OSF) project and made public on 14 July 2022 (1 day before the initial database searches). We originally designed this scoping review as a living review. However, considering the amount of work involved (an extremely large number of records in the searches and a very large number of trials included in the review), our team is currently not able to guarantee future updates.

### Data availability

The data used to inform this review are fully disclosed either in the manuscript or in its Electronic Supplementary Material.
