# Supplementary material for: What We Do Not Know About Stretching in Healthy Athletes: A Scoping Review with Evidence Gap Map from 300 Trials
Source: Sports Med. 2024 Mar 8;54(6):1517–51. doi: 10.1007/s40279-024-02002-7 (PMC11239752; doi:10.1007/s40279-024-02002-7)
Supplement: Supplementary file 1 — Supplementary file1 (DOCX 727 KB) [file 40279_2024_2002_MOESM1_ESM.docx]

**Title:** What don’t we know about stretching in healthy athletes: A scoping review with evidence gap map from 300 trials.

**Electronic supplementary material**

José Afonso^1^, Renato Andrade^2,3,4^, Sílvia Rocha-Rodrigues^5,6^, Fábio Yuzo Nakamura^7^, Hugo Sarmento^8^, Sandro Freitas^9^, Ana Filipa Silva^5,10,11^, Lorenzo Laporta^12^, Maryam Abarghoueinejad^13^, Zeki Akyildiz^14^, Rongzhi Chen^1^, Andreia Pizarro^15,16^, Rodrigo Ramirez-Campillo^17^, Filipe Manuel Clemente^5,10,18^

1 – Centre of Research, Education, Innovation, and Intervention in Sport (CIFI_2_D), Faculty of Sport, University of Porto, Portugal. [jneves@fade.up.pt](mailto:jneves@fade.up.pt) (JA); [up202110014@edu.fade.up.pt](mailto:up202110014@edu.fade.up.pt) (RC)

2 – Clínica Espregueira - FIFA Medical Centre of Excellence, Porto, Portugal. [randrade@espregueira.com](mailto:randrade@espregueira.com) (RA)

3 – Dom Henrique Research Centre, Porto, Portugal. [randrade@espregueira.com](mailto:randrade@espregueira.com) (RA)

4 – Porto Biomechanics Laboratory (LABIOMEP), University of Porto, Portugal. [randrade@espregueira.com](mailto:randrade@espregueira.com) (RA)

5 – Escola Superior de Desporto e Lazer, Instituto Politécnico de Viana do Castelo, Rua Escola Industrial e Comercial de Nun’Alvares, 4900-347 Viana do Castelo, Portugal. [silviars@esdl.ipvc.pt](mailto:silviars@esdl.ipvc.pt) (SRR); [anafilsilva@gmail.com](mailto:anafilsilva@gmail.com) (AFS); [filipe.clemente5@gmail.com](mailto:filipe.clemente5@gmail.com) (FMC)

6 - Tumour & Microenvironment Interactions Group, INEB - Institute of Biomedical Engineering, i3S-Instituto de Investigação e Inovação em Saúde, Universidade do Porto, Rua Alfredo Allen, 4200-153 Porto, Portugal. [silviars@esdl.ipvc.pt](mailto:silviars@esdl.ipvc.pt)

7 – Research Center in Sports Sciences, Health Sciences and Human Development (CIDESD), University of Maia, Maia, Portugal. [fabioy_nakamura@yahoo.com.br](mailto:fabioy_nakamura@yahoo.com.br)

8 – University of Coimbra, Research Unit for Sport and Physical Activity (CIDAF), Faculty of Sport Sciences and Physical Education, Coimbra, Portugal. [hg.sarmento@gmail.com](mailto:hg.sarmento@gmail.com)

9 – Neuromuscular Research Lab, Faculdade de Motricidade Humana, Universidade de Lisboa, Portugal. [sfreitas@fmh.ulisboa.pt](mailto:sfreitas@fmh.ulisboa.pt)

10 – Research Center in Sports Performance, Recreation, Innovation and Technology (SPRINT), 4960-320 Melgaço. [anafilsilva@gmail.com](mailto:anafilsilva@gmail.com) (AFS); [filipe.clemente5@gmail.com](mailto:filipe.clemente5@gmail.com) (FMC)

11 – Health Sciences and Human Development (CIDESD), The Research Centre in Sports Sciences, 5001-801 Vila Real, Portugal. [anafilsilva@gmail.com](mailto:anafilsilva@gmail.com) (AFS)

12 – Núcleo de Estudos em Performance Analysis Esportiva (NEPAE/UFSM), Universidade Federal de Santa Maria. Avenida Roraima, nº 1000, Cidade Universitária, Bairro Camobi, Santa Maria, RS, CEP: 97105-900, Brazil. [Laporta.lorenzo@ufsm.br](mailto:Laporta.lorenzo@ufsm.br) (LL)

13 – Independent researcher. [m.abarghouei.n@gmail.com](mailto:m.abarghouei.n@gmail.com) (MA)

14 – Gazi University, Training and Movement Science, Ankara, Turkey. [zekiakyldz@hotmail.com](mailto:zekiakyldz@hotmail.com) (ZA)

15 – Research Center in Physical Activity, Health and Leisure (CIAFEL), Faculty of Sport, University of Porto, Portugal. [anpizarro@fade.up.pt](mailto:anpizarro@fade.up.pt) (AP)

16 – Laboratory for Integrative and Translational Research in Population Health (ITR), Rua das Taipas, 135, 4050-600 Porto, Portugal. [anpizarro@fade.up.pt](mailto:anpizarro@fade.up.pt) (AP)

17 – Exercise and Rehabilitation Sciences Institute. School of Physical Therapy. Faculty of Rehabilitation Sciences. Universidad Andres Bello. Santiago, 7591538, Chile. [rodrigo.ramirez@unab.cl](mailto:rodrigo.ramirez@unab.cl) (RRC)

18 – Gdańsk University of Physical Education and Sport, 80-336 Gdańsk, Poland. [filipe.clemente5@gmail.com](mailto:filipe.clemente5@gmail.com) (FMC)

**ESM 1. Methods**

***ESM 1.1. Information sources***

A four-step approach was used to collect the data:

1. Automated searches were performed on July 15, 2022 (*i.e.*, after registration of the protocol) and updated on January 1^st^, 2023, in the following databases: CINAHL, EMBASE, PubMed, Scopus, SPORTDiscus and Web of Science (core collection), with no filters or limits applied.
2. If the number of initially included studies was not enough to provide representative data on past and current trends in this field (*i.e.*, <250 studies ^[[1]](#footnote-1)^), with further studies likely making an impact in our results, manual searches would be performed within the reference lists of the studies deemed eligible for inclusion after the automated searches. We would also select representative systematic reviews on the topic of stretching and check their reference list. Since we included >300 studies, this step was not performed.
3. Accordingly, the list of included studies and the inclusion criteria were not sent to external experts.
4. Upon having the final list of included studies, we searched for errata and retractions [1], as well as retrieved pre-registered or pre-published protocols and supplementary files, when available.

***ESM 1.2. Search strategy***

Free text terms were searched using Boolean operators applied to the title or abstract:

(i) stretch* OR flexi* OR "range of motion" OR "proprioceptive neuromuscular facilitation" OR gyrotonic;

AND

(ii) athlet* OR sport* OR player* OR gymnast* OR balle* OR danc* OR "martial art*" OR baseball OR basket* OR cycling OR boxing OR cricket OR football OR golf* OR hockey OR mountaineering OR racquet OR tennis OR rugby OR runn* OR skat* OR skiing OR soccer OR "track and field" OR volleyball* OR "Nordic walking" OR swim* OR "weight lifting" OR weightlifting OR wrestl*;

The resulting code lines for each database are presented in supplementary table 1. A brief explanation is warranted regarding the second code line, as it would be impossible to include all existing sports. Therefore, we combined three strategies: (i) generic terms such as athlet*, sport* and player*; (ii) terms capturing sports to which stretching is traditionally related (*e.g.*, martial arts, dancing); and (iii) sports included as MeSH terms in the NIH National Library of Medicine, except those already encompassed by the first two sets of terms (<https://www.ncbi.nlm.nih.gov/mesh/68013177>).

**Supplementary table 1.** Resulting code lines for each database

| **Database** | **Specificities of each database** | **Code lines generated by the database after performing the searches** |
| --- | --- | --- |
| CINAHL | Nothing to report. | (TI (stretch* OR flexi* OR 'range of motion' OR 'proprioceptive neuromuscular facilitation' OR gyrotonic) OR AB (stretch* OR flexi* OR 'range of motion' OR 'proprioceptive neuromuscular facilitation' OR gyrotonic)) AND (TI (athlet* OR sport* OR player* OR gymnast* OR balle* OR danc* OR "martial art*" OR baseball OR basket* OR cycling OR boxing OR cricket OR football OR golf* OR hockey OR mountaineering OR racquet OR tennis OR rugby OR runn* OR skat* OR skiing OR soccer OR "track and field" OR volleyball* OR "Nordic walking" OR swim* OR "weight lifting" OR weightlifting OR wrestl*) OR AB (athlet* OR sport* OR player* OR gymnast* OR balle* OR danc* OR "martial art*" OR baseball OR basket* OR cycling OR boxing OR cricket OR football OR golf* OR hockey OR mountaineering OR racquet OR tennis OR rugby OR runn* OR skat* OR skiing OR soccer OR "track and field" OR volleyball* OR "Nordic walking" OR swim* OR "weight lifting" OR weightlifting OR wrestl*)) |
| EMBASE | Nothing to report. | (stretch*:ab,ti OR flexi*:ab,ti OR 'range of motion':ab,ti OR 'proprioceptive neuromuscular facilitation':ab,ti OR gyrotonic:ab,ti) AND (athlet*:ab,ti OR sport*:ab,ti OR player*:ab,ti OR gymnast*:ab,ti OR balle*:ab,ti OR danc*:ab,ti OR 'martial art*':ab,ti OR baseball:ab,ti OR basket*:ab,ti OR cycling:ab,ti OR boxing:ab,ti OR cricket:ab,ti OR football:ab,ti OR golf*:ab,ti OR hockey:ab,ti OR mountaineering:ab,ti OR racquet:ab,ti OR tennis:ab,ti OR rugby:ab,ti OR runn*:ab,ti OR skat*:ab,ti OR skiing:ab,ti OR soccer:ab,ti OR 'track and field':ab,ti OR volleyball*:ab,ti OR 'nordic walking':ab,ti OR swim*:ab,ti OR 'weight lifting':ab,ti OR weightlifting:ab,ti OR wrestl*:ab,ti) |
| PubMed | Nothing to report. | (stretch*[Title/Abstract] OR flexi*[Title/Abstract] OR "range of motion"[Title/Abstract] OR "proprioceptive neuromuscular facilitation"[Title/Abstract] OR gyrotonic[Title/Abstract]) AND (athlet*[Title/Abstract] OR sport*[Title/Abstract] OR player*[Title/Abstract] OR gymnast*[Title/Abstract] OR balle*[Title/Abstract] OR danc*[Title/Abstract] OR "martial art*"[Title/Abstract] OR baseball[Title/Abstract] OR basket*[Title/Abstract] OR cycling[Title/Abstract] OR boxing[Title/Abstract] OR cricket[Title/Abstract] OR football[Title/Abstract] OR golf*[Title/Abstract] OR hockey[Title/Abstract] OR mountaineering[Title/Abstract] OR racquet[Title/Abstract] OR tennis[Title/Abstract] OR rugby[Title/Abstract] OR runn*[Title/Abstract] OR skat*[Title/Abstract] OR skiing[Title/Abstract] OR soccer[Title/Abstract] OR "track and field"[Title/Abstract] OR volleyball*[Title/Abstract] OR "Nordic walking"[Title/Abstract] OR swim*[Title/Abstract] OR "weight lifting"[Title/Abstract] OR weightlifting[Title/Abstract] OR wrestl*[Title/Abstract]) |
| Scopus | Title, abstract and keywords. | ( TITLE-ABS-KEY ( stretch* OR flexi* OR "range of motion" OR "proprioceptive neuromuscular facilitation" OR gyrotonic ) AND TITLE-ABS-KEY ( athlet* OR sport* OR player* OR gymnast* OR balle* OR danc* OR "martial art*" OR baseball OR basket* OR cycling OR boxing OR cricket OR football OR golf* OR hockey OR mountaineering OR racquet OR tennis OR rugby OR runn* OR skat* OR skiing OR soccer OR "track and field" OR volleyball* OR "Nordic walking" OR swim* OR "weight lifting" OR weightlifting OR wrestl* ) ) |
| SPORTDiscus | Nothing to report. | (TI (stretch* OR flexi* OR 'range of motion' OR 'proprioceptive neuromuscular facilitation' OR gyrotonic) OR AB (stretch* OR flexi* OR 'range of motion' OR 'proprioceptive neuromuscular facilitation' OR gyrotonic)) AND (TI (athlet* OR sport* OR player* OR gymnast* OR balle* OR danc* OR "martial art*" OR baseball OR basket* OR cycling OR boxing OR cricket OR football OR golf* OR hockey OR mountaineering OR racquet OR tennis OR rugby OR runn* OR skat* OR skiing OR soccer OR "track and field" OR volleyball* OR "Nordic walking" OR swim* OR "weight lifting" OR weightlifting OR wrestl*) OR AB (athlet* OR sport* OR player* OR gymnast* OR balle* OR danc* OR "martial art*" OR baseball OR basket* OR cycling OR boxing OR cricket OR football OR golf* OR hockey OR mountaineering OR racquet OR tennis OR rugby OR runn* OR skat* OR skiing OR soccer OR "track and field" OR volleyball* OR "Nordic walking" OR swim* OR "weight lifting" OR weightlifting OR wrestl*)) |
| Web of Science | Title, abstract and keywords (termed “Topic”). | stretch* OR flexi* OR "range of motion" OR "proprioceptive neuromuscular facilitation" OR gyratonic (Topic) and athlet* OR sport* OR player* OR gymnast* OR balle* OR danc* OR "martial art*" OR baseball OR basket* OR cycling OR boxing OR cricket OR football OR golf* OR hockey OR mountaineering OR racquet OR tennis OR rugby OR runn* OR skat* OR skiing OR soccer OR "track and field" OR volleyball* OR "Nordic walking" OR swim* OR "weight lifting" OR weightlifting OR wrestl* (Topic) |

***ESM 1.3. Data items***

Data was extracted within six domains:

1. Participant-related information:
   1. sample size, age, sex^[[2]](#footnote-2)^, sport;
   2. competitive level was reported according to the Participant Classification Framework [4].
2. Intervention-related information:
   1. focused on acute effects versus chronic adaptations (in the later, intervention length will be retrieved);
   2. stretching modalities (static, dynamic, proprioceptive neuromuscular facilitation [PNF], others), dose (*e.g.,* duration, repetitions, sets, intensity, frequency) and timing (pre- or post-exercise, inter-set stretching, others);
   3. cointerventions in the comparator groups (*i.e.,* if stretching is coupled with other interventions, such as massage or foam rolling) [as defined in eligibility criteria, the *interventions* could not be multimodal, but the *comparators* could].
3. Comparators (when available):
   1. other stretching modalities (*i.e.*, comparisons between different stretching modalities);
   2. the same stretching modality but with different doses or timings;
   3. alternative interventions (*e.g.,* endurance training, strength training);
   4. passive controls.
4. Outcomes:
   1. physiological (blood lactate kinetics, blood creatine kinase [CK] concentration, among others);
   2. biomechanical (*e.g.*, muscle and/or tendon stiffness, ground reaction forces [GRF]);
   3. neural/psychological^[[3]](#footnote-3)^ (*e.g.,* perceived well-being, rate of perceived exertion [RPE]);
   4. performance-related (*e.g.,* countermovement jump [CMJ], range of motion [ROM]):
      1. performance-related outcomes were also grouped according to their factor emphasis (*e.g.,* strength, ROM), to provide an overview of which categories are being assessed;
      2. considering the goal of providing a scoping review with evidence map, outcomes were registered, but their results were not. For example, we registered that *k* studies assessed the CMJ, but the actual results of the effects of stretching on CMJ were irrelevant for our purposes.
5. Study design-based evidence-level:
   1. a color coding was used to denote randomized (green), non-randomized multi-arm/condition (yellow), and single-arm/condition studies (red);
   2. considering the purposes of this scoping review, analyzing the risk of bias in studies would not impact our results or the assessment of research trends [5].
6. Context of intervention:
   1. within-season timing (*e.g.,* pre-season, competitive phase);
   2. within-session timing (*e.g.*, warm-up, post-exercise);
   3. geographical location (country, continent).
7. Additional information:
   1. date of publication;
   2. funding details;
   3. competing interests.

***ESM 1.4. Data management***

Multiple publications from a single trial were grouped for all data analysis purposes. Since studies only analyzed the subjects that completed the interventions and/or assessments, we reported the number of subjects analyzed, which did not always coincide with the sample size reported by the authors in locations such as the abstract. Age was reported as mean and standard deviation (rounded to one decimal case where possible, *i.e.*, if the authors only reported units with did not infer decimal places) or, in case this information was not available, we reported the range. The priority was to report the age of the sample as whole, and so age-related data was only reported separately for the several groups of a study in case the authors did not provide global data.

To convey a reader-friendly manuscript, we adopted a unified terminology, which often meant changing the terminology originally used by the authors. For example, we adopted a broad classification of stretching into static active, static passive, dynamic, ballistic, PNF (among other minor, very specific interventions) [6]. Hence, while some authors may have referred to unassisted/non-assisted/auto-stretching or assisted stretching [e.g., 7, 8-10], we report such methods as static active and static passive stretching, respectively. Where authors only reported “static stretching”, without specifying if passive or active, we tried to obtain that information through the description of the exercises and/or existing figures. Also for purposes of categorization, neural stretching and neural sliding [11, 12] were considered a form of dynamic stretching, while passive neurodynamic nerve gliding [13] was considered a form of static passive stretching.

For interventions, we merely stated what differed between the intervention(s) and comparator(s). For example, usually all the groups within a study performed a standardized warm-up prior to the interventions, which was not reported in our review.

Regarding the within-session timing, we considered warm-up, inter-set and post-exercise stretching. A category of independent sessions was applied only to full stretching sessions outside the main training sessions; if the protocol involved brief warm-up, followed by stretching, followed by testing, it was considered within the broad category of warm-up.

The minimum stretching volume per session was calculated multiplying the number of stretches by the number of sets and repetitions/time but excluding rest time. The term “minimum” was purposefully used to account for poor reporting (*e.g.*, in some trials, it was unclear whether the stretching exercises were bilateral or unilateral, which interferes with the exposure time). This value could not be assessed if the authors failed to report the number of stretching exercises, number of sets, and number of repetitions or time. Occasionally, the authors reported a total time for the stretching intervention, but without reporting the duration of the rest intervals it was difficult to assess the stretching volume per session, especially if information on the execution time of each stretch exercise was unclear (*e.g.*, [14]).

Pertaining the outcomes, we also provided some simplifications. For example, tests that are usually applied to assess speed (e.g., 20-m sprint) can also be used to assess acceleration and other derived data, but we referred to them as tests for speed. Similarly, strength and power-based tests (e.g., bench press 1 repetition maximum [1RM], CMJ) may be used to derive measures such as torque and jerk but were categorized as strength/power. With so many studies included, such simplifications are expected to help providing a clearer picture of what categories of outcomes are being applied in research on stretching applied to athletes. Finally, many studies reported the application of agility tests, but in fact only assess change of direction (COD), which is only one component of agility and does not represent agility as a whole [15-17].

**ESM 2. Results**

***ESM 2.1. Study selection***

Initial database searches retrieved a total of 198,725 records, of which 81,555 were duplicates. Screening of 117,170 records resulted in the exclusion of 116,725 records (due to article type or PICOS [Participants, Interventions, Comparators, Outcomes, Study Design]). We could not retrieve the full text for three studies, which where therefore excluded. Full-text analysis was performed for 442 potentially eligible studies, of which 309 studies were deemed eligible for inclusion and 133 were excluded (1 due to article type and 132 due to PICOS; studies and reasons for exclusion are available in a supplementary file: <https://osf.io/u2dxw>).

Updated searches were performed on January 1^st^, 2023: 20,684 further records were retrieved, of which 8,092 were duplicates and 6,069 had already appeared in the initial searches. Screening of the new 6,523 records resulted in 6,510 being excluded. Full-text analysis of 13 studies resulted in the exclusion of six studies. Eliminated studies and reasons for their exclusion during the updated searches can be consulted in a supplementary file: <https://osf.io/rvdqz>). With the inclusion of seven additional studies in the updated searches, a total of 316 studies were included in the review, corresponding to 300 trials (i.e., one or more trials resulted in two or more published studies) [7-12, 18-250][13, 14, 251-325]^[[4]](#footnote-4)^;

***ESM 2.2. Publication-, participant-, and context-level details***

*ESM 2.2.1. Age*

Age was unreported in four trials (1.3%) [116, 137, 160, 194]. One trial reported an age of 24.6±5.0 years [229], but this included a group with 10 controls that failed our eligibility criteria and were not considered in our analysis. Similarly, one trial reported an age of 20.0±1.5 years [295], but this included 42 recreational athletes beyond the 18 collegiate players, with the former not abiding by our eligibility criteria. Another trial reported an age of 19.3±1.2 years, but this included the original 24 recruited athletes, of which 12 dropped out [91]. One trial only stated that young athletes were between 14 and 15 years and adult athletes were ≥19 years of age [200]. One trial merely stated the athletes were Under-19 [278], and another only specified they were high-school students [157].

Trials reported age in the form of mean ± standard deviation (SD) (e.g., [177, 231]), mean and range (e.g., [52, 302]), range alone (e.g., [287, 316]), mean alone [22, 217], or median and interquartile range (IQR) [75]. Some trials reported the age for the entirety of the sample (e.g., [77, 84]), others reported age only for each specific group, according to intervention (e.g., [57, 62]) or sex (e.g., [289, 308]). A single trial reported age in the form of median (14.5) and IQR (12.5-16.5) [75].

Age was reported in the form of mean±SD in 264 trials (88.0%). Most commonly, SDs were relatively small in comparison with the mean (e.g., 18.5±0.6 [320], *i.e.*, SD equals 3.2% of the mean), suggesting that distribution curves were likely normal and hence the sample was homogeneous regarding age. However, this was not always the case, especially with older samples. For example, one trial had three groups (longitudinal stretching, transversal stretching, and controls), with the SD corresponding to 20.6%, 22.4% and 26.9% of the mean, respectively.

*ESM 2.2.2. Length of interventions*

One trial had insufficient information to assess whether it was acute or chronic [88], while four trials/five studies (1.3%) assessed both acute and chronic effects [152, 177, 178, 255, 284]. One trial did not provide sufficient information to assess study length or number of weekly sessions, having only provided the total number of sessions (16) [221].

**Supplementary table 2.** Publication-, participant-, and context-level details

| **Trials (🟢🟡🔴)**  **(Language, Country, Continent)** | **Sample size (*N)***  ***Sex: n (%)***  ***Age*** *(mean±SD except otherwise stated)* | **Sport(s)** | **PCF** | **Funding** | **Competing interests** | **Intervention length**  *If chronic (no. weeks, no. weekly sessions, total no. sessions)*  **Within-season timing**  **Within-session timing** |
| --- | --- | --- | --- | --- | --- | --- |
| Abadi et al. [18] **🟢**  (Persian, Iran, Asia) | 24  ♀ (100%)  9.7±1.4 | Artistic Gymnastics | Tier 2 | Unreported | Unreported | Acute  Unreported  Warm-up |
| Agopyan et al. [19] **🟢**  (English, Turkey, Europe and Asia) | 29  ♂: 15 (51.7%); ♀: 14 (48.3%)  ♂: 11.7±0.4; ♀: 11.6±0.5 | Swimming | Tier 2 | Unreported | Unreported | Acute  Unreported  Warm-up |
| Ahmadabadi et al. [20] **🟢**  (Persian, Iran, Asia) | 16  ♀ (100%)  9.6±1.5 | Artistic Gymnastics | Tier 2 | Unreported | Unreported | Chronic (4 weeks, 3x/week, 12 sessions)  Unreported  Warm-up |
| Akarsu et al. [21] **🟡**  (English, Turkey, Europe and Asia) | 21  Unreported  16.0±0.9 | Taekwondo | Tier 2 | Unreported | Unreported | Acute  Unreported  Warm-up |
| Akehurst et al. [22] **🟢**  (English, United Kingdom, Europe) | 22  ♂: 14 (63.6%); ♀: 8 (36.4%)  ♂: 26±?; ♀: 27±? | Field Hockey | Tiers 3 and 4 | Open Access funding enable and organized by Projekt DEAL | One author was Editor-in-Chief of the journal where the study was published | Acute  Off-season  Post-exercise |
| Alipasali et al. [23]  Alipasali et al. [24] **🟢**  (English, Greece, Europe) | 27 (of 50) for sprint; 42 (of 50) for CMJ  ♂ (100%)  21.6±2.1 (sprint); 21.5±1.8 (CMJ) | Volleyball | Tier 2 | No funding | The authors declared no competing interests | Chronic (6 weeks, 3x/week, 18 sessions)  Competitive season  Warm-up |
| Almeida Júnior et al. [25] **🟢**  (English, Brazil, South America) | 18  ♂ (100%)  25.0±3.1 | Brazilian Jiu-Jitsu | Tier 2 | FAPITEC/SE | The authors declared no competing interests | Acute  Unreported  Warm-up |
| Almeida Júnior et al. [26] **🟢**  (English, Brazil, South America) | 12  ♂ (100%)  22.8±5.5 | Judo | Tier 2 | FAPITEC/SE | Unreported | Chronic (10 weeks, 3x/week, 30 sessions)  Unreported  Warm-up |
| Alp [27] **🟢**  (English, Turkey, Europe and Asia) | 14  ♂ (100%)  20.3±1.1 | Handball | Tier 2 | Unreported | Unreported | Acute  Unreported  Warm-up |
| Alp et al. [28] **🟢**  (English, Turkey, Europe and Asia) | 14  ♂ (100%)  21.3±0.9 | Taekwondo | Tier 2 | Unreported | The authors declared no competing interests | Acute  Unreported  Warm-up |
| Amir Vazini, Parnow [29] **🟢**  (English, Iran, Asia) | 22  Unreported  23±4 | Soccer | Tier 2 | Unreported | The authors declared no competing interests | Acute  Unreported  Warm-up |
| Amiri-Khorasani [30] **🟡**  (English, Iran, Asia) | 15  ♂ (100%)  21.2±2.0 | Soccer | Tier 2 | Unreported | Unreported | Acute  Unreported  Warm-up |
| Amiri-Khorasani [31] **🟢**  (English, Iran, Asia) | 24  ♀ (100%)  22.1±0.8 | Soccer | Tier 2 | Unreported | The authors declared no competing interests | Acute  Competitive season  Warm-up |
| Amiri-Khorasani, Ferdinands [32] **🟢**  (English, Iran, Asia) | 24  ♂ (100%)  19.4±1.8 | Soccer | Tier 2 | Unreported | Unreported | Acute  Unreported  Warm-up |
| Amiri-Khorasani, Kellis [33] **🟢**  (English, Iran, Asia) | 12  ♂ (100%)  18.8±0.8 | Soccer | Tier 2 | Unreported | Unreported | Acute  Competitive season  Warm-up |
| Amiri-Khorasani, Sotoodeh [34] **🟢**  (English, Iran, Asia) | 16  ♂ (100%)  21.5±2.0 | Soccer | Tier 2 | Unreported | Unreported | Acute  Unreported  Warm-up |
| Amiri-Khorasani et al. [35] **🟡**  (English, Malaysia, Asia) | 6  ♂ (100%)  19.2±1.8 | Soccer | Tier 2 | University Malaya Research Grant | Unreported | Acute  Unreported  Warm-up |
| Amiri-Khorasani et al. [36] **🟢**  (English, Iran, Asia) | 20  Unreported  16.9±0.9 | Soccer | Tier 3 | Unreported | Unreported | Acute  Competitive season  Warm-up |
| Amiri-Khorasani et al. [37]  Amiri-Khorasani et al. [38] **🟡**^a^  (English, Iran, Asia) | 18  ♂ (100%)  19.2±1.8 | Soccer | Tier 3 | University Malaya Research Grant | Unreported | Acute  Competitive season  Warm-up |
| Amiri-Khorasani et al. [39] **🟢**  (English, Iran, Asia) | 19  ♂ (100%)  22.5±2.5 | Soccer | Tier 3 | Unreported | Unreported | Acute  Unreported  Warm-up |
| Andre et al. [40] **🟢**  (English, USA, North America) | 40  ♂ (100%)  16.0±1.0 | American Football Track and Field | Tier 2 | Unreported | Unreported | Acute  Unreported  Warm-up |
| Andrejić et al. [41] **🟢**  (English, Serbia, Europe) | 23  ♂ (100%)  13.6±0.5 | Basketball | Tier 2 | Unreported | Unreported | Acute  Unreported  Warm-up |
| Annino et al. [42] **🟢**  (English, Italy, Europe) | 10  ♂ (100%)  29.0±6.7 | Basketball | Tier 3 | No funding | The authors declared no competing interests | Acute  Competitive season  Warm-up |
| Ari [43] **🟢**  (English, Turkey, Europe and Asia) | 8  ♀ (100%)  15.4±1.1 | Wrestling | Tier 2 | No funding | The authors declared no competing interests | Acute  Pre-season  Warm-up |
| Arihiro et al. [44] **🟢**  (English, Japan, Asia) | 30  ♂ (100%)  Controls: 20.7±1.3; Pilates: 19.5±1.5; Stretching: 19.3 ±1.7 | Rugby | Tier 3 | No funding | The authors declared no competing interests | Chronic (3 weeks, unreported, 10 sessions)  Unreported  Warm-up |
| Avaz et al. [45] **🟢**  (Persian, Iran, Asia) | 31  ♂ (100%)  21.2±1.7 | Volleyball | Tier 2 | Unreported | Unreported | Acute  Unreported  Warm-up |
| Avedesian et al. [46] **🟢**  (English, USA, North America) | 12  ♀ (100%)  19.8±1.2 | Volleyball | Tier 3 | Unreported | The authors declared no competing interests | Acute  Unreported  Warm-up |
| Avloniti et al. [47] **🟢**  (English, Greece, Europe) | 34  ♂ (100%)  20.5±1.4 | Unreported | Tier 2 | Bodosakis Foundation (Greece)  Grant funding CE-80739 | The authors declared no competing interests | Acute  Unreported  Warm-up |
| Avloniti et al. [48] **🟢**  (English, Greece, Europe) | 40  ♂ (100%)  Single set: 21.2±1.6; Multiple sets: 20.8±0.8 | Unreported | Tier 2 | Bodosakis Foundation (Greece)  Grant funding CE-80739 | The authors declared no competing interests | Acute  Unreported  Warm-up |
| Ayala, De Baranda [49]  De Baranda, Ayala [50] **🟢**  (Spanish, Spain, Europe) | 28  ♂ (100%)  17.6±0.8 | Soccer | Tier 2 | Unreported | Unreported | Acute  Unreported  Warm-up |
| Ayala et al. [51] **🟢**  (English, Spain, Europe) | 18  ♀ (100%)  21.3±2.5 | Futsal | Tiers 3 and 4 | Program of Human Resources Formation by Science and Technology, Agency for Science and Technology of Murcia | Unreported | Chronic (8 weeks, 3x/week, 24 sessions)  Competitive season  Unreported |
| Aydoǧ et al. [52] **🟡**  (Turkish, Turkey, Europe and Asia) | 10  ♂ (100%)  14 (range 12 to 16) | Artistic Gymnastics Weightlifting | Tier 3 | Unreported | Unreported | Acute  Unreported  Warm-up |
| Azuma, Someya [53] **🟢**  (English, Japan, Asia) | 124  ♂ (100%)  16.2±0.8 | Soccer | Tier 3 | Unreported | Unreported | Chronic (12 weeks, 3x/week, 36 sessions)  Competitive season  Post-exercise + independent sessions |
| Babbar et al. [54] **🟢**  (English, India, Asia) | 42  ♂ (100%)  17.0±0.5 | Soccer | Tier 2 | Unreported | Unreported | Acute  Unreported  Warm-up |
| Balci et al. [11] **🟢**  (English, Turkey, Europe and Asia) | 74  ♂: 59 (79.7%); ♀: 15 (20.3%)  Neural sliding: 17.9±2.3; Neural stretching: 18.1±2.3 | Wrestling | Tier 3 | Unreported | The authors declared no competing interests | Acute  Unreported  Warm-up |
| Bali, Guru [55] **🟢**  (English, India, Asia) | 42  ♂ (100%)  Mulligan’s Adductor: 23.6±5.7; Static Adductor: 23.6±5.8 | Soccer | Tiers 2 and 3 | Unreported | Unreported | Chronic (1 week, 3x/week, 3 sessions)  Unreported  Unreported |
| Barbosa et al. [56] **🟢**  (English, Brazil, South America) | 11  ♂ (100%)  21.5±2.4 | Volleyball | Tier 2 | CAPES 2014–2016 | The authors declared no competing interests | Acute  Unreported  Warm-up |
| Baumgart et al. [57] **🟢**  (English, Germany, Europe) | 62  ♂: 33 (53.2%); ♀: 29 (46.7%)  Controls: 27.9±7.4; Soccer: 25.1±5.6; Stretching: 26.9±6.2 | Soccer | Tier 2 | Unreported | The authors declared no competing interests | Acute  Unreported  Unreported |
| Bazett-Jones et al. [58] **🟢**  (English, USA, North America) | 21  ♀ (100%)  18.6±0.7 | Track and Field | Tier 3 | Unreported | Unreported | Chronic (6 weeks, 4x/week, 24 sessions)  Pre-season  Warm-up |
| Bazett-Jones et al. [59] **🟢**  (English, USA, North America) | 10  ♂ (100%)  20.6±1.5 | American Football Track and Field | Tier 3 | Unreported | Unreported | Acute  Unreported  Warm-up |
| Beckett et al. [60] **🟢**  (English, Australia, Oceania) | 12  ♂ (100%)  23±4 | Australian Rules Football Field Hockey Rugby Soccer | Tier 2 | Unreported | Unreported | Acute  Competitive season  Inter-set |
| Behara, Jacobson [61] **🟢**  (English, USA, North America) | 14  Unreported  20.0±1.4 | American Football | Tier 3 | Foam rollers donated by Rumble Roller® | Unreported | Acute  Off-season  Warm-up |
| Belkhiria-Turki et al. [62] **🟢**  (English, Tunisia, Africa) | 37  ♂ (100%)  Controls: 20.8±1.8; Stretching while moving: 20.6±1.0; Stretching in place: 20.9±1 | Soccer | Tier 2 | Unreported | Unreported | Chronic (8 weeks, 3x/week, 24 sessions)  Competitive season  Warm-up |
| Belkhiria-Turki et al. [63] **🟢**  (English, Tunisia, Africa) | 28  ♂: 15 (53.6%); ♀: 13 (46.4%)  ♂: 22.7±1.9; ♀: 22.1±0.3 | Artistic Gymnastics Basketball Handball Soccer Swimming Track and Field | Tier 2 | Unreported | Unreported | Acute  Competitive season  Warm-up |
| Bello et al. [64] **🟢**  (English, Brazil, South America) | 14  ♂ (100%)  PNF: 24±3; Static stretching: 23±5 | Indoor Soccer | Tier 3 | Unreported | Unreported | Chronic (16 weeks, 3x/week, 48 sessions)  Unreported  Post-exercise |
| Ben Maaouia et al. [65] **🟢**  (English, Tunisia, Africa) | 20  ♂ (100%)  17.4±0.9 | Soccer | Tier 3 | Higher Institute of Sport and Physical Education of Ksar Said, Tunis, Tunisia | The authors declared no competing interests | Acute  Unreported  Warm-up |
| Bingul et al. [66] **🟡**  (English, Turkey, Europe and Asia) | 23  Unreported  15.0±0.8 | Soccer | Tier 2 | Unreported | Unreported | Acute  Unreported  Warm-up |
| Bishop, Middleton [67] **🟢**  (English, United Kingdom, Europe) | 25  ♂ (100%)  20.3±1.3 | Team Sports (unclear which) | Tier 2 | Unreported | Unreported | Acute  Unreported  Warm-up |
| Bogdanis et al. [68] **🟢**  (English, Greece, Europe) | 16  ♂ (100%)  24±4 | Artistic Gymnastics | Tier 4 | Unreported | Unreported | Acute  Unreported  Warm-up |
| Boudenot et al. [69] **🟢**  (French, France, Europe) | 7  ♂ (100%)  19.7±1.1 | Kayak Swimming Undisclosed team sports | Tier 2 | Unreported | The authors declared no competing interests | Acute  Unreported  Warm-up |
| Bouthin, Edouard [70] **🟢**  (French, France, Europe) | 42  Unreported  24.6 (range: 20-32) | Soccer | Tier 2 | Unreported | The authors declared no competing interests | Chronic (8 weeks, unreported, unreported)  Competitive season  Post-exercise |
| Brodowicz et al. [71] **🟢**  (English, USA, North America) | 24  ♂ (100%)  20.7±1.2 | Baseball | Tier 2 | Unreported | Unreported | Acute  Unreported  Unreported |
| Burkett et al. [72] **🟢**  (English, USA, North America) | 29  ♂ (100%)  20.0±1.7 | American Football | Tier 3 | Unreported | Unreported | Acute  Off-season  Warm-up |
| Burkett et al. [73] **🟢**  (English, USA, North America) | 15  ♀ (100%)  20.5±1.5 | Unreported (NCAA Division I, but unclear sports) | Tier 3 | Unreported | Unreported | Acute  Unreported  Warm-up |
| Buttifant, Hrysomallis [74] **🟢**  (English, Australia, Oceania) | 12  ♂ (100%)  21±2 | Australian Rules Football | Tier 3 | No funding | The authors declared no competing interests | Acute  Unreported  Warm-up |
| Caliskan et al. [75] **🟢**  (English, Turkey, Europe and Asia) | 20  ♂ (100%)  Median 14.5, IQR 12-5-16.5 | Basketball | Tier 2 | Unreported | The authors declared no competing interests | Acute  Unreported  Warm-up |
| Caplan et al. [76] **🟢**  (English, United Kingdom, Europe) | 18  Unreported  20.2±1.1 | Rugby League | Tier 2 | No funding | The authors declared no competing interests | Chronic (5 weeks, 4x/week, 20 sessions)  Competitive season  Post-exercise |
| Carvalho et al. [77] **🟢**  (English, Brazil, South America) | 16  ♂ (100%)  14.5±2.8 | Tennis | Tier 2 | Unreported | Unreported | Acute  Unreported  Warm-up |
| Carvalho et al. [78] **🟡**  (Spanish, Brazil, South America) | 9  ♂: 5 (55.6%); ♀: 4 (44.4%)  14±1 | Tennis | Tier 2 | Unreported | Unreported | Acute  Unreported  Post-exercise |
| Celik [79] **🟢**  (English, Turkey, Europe and Asia) | 36  ♀ (100%)  Range: 13-15 | Volleyball | Tier 2 | No funding | The authors declared no competing interests | Acute  Unreported  Warm-up |
| César et al. [80] **🟢**  (English, Brazil, South America) | 21  ♂ (100%)  27.0±5.9 | Brazilian Jiu-Jitsu | Tier 2 | Unreported | Unreported | Acute  Unreported  Post-exercise |
| Cetin et al. [81] **🟡**  (English, Turkey, Europe and Asia) | 20  ♂ (100%)  20.0±1.1 | Track and Field | Tier 2 | No funding | The authors declared no competing interests | Acute  Unreported  Warm-up |
| Chaouachi et al. [82] **🟡**  (English, Tunisia, Africa) | 19  ♂ (100%)  20.6±1.2 | Multiple sports, both team and individual (unclear which) | Tiers 3 and 4 | Tunisian Ministry of Scientific Research, Technology and Development of Competences | Unreported | Acute  Off-season  Warm-up |
| Chaouachi et al. [83] **🟡**  (English, Tunisia, Africa) | 14  ♂ (100%)  18±2 | Rowing | Tier 4 | No funding | The authors declared no competing interests | Acute  Competitive season  Warm-up |
| Chatzopoulos et al. [84] **🟡**  (English, Greece, Europe) | 25  ♂ (100%)  11.8±1.7 | Taekwondo | Tier 2 | No funding | The authors declared no competing interests | Acute  Unreported  Warm-up |
| Chatzopoulos et al. [85] **🟡**  (English, Greece, Europe) | 31  ♀ (100%)  17.3±0.5 | Basketball Handball Track and Field (sprint and long jump) Volleyball | Tier 2 | Unreported | Unreported | Acute  Competitive season  Warm-up |
| Chatzopoulos et al. [86] **🟢**  (English, Greece, Europe) | 24  ♀ (100%)  15.1±0.9 | Soccer | Tier 2 | Unreported | Unreported | Acute  Unreported  Warm-up |
| Chen et al. [87] **🟢**  (English, Taiwan, Asia) | 12  ♂: 10 (83.3%); ♀: 2 (16.7%)  21.6±4.1 | Tennis | Tier 3 | No funding | The authors declared no competing interests | Acute  Off-season  Warm-up |
| Chen et al. [88] **🟢**  (English, Taiwan, Asia) | 10  ♂ (100%)  23.5±1.6 | Volleyball | Tier 2 | Ministry of Science and Technology (MOST), Taiwan, grant 107-2221-E-131-021-MY3 | The authors declared no competing interests | Unreported (unclear if acute or chronic)  Unreported  Post-exercise |
| Chinnavan et al. [89] **🟢**  (English, India, Asia) | 30  ♂ and ♀ (unclear percentage)  Range: 17-20 | Soccer | Tier 2 | Unreported | The authors declared no competing interests | Chronic (4 weeks, 5x/week, 20 sessions)  Unreported  Warm-up + Post-exercise |
| Christensen et al. [91] **🟢**  (English, USA, North America) | 12  ♀ (100%)  19.3±1.2 [of the original 24 players, but there were 12 dropouts] | Soccer | Tier 3 | Unreported | Unreported | Acute  Off-season  Warm-up |
| Christensen, Nordstrom [90] **🟢**  (English, USA, North America) | 68  ♂: 36 (52.9%); ♀: 32 (47.1%)  20.5±1.4 | American Football Basketball Soccer Softball Track and Field Volleyball Wrestling | Tier 3 | Unreported | Unreported | Acute  Off-season (63) and competitive season (5)  Warm-up |
| Chtourou et al. [92] **🟢**  (English, Tunisia, Africa) | 20  ♂ (100%)  18.6±1.3 | Soccer | Tier 3 | No funding | The authors declared no competing interests | Acute  Unreported  Warm-up |
| Church et al. [93] **🟢**  (English, USA, North America) | 40  ♀ (100%)  20.3±1.6 | Rowing Tennis Track and Field (jumpers, throwers, and sprinters) Volleyball | Tier 3 | Unreported | Unreported | Acute  Unreported  Warm-up |
| Colak [94] **🟡**  (English, Turkey, Europe and Asia) | 15  ♀ (100%)  22.1±2.7 | Soccer | Tier 4 | Unreported | Unreported | Acute  Pre-season  Warm-up |
| Coons et al. [95] **🟢**  (English, USA, North America) | 25  ♀ (100%)  15.1±1.2 | Volleyball | Tier 2 | Unreported | Unreported | Chronic (4 weeks, 3x/week, 12 sessions)  Pre-season  Warm-up |
| Correia et al. [96] **🟡**  (Portuguese, Brazil, South America) | 18  ♂ (100%)  20.6±2.1 | Soccer | Tier 2 | Unreported | Unreported | Acute  Unreported  Unreported |
| Cwirlej-Sozanska et al. [97] **🔴**  (English, Poland, Europe) | 25  ♂ (100%)  16.6±0.5 | Track and Field | Tier 3 | No funding | The authors declared no competing interests | Acute  Pre-season  Warm-up |
| da Silva et al. [98] **🔴**  (Portuguese, Brazil, South America) | 28  ♂: 16 (57.1%); ♀: 12 (42.9%)  ♂: 16.0±0.8; ♀: 14.1±1.0 | Track and Field | Tier 2 | Unreported | Unreported | Acute  Unreported  Warm-up |
| Dalamitros et al. [99] **🟡**  (English, Greece, Europe) | 19  ♂: 10 (52,6%); ♀: 9 (47,4%)  19.3±2.2 | Swimming | Tier 3 | No funding | The authors declared no competing interests | Acute  Off-season  Warm-up |
| Dallas et al. [100] **🟢**  (English, Greece, Europe) | 18  ♂ and ♀ (unclear percentage)  21.8±1.8 | Artistic gymnastics | Tier 2 | Unreported | The authors declared no competing interests | Acute  Unreported  Warm-up |
| Dalrymple et al. [101] **🟢**  (English, USA, North America) | 12  ♀ (100%)  19.5±6.1 | Track and Field | Tier 3 | Unreported | Unreported | Acute  Off-season  Warm-up |
| Damasceno et al. [102] **🟢**  (English, Brazil, South America) | 11  ♂ (100%)  35.7±6.1 | Track and Field (long-distance running) | Tier 2 | São Paulo Research Foundation (FAPESP: 2011/10742-9, 2011/02769-4, and 2010/13913-6) | The authors declared no competing interests | Acute  Pre-season  Warm-up |
| Darcadia et al. [103] **🟡**  (Portuguese, Brazil, South America) | 28  ♂ (100%)  15.0±0.5 | Soccer | Tier 2 | Unreported | Unreported | Acute  Unreported  Warm-up |
| Dawson et al. [104] **🟡**  (English, Australia, Oceania) | 17  Unreported  24.2±2.9 | Australian Rules Football | Tier 3 | Australian Football League | Unreported | Acute  Competitive season  Post-exercise |
| de Almeida Leme et al. [105] **🟢**  (Portuguese, Brazil, South America) | 17  ♂ (100%)  Range: 15-17 | Soccer | Tier 2 | Unreported | Unreported | Acute  Competitive season  Warm-up |
| de Castro et al. [106] **🟢**  (English, Brazil, South America) | 162  ♂ (100%)  Range: 10-18 | Soccer | Tier 2 | Unreported | Unreported | Chronic (12 weeks, 3x/week, 36 sessions)  Competitive season  Unreported |
| De Oliveira Júnior et al. [107] **🟡**  (English, Brazil, South America) | 8  ♂ (100%)  21±5 | Basketball | Tier 2 | Unreported | The authors declared no competing interests | Acute  Unreported  Warm-up |
| de Oliveira, Pinto Lopes Rama [108] **🟢**  (Portuguese, Portugal, Europe) | 22  ♂ (100%)  24.0±6.2 | Handball Rugby7 | Tier 3 | Unreported | Unreported | Acute  Unreported  Warm-up |
| de Rezende et al. [109] **🟢**  (English, Brazil, South America) | 8  ♀ (100%)  15.4±0.5 | Volleyball | Tier 2 | Unreported | Unreported | Acute  Unreported  Warm-up |
| Di Cagno et al. [110] **🟡**^b^  (English, Italy, Europe) | 38  Unreported  14.1±3.2 | Rhythmic Gymnastics | Tiers 3 and 4 | Unreported | Unreported | Acute  Competitive season  Warm-up |
| Di Cagno et al. [111] **🟡**  (English, Italy, Europe) | 18  ♂ (100%)  21.5±3.1 | Volleyball | Tier 3 | No funding | The authors declared no competing interests | Acute  Competitive season  Warm-up |
| Donti et al. [112] **🟢**  (English, Greece, Europe) | 27  ♀ (100%)  19.9±3.0 | Artistic Gymnastics Basketball Handball Rhythmic Gymnastics Volleyball | Tier 2 | No funding | The authors declared no competing interests | Acute  Unreported  Warm-up |
| Donti et al. [113] **🔴**  (English, Greece, Europe) | 20  ♀ (100%)  Rhythmic gymnastics: 21.3±1.6; Volleyball: 24.3±4.7 | Rhythmic Gymnastics Volleyball | Tier 3 | No funding | The authors declared no competing interests | Acute  Competitive season  Warm-up |
| Donti et al. [114] **🟢**  (English, Greece, Europe) | 77  ♀ (100%)  Range: 8-10 | Artistic Gymnastics | Tier 2 | No funding | The authors declared no competing interests | Chronic (15 weeks, 3x/week, 45 sessions)  Competitive season  Warm-up |
| Drews, Goltz [115] **🟢**  (German, Germany, Europe) | 48  Unreported  Controls: 24.9±6.7; Longitudinal stretching: 25.2±5.2; Transversal stretching: 26.3±5.9 | Soccer | Tier 2 | Unreported | Unreported | Chronic (4 weeks, 2x/week, 8 sessions)  Unreported  Warm-up OR post-exercise |
| Ebadi, Cetin [116] **🟡**  (English, Turkey, Europe and Asia) | 15  ♂ (100%)  Unreported | Basketball Soccer | Tier 3 | Unreported | The authors declared no competing interests | Acute  Competitive season  Warm-up |
| Egan et al. [7] **🔴**  (English, USA, North America) | 11  ♀ (100%)  20.0±1.1 | Basketball | Tier 3 | Unreported | Unreported | Acute  Off-season  Warm-up |
| Eken, Bayer [117] **🟡**  (English, Turkey, Europe and Asia) | 12  ♂ (100%)  18.5±1.0 | Kickboxing | Tier 4 | No funding | The authors declared no competing interests | Acute  Unreported  Warm-up |
| Espi-Lopez et al. [118] **🟢**  (English, Spain, Europe) | 42  Unreported  Manual therapy: 21.0±2.5; PNF: 22.4±5.3 | Field hockey | Tier 2 | No funding | The authors declared no competing interests | Chronic (3 weeks, 1x/week, 3 sessions)  Competitive season  Independent sessions |
| Evetovich et al. [119] **🔴**  (English, USA, North America) | 15 ^c^  ♀ (100%)  19.9±0.8 | Basketball Volleyball | Tier 3 | Unreported | Unreported | Acute  Off-season  Warm-up |
| Faigenbaum et al. [120] **🟢**  (English, USA, North America) | 30  ♂: 26 (86.7%); ♀: 4 (13.3%)  15.5±0.9 | American Football Weightlifting | Tier 2 | Unreported | Unreported | Acute  Unreported  Warm-up |
| Faigenbaum et al. [121] **🟢**  (English, USA, North America) | 19  ♂ (100%)  16.5±1.1 | American Football Lacrosse | Tier 2 | Unreported | Unreported | Acute  Unreported  Warm-up |
| Faigenbaum et al. [122] **🟢**  (English, USA, North America) | 18  ♀ (100%)  15.3±1.2 | Basketball Lacrosse Soccer Track and Field Volleyball | Tier 2 | Unreported | Unreported | Acute  Unreported  Warm-up |
| Famisis [123] **🟢**  (English, Greece, Europe) | 18  Unreported  26.0±2.4 | Soccer | Tier 2 | Unreported | Unreported | Acute  Competitive season  Warm-up |
| Farshidi et al. [124] **🟢**  (English & Italian, Austria, Europe) | 45  ♂ (100%)  Controls: 17.1±0.1; PNF: 17.0±0.7; Static stretching: 17.1±0.5 | Soccer | Tier 3 | Grant (Project J4484) from Australian Science fund | Unreported | Acute  Unreported  Post-exercise |
| Fattahi-Bafghi, Amiri-Khorasani [125] **🟢**  (English, Iran, Asia) | 15  Unreported  24.7±4.6 | Soccer | Tier 2 | The Research Deputy of Bafgh Branch, Islamic Azad University | Unreported | Chronic (3 weeks, 3x/week, 9 sessions)  Unreported  Warm-up |
| Favero et al. [126] **🟢**  (English, Australia, Oceania) | 10  ♂ (100%)  22.0±2.3 | Touch Football | Tier 2 | Unreported | Unreported | Acute  Competitive season  Warm-up |
| Feitosa Junior et al. [127] **🟢**  (Portuguese, Brazil, South America) | 23  ♂ (100%)  Controls: 27.8±6.2; Stretching: 29.2±6.9 | Brazilian Jiu-Jitsu | Tier 2 ^d^ | Unreported | The authors declared no competing interests | Acute  Unreported  Warm-up |
| Fernandes et al. [128] **🟢**  (English, Canada, North America) | 11  ♂ (100%)  27±4 | Brazilian Jiu-Jitsu | Tier 4 | CAPES [BEX4348/09-5], Ministry of Culture, Brazil Grants from the National Research and Technological Development Council (CNPq) and the State of Rio de Janeiro Research (FAPERJ) | Unreported | Acute  Unreported  Warm-up |
| Ferreira et al. [129] **🟢**  (Portuguese, Brazil, South America) | 22  ♂ (100%)  26.3±3.9 | Soccer | Tier 3 | Unreported | Unreported | Acute  Competitive season  Warm-up |
| Ferri-Caruana et al. [130] **🟢**  (English, Spain, Europe) | 18  ♀ (100%)  13±2 | Artistic Gymnastics | Tiers 2 and 3 | Unreported | Unreported | Chronic (7 weeks, 4x/week, 28 sessions)  Unreported  Warm-up |
| Fletcher, Anness [131] **🟢**  (English, United Kingdom, Europe) | 18  ♂: 10 (55.6%); ♀: 8 (44.4%)  ♂: 19.2±1.1; ♀: 20.2±2.9 | Track and Field (sprinters) | Tier 2 | Unreported | Unreported | Acute  Competitive season  Warm-up |
| Fletcher, Jones [132] **🟢**  (English, United Kingdom, Europe) | 97  ♂ (100%)  23.0±8.4 | Rugby Union | Tier 2 | Unreported | Unreported | Acute  Unreported  Warm-up |
| Fletcher, Monte-Colombo [133]  Fletcher, Monte-Colombo [134] **🟢**  (English, United Kingdom, Europe) | 27 in study (a); 21 in study (b) (subset)  ♂ (100%)  20.5±2.2 (a); 20.8±2.3 (b) | Soccer | Tier 2 | University of Bedfordshire | Unreported | Acute  Unreported  Warm-up |
| Forte et al. [135] **🟢**  (English, unclear country [Italy or Croatia], Europe) | 30  Unreported  Controls: 20.4±3.1; Dynamic stretching: 21.3±3.2; Static stretching: 20.7±2.9 | Volleyball | Tier 2 | Unreported | Unreported | Chronic (6 weeks, 3x/week, 18 sessions)  Competitive season  Unreported |
| Frantz, Ruiz [136] **🟡**  (English, USA, North America) | 17  Unreported  19.6±1.4 | Baseball | Tier 2 | No funding | Unreported | Acute  Competitive season  Warm-up |
| Fredericson et al. [137] **🟢**  (English, USA, North America) | 5  ♂ (100%)  Unreported | Track and Field (distance running) | Tier 3 | No funding | Unreported | Acute  Unreported  Warm-up |
| Frikha et al. [138] **🟢**  (English, Saudi Arabia, Asia) | 18  ♂ (100%)  13.0±0.7 | Soccer | Tier 2 | Unreported | Unreported | Acute  Unreported  Warm-up |
| Funk et al. [139] **🟢**  (English, USA, North America) | 40  ♂: 20 (50.0%); ♀: 20 (50.0%)  19.1±1.4 | Baseball Field hockey Rowing | Tier 3 | Unreported | Unreported | Acute  Unreported  Warm-up + Post-exercise |
| Funk et al. [140] **🟢**  (English, USA, North America) | 30  Unreported  Range: 18-22 | American Football | Tier 2 | Unreported | Unreported | Acute  Unreported  Warm-up |
| Gabbe et al. [141] **🟢**  (English, Australia, Oceania) | 220  ♂ (100%)  Eccentric training: 23.4 (range: 18.0-35.0); Stretching: 23.9 (range: 17.4-36.0) | Australian Rules Football | Tier 2 | Grant from Sport and Recreation Victoria Public Health Research Fellowship (237024) from the National Health and Medical Research Council of Australia | Unreported | Chronic (12 weeks, unreported, 5 sessions)  Pre-season + competitive season  Post-exercise |
| Galazoulas [142] **🟢**  (English, Greece, Europe) | 16  ♂ (100%)  21.8±1.2 | Basketball | Tier 2 | Unreported | Unreported | Acute  Competitive season  Warm-up |
| Galazoulas [143] **🟢**  (English, Greece, Europe) | 20  ♂ (100%)  23.4±0.5 | Basketball | Tier 2 | Unreported | Unreported | Acute  Off-season  Warm-up |
| Galetin et al. [144] **🟡**  (English, Serbia, Europe) | 61  ♀ (100%)  13.7± 0.7 | Volleyball | Tier 2 | Unreported | Unreported | Acute  Unreported  Warm-up |
| Gao et al. [145] **🟡**  (English, China, Asia) | 10  ♀ (100%)  22.0±1.0 | Aerobics Gymnastics | Tier 3 | Unreported | Unreported | Acute  Unreported  Warm-up |
| Gelen [146] **🟢**  (English, Turkey, Europe and Asia) | 26  ♂ (100%)  23.3±3.2 | Soccer | Tier 3 | Unreported | Unreported | Acute  Unclear if pre-season or competitive season (4th week of a 16-week season)  Warm-up |
| Gelen et al. [147] **🟢**  (English, Turkey, Europe and Asia) | 53  ♂ (100%)  21.9±2.6 | Basketball Soccer Volleyball | Tier 2 | No funding | The authors declared no competing interests | Acute  Competitive season  Warm-up |
| Gelen et al. [148] **🟢**  (English, Turkey, Europe and Asia) | 26  Unreported  15.1±4.2 | Tennis | Tier 4 | Unreported | Unreported | Acute  Competitive season  Warm-up |
| Gergley [149]  Gergley [150] **🟡**  (English, USA, North America) | 15 and 9 (subset of the 15)  ♂ (100%)  20.6±1.9; 20.4±1.8 | Golf | Tier 2 | Office of Research and Sponsored Programs through the Research Enhancement Program. James I. Perkings Faculty Research Academy | Unreported | Acute  Unreported  Warm-up |
| Ghasemi et al. [151] **🟡**  (English, Iran, Asia) | 9  ♀ (100%)  24.1±3.6 | Basketball | Tier 3 ^e^ | Grant from the Postgraduate Studies and Research Program, Tehran University of Medical Sciences | The authors declared no competing interests | Acute  Unreported  Warm-up |
| Gonçalves et al. [152] **🟢**  (Portuguese, Brazil, South America) | 18  ♂ (100%)  Dynamic stretching: 15.7±0.7; Static stretching: 15.9±0.9 | Soccer | Tier 2 | Unreported | The authors declared no competing interests | Acute and chronic (16 weeks, <1 sessions/week, 12 sessions)  Competitive season  Unreported |
| Gürses, Akgül [153] **🟢**  (Turkish, Turkey, Europe and Asia) | 26  ♂ (100%)  21.9±2.2 | Soccer | Tier 2 | Unreported | Unreported | Acute  Unreported  Warm-up |
| Haag et al. [154] **🟡**  (English, USA, North America) | 12  Unreported  20.3±1.1 | Baseball | Tier 3 | Unreported | Unreported | Acute  Pre-season  Warm-up |
| Haddad et al. [155] **🟡**  (English, Tunisia, Africa) | 16  ♂ (100%)  18.2±1.2 | Soccer | Tier 3 | Unreported | Unreported | Acute  Pre-season  On previous day |
| Haddad et al. [156] **🟢**  (English, Qatar, Asia) | 8  ♂ (100%)  17.3±1.1 | Track and Field | Tier 3 | Qatar University – University Grant QUUG-CAS-DSSP-15 \16-15 and Qatar National Library | The authors declared no competing interests | Acute  Competitive season  Warm-up |
| Han et al. [157] **🟢**  (English, South Korea, Asia) | 28  Unreported  High-school students of unreported age | Track and Field | Tier 2 | Unreported | Unreported | Chronic (12 weeks, 6x/week, 72 sessions)  Unreported  Warm-up |
| Heisey, Kingsley [158] **🟢**  (English, USA, North America) | 18  ♀ (100%)  Controls: 20±1; Static stretching: 20±1 | Track and Field | Tier 3 | Unreported | Unreported | Acute  Competitive season  Warm-up |
| Herman, Smith [159] **🟢**  (English, USA, North America) | 20  ♂ (100%)  20.3±0.0 | Wrestling | Tier 3 | Unreported | Unreported | Chronic (4 weeks, 5x/week, 20 sessions)  Pre-season  Warm-up |
| Higuchi et al. [160] **🔴**  (English, Japan, Asia) | 34  ♂ (100%)  Unreported | Baseball | Tier 2 | No funding | The authors declared no competing interests | Acute  Unreported  Warm-up |
| Holt, Lambourne [161] **🟢**  *Erratum*: Holt, Lambourne [326]  (English, USA, North America) | 63  ♂ (100%)  20.7±1.8 | Soccer | Tier 3 | Unreported | Unreported | Acute  Unreported  Warm-up |
| Hough et al. [162] **🟢**  (English, United Kingdom, Europe) | 11  ♂ (100%)  21.0±2.0 | Cricket Field hockey Soccer Squash Track and field | Tier 2 | Unreported | Unreported | Acute  Unreported  Warm-up |
| Hsu et al. [163] **🟢**  (English, Taiwan, Asia) | 23  ♂: 14 (60.9%); ♀: 9 (39.1%)  ♂: 20.7±0.9; ♀: 20.5±0.7 | Table Tennis | Tier 3 | NSYSU-KMU Joint Research Project (NSYSUKMU107-P021) | Unreported | Acute  Unreported  Warm-up |
| Huang et al. [164] **🟢**  (English, China, Asia) | 14  ♂ (100%)  22.6±1.7 | Soccer | Tier 3 | No funding | The authors declared no competing interests | Acute  Unreported  Warm-up |
| Huang, Zhu [165] **🟡**  (Chinese, China, Asia) | 19  ♂ (100%)  24.3±2.9 | Taekwondo | Tier 4 | Unreported | Unreported | Acute  Competitive season  Warm-up |
| Ide et al. [166] **🟢**  (English, Brazil, South America) | 15  ♂ (100%)  21±3 | Soccer | Tier 2 | FAPESP (Fundação de Amparo à Pesquisa no Estado de São Paulo), Brazil, Grant 2012/20309-3 | Unreported | Acute  Competitive season  Warm-up |
| Ishak et al. [167]  Ishak et al. [168] **🟡**  (English, Malaysia, Asia) | 13 and 15  ♂ (100%)  22.1±0.6 | Handball Futsal | Tier 2 | University Research Grant No.2016-0202-102-01 | The authors declared no competing interests | Acute  Unreported  Warm-up |
| Ishak et al. [169] **🟡**  (English, Malaysia, Asia) | 14  ♂ (100%)  23.4±1.3 | Soccer | Tier 2 | Research and Innovation Centre, Universiti Pendidikan Sultan Idris, Tanjong | Unreported | Acute  Pre-season  Warm-up |
| Jang et al. [170] **🟢**  (English, South Korea, Asia) | 16  ♂ (100%)  21.8±1.5 | Badminton | Tier 2 | National Research Foundation of Korea Grant (NRF-2014S1A5A8019804) | The authors declared no competing interests | Acute  Unreported  Warm-up |
| Jemni et al. [171] **🟢**  (English, United Kingdom, Europe) | 21  ♂ (100%)  21.9±1.8 | Soccer | Tier 2 | Unreported | Unreported | Acute  Unreported  Warm-up |
| Jing, Yang [172] **🟢**  (Chinese, China, Asia) | 22  ♂ (100%)  Dynamic stretching: 21.3±2.0; Static stretching: 20.9±4.1 | Volleyball | Tier 3 | Hebei Provincial Philosophy and Social Science Fund Project (HB16TY022) | Unreported | Chronic (8 weeks, 4x/week, 32 sessions)  Unreported  Unreported |
| Johnson et al. [173] **🟢**  (English, USA, North America) | 27  ♀ (100%)  11.5±1.7 | Artistic Gymnastics | Tiers 2 and 3 | Internal Department Funds, Brigham Young University | The authors declared no competing interests | Acute  Unreported  Warm-up |
| Jordan et al. [174] **🟢**  (English, USA, North America) | 14  ♂ (100%)  13.6±0.6 | Soccer | Tier 3 | Unreported | Unreported | Acute  Unreported  Warm-up |
| Junqueira et al. [175] **🟢**  (Portuguese, Brazil, South America) | 30  ♀ (100%)  Range: 14-21 | Basketball | Tier 2 | Unreported | Unreported | Chronic (2 weeks, unreported, unreported)  Unreported  Warm-up |
| Kafkas et al. [176] **🟡**  (English, Turkey, Europe and Asia) | 14  ♀ (100%)  22.5±2.5 | Swimming | Tier 3 | Unreported | The authors declared no competing interests | Acute  Unreported  Warm-up |
| Karloh et al. [177]  Karloh et al. [178] **🟡**  (Portuguese, Brazil, South America) | 8  ♀ (100%)  13.3±0.9 | Rhythmic Gymnastics | Tier 2 | Unreported | Unreported | Acute and chronic (6 weeks, 2x/week, 11 sessions)  Competitive season  Warm-up |
| Kazemi et al. [179] **🟢**  (English, Iran, Asia) | 24  ♂ (100%)  22.7±3.5 | Table Tennis | Tier 2 | Unreported | Unreported | Acute  Unreported  Warm-up |
| Kilit et al. [180] **🟢**  (English, Turkey, Europe and Asia) | 26  ♂ (100%)  13.4±0.3 | Tennis | Tier 3 | Unreported | The authors declared no competing interests | Acute  Pre-season  Warm-up |
| Konrad et al. [181] **🟢**  (English, Italy, Europe) | 18  ♂ (100%)  30.0±6.1 | Running Triathlon | Tier 2 | Grant (Project J4484) from Australian Science fund | The authors declared no competing interests | Acute  Unreported  Warm-up |
| Kornberg et al. [182] **🟢**  (English, Australia, Oceania) | 10  ♂: 5 (50.0%); ♀: 5 (50.0%)  Range: 18-30 | Track and Field | Tier 4 | Unreported | Unreported | Acute  Unreported  Warm-up |
| Krčmár et al. [183] **🟢**  (English, Slovakia, Europe) | 12  ♂: 10 (83.3%); ♀: 2 (16.7%)  20±1.1 | Ice hockey Karate Soccer Track and Field Volleyball | Tier 2 | Unreported | The authors declared no competing interests | Acute  Unreported  Warm-up |
| Kruse et al. [184]  Kruse et al. [185] **🟢**  (English, USA, North America) | 11 and 10  ♀ (100%)  20.0±1.6; 19.9±1.6 | Volleyball | Tier 3 | Unreported | Unreported | Acute  Off-season  Warm-up |
| Kurt [186] **🟢**  (English, Turkey, Europe and Asia) | 24  ♂ (100%)  22.7±3.3 | Judo Karate Muay Thai Taekwondo | Tier 2 | Unreported | The authors declared no competing interests | Acute  Unreported  Warm-up |
| Kurt, Firtin [187] **🟡**  (English, Turkey, Europe and Asia) | 20  Unreported  25.3±4.3 | Soccer | Tier 3 | No funding | The authors declared no competing interests | Acute  Pre-season  Warm-up |
| Kurtdere et al. [188] **🟢**  (English, Turkey, Europe and Asia) | 17  ♂: 6 (35.3%); ♀: 11 (64.7%)  20.5±1.6 | Judo | Tier 4 | No funding | The authors declared no competing interests | Acute  Unreported  Warm-up |
| Kyranoudis et al. [189] **🟢**  (English, Greece, Europe) | 24  Unreported  21.7±0.9 | Soccer | Tier 2 | Unreported | Unreported | Acute  Competitive season  Warm-up |
| Kyranoudis et al. [190] **🟢**  (English, Greece, Europe) | 16  Unreported  21.4±3.3 | Soccer | Tier 2 | Unreported | Unreported | Acute  Unreported  Warm-up |
| Laudner et al. [191] **🔴**  (English, USA, North America) | 33 ^c^  ♂ (100%)  19.8±1.3 | Baseball | Tier 3 | Unreported | Unreported | Acute  Unreported  Warm-up |
| Li et al. [192] **🟢**  (English, China, Asia) | 20  ♂ (100%)  21±1.1 | Swimming | Tier 2 | No funding | The authors declared no competing interests | Acute  Unreported  Warm-up |
| Lin et al. [193] **🟢**  (English, Taiwan, Asia) | 40  ♂: 25 (62.5%); ♀ 15 (37.5%)  21.4 | Badminton | Tier 2 | Supported by the NSYSU-KMU as joint research projects (NSYSUKMU 107-P021; NSYSUKMU 109-P005 | The authors declared no competing interests | Acute  Unreported  Warm-up |
| Little, Williams [194] **🟡**  (English, United Kingdom, Europe) | 18  ♂ (100%)  Unreported | Soccer | Tier 3 | Unreported | Unreported | Acute  Competitive season  Warm-up |
| Lotfi et al. [195] **🟡**  (English, Iran, Asia) | 12  Unreported  14.8±0.4 | Futsal | Tier 2 | Unreported | The authors declared no competing interests | Acute  Unreported  Warm-up |
| Loughran et al. [196] **🟢**  (English, United Kingdom, Europe) | 17  ♂ (100%)  20.9 (range 18-30) | Gaelic Football | Tier 3 | No funding | The authors declared no competing interests | Acute  Unreported  Warm-up |
| Lowery et al. [197] **🟢**  (English, USA, North America) | 10  ♂ (100%)  24±5 | Running | Tier 3 | Unreported | Unreported | Acute  Unreported  Warm-up |
| Makaruk et al. [198] **🟢**  (English, Poland, Europe) | 30  ♂ (100%)  19.3±4.0 | Track and Field (sprint and jump) | Tier 2 | Unreported | Unreported | Chronic (4 weeks, 3x/week, 12 sessions)  Off-season  Warm-up |
| Manzi et al. [199] **🟢**  (English, Italy, Europe) | 24  Unreported  Global postural re-education: 27.1±4.5; Stretching with vibration: 28.6±2.7; Stretching without vibration: 27.7±5.3 | Soccer | Tier 3 | Unreported | Unreported | Chronic (4 weeks, 3x/week, 12 sessions)  Pre-season  Unreported |
| Mariscal et al. [200] **🟢**  (English, Spain, Europe) | 33  ♂ (100%)  Youth: between 14 and 15 (n=13); Adults: 19 and older (n=20) | Soccer | Tier 2 | No funding | The authors declared no competing interests | Acute  Competitive season  Warm-up |
| Martin et al. [201] **🟡**  (English, Slovakia, Europe) | 12  Unreported  22.9±3.4 | Ice Hockey | Tier 3 | VEGA 1/0414/15 | Unreported | Chronic (3 weeks, 1x/week, 3 sessions)  Unreported  Warm-up |
| Martinez-Chicote et al. [202] **🟢**  (English, Spain, Europe) | 20  ♂: 14 (70.0%); ♀: 6 (30.0%)  17.8±1.7 | Tennis | Tier 2 | Unreported | Unreported | Acute  Unreported  Warm-up |
| Mascarin et al. [203] **🟢**  (English, Brazil, South America) | 21  ♀ (100%)  16.2±1.0 | Handball | Tier 4 | Unreported | Unreported | Acute  Competitive season  Warm-up |
| McNeal, Sands [204] **🟢**  (English, USA, North America) | 13  ♀ (100%)  13.3±2.6 | Artistic Gymnastics | Tier 2 | Unreported | Unreported | Acute  Unreported  Warm-up |
| McNeal et al. [205] **🟢**  (English, USA, North America) | 22  ♀ (100%)  13.8±2.3 | Artistic Gymnastics | Tiers 2 and 3 | Unreported | Unreported | Acute  Competitive season  Warm-up |
| Meerits et al. [206] **🟡**  (English, Estonia, Europe) | 12  ♂ (100%)  22.0±2.1 | Track and Field (sprint, jumps, decathlon) | Tier 3 | Ministry of Education and Research project IUT20-58 | Unreported | Acute  Unreported  Warm-up |
| Melocchi et al. [207] **🟡**  (English, Italy, Europe) | 8  ♀ (100%)  14.1±2.0 | Artistic Gymnastics | Tier 3 | No funding | The authors declared no competing interests | Acute  Unreported  Warm-up |
| Mendez-Sanchez et al. [12] **🟢**  (English, Spain, Europe) | 8  ♂ (100%)  21±3 | Soccer | Tier 2 | No funding | The authors declared no competing interests | Acute  Unreported  Warm-up |
| Merrigan et al. [208] **🟡**  (English, USA, North America) | 19  ♂: 10 (52.6%); ♀: 9 (47.4%)  ♂: 19.7±1.0; ♀: 19.3±1.3 | Swimming | Tier 3 | No funding | The authors declared no competing interests | Acute  Unreported  Post-exercise |
| Mikolajec et al. [209] **🟡**  (English, Poland, Europe) | 14  ♂ (100%)  23.2±3.8 | Basketball | Tier 3 | Unreported | Unreported | Acute  Pre-season  Warm-up |
| Miladi et al. [210] **🟢**  (English, France, Europe) | 10  Unreported  25.7±2.4 | Soccer | Tier 2 | Unreported | Unreported | Acute  Competitive season  Inter-set |
| Mojock et al. [211] **🟢**  (English, USA, North America) | 12  ♀ (100%)  30±9 | Track and Field (distance running) | Tier 2 | Unreported | Unreported | Acute  Unreported  Warm-up |
| Molacek et al. [212] **🟢**  (English, USA, North America) | 15  ♂ (100%)  19.9±1.1 | American Football | Tier 3 | Unreported | Unreported | Acute  Off-season  Warm-up |
| Montalvo, Dorgo [213] **🟢**  (English, USA, North America) | 11  ♂: 9 (81.8%); ♀: 2 (18.2%)  23.2±2.5 | Artistic Gymnastics | Tiers 2 and 3 | No funding | The authors declared no competing interests | Acute  Unreported  Warm-up |
| Moore, Hutton [214] **🟢**  (English, USA, North America) | 21  ♀ (100%)  Range: 17-23 | Artistic Gymnastics | Tier 2 | University of Washington, Graduate School Research Fund Biomedical Sciences Support Grant, RR-07096, NIH | Unreported | Acute  Unreported  Warm-up |
| Mor et al. [215] **🟡**  (Turkish, Turkey, Europe and Asia) | 22  ♂ (100%)  11.5±0.5 | Soccer | Tier 2 | Unreported | Unreported | Acute  Unreported  Warm-up |
| Moran et al. [216] **🟢**  (English, Ireland, Europe) | 18  ♂ (100%)  23.2±3.2 | Golf | Tier 3 | Unreported | Unreported | Acute  Unreported  Warm-up |
| Moran et al. [217] **🟢**  (English, USA, North America) | 15  ♂: 11 (68.8%); ♀: 5 (31.3%) ^f^  ♂: 21.3 (no SD or range); ♀: 19.6 (no SD or range) | Swimming | Tier 3 | Unreported | Unreported | Acute  Off-season  Warm-up |
| Moreno-Perez et al. [218] **🟢**  (English, Spain, Europe) | 26  ♂ (100%)  19.2±4.2 | Tennis | Tier 4 | Universidad Francisco de Vitoria and Banco Santander via grants (UFV-2020/44) | The authors declared no competing interests | Acute  Pre-season  Warm-up |
| Needham et al. [219] **🟢**  (English, United Kingdom, Europe) | 20  Unreported  17.2±1.2 | Soccer | Tier 3 | Unreported | Unreported | Acute  Competitive season  Warm-up |
| Nelson et al. [220] **🟢**  (English, USA, North America) | 16  ♂: 11 (68.8%); ♀: 5 (31.3%)  ♂: 21±2; ♀: 19±1 | Track and Field (multiple event athletes, including sprints, jumps, decathlon) | Tier 3 | Unreported | Unreported | Acute  Competitive season  Warm-up |
| Nobre et al. [221] **🟢**  (English, Brazil, South America) | 18  ♂ (100%)  14.0±1.1 | Handball | Tier 2 | CAPES Brazilian support agency | The authors declared no competing interests | Chronic (unreported, unreported, 16 sessions)  Unreported  Warm-up |
| Notarnicola et al. [222] **🟢**  (English, Italy, Europe) | 30  ♂ (100%)  17±1 | Basketball | Tier 2 | No funding | The authors declared no competing interests | Chronic (20 weeks, 2x/week, 40 sessions)  Unreported  Post-exercise |
| Nuri et al. [223] **🟢**  (English, Iran, Asia) | 30  ♀ (100%)  Active warm-up: 19.3±3.1; Passive warm-up: 20.0±1.2; Stretching: 19.8±5.7 | Taekwondo | Tier 3 | Unreported | The authors declared no competing interests | Acute  Unreported  Warm-up |
| O'Sullivan et al. [224] **🟢**  (English, Ireland, Europe) | 18 ^g^  ♂: 16 (88.9%); ♀: 2 (11.1%)  21±1 | Unclear: "involved in competitive sports" | Tier 2 | Unreported | The authors declared no competing interests | Acute  Unreported  Warm-up |
| Ohshita, Mitsuzono [10] **🟢**  (Japanese, Japan, Asia) | 22  ♂ (100%)  Controls: 20.9±1.3; Static active stretching: 22.5±3.2; Static passive stretching: 22.6±3.7 | Track and Field (long-distance running) | Tier 4 ^h^ | Unreported | Unreported | Acute  Unreported  Inter-set |
| Olivares-Arancibia et al. [225] **🟢**  (English, unclear country [Chile or Spain], South America or Europe) | 70  ♂: 57 (81.4%); ♀: 13 (18.6%)  21.0±1.9 | Basketball Soccer Volleyball | Tier 2 | Unreported | Unreported | Acute  Unreported  Warm-up |
| Oliveira et al. [226] **🟢**  (English, Brazil, South America) | 12  ♂ (100%)  17.7±0.9 | Soccer | Tier 3 | São Paulo Research Foundation (FAPESP: 2014/16164-5) | The authors declared no competing interests | Acute  Pre-season  Warm-up |
| Oña Tacan et al. [227] **🟡**  (English, Equator, South America) | 36  ♂ (100%)  Range: 14-15 | Karate | Tier 2 | Unreported | The authors declared no competing interests | Chronic (20 weeks, 3x/week, 60 sessions)  Competitive season  Unreported |
| Oskouei et al. [228] **🟢**  (English, Iran, Asia) | 12  ♂ (100%)  22.4±2.9 | Soccer | Tier 2 | No funding | The authors declared no competing interests | Acute  Unreported  Warm-up |
| Osternig et al. [229] **🟡**  (English, USA, North America) | 20 ^c^  ♂: 10 (50.0%); ♀: 10 (50.0%)  24.6±5.0, but this included 10 controls that failed our eligibility criteria | Track and Field (distance runners, sprinters) Volleyball | Tier 2 | Unreported | Unreported | Acute  Unreported  Warm-up |
| Oyama et al. [9] **🟡**  (English, USA, North America) | 15  ♂ (100%)  20.4±1.4 | Baseball | Tier 2 | No funding | The authors declared no competing interests | Acute  Off-season  Independent sessions |
| Pagaduan et al. [230] **🟢**  (English, Bosnia and Herzegovina, Europe) | 29  ♂ (100%)  19.4±1.1 | Soccer | Tier 2 | Unreported | Unreported | Acute  Unreported  Warm-up |
| Panidi et al. [231] **🟢**  (English, Greece, Europe) | 21  ♀ (100%)  13.5±1.4 | Volleyball | Tier 2 | Austrian Science Fund FWF (Project P 32078-B) | The authors declared no competing interests | Chronic (12 weeks, 5x/week, 60 sessions)  Unreported  Warm-up |
| Papadimitriou et al. [232] **🟡**  (English, Greece, Europe) | 23  ♂: 11 (47.8%) ; ♀: 12 (52.2%)  13±2 | Swimming | Tier 2 | Unreported | Unreported | Chronic (8 weeks, 3x/week, 24 sessions)  Unreported  Warm-up |
| Papia et al. [233] **🟢**  (English, Greece, Europe) | 19  ♀ (100%)  9.8±0.5 | Artistic Gymnastics | Tier 2 | Unreported | Unreported | Acute  Competitive season  Warm-up |
| Pellegrini et al. [234] **🟢**  (English, Italy, Europe) | 11  ♂ (100%)  Range: 16-19 | Baseball | Tier 2 | Unreported | The authors declared no competing interests | Chronic (4, 1x/week for physiotherapist-assisted stretches, 2x/week for home-based stretches, 4 and 8 sessions)  Competitive season  Independent sessions |
| Penichet-Tomas et al. [235] **🟢**  (English, Spain, Europe) | 8  ♂ (100%)  24.8±3.4 | Rowing | Tier 2 | No funding | The authors declared no competing interests | Acute  Unreported  Unreported |
| Pojskic et al. [236] **🟢**  (English, Bosnia and Herzegovina, Europe) | 21  ♂ (100%)  20.1±1.7 | Soccer | Tier 2 | Unreported | The authors declared no competing interests | Acute  Unreported  Warm-up |
| Polat et al. [237] **🟡**  (English, Turkey, Europe and Asia) | 13  ♂ (100%)  20.2±2.1 | Wrestling (free style) | Tier 3 | Unreported | The authors declared no competing interests | Acute  Unreported  Warm-up |
| Pooley et al. [238] **🟡**  (English, United Kingdom, Europe) | 10  ♂ (100%)  16±1 | Soccer | Tier 3 | Unreported | The authors declared no competing interests | Acute  Competitive season  Post-exercise |
| Pooley et al. [239] **🟡**  (English, United Kingdom, Europe) | 15  ♂ (100%)  16±1 | Soccer | Tier 3 | No funding | The authors declared no competing interests | Acute  Competitive season  Post-exercise |
| Popelka, Pivovarniček [240]  Popelka et al. [241] **🟡**  (English, Slovakia, Europe) | 10  Unreported  15.4±0.5 | Volleyball | Tier 2 | VEGA 1/0583/18 | Unreported | Chronic (10 weeks, 1x/week, 10 sessions)  Competitive season  Warm-up |
| Popelka, Pivovarniček [242] **🟢**  (English, Slovakia, Europe) | 16  Unreported  13.4±0.5 | Volleyball | Tier 2 | Department of Physical Education and Sports, Faculty of Arts, Matej Bel University in Banská Bystrica | Unreported | Chronic (6 weeks, 1x/week, 6 sessions)  Competitive season  Warm-up |
| Portilla-Dorado et al. [243] **🟢**  (Spanish, Colombia, South America) | 23  ♂ (100%)  24.4±3.9 | Indoor Soccer | Tier 3 | Unreported | The authors declared no competing interests | Chronic (8 weeks, 3x/week, 24 sessions)  Unreported  Warm-up |
| Racil et al. [244] **🟢**  (English, Tunisia, Africa) | 34  ♂ (100%)  15.7±0.7 | Track and Field (hurdlers) | Tier 2 | No funding | The authors declared no competing interests | Chronic (12 weeks, 4x/week, 48 sessions)  Unreported  Warm-up |
| Reis et al. [245] **🟢**  (English, Brazil, South America) | 17 ^c^  ♂ (100%)  25.7±4.8 | Indoor Soccer | Tier 2 | Unreported | The authors declared no competing interests | Acute  Unreported  Warm-up |
| Reuther et al. [246] **🟡**  (English, USA, North America) | 17  ♂ (100%)  17.7±0.9 | Baseball | Tier 2 | No funding | The authors declared no competing interests | Chronic (1 week, 5x/week, 5 sessions)  Unreported  Post-exercise |
| Robey et al. [247] **🟢**  (English, Australia, Oceania) | 20  ♂: 12 (60.0%); ♀: 8 (40.0%)  Club: 20.2±2.2; Elite: 21.1±3.6 | Rowing | Tiers 2 and 3 | Unreported | Unreported | Acute  Competitive season  Post-exercise |
| Rodriguez-Marroyo et al. [248] **🟢**  (English, Spain, Europe) | 16  ♂ (100%)  15.7±0.4 | Soccer | Tier 2 | Unreported | Unreported | Chronic (6 weeks, 2x/week, 12 sessions)  Competitive season  Post-exercise |
| Rogan et al. [249] **🟡**  (German, Switzerland, Europe) | 12  ♂ (100%)  Elite A 18.8±1.0; Junioren B 16.8±0.4 | Ice Hockey | Tier 2 | Unreported | The authors declared no competing interests | Acute  Competitive season  Warm-up |
| Romero-Franco et al. [250] **🟢**  (English, Spain, Europe) | 32  ♂ (100%)  Controls: 25.1±5.0; Dynamic stretching: 25.8±3.8; Static stretching: 24.9±4.6 | Track and Field (sprinters) | Tier 3 | Unreported | The authors declared no competing interests | Acute  Competitive season  Warm-up |
| Sagiroglu et al. [251] **🟢**  (English, Turkey, Europe and Asia) | 16  ♂ (100%)  23.9±3.7 [3.6 in abstract, 3.7 in body of manuscript] | Judo Karate Muay Thai Taekwondo | Tier 2 | Unreported | The authors declared no competing interests | Acute  Unreported  Warm-up |
| Sampaio-Jorge et al. [252] **🟢**  (English, Brazil, South America) | 6  ♂ (100%)  25.6±4.2 | Basketball | Tier 3 | FAPERJ (unclear code) | Unreported | Acute  Pre-season  Warm-up |
| Sánchez-Sánchez et al. [253] **🟢**  (Spanish, Spain, Europe) | 17  ♂: 10 (58.8%); ♀: 7 (41.2%)  20.8±1.1 | Basketball Futsal Handball Soccer | Tier 2 | Unreported | Unreported | Acute  Unreported  Warm-up |
| Sands et al. [254] **🟢**  (English, USA, North America) | 11  ♀ (100%)  20.6±2.5 | Synchronized Swimming | Tier 4 | Unreported | Unreported | Acute  Pre-season  Post-exercise |
| Sands et al. [255] **🟢**  (English, USA, North America) | 10  ♂ (100%)  10.1±1.5 | Artistic Gymnastics | Tier 4 | U.S. Elite Coaches Association for Women's Gymnastics | Unreported | Acute and chronic (4 weeks, 5x/week, 20 sessions)  Unreported  Warm-up |
| Satkunskiene et al. [13] **🟢**  (English, Lithuania, Europe) | 15  ♂ (100%)  18.0±1.4 | Soccer | Tier 2 | Unreported | Unreported | Acute  Unreported  Warm-up |
| Sauers et al. [14] **🟡**  (English, USA, North America) | 30  ♂ (100%)  20.2±1.2 | Baseball | Tier 2 | Unreported | Unreported | Acute  Unreported  Warm-up |
| Sayers et al. [256] **🟢**  (English, USA, North America) | 20  ♀ (100%)  Range: 18-29 | Soccer | Tier 3 | Unreported | Unreported | Acute  Competitive season  Warm-up |
| Schmitt et al. [257]  Schmitt et al. [258] **🟢**  (English, Canada, North America) | 8 ^i^  ♀ (100%)  Range: 17-24 | Soccer | Tier 3 | National Research Council of Canada - Industrial Research Assistance Project #02753E | Unreported | Chronic (2 weeks, 5x/week, 10 sessions)  Unreported  Warm-up |
| Seçer, Kaya [259] **🟢**  (English, Turkey, Europe and Asia) | 30  ♂ (100%)  18.8±0.7 | Soccer | Tier 2 | No funding | Unreported | Acute  Unreported  Warm-up |
| Sekir et al. [260]  Sekir et al. [261]  Sekir et al. [8] **🟢**  (English, Turkey, Europe and Asia) | 12  ♀ (100%)  20±2 | Track and Field (several different events) | Tier 3 | Unreported | Unreported in two of the publications. The authors declared no competing interests in only one of the publications. | Acute  Unreported  Warm-up |
| Selkar et al. [262] **🟢**  (English, India, Asia) | 40  ♂ (100%)  Range: 18-24 or 25 (inconsistent reporting) | Track and Field (long-distance running) | Tier 2 | Unreported | Unreported | Chronic (2 weeks, unreported, unreported)  Unreported  Warm-up |
| Sermaxhaj et al. [263]  Sermaxhaj et al. [264]  Sermaxhaj et al. [265]  Sermaxhaj et al. [266]  Sermaxhaj et al. [267] **🟡**  (English, Kosovo, Europe) ^j^ | 68  Unreported  U13 team: 12.1±0.3; U15 team: 13.9±0.5; U17 team: 15.6±0.4 | Soccer | Tiers 2 and 3 | Unreported | Unreported in three of the publications. The authors declared no competing interests in two of the publications. | Chronic (16 weeks, 3x/week, 48 sessions)  Competitive season  Post-exercise |
| Sheard, Paine [268] **🟢**  (English, United Kingdom, Europe) | 56  ♂: 37 (66.1%); ♀: 19 (33.9%)  ♂: 21.1±1.3; ♀: 21.5±1.0 | Netball Rugby Soccer | Tier 3 | Unreported | Unreported | Acute  Competitive season  Warm-up |
| Shekadar et al. [269] **🟡**  (English, India, Asia) | 60  ♂ (100%)  Range: 18-30 | Volleyball | Tier 2 | No funding | The authors declared no competing interests | Acute  Unreported  Unclear |
| Shitara et al. [270] **🟢**  (English, Japan, Asia) | 113  ♂ (100%)  Range: 15-17 | Baseball | Tier 2 | Unreported | Unreported | Chronic (~21 weeks, 7x/week, 150 sessions)  Pre-season  At night |
| Siatras et al. [271] **🟢**  (English, Greece, Europe) | 11  ♂ (100%)  9.8±0.8 | Artistic Gymnastics | Tier 2 | Unreported | Unreported | Acute  Competitive season  Warm-up |
| Silva et al. [272] **🟢**  (English, Brazil, South America) | 13  ♀ (100%)  14.5±2.4 | Rhythmic Gymnastics | Tier 4 | Unreported | Unreported | Acute  Unreported  Warm-up |
| Sim et al. [273] **🟡**  (English, Australia, Oceania) | 13  ♂ (100%)  24±2 | Australian Rules Football Rugby Soccer | Tier 2 | Unreported | Unreported | Acute  Pre-season  Warm-up |
| Skarabot et al. [274] **🟢**  (English, Slovenia, Europe) | 11  ♂: 6 (54.5%); ♀: 5 (45.5%)  15.3±1.0 | Swimming | Tier 2 | Unreported | Unreported | Acute  Unreported  Warm-up |
| Solon Júnior, Neto [275] **🟢**  (Portuguese, Brazil, South America) | 13  ♂ (100%)  26.9±2.5 | Volleyball | Tier 2 | Unreported | Unreported | Acute  Unreported  Warm-up |
| Song et al. [276] **🔴**  (English, China, Asia) | 15  ♂ (100%)  14.0±1.2 | Track and Field | Tier 2 | National Natural Science Foundation of China (81772423) | The authors declared no competing interests | Acute  Unreported  Warm-up |
| Stevanovic et al. [277] **🟡**  (English, Serbia, Europe) | 12  ♂ (100%)  18.0±0.4 | Basketball | Tier 2 | Ministry for Education, Science and Tecnhological Development of Republic of Serbia (project number 175012) | The authors declared no competing interests | Acute  Unreported  Warm-up |
| Stewart et al. [278] **🟢**  (English, Australia, Oceania) | 14  Unreported  Under-19 (no additional information) | Rugby league | Tier 3 | Unreported | Unreported | Acute  Competitive season  Warm-up |
| Stojanovic et al. [279] **🟢**  (English, Serbia, Europe) | 23  Unreported  15.3±0.8 | Basketball | Tier 2 | No funding | The authors declared no competing interests | Acute  Competitive season  Warm-up |
| Su et al. [280] **🟢**  (English, China, Asia) | 60  Unreported  20.1±1.3 | Soccer | Tier 2 | Unreported | Unreported | Acute  Unreported  Warm-up |
| Sudhakar, Padmasheela [281] **🟢**  (English, India, Asia) | 30  ♂ (100%)  Range: 18-24 | Volleyball | Tier 2 | Unreported | Unreported | Chronic (4 weeks, 4x/week, 16 sessions)  Unreported  Warm-up |
| Taber et al. [282] **🟢**  (English, USA, North America) | 10  ♂ (100%)  19.9±1.5 | American Football | Tier 3 | No funding | The authors declared no competing interests | Acute  Unreported  Warm-up |
| Takeuchi, Tsukuda [283] **🟢**  (English, Japan, Asia) | 14  ♂ (100%)  20.2±0.7 | Basketball | Tier 2 | No funding | The authors declared no competing interests | Acute  Unreported  Warm-up |
| Taleb-Beydokhti, Haghshenas [284] **🟢**  (English, Iran, Asia) | 12  ♂ (100%)  19.7±4.0 | Handball | Tier 2 | Unreported | Unreported | Acute and chronic (6 weeks, 2x/week, 12 sessions)  Unreported  Warm-up |
| Tammam, Hashem [285] **🟡**  (English, Saudi Arabia, Asia) | 37  ♂ (100%)  16.4±0.5 | Volleyball | Tier 2 | Deanship of Scientific Research (EAR-2017-1-8-F 7521), Northern Border University, Arar, K.S.A. | Unreported | Chronic (6 weeks, 3x/week, 18 sessions)  Off-season  Warm-up |
| Taylor et al. [286] **🟡**  (English, Australia, Oceania) | 13  Unreported  19.6±0.8 | Netball | Tier 3 | Unreported | Unreported | Acute  Unreported  Warm-up |
| Toft et al. [287] **🔴**  (English, Denmark, Europe) | 12  ♂ (100%)  Range: 23-31 | Handball Soccer | Tier 2 | Unreported | Unreported | Chronic (3 weeks, 14x/week,42 sessions)  Competitive season  Warm-up |
| Torres et al. [288] **🟢**  (English, USA, North America) | 11  ♂ (100%)  19.6±1.7 | Track and Field (throwers) | Tier 3 | Unreported | Unreported | Acute  Pre-season  Warm-up |
| Tsolakis et al. [289] **🟢**  (English, Greece, Europe) | 20  ♂: 10 (50.0%); ♀: 10 (50.0%)  ♂: 20.9±4.2; ♀: 22.5±2.3 | Fencing | Tier 4 | Unreported | Unreported | Acute  Unreported  Warm-up |
| Turki et al. [290] **🟢**  (English, Tunisia, Africa) | 16  ♂ (100%)  20.9±1.3 | Handball Soccer | Tier 3 | Tunisian Ministry of Scientific Research, Technology and Development of Competences | Unreported | Acute  Competitive season  Warm-up |
| Turki et al. [291] **🟢**  (English, Tunisia, Africa) | 16  ♂ (100%)  16.9±0.3 | Volleyball | Tier 4 | Unreported | Unreported | Acute  Competitive season  Warm-up |
| Turna et al. [292] **🟢**  (English, Turkey, Europe and Asia) | 30  ♂: 15 (50.0%); ♀: 15 (50.0%)  ♂: 17.0±0.8; ♀: 17.1±0.7 | Archery | Tier 2 | Unreported | The authors declared no competing interests | Acute  Unreported  Warm-up |
| Unick et al. [293] **🟢**  (English, USA, North America) | 16  ♀ (100%)  19.2±1.0 | Basketball | Tier 3 | Unreported | Unreported | Acute  Pre-season  Warm-up |
| Valdivia et al. [294] **🟢**  (Spanish, Mexico, North America) | 16  ♀ (100%)  19.0±2.7 | Soccer | Tier 2 | Unreported | Unreported | Chronic (4 weeks, 5x/week, 20 sessions)  Unreported  Post-exercise |
| Van Gelder, Bartz [295] **🟢**  (English, USA, North America) | 18 ^c^  ♂ (100%)  20.0±1.5, but this included 42 recreational players beyond the 18 collegiate players | Basketball | Tier 3 | GVSU S3 Grant | Unreported | Acute  Off-season  Warm-up |
| Van Zyl et al. [296] **🟢**  (English, South Africa, Africa) | 52  ♀ (100%)  Range: 8-10 | Artistic Gymnastics | Tier 2 | Unreported | Unreported | Acute  Unreported  Warm-up |
| Vasconcellos et al. [297] **🟡**  (Portuguese, Brazil, South America) | 45  ♂ (100%)  14.0±0.7 | Soccer | Tier 2 | Unreported | Unreported | Acute  Unreported  Warm-up |
| Vasconcellos et al. [298] **🟢**  (English, Brazil, South America) | 45  ♂ (100%)  14.0±0.7 | Soccer | Tier 2 | Grants from the Carlos Chagas Filho Foundation for Research Support in Rio de Janeiro (FAPERJ) and the Brazilian National Council for Scientific and Technological Development (CNPq) | The authors declared no competing interests | Acute  Competitive season  Warm-up |
| Veevo et al. [299] **🟡**  (English, Estonia, Europe) | 9  ♀ (100%)  22.0±0.8 | Basketball | Tier 3 | Unreported | Unreported | Acute  Unreported  Warm-up |
| Velasque et al. [300] **🟢**  (English & Italian, Brazil, South America) | 14  ♂ (100%)  16.2±0.5 | Soccer | Tier 2 | Unreported | The authors declared no competing interests | Acute  Unreported  Warm-up |
| Walker et al. [301] **🟡**  (English, USA, North America) | 11  ♂ (100%)  24.1± 4.1 | American Football | Tier 2 | Unreported | The authors declared no competing interests | Acute  Unreported  Warm-up |
| Wallmann et al. [302] **🟢**  (English, USA, North America) | 12  ♀ (100%)  19.2 (range 18-25) | Soccer | Tier 3 | Unreported | Unreported | Acute  Unreported  Warm-up |
| Walsh [303] **🟢**  (English, United Kingdom, Europe) | 10  ♂: 7 (70.0%); ♀: 3 (30.0%)  20±1 | Rugby Union Soccer Tennis | Tier 2 | No funding | The authors declared no competing interests | Acute  Unreported  Warm-up |
| Werstein, Lund [304] **🟢**  (English, USA, North America) | 15  ♀ (100%)  20.1±5.9 | Rugby Union Soccer | Tiers 2 and 3 | Unreported | Unreported | Acute  Off-season  Warm-up |
| West et al. [305] **🟢**  (English, USA, North America) | 12  ♂ (100%)  21.3±2.3 | Cycling | Tier 2 | Dr. Mike Greenwood’s Exercise and Sport Nutrition Laboratory external funding as well as Alter-G’s, Inc. (Fremont, CA) donation of the G-Trainer Anti-Gravity Treadmill | Unreported | Acute  Unreported  Post-Exercise |
| Williams et al. [306] **🟢**  (English, USA, North America) | 29  ♂: 4 (13.8%); ♀: 25 (86.2%)  19.5±1.2 | Swimming | Tier 3 | Unreported | Unreported | Acute  Unreported  Warm-up |
| Wilson et al. [307] **🟢**  (English, USA, North America) | 10  ♂ (100%)  25±7 | Track and Field (long-distance running) | Tier 2 | Unreported | Unreported | Acute  Off-season  Warm-up |
| Winchester et al. [308] **🟡**  (English, USA, North America) | 22  ♂: 11 (50.0%); ♀: 11 (50.0%)  ♂: 20.2±1.3; ♀: 20.3±1.2 | Track and Field (Jump events and multi-events) | Tier 3 | Unreported | Unreported | Acute  Competitive season  Warm-up |
| Wong et al. [309] **🟢**  (English, China, Asia) | 20  Unreported  16.8±0.4 | Soccer | Tier 2 | Unreported | The authors declared no competing interests | Chronic (1 week, 3x/week, 3 sessions)  Off-season  Warm-up |
| Yamaguchi et al. [310] **🟢**  (English, Japan, Asia) | 7  ♂ (100%)  21.3±2.1 | Track and Field (long-distance running) | Tier 2 | JSPS KAKENHI Grant number 24700655 | Unreported | Acute  Off-season  inter-set |
| Yamaguchi et al. [311] **🟢**  (English, Japan, Asia) | 8  ♂ (100%)  19.9±1.1 | Track and Field (long-distance running) | Tier 2 | Japan Society for the Promotion of Science - JSPS KAKENHI Grant no.JP26750273 | Unreported | Acute  off-season  Warm-up |
| Yamaguchi et al. [312] **🟢**  (English, Japan, Asia) | 16  ♂ (100%)  20.9±2.1 | Track and Field (long-distance running) | Tier 2 | Japan Society for the Promotion of Science - JSPS KAKENHI Grant no.JP26750273 and JP24700655 | Unreported | Acute  off-season  Warm-up |
| Yaşli, Müniroğlu [313] **🟢**  (Turkish, Turkey, Europe and Asia) | 18  ♂ (100%)  21.8±4.2 | Soccer | Tier 2 | Unreported | Unreported | Chronic (8 weeks, 3x/week, 24 sessions)  Unreported  Warm-up |
| Yildiz [314] **🟢**  (English, Turkey, Europe and Asia) | 42  Unreported  24.3±5.5 | Soccer | Tier 2 | Unreported | Unreported | Acute  Competitive season  Warm-up |
| Yıldırım et al. [315] **🟢**  (English, Turkey, Europe and Asia) | 12  ♂ (100%)  18.7±1.5 | Wrestling | Tier 4 | Unreported | The authors declared no competing interests | Acute  Unreported  Post-exercise |
| Young et al. [316] **🟢**  (English, Australia, Oceania) | 16  ♂ (100%)  Range: 18-33 | Australian Rules Football | Tier 2 | Unreported | Unreported | Acute  Unreported  Warm-up |
| Zakas et al. [317] **🟢**  (English, Greece, Europe) | 17  Unreported  15.8±0.6 | Soccer | Tier 3 | Unreported | Unreported | Acute  Unreported  Warm-up |
| Zakas [318] **🟢**  (English, Greece, Europe) | 15  Unreported  16.0±0.5 | Soccer | Tier 2 | Unreported | Unreported | Acute  Unreported  Warm-up |
| Zakas et al. [319] **🟢**  (English, Greece, Europe) | 16  ♂ (100%)  13.0±0.5 | Soccer | Tier 2 | Unreported | Unreported | Acute  Unreported  Warm-up |
| Zakas et al. [320] **🟢**  (English, Greece, Europe) | 14  ♂ (100%)  18.5±0.6 | Soccer | Tier 3 | Unreported | Unreported | Acute  Unreported  Warm-up |
| Zakas et al. [321] **🟢**  (English, Greece, Europe) | 15  Unreported  25.0±1.5 | Soccer | Tier 3 | Unreported | Unreported | Acute  Unreported  Warm-up |
| Zakas et al. [322] **🟢**  (English, Greece, Europe) | 18  Unreported  16.0±0.5 | Soccer | Tier 2 | Unreported | Unreported | Acute  Unreported  Warm-up |
| Zmijewski et al. [323] **🟢**  (English, Poland, Europe) | 13  ♀ (100%)  22.1±3.2 | Handball | Tier 2 | Unreported | Unreported | Acute  Transition from pre-season to competitive season  Warm-up |
| Zourdos et al. [324] **🟢**  (English, USA, North America) | 14  ♂ (100%)  23.0±4.3 | Running | Tier 2 | No funding | Unreported | Acute  Unreported  Warm-up |
| 孙勇 [325] **🟢**  (Chinese, China, Asia) | 20  ♂ (100%)  18.3±2.3 | Basketball | Tier 3 | Unreported | Unreported | Acute  Unreported  Warm-up |

*Legend (ordered by appearance)*: **🟢🟡🔴** Green stands for randomized trial, yellow for non-randomized multi-arm/multi-condition, red for non-randomized single-arm/single-condition. ♂ Female. ♀ Male. CMJ – Countermovement jump. IQR – Interquartile range. PCF – Participant Classification Framework [4]. PNF – Proprioceptive neuromuscular facilitation. SD – Standard deviation. ^a^ No mention to randomization in the 2011 paper: "aim to randomized”, but unclear whether this was ad hoc. ^b^ The authors stated the study was randomized, but only testing was randomized, not the interventions. ^c^ Excluding the participants that were not tier 2 or higher. ^d^ The authors did not explicitly mention competition, but the term used to describe the participants is fighters, which we interpreted as implying competition. ^e^ Assessed through information in the acknowledgments section. ^f^ Unclear if the non-analysed participant was male or female. ^g^ Excluding 18 previously injured subjects belonging to the experimental group; only the controls were considered. ^h^ Assessed through the reported times. ^i^ In Schmitt et al. [258], only experiment #2 was considered. ^j^ Some papers present only one of the teams, others present all the teams, but it is the same trial.

*Reporting details*: (i) Sample size refers only to athletes assessed and may differ from the initially reported study sample. (ii) Age was reported in years, rounded to one decimal case if sufficient data was available). Mean±SD were presented except if otherwise stated, and reported separately for different groups only if no common value was provided by the authors. (iii) Within-season timing: when not officially reported but dates were available, we tried our best to infer within-season timing based on typical competitive calendars for the specific country.

***ESM 2.3*** ***Intervention-level details***

*ESM 2.3.1. Stretching interventions*

PNF was applied in 36 trials (12.0%: 1 agonist contract-relax, 22 contract-relax, 4 contract-relax agonist contract, 1 contract-relax antagonist contract, 1 dynamic reversion, 2 hold-relax, 2 rhythmic stabilization, 5 unclear method; trials could have more than one PNF modality).

SGA^®^ and Mulligan’s stretching (static passive stretching with additional manual traction) in 2 trials (0.7%) each, SGA^®^ with passive component, global postural reeducation and Fauls stretching routine (static passive stretching combined with rolling and waving motions) [14] in 1 trial (0.3%) each.

Two trials (0.7%) provided information so scarce that it was impossible to ascertain which stretching modality was used [105, 195].

*ESM 2.3.2. Non-stretching comparators*

Non-stretching related comparators included aerobic-based activities (*e.g.*, cycling [210]; 13 trials, 4.3%), body tempering (1 trial, 0.3%), cold-water immersion (3 trials, 1.0%), foam rolling (7 trials, 2.3%), hot shower alternated with cold-water immersion (1 trial, 0.3%), hot water immersion (1 trial, 0.3%), local and/or whole-body vibration (not concomitantly with stretching; 2 trials, 0.7%), manual therapies (*e.g.*, massage [45]; 4 trials, 1.3%), moist heat pack application (1 trial, 0.3%), multimodal exercise and/or warm-up programs (*e.g.*, FIFA 11+ [45]; 20 trials, 6.7%), Pilates (2 trials, 0.7%), pool walking (1 trial, 0.3%), self-myofascial “release” (2 trials, 0.7%), sport-specific activities (*e.g.*, hurdle exercises [244], 2 trials, 0.7%), strength-based training (*e.g.*, resistance training [218], plyometrics [244]; 25 trials, 8.3%), ultrasound (1 trial, 0.3%), and walking (1 trial, 0.3%).

*ESM 2.3.3. Anatomical regions stretched*

The majority of trials (k=224, 74.7%) focused on stretching the lower limbs (Figure 3A), either globally (*e.g.*, [324]) or more locally (*e.g.*, quadriceps [233]), and an additional 13 trials (4.3%) stretched the lower limbs and the trunk. The upper limbs were stretched in 23 trials (7.7%), either globally (*e.g.*, [299]) or on more specific anatomical regions (*e.g.*, forearm and wrist [80]). Thirty-one trials (10.3%) stretched the full body. In six trials from 7 studies (2.0%), it was unclear which regions were stretched [29, 105, 195, 230, 240-242]. Three trials (1.0%) focused on trunk muscles [301, 306] or upper limbs and trunk [288].

*ESM 2.3.4. Number of stretches per intervention*

The number of stretching exercises was unreported in 14 trials (4.7%). Five trials (1.7%) reported the number of stretching exercises for one stretching modality, but not for the other(s) [64, 93, 175, 199, 222], while two trials had unclear reporting (possibly eight [109] and nine [115] stretches).

We refrained from calculating means and standard deviations, as there was considerable complexity that could result in miscalculations. For example, several trials had differing number of stretches for different stretching modalities (*e.g.*, 12 dynamic stretches vs 13 static active stretches [18]), with that difference reaching a threefold magnitude (*e.g.*, 15 stretches in the combined group vs five in the single modality group [196]). Some trials had combined stretching modalities with differing number of stretches representing each modality (*e.g.*, the athletes performed three static active stretches + one static passive stretching [19]). Other trials had interventions with multiple stretches, but only a subset was performed in each session (*e.g.*, four stretches overall, but only one performed in each session [26]).

*ESM 2.3.5. Number of sets*

In ten trials (3.3%) [10, 44, 45, 69, 96, 105, 135, 139, 195, 213] the number of sets was unreported and could not be assessed, and six trials (2.0%) [64, 93, 138, 152, 222, 253] reported the number of sets for one stretching modality but not for the others (*e.g.*, two sets for static active stretching, unclear number of sets for dynamic stretching [253]). Additionally, one trial had a vague description: “repeated until the participants were instructed to stop stretching” [88].

Occasionally, trials prescribed a different number of sets depending on whether the exercises were unilateral or bilateral (e.g., 1 set for bilateral stretches vs 2 sets for unilateral stretches [144], or vice-versa [51]). There were additional cases of non-uniform number of stretches being prescribed [98, 103, 273, 286] (e.g., 2 sets for the hamstrings and quadriceps, but only 1 set for soleus and gastrocnemius [273]). Three trials (1.0%) [89, 104, 165] had a variable number of sets (e.g., athletes could perform anywhere between 3 to 5 sets in [89]), with unclear criteria to define how this feature was monitored and whether it had an impact on the results.

*ESM 2.3.6. Number of repetitions per set*

Ten trials (3.3%%) did not report this information [10, 29, 44, 96, 105, 136, 211, 213, 234, 237], while seven trials (2.3%) [18, 93, 138, 152, 161, 222, 253] reported repetitions/time for some stretching interventions, but not all (*e.g.*, 5 s for static passive stretching, unreported for dynamic stretching [161]). Moreover, twelve trials (4.0%) [42, 55, 85, 101, 131, 146, 157, 170, 193, 219, 277, 283] had unclear descriptions for at least one of the stretching modalities, precluding a solid assessment of this information. This was very common for interventions using dynamic stretching, with the athletes performing the stretches across a specified distance (*e.g.*, across 18 m [193]), and therefore it was not possible to confidently assess the number of repetitions or the time.

When reported, this information was usually conveyed in time per set (commonly in seconds) for most stretching interventions (*e.g.*, static active, PNF), but that was not always the case for ballistic and dynamic stretching. In some trials, ballistic stretching was reported in time (*e.g.*, [226]), while in others it was reported in number of repetitions (*e.g.*, [297]). The same occurred with dynamic stretching (*e.g.*, time [61] vs repetitions [45]). For dynamic stretching, sometimes the reporting was mixed even within the same study (*e.g.*, 30 repetitions for bilateral stretches, 15 s on each side for unilateral stretches [30, 37, 38]). It was not always clear whether the number of repetitions was global, or for each side.

*ESM 2.3.7. Rest intervals*

Rest intervals were not applicable in seven trials (2.3%) (*e.g.*, [25]) and unreported in 106 trials (35.3%) (*e.g.*, [29]). Moreover, 13 trials (4.3%) reported rest intervals for some but not all of the stretching interventions (*e.g.*, 10 s rest for dynamic stretching, unreported for static active stretching [85]).

**Supplementary table 3.** Intervention-level details

| **Trials** | **Stretching modalities** | **Non-stretching comparators** (or stretching combined with other protocols) | **Anatomical regions stretched**  **No. stretches per intervention** | **No. sets**  **No. reps/time**  **Rest intervals** | **Minimum stretching volume per session** | **Stretching velocity**  **Stretching intensity** |
| --- | --- | --- | --- | --- | --- | --- |
| Abadi et al. [18] | DS SAS | No-stretching controls | Full-body  DS: 12 SAS: 13 | 2  DS: unreported SAS: 15 s  5 s | DS: insufficient information to assess SAS: 390 s | Unreported  Unreported |
| Agopyan et al. [19] | SAS + SPS | No-stretching controls | Lower limbs  SAS: 3 + SPS: 1 | 2  30 s  10 s | 240 s | Unreported  Maximum ROM while avoiding pain |
| Ahmadabadi et al. [20] | SAS | No-stretching controls | Full-body  13 | First two weeks: 2 Last two weeks: 3  15 s  5 s | First two weeks: 390 s Last two weeks: 585 s | Unreported  Unreported |
| Akarsu et al. [21] | SAS | No-stretching controls Self Myofascial release | Lower limbs  4 | 1  10 s  20 s | 40 s | Unreported  Unreported |
| Akehurst et al. [22] | SAS  SAS + vibration | — | Quadriceps  3 | 3  Two first stretches: 30 s Third stretch: 60 s  120 s | 360 s | Unreported  Unreported |
| Alipasali et al. [23]  Alipasali et al. [24] | DS SAS | No-stretching controls | Lower limbs  6 | 2  10 s  10 s for bilateral exercises 0 s for unilateral exercises | 120 s | Unreported  Maximum ROM while avoiding pain or soreness |
| Almeida Júnior et al. [25] | GAS^®^ | No-stretching controls | Upper limbs  1 | 1  900 s  Not applicable | 900 s | Unreported  Unreported |
| Almeida Júnior et al. [26] | GAS^®^ with passive component | Calisthenics | Full-body  4 (but only 1 per session) | 1  900 s  Not applicable | 900 s | Unreported  Unreported |
| Alp [27] | DS SAS | No-stretching controls | Lower limbs  4 | 3  30 s  20 s | 360 s | DS: as fast as they could SAS: unreported  DS: unreported SAS: to point of discomfort |
| Alp et al. [28] | DS SAS | No-stretching controls | Lower limbs  4 | 3  30 s  20 s | 360 s | DS: as fast as they could SAS: unreported  DS: unreported SAS: to point of discomfort |
| Amir Vazini, Parnow [29] | DS Static stretching (unclear if active or passive) | FIFA 11+ | Unreported  Unreported | 2  Unreported  Unreported | Insufficient information to assess | Unreported  Unreported |
| Amiri-Khorasani [30] | DS SAS | No-stretching controls | Lower limbs  6 | 1  DS: 30 reps (if bilateral) or 15 s each side (if unilateral) SAS: 15 s  Unreported | DS: ~180 s SAS: 90 s | DS: 1 repetition per second SAS: unreported  DS: unreported SAS: maximum ROM while avoiding pain |
| Amiri-Khorasani [31] | DS DS + SAS SAS | No-stretching controls | Lower limbs  DS: 6 DS + SAS: 12 SAS: 6 | DS: 4 DS + SAS: 4+1 SAS: 1  DS: 5 reps DS + SAS: 5 reps + 15 s SAS: 15 s  Unreported | DS: 120 reps DS + SAS: 120 reps + 90 s SAS: 90 s | DS: 1x no bouncing, 1x slow speed, 1x moderate speed, 1x as fast as possible SAS: unreported  DS: maximum ROM SAS: unreported |
| Amiri-Khorasani, Ferdinands [32] | DS SAS | No-stretching controls | Lower limbs  DS: 6 SAS: 6 | DS: 3 SAS: 1  DS: 5 reps SAS: 30 s  Unreported | DS: 90 reps SAS: 180 s | DS: 1x slow speed, 1x moderate speed, 1x as fast as possible SAS: gradually  DS: maximum ROM SAS: maximum ROM while avoiding pain |
| Amiri-Khorasani, Kellis [33] | DS  SAS | — | Quadriceps  1 | DS: 3 SAS: 1  DS: 5 reps SAS: 30 s  Unreported | DS: 15 reps SAS: 30 s | DS: 1x slow speed, 1x moderate speed, 1x as fast as possible SAS: gradually  DS: maximum ROM SAS: maximum ROM while avoiding pain |
| Amiri-Khorasani, Sotoodeh [34] | DS DS + SAS SAS | No-stretching controls | Lower limbs  DS: 6 DS + SAS: 12 SAS: 6 | 1  DS: 5 reps DS + SAS: 5 reps + 30 s SAS: 30 s  Unreported | DS: 30 reps DS + SAS: 30 reps + 180 s SAS: 180 s | DS: 1x slow speed, 1x moderate speed, 1x as fast as possible SAS: slowly  DS: maximum ROM SAS: unreported |
| Amiri-Khorasani et al. [35] | DS  SAS | — | Quadriceps  1 | DS: 3 SAS: 1  DS: 5 reps SAS: 30 s  Unreported | DS: 15 reps SAS: 30 s | DS: 1x slow speed, 1x moderate speed, 1x as fast as possible SAS: gradually  Unreported |
| Amiri-Khorasani et al. [36] | DS DS + SAS SAS SAS + DS | No-stretching controls | Lower limbs  DS: 6 DS + SAS: 12 SAS: 6 | DS: 3 DS + SAS: 3+1 SAS: 1  DS: 5 reps DS + SAS: 5 reps + 30 s (each side) SAS: 30 s (each side)  Unreported | DS: 90 reps DS + SAS: 90 reps + 360 s SAS: 360 s | DS: 1x slow speed, 1x moderate speed, 1x as fast as possible SAS: slowly  DS: maximum ROM SAS: unreported |
| Amiri-Khorasani et al. [37]  Amiri-Khorasani et al. [38] | DS SAS | No-stretching controls | Lower limbs  6 | 1  DS: 30 reps (if bilateral) or 15 s each side (if unilateral) SAS: 15 s  Unreported | DS: ~180 s SAS: 90 s | DS: 1 repetition per second SAS: unreported  DS: maximum ROM SAS: maximum ROM while avoiding pain |
| Amiri-Khorasani et al. [39] | DS DS + SAS SAS | No-stretching controls | Lower limbs  DS: 5 DS + SAS: 10 SAS: 5 | 1  DS: 30 reps (if bilateral) or 30 s each side (if unilateral) SAS: 15 s  Unreported | DS: ~150 s DS + SAS: ~300s SAS: 150 s | DS: 1 repetition per second SAS: unreported  DS: maximum ROM SAS: maximum ROM while avoiding pain |
| Andre et al. [40] | SPS | No-stretching controls | Upper limbs  4 | 1  30 s (two of the stretches were performed unilaterally)  Unreported | 180 s | Unreported  Unreported |
| Andrejić et al. [41] | PNF (CRAC) (2 sets) PNF (CRAC) (4 sets) SAS (2 sets) SAS (4 sets) | No-stretching controls | Lower limbs  PNF (CRAC): 2 SAS: 4 | LV: 2 HV: 4  PNF: ~40 s (each side) SAS: 20 s  5 s | LV PNF: 320 s High-volume PNF: 640 s LV SAS: 160 s HV SAS: 320 s | Unreported  To point of mild discomfort |
| Annino et al. [42] | DS  SAS | — | Lower limbs  8 | 1  DS: Unclear (walking half a basketball court three times each way) SAS: 30 s (each side)  Unreported | DS: insufficient information to assess SAS: 450 s | DS: unreported SAS: slowly  DS: progressed from moderate to high intensity SAS: maximum ROM |
| Ari [43] | DS DS + SPS SPS SPS + DS | No-stretching controls | Full-body  6 | DS: 2 DS + SPS: 1 SPS: 2 SPS + DS: 1  30 s (each side)  0 s | 720 s | DS: slowly SPS: unreported  Unreported |
| Arihiro et al. [44] | SAS | No-stretching controls Pilates | Lower limbs  6 | Unreported  Unreported  Unreported | Insufficient information to assess | Unreported  Unreported |
| Avaz et al. [45] | DS | No-stretching controls 12 exercises selected from FIFA 11+ Massage | Lower limbs  3 | Unreported  15 reps  Unreported | 45 reps | 1 repetition every 2 seconds  Unreported |
| Avedesian et al. [46] | SAS | Dynamic WU | Lower limbs  7 | 1  30 s (each side)  Unreported | 420 s | Unreported  To point of discomfort |
| Avloniti et al. [47] | SAS (10 s) SAS (15 s) SAS (20 s) SAS (30 s) SAS (40 s) SAS (60 s) | No-stretching controls | Lower limbs  5 | 1  10 s (each side) 15 s (each side) 20 s (each side) 30 s (each side) 40 s (each side) 60 s (each side)  0 s | 100 s (smaller duration group) to 600 s (larger duration group) | Unreported  To point of discomfort |
| Avloniti et al. [48] | SAS (20 s) SAS (30 s) SAS (40 s) SAS (60 s)  SAS (2x10 s) SAS (3x10 s) SAS (4x10 s) SAS (6x10 s) | No-stretching controls | Lower limbs  5 | Single set: 1 Multiple sets: 2, 3, 4 or 6  Single set: 20 s, 30 s, 40 s, 60 s (each side) Multiple sets: 10 s (each side)  Unreported | 200 s (smaller duration groups) to 1200 s (larger duration groups) | Unreported  To point of discomfort |
| Ayala, De Baranda [49]  De Baranda, Ayala [50] | DS  SAS SPS | — | Hamstrings  2 | 1  DS: 15 reps SAS: 30 s SPS: 30 s  Unreported | DS: 15 reps SAS: 60 s SPS: 60 s | DS: 1 repetition every 2 seconds SAS: unreported SPS: unreported  DS: maximum ROM SAS: feeling a stretch SPS: maximum ROM |
| Ayala et al. [51] | SAS | No-stretching controls | Lower limbs  4 | Bilateral: 2 Unilateral: 1 *Unclear how many exercises were bilateral and unilateral*  30 s  20 s | Insufficient information to assess | Unreported  Unreported |
| Aydoǧ et al. [52] | SAS | No-stretching controls | Lower limbs  6 | 3  30 s  Unreported | 540 s | Unreported  Unreported |
| Azuma, Someya [53] | SPS | No-stretching controls | Lower limbs  Unreported | 3  30 s  30 s | Insufficient information to assess | Unreported  Maximum ROM while avoiding pain |
| Babbar et al. [54] | DS Static stretching (unclear if active or passive) | No-stretching controls | Lower limbs  6 | 1  DS: 15 (each side) Static stretching: 30 s (each side)  Unreported | DS: 180 reps Static stretching: 360 s | DS: 5x slowly + 10x as quickly and powerful as possible without bouncing Static stretching: unreported  DS: unreported Static stretching: maximum ROM while avoiding pain |
| Balci et al. [11] | Neural stretching [variation of DS]  Neural sliding [variation of DS] | — | Lower limbs  1 | 3  60 s (each side)  30 s | 360 s | 1 repetition every 2 seconds  Unreported |
| Bali, Guru [55] | SPS  Mulligan's stretching [SPS + manual traction] | — | Hip adductors  1 | Mulligan stretching: 3 SPS: 4  Mulligan stretching: end ROM with 15 s hold + new end ROM (unclear total duration) SPS: 2x30 s  Mulligan stretching: Unreported SPS: 30 s | Mulligan stretching: insufficient information to assess SPS: 240 s | Unreported  Maximum ROM |
| Barbosa et al. [56] | SAS after running SAS before running | No intervention (rest 20 min) No-stretching controls | Lower limbs  3 | 3  30 s (each side)  30 s | 540 s | Unreported  To point of discomfort |
| Baumgart et al. [57] | Static stretching (unclear if active or passive) | No-stretching controls Soccer-specific training program | Lower limbs  4 | 2  20 s  10 s | 40 s | Unreported  To point of discomfort |
| Bazett-Jones et al. [58] | SAS | No-stretching controls | Hamstrings  1 | 4  45 s (each side)  45 to 60 s | 360 s | Unreported  To point of mild discomfort |
| Bazett-Jones et al. [59] | SPS | No-stretching controls Potentiation group: 3 x 3 reps at 90% 1RM | Lower limbs  4 | 3  30 s (each side)  20 s | 180 s | Unreported  Maximum ROM while avoiding pain |
| Beckett et al. [60] | Static stretching (unclear if passive or active) between sets of repeated sprint. Static stretching (unclear if passive or active) between sets of COD. | Rest between sets of repeated sprint Rest between sets of COD | Lower limbs  6 | 1  20 s (each side)  Unreported | 220 s | Unreported  To point of mild discomfort |
| Behara, Jacobson [61] | DS | Deep tissue roller No-stretching controls | Lower limbs  4 | 1  60 s (each side)  0 s | 480 s | Slowly  Unreported |
| Belkhiria-Turki et al. [62] | DS while moving (termed "active DS") DS while stationary (termed "static DS") | No-stretching controls | Lower limbs  5 | 2  14 reps  10 s | 140 reps | Self-selected pace, but performed slowly, smoothly and continuously, without ballistic or abrupt movements  Through active ROM |
| Belkhiria-Turki et al. [63] | DS (4 sets) DS (8 sets) DS (12 sets)  SPS (4 sets) SPS (8 sets) SPS (12 sets) | No-stretching controls | Lower limbs  3 | LV: 4 IV: 8 HV: 12  15 s  15 s | LV: 180 s IV: 360 s HV: 540 s | DS: 1 repetition per second SPS: slowly  DS: unreported SPS: until the subject verbally instructed the examiner to stop the stretching |
| Bello et al. [64] | PNF (RS)  SPS | — | Lower limbs  PNF (RS): 2 SPS: unreported | PNF (RS): 20 (2x10) SPS: Unreported  PNF (RS): 30 s SPS: 10 s  Unreported | PNF (RS): 1200 s SPS: insufficient information to assess | Unreported  With no pain |
| Ben Maaouia et al. [65] | DS SPS | No-stretching controls | Lower limbs  3 | 3  20 s  7 to 8 s | 180 s | DS: 1 repetition every 2 seconds SPS: Unreported  DS: unreported SPS: to point of mild discomfort |
| Bingul et al. [66] | DS SAS | No-stretching controls | Full-body  10 | DS: 1 SAS: 2  30 s  10 s | DS: 300 s SAS: 600 s | DS: 12 to 15 reps each 30 s SAS: 10 reps each 30 s  Unreported |
| Bishop, Middleton [67] | SAS | No-stretching controls | Lower limbs  7 | 1  20 s  Unreported | 140 s | Unreported  To point of discomfort |
| Bogdanis et al. [68] | SPS (continuous) SPS (intermittent) | No-stretching on contralateral limb | Lower limbs  1 | Continuous SPS: 1 Intermittent SPS: 3  Continuous SPS: 90 s Intermittent SPS: 30 s  Continuous SPS: 0 s Intermittent SPS: 30 s | 90 s | Unreported  To 90% of the point of discomfort |
| Boudenot et al. [69] | DS SPS | Ergocycle No-stretching controls | Lower limbs  Unreported | Unreported  15 s  Unreported | Insufficient information to assess | Unreported  Unreported |
| Bouthin, Edouard [70] | SAS | No-stretching controls | Lower limbs  5 | 2  10 s  0 s | 100 s | Unreported  Unreported |
| Brodowicz et al. [71] | SAS  SAS + heat (hot packs) SAS + ice (ice bags) | — | Hamstrings  4 | 1  180 s  Unreported | 720 s | Unreported  Unreported |
| Burkett et al. [72] | SAS | No-stretching controls Submaximal jump WU Weighted jump WU | Lower limbs  14 | 1  20 s  Unreported | 280 s | Unreported  Unreported |
| Burkett et al. [73] | SAS | No-stretching controls Submaximal jump WU Weighted jump WU | Lower limbs  14 | 1  20 s  Unreported | 280 s | Unreported  Unreported |
| Buttifant, Hrysomallis [74] | SAS | Box squats with barbell Box squats with elastic resistance bands | Lower limbs  4 | 2  30 s  Unreported | 240 s | Unreported  Unreported |
| Caliskan et al. [75] | SAS (2 min) [The authors erroneously termed it passive stretching]  SAS (5 min) [The authors erroneously termed it passive stretching] | — | Lower limbs  2 | 2-min group: 2 5-min group: 5  30 s  30 s | 2 min group: 120 s 5 min group: 300 s | Unreported  With no pain or discomfort |
| Caplan et al. [76] | PNF (CR)  SAS | — | Hamstrings  1 | 3  10 s  10 s | 30 s | Unreported  PNF (CR): until the subject reported a maximum stretch on a scale from 1 to 10. SAS: to point of discomfort |
| Carvalho et al. [77] | DS SAS SPS | No-stretching controls | Lower limbs  3 | 3  15 s  Unreported | 135 s | DS: 1 repetition every 2 seconds SAS + SPS: unreported  DS: reach a greater stretch in each repetition SPS + SAS: to point of mild discomfort |
| Carvalho et al. [78] | Static stretching (unclear if active or passive)  PNF (unreported method) | — | Lower limbs  5 | 3  15 s  Unreported | 225 s | Unreported  PNF: maximum ROM Static stretching: unreported |
| Celik [79] | SAS (longer duration per stretch, fewer sets). SAS (smaller duration per stretch, more sets; the authors termed this "cyclic stretching") | No-stretching controls | Shoulder  1 | SAS: 2 SAS ("cyclic stretching"): 6 (2x3)  SAS: 45 s SAS ("cyclic stretching"): 15 s  15 s | 90 s | Slowly  Maximum ROM while avoiding pain |
| César et al. [80] | SPS | CWI No-stretching controls | Forearm and wrist  1 | 9  30 s (each side)  30 s after each 3 sets | 540 s | Slowly  Until the greatest discomfort was reported by the volunteer |
| Cetin et al. [81] | SAS | No-stretching controls | Lower limbs  6 | 2  20 s  Unreported | 240 s | Unreported  Unreported |
| Chaouachi et al. [82] | DS DS + SAS (less than point of discomfort) DS + SAS (to point of discomfort) SAS (to point of discomfort) SAS (less than point of discomfort) SAS (to point of discomfort) + DS SAS (less than point of discomfort) + DS | No-stretching controls | Lower limbs  DS: 5 DS + SAS: 9 SAS: 4 | DS: 2 DS + SAS: 1 SAS: 2  30 s (each side)  10 s between exercise 0 s between sides | DS: 600 s DS + SAS: 540 s SAS: 480 s | DS: slowly, smoothly and continuously without ballistic or abrupt movements. SAS: unreported  DS: through active ROM SAS: to point of discomfort for the maximal intensity condition and to ~90% of point of discomfort for submaximal condition |
| Chaouachi et al. [83] | DS  SPS | — | Lower limbs  1 | 8  30 s (each side)  20 s | 480 s | DS: 1 repetition every 2 seconds SPS: unreported  DS: maximum ROM SPS: to point of discomfort |
| Chatzopoulos et al. [84] | DS SAS | No-stretching controls | Lower limbs  3 | 1  DS: 15 reps (each side) SAS: 30 s (each side)  10 to 15 s | DS: 90 reps SAS: 180 s | DS: variable, but ~1 repetition every 2 seconds. 5x slowly and 10x as quickly as possible SAS: unreported  DS: maximum ROM SAS: to point of mild discomfort |
| Chatzopoulos et al. [85] | DS SAS | No-stretching controls | Full-body  DS: 8 SAS: 7 | 1  DS: unclear (across 18 m distance) SAS: 30 s (each side)  DS: 10 s SAS: unreported | DS: insufficient information to assess SAS: 420 s | Unreported  DS: unreported SAS: to point of mild discomfort |
| Chatzopoulos et al. [86] | DS (6 reps) DS (12 reps) DS (18 reps) | No-stretching controls | Lower limbs  3 | 1  LV: 6 reps (each side) IV: 12 reps (each side) HV: 18 reps (each side)  Unreported | LV: 36 reps IV: 72 reps HV: 108 reps | 1 repetition every 2 seconds, without bouncing  Maximum ROM |
| Chen et al. [87] | DS | Running Single leg slide curl | Lower limbs  1 | 1  12 reps (each side)  Unreported | 24 reps | The first 6x slowly, the ensuing 6x as quickly and powerfully as possible without bouncing  Maximum ROM |
| Chen et al. [88] | SAS (15 min) SAS (30 min) | No-stretching controls | Lower limbs and trunk  4 | Unclear. "Repeated until the participants were instructed to stop stretching"  15 s  7.5 s | Insufficient information to assess | Unreported  Maximum ROM |
| Chinnavan et al. [89] | SAS + DS + PNF (unclear modality) | Pilates | Lower limbs  Unreported | 3 to 5  30 s (each side)  15 s | Insufficient information to assess | Unreported  Feeling a stretch |
| Christensen et al. [91] | SAS | Mini-band WU Medicine-ball WU Light jogging WU | Full-body  9 | 1  15 s (each side)  Unreported | 270 s | Unreported  To point of light discomfort |
| Christensen, Nordstrom [90] | PNF (CR) | Dynamic WU No-stretching controls | Lower limbs  4 | 1  ~30 s (each side)  Unreported | 240 s | Unreported  Unreported |
| Chtourou et al. [92] | DS SAS | No-stretching controls | Lower limbs  3 | 3  20 s  7 to 8 s | 180 s | DS: 5x slowly, then 10x as quickly as possible without bouncing SAS: unreported  DS: unreported SAS: to point of mild discomfort |
| Church et al. [93] | PNF (CRAC) SAS | No-stretching controls | Lower limbs  PNF (CRAC): unreported SAS: 6 | PNF (CRAC): 3 SAS: unreported  PNF (CRAC): ~60 s SAS: unreported  Unreported | Insufficient information to assess | Unreported  PNF (CRAC): until feeling of tightness SAS: unreported |
| Colak [94] | DS | No-stretching controls | Lower limbs  6 | 1  15 reps  15 s | 90 reps | 5 reps slowly, then 10 reps quickly  Unreported |
| Coons et al. [95] | DS  SAS | — | Lower limbs  1 | DS: 4 SAS: 2  DS: 15 reps (each side) SAS: 30 s (each side)  20 s | DS: 120 reps SAS: 120 s | DS: 1 repetition every 2 seconds; 5x slowly, then 10x quickly SAS: slowly  DS: unreported SAS: to point of mild discomfort |
| Correia et al. [96] | PNF (RS) | No-stretching controls | Full-body  3 | Unreported  Unreported  Unreported | Insufficient information to assess | Unreported  Unreported |
| Cwirlej-Sozanska et al. [97] | SAS | — | Lower limbs  3 | 1  60 s (two of the stretches were performed unilaterally)  Unreported | 300 s | Unreported  Unreported |
| da Silva et al. [98] | SAS | — | Lower limbs  4 | 8 (quadriceps and hamstrings) + 7 (adductors and triceps surae)  20 s  20 s | 1200 s | Unreported  Unreported |
| Dalamitros et al. [99] | DS | No-stretching controls | Full-body  7 | 1  10 s  10 s | 70 s | Unreported  Unreported |
| Dallas et al. [100] | PNF (assumed CR due to description)  SAS SAS + vibration | — | Lower limbs  3 | 1  15 s  15 s | 45 s | PNF (CR): unclear SAS: slowly  SAS + vibration: slowly  To point of discomfort |
| Dalrymple et al. [101] | DS SAS | No-stretching controls | Lower limbs  4 | DS: 2 SAS: 3  DS: unclear (across 18 m distance) SAS: 15 s  20 s | 180 s | Unreported  Unreported |
| Damasceno et al. [102] | SAS + SPS | No-stretching controls | Lower limbs  SAS: 5 + SPS: 2 | 3  30 s  Unreported | 630 s | Unreported  8-9 score on the Borg CR10 scale |
| Darcadia et al. [103] | SAS | No-stretching controls | Lower limbs  4 | 8 for two exercises + 7 for two other exercises  20 s  20 s | 600 s | Unreported  Unreported |
| Dawson et al. [104] | SPS | Hot shower alternated with CWI No-stretching controls Pool walking | Lower limbs and trunk  Unreported | 2 to 3  30 s  Unreported | Insufficient information to assess | Unreported  Unreported |
| de Almeida Leme et al. [105] | Stretching (unclear modality) | No-stretching controls | Unreported  Unreported | Unreported  Unreported  Unreported | Insufficient information to assess | Unreported  Unreported |
| de Castro et al. [106] | SAS | No-stretching controls | Lower limbs  4 | 3  30 s (each side)  0 s | 720 s | Unreported  Maximum ROM while avoiding pain |
| De Oliveira Júnior et al. [107] | PNF (CR) (35 s)  PNF (CR) (65 s) | — | Lower limbs  1 | 4  LV: 35 s (each side) HV: 65 s (each side)  10 s | LV: 280 s HV: 520 s | Unreported  To point of discomfort |
| de Oliveira, Pinto Lopes Rama [108] | SAS | No-stretching controls | Lower limbs  5 | 1  30 s (each side)  5 s | 300 s | Unreported  Unreported |
| de Rezende et al. [109] | SAS | Cycloergometer Leg press No-stretching controls Specific vertical jumping | Lower limbs  Unclear: possibly 8 | 4  30 s  20 s between exercises 30 s between sets | 960 s | Unreported  Between 3 and 4 (annoying, uncomfortable, troublesome pain) on a VAS from 0 to 10 |
| Di Cagno et al. [110] | SAS | No-stretching controls | Lower limbs  4 | 3  > 30 s (each side; one exercise was bilateral)  Unreported | 630 s | Unreported  To point of light discomfort |
| Di Cagno et al. [111] | DS  SPS | — | Upper limbs  DS: 6 SPS: 3 | DS: 10 SPS: 2  DS: 6 s SPS: 3 x 30 s  DS: unreported SPS: 10 s between reps, 15 s between sets | DS: 360 s SPS: 540 s | DS: slow to moderate velocity SPS: unreported  DS: maximum ROM SPS: maximum ROM while avoiding pain |
| Donti et al. [112] | SAS (continuous)  SAS (intermittent) | — | Hip extensors  1 | Continuous SAS: 1 Intermittent SAS: 6  Continuous SAS: 180 s Intermittent SAS: 30 s  Continuous SAS: 0 s Intermittent SAS: 30 s | 180 s | Unreported  To point of discomfort: 80 to 90 on a 100-point VAS |
| Donti et al. [113] | SAS | — | Calf  1 | 1  60 s  Not applicable | 60 s | Slowly  To point of discomfort: 80 to 90 on a 100-point VAS |
| Donti et al. [114] | SAS (continuous)  SAS (intermittent) | — | Hip extensors  1 | Continuous SPS: 1 Intermittent SPS: 3  Continuous SPS: 90 s Intermittent SPS: 30 s  Continuous SAS: 0 s Intermittent SAS: 30 s | 90 s | Unreported  To point of discomfort: 90 to 100 on a 100-point VAS |
| Drews, Goltz [115] | SPS (longitudinal) SPS (transversal) | No-stretching controls | Hip adductors  Unclear: possibly 9 | 1  30 s  15 s | 270 s | Unreported  Unreported |
| Ebadi, Cetin [116] | SAS (15 s) SAS (30 s) SAS (45 s) | No-stretching controls | Lower limbs  17 | 1  LV: 15 s IV: 30 s HV: 45 s  5 s | LV: 255 s IV: 510 s HV: 765 s | Unreported  Unreported |
| Egan et al. [7] | SPS + SAS | — | Quadriceps  4 | 4  30 s  20 s | 480 s | Unreported  To point of mild discomfort |
| Eken, Bayer [117] | PNF (CRAntC)  PNF (CRAntC) (half the volume) + massage | Massage PNF (CRAntC) (half the volume) + massage Running | Full-body  7 | 1  20 s (each side)  Unreported | 280 s | Unreported  To a mild stretch or restriction point |
| Espi-Lopez et al. [118] | PNF (CR) | Manual therapy | Lower limbs  7 | 4  35 s (each side)  10 s | 1960 s | Unreported  Feeling a stretch |
| Evetovich et al. [119] | SAS + SPS. | — | Quadriceps  SAS: 1 + SPS: 3 | 4  30 s  20 s | 480 s | Unreported  To point of mild discomfort |
| Faigenbaum et al. [120] | SAS | Dynamic WU SAS followed by dynamic WU | Full-body  5 | 2  30 s  5 s | 300 s | Slowly  To point of mild discomfort |
| Faigenbaum et al. [121] | SAS | Dynamic WU | Full-body  5 | 3  20 s  Unreported | 300 s | Unreported  To point of mild discomfort |
| Faigenbaum et al. [122] | SAS | Dynamic exercises Dynamic exercises with weighted vest (2% body mass) Dynamic exercises with weighted vest (6% body mass) | Full-body  5 | 2  30 s  5 s | 300 s | Slowly  To point of mild discomfort |
| Famisis [123] | SAS | Calisthenics with full ROM Calisthenics without full ROM | Full-body  5 | 2  10 s  10 s | 100 s | Slowly  Feeling a stretch, but no pain |
| Farshidi et al. [124] | PNF (CRAC) SPS | No-stretching controls | Lower limbs  1 | 5  30 s (each side)  30 s | 300 s | Unreported  To point of mild discomfort |
| Fattahi-Bafghi, Amiri-Khorasani [125] | DS SAS | No-stretching controls | Lower limbs  6 | 1  DS: 5 reps SAS: 30 s  Unreported | DS: 30 reps SAS: 180 s | DS: 1x slow, 1x moderate, 1x as fast-as-possible SAS: slowly  Maximum ROM |
| Favero et al. [126] | SAS | No-stretching controls | Lower limbs  4 | 2  45 s (each side)  Unreported | 720 s | Unreported  To point of discomfort |
| Feitosa Junior et al. [127] | SAS | No-stretching controls | Wrists  4 | 2  120 s  Unreported | 960 s | Unreported  To point of light discomfort |
| Fernandes et al. [128] | SPS  SPS + vibration | — | Quadriceps  1 | 3  30 s  30 s | 90 s | Unreported  To point of maximum discomfort |
| Ferreira et al. [129] | DS  SAS | — | Lower limbs  4 | 1  DS: 10 reps (each side) SAS: 10 s (each side)  Unreported | DS: 80 reps SAS: 80 s | Unreported  To point of discomfort |
| Ferri-Caruana et al. [130] | PNF (with concentric, eccentric, and isometric phases) [termed dynamic ROM by the authors]  SPS | — | Lower limbs  PNF: 8 SPS: 4 | PNF: 5 SPS: 1 for the 3 unilateral exercises, 2 for the bilateral exercise  PNF: 15 s (each side) SPS: 90 s (each side; 1 stretch was bilateral)  Unreported | PNF: 1200 s SPS: 720 s | PNF: very slowly SPS: unreported  PNF: to 90% of point of discomfort SPS: unreported |
| Fletcher, Anness [131] | DS (while moving) [termed active DS by the authors] DS (while stationary) [termed static DS combined with active DS by the authors]  SPS + DS [termed SPS combined with active DS by the authors] | — | Lower limbs  DS (stationary): 5 DS (while moving): 5 DS (while moving) + SPS: 5 + 5 | DS (stationary): 2 DS (while moving): 2 DS (while moving) + SPS: 2 + 3  DS (stationary): 8 reps (each side) DS (while moving): unclear (over 20 m distance) DS (while moving) + SPS: unclear (over 20 m distance) + 22 s  DS (stationary): 10 s DS (while moving): 20 m walked-back recovery DS (while moving) + SPS: 10 s | DS (stationary): 160 reps DS (while moving): unclear; 10x over a 20-m distance DS (while moving) + SPS: unclear; 10x over a 20-m distance + 330 s | DS (stationary): controlled movement DS (while moving): controlled movement DS (while moving) + SPS: slowly  DS (stationary): through active ROM DS (while moving): through active ROM SPS: to point of mild discomfort |
| Fletcher, Jones [132] | DS (while moving) [termed active DS by the authors] DS (stationary) [termed static DS by the authors]  SAS SPS | — | Lower limbs  DS (stationary): 5 DS (while moving): 5 SAS: 7 SPS: 7 | 1  DS (while moving): 20 reps (each side) DS (stationary): 20 reps (each side)  SAS: 20 s SPS: 20 s  DS (while moving): walk-back recovery DS (stationary): unreported SAS: unreported SPS: unreported | DS (while moving): 200 s DS (stationary): 200 s SAS: 140 s SPS: 140 s | DS (while moving): controlled movement DS (stationary): controlled movement SAS: unreported SPS: slowly  DS (while moving): through active ROM DS (stationary): through active ROM SAS: maximum ROM SPS: to point of mild discomfort |
| Fletcher, Monte-Colombo [133]  Fletcher, Monte-Colombo [134] | DS SPS | No-stretching controls | Lower limbs  DS: 6 SPS: 8 | DS: 2 SPS: 2 sets for 4 of the stretches. 1 set for the other 4 stretches  DS: 12 reps SPS: 15 s  DS: Unreported SPS: 5 s | DS: 144 reps SPS: 360 s | DS: in a controlled manner SPS: unreported  DS: through active ROM SPS: to point of mild discomfort |
| Forte et al. [135] | DS SAS | No-stretching controls | Lower limbs  Unreported | Unreported  15 s  10 s for bilateral exercises, 0 s for unilateral exercises | Insufficient information to assess | DS: unreported SAS: slowly  DS: moderate to high intensity SAS: maximum ROM while avoiding pain |
| Frantz, Ruiz [136] | SAS | Dynamic WU No-stretching controls | Lower limbs  22 | 1  Unreported  Unreported | Insufficient information to assess | Unreported  Unreported |
| Fredericson et al. [137] | SAS (for iliotibial band, with arms at side)  SAS (for iliotibial band, with arms extending overhead) SAS (for iliotibial band, with arms reaching diagonally downward) | — | Lower limbs  1 | 3  30 s  30 s | Insufficient information to assess | Slowly  Feeling a stretch |
| Frikha et al. [138] | BS DS SAS | No-stretching controls | Lower limbs  6 | BS: unreported DS: unreported SAS: 2  BS: unreported DS: unreported SAS: 30 s  BS: 15 s DS: 15 s SAS: 30 s | BS: insufficient information to assess DS: insufficient information to assess SAS: 360 s | BS: 1 repetition per second DS: 1 repetition every 2 seconds SAS: unreported  BS: to extreme ROM DS: unreported SAS: to ~90% of point of discomfort |
| Funk et al. [139] | PNF (CR)  SPS | — | Lower limbs  1 | Unreported  PNF (CR): 30 s SPS: 15 s  PNF (CR): unreported SPS: 30 s | Insufficient information to assess | Unreported  PNF (CR): through pain-free ROM SPS: feeling a stretch |
| Funk et al. [140] | SAS | Moist heat pack application (20 min) | Hamstrings  1 | 3  30 s  Unreported | 90 s | Unreported  Feeling a stretch |
| Gabbe et al. [141] | SAS | Eccentric strength training | Lower limbs and trunk  5 | 3  30 s (each side) except lumbar spine rotation (15 s)  Unreported | 270 s | Unreported  Unreported |
| Galazoulas [142] | SAS (3 sets)  SAS (30 sets) | — | Lower limbs  2 | LV: 3 HV: 30  5 s  10 s | LV: 30 s HV: 300 s | Unreported  Feeling a stretch |
| Galazoulas [143] | DS  SAS | — | Lower limbs  5 | 2  10 s (each side)  DS: unreported SAS: 10 s | 200 s | DS: unreported SAS: slowly  DS: maximum ROM SAS: maximum ROM while avoiding pain |
| Galetin et al. [144] | SAS (30 s)  SAS (60 s) SAS (90 s) | — | Lower limbs  6 | 1 for 4 bilateral stretches; 2 for 2 unilateral stretches  LV: 30 s (each side) IV: 60 s (each side) HV: 90 s (each side)  Unreported | LV: 390 s IV: 780 s HV: 1170 s | Unreported  Unreported |
| Gao et al. [145] | BS  SAS | No-stretching controls | Lower limbs  3 | 3  30 s (each side; 2 stretches were unilateral)  30 s | 450 s | BS: 1 repetition per second SAS: unreported  To point of light discomfort |
| Gelen [146] | DS SAS SAS + DS | No-stretching controls | Lower limbs  DS: 12 DS + SAS: 17 SAS: 5 | 2  DS: unclear (across 15 m distance) SAS: 20 s  10 s | DS: insufficient information to assess DS + SAS: insufficient information to assess SAS: 200 s | DS: unreported SAS: slowly  DS: unreported SAS: to point of mild discomfort |
| Gelen et al. [147] | SAS | No-stretching controls | Lower limbs  5 | 3  20 s  15 s | 300 s | Unreported  To point of mild discomfort |
| Gelen et al. [148] | SAS + SPS | Dynamic exercises HV upper extremity plyometrics No-stretching controls | Upper limbs  7 | 3  10 s  30 s | 210 s | Slowly  Intense sensitivity level |
| Gergley [149]  Gergley [150] | SPS | Dynamic WU | Full-body  12 | 3  10 s (each side; only two stretches were bilateral)  Unreported | 660 s | Unreported  Unreported |
| Ghasemi et al. [151] | SPS | No-stretching controls | Plantar flexors  1 | 4  20 s  10 s | 80 s | 20° per second  Unclear: to 20° of ankle dorsiflexion |
| Gonçalves et al. [152] | PNF (CR) [termed "DS" by the researchers]  SPS | — | Lower limbs  4 | PNF (CR): 4 SPS: unreported  PNF (CR): >15 s SPS: unreported  Unreported | PNF (CR): 240 s SPS: insufficient information to assess | Unreported  Maximum ROM |
| Gürses, Akgül [153] | DS DS + SAS SAS | No-stretching controls | Lower limbs  DS: 7 DS + SAS: 4 + 5 SAS: 5 | DS: 3 DS + SAS: 3+1 SAS: 2  DS: 30 s SAS: 20 s (1st set), 30 s (2nd set)  Unreported | DS: 630 s DS + SAS: 485 s SAS: 250 s | DS: moderate speed SAS: unreported  DS: through active ROM SAS: unreported |
| Haag et al. [154] | SAS + SPS | No-stretching controls | Upper limbs  6 | 1  30 s (only throwing shoulder)  10 s | 180 s | Unreported  To point of mild discomfort |
| Haddad et al. [155] | DS SAS | No-stretching controls | Lower limbs  5 | 2  30 s (each side)  15 s between sides and 30 s between exercises | 600 s | Unreported  Unreported |
| Haddad et al. [156] | DS SAS | No-stretching controls | Lower limbs  2 | DS: 5 SAS: 2  DS: 30 s SAS: 75 s  Unreported | 300 s | DS: not ballistic SAS: unreported  DS: maximum ROM while avoiding pain SAS: to point of discomfort |
| Han et al. [157] | DS SAS SAS +DS | No-stretching controls | Lower limbs  DS: 6 DS + SAS: 11 SAS: 5 | DS: 2 DS + SAS: 2+1 SAS: 1  DS: unclear (across 20 m distance) DS + SAS: unclear (across 20-m distance) + 30 s SAS: 30 s  DS: across wall-back recovery SAS: 15 s | DS: insufficient information to assess DS + SAS: insufficient information to assess SAS: 150 s | Unreported  DS: through active ROM SAS: to point of mild discomfort |
| Heisey, Kingsley [158] | SAS | No-stretching controls | Lower limbs  3 | 2  30 s (each side)  10 s | 360 s | Unreported  To point of discomfort |
| Herman, Smith [159] | DS  SAS | — | Full-body  DS: 11 SAS: 8 | 1  DS: 10 reps SAS: 30 s  Unreported | DS: 110 reps SAS: 240 s | DS: moderate pace SAS: unreported  Unreported |
| Higuchi et al. [160] | SAS | — | Upper limbs  1 | 5  30 s  10 s | 150 s | Unreported  Maximum ROM while avoiding pain |
| Holt, Lambourne [161]  *Erratum*: Holt, Lambourne [326] | DS SPS | Dynamic WU No-stretching controls | Lower limbs  DS: 8 SPS: 5 | DS: 10 SPS: 3  DS: Unreported SPS: 5 s  DS: Unreported SPS: 1 s | DS: insufficient information to assess SPS: 75 s | Unreported  DS: unreported SPS: slightly painful yet tolerable muscle discomfort |
| Hough et al. [162] | DS SPS | No-stretching controls | Lower limbs  5 | 1  DS: 15 reps SPS: 30 s (each side)  10 to 15 s | DS: 75 reps SPS: 300 s | DS: 5x slowly and 10x as quickly as possible without bouncing SPS: unreported  DS: unreported SPS: to point of mild discomfort, but no pain |
| Hsu et al. [163] | DS + SAS | DS followed by foam rolling DS followed by vibration foam | Lower limbs  DS: 8 SAS: 5 | DS: 1 SAS: 2  DS: 10 reps (each side) SAS: 30 s  10 s | 80 reps + 300 s | DS: unreported SAS: slowly  DS: through active ROM SAS: unreported |
| Huang et al. [164] | DS + soleus stretching DS without soleus stretching | No-stretching controls | Lower limbs  DS (with soleus stretch): 4 DS (without soleus stretch): 3 | 3  30 s (each side)  Unreported | Soleus group: 720 s Non-soleus group: 540 s | Unreported  Maximum ROM |
| Huang, Zhu [165] | PNF (CR) | No-stretching controls | Lower limbs  Unreported | 2 to 3  ~60 s  Unreported | Insufficient information to assess | Slowly  Maximum ROM |
| Ide et al. [166] | BS SPS | No-stretching controls Parallel squat | Lower limbs  4 | 3  30 s (each side; 1 stretch was bilateral)  15 s | 630 s | BS: 1 repetition per second SPS: unreported  BS: unreported SPS: maximum ROM |
| Ishak et al. [167]  Ishak et al. [168] | DS (1 set)  DS (2 sets) DS (3 sets) | — | Lower limbs  5 | LV: 1 IV: 2 HV: 3  20 reps  30 s | LV: 100 reps IV: 200 reps HV: 300 reps | Gently  Unreported |
| Ishak et al. [169] | BS (1 set)  BS (2 sets) BS (3 sets) | — | Lower limbs  5 | LV: 1 IV: 2 HV: 3  20 reps  30 s | LV: 100 reps IV: 200 reps HV: 300 reps | 1 repetition per second  Unreported |
| Jang et al. [170] | DS SAS | Resistance DS (weighted vests and dumbbells) | Full-body  DS: 9 SAS: 7 | 1  DS: unclear (across 40 m distance) SAS: 30 s (each side)  DS: unreported SAS: 5 s | DS: insufficient information to assess SAS: 420 s | Unreported  DS: light intensity SAS: unreported |
| Jemni et al. [171] | SAS  SAS + vibration | — | Lower limbs  2 | 1  45 s  10 s | 90 s | Unreported  Unreported |
| Jing, Yang [172] | DS  SAS | — | Lower limbs  4 | 5  30 s  30 s | 600 s | DS: unreported SAS: gradually  DS: unreported SAS: maximum ROM |
| Johnson et al. [173] | SAS  SAS + vibration | — | Lower limbs  3 | 4  30 s  5 s | 360 s | Unreported  Unreported |
| Jordan et al. [174] | PNF (HR)  SAS | — | Lower limbs  4 | 2  PNF (HR): 46 s (each side) SAS: 30 s (each side)  Unreported | PNF (HR): 736 s SAS: 480 s | Unreported  To point of mild discomfort |
| Junqueira et al. [175] | GAS^®^  SAS | No-stretching controls | GAS^®^: full-body SAS: unreported  GAS^®^: 3 SAS: unreported | 1  GAS^®^: 600 s SAS: 20 s  Unreported | GAS^®^: 1800 s SAS: insufficient information to assess | Unreported  Unreported |
| Kafkas et al. [176] | SAS | Dry land WU In-water WU No-stretching controls | Full-body  10 | 2  30 s (each side)  10 s | 1200 s | Slowly  To point of mild discomfort |
| Karloh et al. [177]  Karloh et al. [178] | Mulligan's stretching [SPS + manual traction]  SPS | — | Lower limbs  1 | 2  30 s (each side)  30 s | 120 s | Unreported  Unreported |
| Kazemi et al. [179] | DS  SAS | No-stretching controls | Upper limbs  5 | DS: 1 SAS: 2  DS: 15 reps SAS: 15 s  DS: Unreported SAS: 15 s | DS: 75 reps SAS: 150 s | DS: high speed SAS: unreported  DS: maximum ROM while avoiding pain Static stretching: to point of discomfort |
| Kilit et al. [180] | DS DS + SPS SPS SPS + DS | No-stretching controls | Lower limbs  DS: 6 DS + SPS: 12 SPS: 6 SPS+ DS: 12 | DS: 2 DS + SPS: 1 SPS: 2 SPS+ DS: 1 SPS: 2  30 s  0 s | 360 s | DS: slowly SPS: unreported  To point of discomfort |
| Konrad et al. [181] | PNF (CR) for quadriceps PNF (CR) for triceps surae | No-stretching controls | Lower limbs  1 | 4  15 s  Unreported | 60 s | Unreported  To point of discomfort |
| Kornberg et al. [182] | SPS on one leg | No stretching on the other leg | Lower limbs and trunk  1 | 1  7 s  Not applicable | 7 s | Unreported  Unreported |
| Krčmár et al. [183] | BS + DS  DS | DS followed by running drills DS followed by half-squat jumps DS followed by drop jumps No-stretching controls | Lower limbs  BS + DS: 4+5 DS: 5 | 1  BS + DS: 30 s on both legs + 15 reps DS: 15 reps  15 s | BS + DS: 75 reps + 240 s DS: 75 reps | BS: bouncing rapidly DS: 5x slowly + 10x quicker but without bouncing  Unreported |
| Kruse et al. [184]  Kruse et al. [185] | DS SAS | Aerobic WU | Lower limbs  DS: 14 SAS: 7 | 1  DS: 30 s SAS: 30 s (each side)  0 s | 420 s | DS: progressed from slow to high velocity SAS: unreported  DS: progressed from slow to high intensity through active ROM SAS: to point of discomfort |
| Kurt [186] | DS SAS | Local vibration Whole body vibration | Lower limbs  DS: 8 SAS: 6 | DS: 2 SAS: 1  DS: 15 s SAS: 20 s (each side)  DS: 30 s SAS: 20 s | 240 s | DS: unreported SAS: slowly  DS: unreported SAS: feeling a stretch |
| Kurt, Firtin [187] | DS SAS | No-stretching controls | Lower limbs and trunk  7 | DS: 2 SAS: 1  DS: 20 s SAS: 20 s (each side; 1 stretch was bilateral)  10 s | DS: 280 s SAS: 260 s | Unreported  Unreported |
| Kurtdere et al. [188] | SAS (10 s)  SAS (20 s) SAS (30 s) | — | Lower limbs  4 | 3  LV: 10 s (each side; 1 stretch was bilateral) IV: 20 s (each side; 1 stretch was bilateral) HV: 30 s (each side; 1 stretch was bilateral)  0 s | LV: 210 s IV: 420 s HV: 630 s | Unreported  To point of mild discomfort |
| Kyranoudis et al. [189] | SAS | SAS followed by foam rolling | Lower limbs  4 | 1  10 s (each side)  0 s between sides 20 s between different muscle groups | 80 s | Unreported  Feeling a stretch |
| Kyranoudis et al. [190] | DS  SAS | — | Lower limbs  6 | 2  10 s (each side)  0 s | 240 s | Unreported  Maximum ROM while avoiding pain |
| Laudner et al. [191] | SPS | — | Upper limbs  1 | 3  30 s  30 s | 90 s | Unreported  Maximum ROM |
| Li et al. [192] | PNF (CR)  SPS | — | Upper limbs  1 | 15  PNF (CR): 21 s SPS: 20 s  5 s | PNF (CR): 315 s SPS: 300 s | Slowly  Maximum ROM |
| Lin et al. [193] | DS | DS followed by vibration foam rolling | Full-body  8 | 2  Unclear (across 18 m distance)  10 s | Insufficient information to assess | Gradually  Maximum ROM |
| Little, Williams [194] | DS SAS | No-stretching controls | Lower limbs  5 | 1  30 s (each side)  20 s | 150 s | DS: 1 repetition per second for unilateral exercises, 1 repetition every 2 seconds for bilateral exercises SAS: unreported  DS: maximum ROM SAS: maximum ROM while avoiding pain |
| Lotfi et al. [195] | Stretching (unclear modality) | No-stretching controls | Unreported  Unreported | Unreported  10 s  Unreported | Insufficient information to assess | Unreported  Unreported |
| Loughran et al. [196] | SAS SAS + DS | No-stretching controls | Lower limbs  DS + SAS: 10 + 5 SAS: 5 | DS + SAS: 2+1 SAS: 1  30 s  Unreported | SAS + DS: 750 s SAS: 150 s | Unreported  DS: unreported SAS: to point before discomfort |
| Lowery et al. [197] | SAS | No-stretching controls | Lower limbs  6 | 3  30 s  30 s | 540 s | Unreported  Unreported |
| Makaruk et al. [198] | SAS | Isometric training No-stretching controls | Lower limbs  4 | 1  30 s  20 s | 120 s | Unreported  Sub-maximal intensity |
| Manzi et al. [199] | GPR  SAS SAS + vibration | — | Lower limbs  GRP: 1 SAS: Unreported | 6  20 s  20 s | GPR: 120 s SAS: insufficient information to assess | Unreported  Unreported |
| Mariscal et al. [200] | BS  SPS | — | Lower limbs  4 | 2  BS: 8 reps (each side) SPS: 10 s (each side)  Unreported | BS: 128 reps SPS: 80 s | Unreported  Unreported |
| Martin et al. [201] | DS  SAS | — | Lower limbs  Unreported | DS: 1 SAS: 2  DS: 6 to 10 reps SAS: 10 to 15 s  DS: 2 to 5 s SAS: 5 to 10 s | Insufficient information to assess | Unreported  To point of mild discomfort (1-3 intensity) |
| Martinez-Chicote et al. [202] | BS PNF (insufficient information to assess specific method) SAS | No-stretching controls | Lower limbs  5 | 1  30 s  Unreported | 150 s | Unreported  Maximum ROM while avoiding pain |
| Mascarin et al. [203] | SAS | Dynamic WU SAS followed by dynamic WU | Upper limbs  5 | 3  30 s  15 s | 450 s | Unreported  To point of mild discomfort |
| McNeal, Sands [204] | SPS | No-stretching controls | Lower limbs  3 | 1  30 s  Unreported | 90 s | Unreported  To point of mild discomfort |
| McNeal et al. [205] | SPS  SPS + vibration | — | Lower limbs  2 | 4  10 s (each side)  5 s | 160 s | Unreported  Maximum ROM while avoiding pain |
| Meerits et al. [206] | DS  SAS | — | Hamstrings  1 | 9 (3x3)  20 s (each side)  30 s | 180 s | DS: at a frequency of 60 bpm SAS: unreported  DS: unreported SAS: 75% of maximal intensity (Borg's Scale of Perceived Exertion) |
| Melocchi et al. [207] | DS SAS | No-stretching controls | Full-body  DS: 7 SAS: 10 | 1  DS: 3 to 10 reps SAS: 20 s  0 s | DS: 21 reps SAS: 200 s | Unreported  Unreported |
| Mendez-Sanchez et al. [12] | SPS  SPS + Neural sliding [variation of DS] | — | Lower limbs  DS + DS: 2 SPS: 1 | 1  DS + SPS: 120 s + 300 s SPS: 300 s  Not applicable | SPS: 300 s SPS + DS: 420 s | Unreported  Unreported |
| Merrigan et al. [208] | SPS  SPS + vibration | — | Full-body  9 | 1  30 s (each side; 3 stretches were bilateral)  30 s | 450 s | Unreported  Unreported |
| Mikolajec et al. [209] | SAS | No-stretching controls Strength training | Lower limbs  4 | 3  10 s  Unreported | 120 s | Unreported  80-90% of maximum ROM |
| Miladi et al. [210] | DS | Low intensity cycling No-stretching controls | Lower limbs  4 | 1  30 s  30 s | 120 s | Gradually  To point of discomfort |
| Mojock et al. [211] | SAS | No-stretching controls | Lower limbs  5 | 4  Unreported  Unreported | Insufficient information to assess | Unreported  To point of maximal tension |
| Molacek et al. [212] | PNF (CR) (2 sets) PNF (CR) (5 sets) SPS (2 sets) SPS (5 sets) | No-stretching controls | Upper limbs  2 | LV PNF (CR): 2 HV PNF (CR): 5 LV SPS: 2 HV SPS: 5  PNF (CR): 25 s SPS: 20 s  60 s | LV PNF (CR): 100 s HV PNF (CR): 250 s LV SPS: 80 s HV SPS: 200 s | Unreported  To point of moderate tension |
| Montalvo, Dorgo [213] | DS DS + SAS  SAS SAS + DS | — | Full-body  DS: 15 DS + SAS: 30 SAS: 15 | Unreported  Unreported  Unreported | Insufficient information to assess | Unreported  Unreported |
| Moore, Hutton [214] | PNF (CR) PNF (CRAC)  SPS | — | Hip extensors  1 | 3  PNF (both methods): 14 s SPS: 9 s  Unreported | PNF (CR): 42 s PNF (CRAC): 42 s SPS: 27 s | Unreported  Unreported |
| Mor et al. [215] | DS  SAS | — | Lower limbs  18 | 1  DS: 20 s SAS: 10 to 15 s  10 s | DS: 360 s SAS: 180 s | Unreported  DS: increasing intensity SAS: to pain threshold |
| Moran et al. [216] | DS SAS | No-stretching controls | Full-body  9 | 3  DS: 10 reps SAS: 30 s  20 s | DS: 270 reps SAS: 810 s | DS: slowly on the 1st set, quickly but without bouncing on subsequent sets SAS: unreported  DS: maximum ROM SAS: to point of mild discomfort |
| Moran et al. [217] | SAS | Dynamic WU | Full-body  9 | 2  30 s  5 s | 540 s | Unreported  To point of mild discomfort |
| Moreno-Perez et al. [218] | DS | Heavy load leg press | Lower limbs and trunk  6 | 3  30 s  15 s | 540 s | In a controlled manner  From low to high intensity |
| Needham et al. [219] | DS  SAS | DS followed by 8 front squats w/20% body mass | Lower limbs  6 | 2  DS: unclear (distance of 20 yards) SAS: 15 s (each side)  DS: unclear (20 yards walking back to the start position) SAS: 0 s | DS: insufficient information to assess SAS: 360 s | DS: unreported SAS: slowly  DS: progressed from moderate to high intensity SAS: to point of mild discomfort |
| Nelson et al. [220] | SPS (both limbs) SPS (forward limb stretched) SPS (rear limb stretched) | No-stretching controls | Lower limbs  3 | 4  30 s  10 to 20 s | 360 s | Unreported  To point of discomfort |
| Nobre et al. [221] | PNF (dynamic reversion) | No-stretching controls | Upper limbs  2 | 2  First session: 15 reps. On every three sessions there were five reps added to the total amount.  30 s | 60 reps (first week) to 160 reps (last week) | Slowly  Unreported |
| Notarnicola et al. [222] | First month: SPS Second month: SAS  Third month: Mezieres stretching Fourth month: PNF (CR) Fifth month: DS | No-stretching controls | Lower limbs  DS: unreported Mezieres stretching: 2 PNF (CR): unreported SAS: unreported SPS: unreported | DS: unreported Mezieres stretching: 1 PNF (CR): unreported SAS: 2 SPS: 2  DS: unreported Mezieres stretching: 300 s PNF (CR): 50 to 55 s (each side) SAS: 25 to 30 s (each side) SPS: 25 to 30 s (each side)  Unreported | Insufficient information to assess | DS: slowly Mezieres stretching: unreported PNF (CR): slowly SAS: unreported SPS: unreported  DS: maximum ROM Mezieres stretching: unreported PNF (CR): maximum ROM SAS: unreported SPS: unreported |
| Nuri et al. [223] | SPS | Active WU Passive warm–up (Ultrasound) | Lower limbs  1 | 3  30 s  15 s | 90 s | Unreported  Unreported |
| O'Sullivan et al. [224] | DS  SAS | — | Lower limbs  1 | 3  30 s  Unreported | 90 s | Unreported  Feeling a stretch |
| Ohshita, Mitsuzono [10] | SAS SPS | No-stretching controls | Lower limbs  2 | Unreported  Unreported  Unreported | Insufficient information to assess | Unreported  SAS: unreported SPS: feeling muscle tension |
| Olivares-Arancibia et al. [225] | SPS SPS + vibration | No-stretching controls | Hamstrings  1 | 6  30 s (each side)  0 s | 360 s | Unreported  Unreported |
| Oliveira et al. [226] | BS PNF (HR) SAS SPS | No-stretching controls | Lower limbs  4 | 3  BS: 60 s PNF (HR): 30 s SAS: 30 s SPS: 30 s  BS: unreported PNF (HR): 30 s SAS: 30 s SPS: 30 s | BS: 720 s PNF (HR): 360 s SAS: 360 s SPS: 360 s | BS: 1 repetition per second PNF (HR): unreported SAS: unreported SPS: unreported  BS: unreported PNF (HR): to point of maximum discomfort (level 100 of 150 on the SIS) SAS: unreported SPS: unreported |
| Oña Tacan et al. [227] | SAS (termed maximal contraction method by the authors)  SPS (termed dynamic passive insistence by the authors) | — | Lower limbs and trunk  1 | 20 (4x5)  10 s  SAS: 10 s SPS: 20 s | 200 s | Unreported  SAS: maximum ROM SPS: surpass maximum ROM by 3-4° |
| Oskouei et al. [228] | SAS (hamstrings) SAS (quadriceps) SAS (quadriceps + hamstrings) | No-stretching controls | Lower limbs  SAS (hamstrings): 1 SAS (quadriceps): 1 SAS (quadriceps + hamstrings): 2 | 3  30 s  30 s | Single muscle group protocols: 90 s Both muscle groups protocols: 180 s | Unreported  To point of mild discomfort |
| Osternig et al. [229] | PNF (agonist CR) PNF (CR)  SPS | — | Lower limbs  1 | 1  PNF (Agonist CR): 5 reps PNF (CR): 5 reps SPS: 80 s  Unreported | PNF (agonist CR): 5 reps PNF (CR): 5 reps SPS: 80 s | Slowly  To point of restriction |
| Oyama et al. [9] | SAS (horizontal cross-arm stretch)  SAS (sleeper stretch at 90°) SAS (sleeper stretch at 45°) | — | Upper limbs  1 | 3  30 s  30 s | 90 s | Unreported  Feeling a stretch |
| Pagaduan et al. [230] | DS  DS + SPS SPS | No WU SPS followed by WU SPS followed by WU followed by DS WU | Lower limbs  7 | 2  20 s  10 s | DS: 280 s DS + SPS: 560 s SPS: 280 s | Unreported  DS: unreported SPS: to point of discomfort |
| Panidi et al. [231] | SAS on one leg | No stretching on contralateral limb | Plantar flexors  6 | 2  Weeks 0-3: 45 s Weeks 3-6: 60 s Weeks 6-12: 75 s  0 s | Weeks 0-3: 540 s Weeks 3-6: 720 s Weeks 6-12: 900 s | Unreported  Near the point of discomfort, 8–9 in a 0-10 scale |
| Papadimitriou et al. [232] | PNF (CR) | No-stretching controls | Lower limbs  6 | 1  60 s  0 s | 360 s | Unreported  Maximum ROM while avoiding pain |
| Papia et al. [233] | SPS on one leg | No stretching on contralateral limb | Quadriceps  1 | 1  90 s  Not applicable | 90 s | Unreported  To point of discomfort: 90 to 100 on a 100-point VAS |
| Pellegrini et al. [234] | SPS + SAS | No-stretching controls | Upper limbs  6 | 1  Unreported  Unreported | Insufficient information to assess | Unreported  Unreported |
| Penichet-Tomas et al. [235] | SAS | Foam rolling | Lower limbs and trunk  5 | 3  30 s  5 s | 450 s | Unreported  Feeling a stretch, but no pain |
| Pojskic et al. [236] | DS | No-stretching controls Prolonged intermittent low-intensity isometric exercise Prolonged intermittent low-intensity isometric exercise with additional external load ~30% body weight | Unreported  7 | 2  20 s  10 s | 280 s | Unreported  Unreported |
| Polat et al. [237] | BS | No-stretching controls | Full-body  13 | 1  Unreported  0 s | Insufficient information to assess | Unreported  Unreported |
| Pooley et al. [238] | SAS | No-stretching controls | Lower limbs  7 | 2  15 s  Unreported | 210 s | Unreported  Unreported |
| Pooley et al. [239] | SAS | Active recovery (cycle ergometer) CWI | Lower limbs and trunk  7 | 2  15 s  Unreported | 210 s | Unreported  Unreported |
| Popelka, Pivovarniček [240]  Popelka et al. [241] | DS  Static stretching (unclear if active or passive) | — | Unreported  Unreported | 2  DS: 8 to 10 reps SAS: 10 to 15 s  DS: 2 to 5 s SAS: 5 to 10 s | Insufficient information to assess | DS: 8 reps in 5 seconds SAS: unreported  1–3 with slight pain |
| Popelka, Pivovarniček [242] | DS | Foam rolling | Unreported  Unreported | 1  10 to 12 reps  5 to 8 s | Insufficient information to assess | Unreported  1–3 with slight pain |
| Portilla-Dorado et al. [243] | PNF (CR) | Foam rolling No-stretching controls | Lower limbs  8 (rotating, so only 3 stretches per day) | 2  36 s  20 s | 216 s | Unreported  Unreported |
| Racil et al. [244] | DS | DS + plyometrics Plyometrics No WU group | Lower limbs  7 | 3  5 s (each side)  30 s | 105 s | Unreported  Unreported |
| Reis et al. [245] | PNF (CR) SPS | No-stretching controls | Quadriceps  1 | PNF: 6 (2x3) SPS: 2  30 s  10 s | PNF (CR): 180 s SPS: 60 s | Unreported  PNF (CR): maximum ROM SPS: to point of first reported pain |
| Reuther et al. [246] | SPS | No-stretching controls | Upper limbs  1 | 3  30 s  30 s | 90 s | Unreported  Feeling a stretch |
| Robey et al. [247] | SAS | CWI Hot water immersion No-stretching controls | Lower limbs  8 | 2  30 s  Unreported | 480 s | Unreported  Unreported |
| Rodriguez-Marroyo et al. [248] | SAS | Running exercises | Lower limbs  5 | Weeks 1-2: 2 Weeks 3-4: 4 Weeks 5-6: 6  15 s  15 s | Weeks 1-2: 150 s Weeks 3-4: 300 s Weeks 5-6: 450 s | Unreported  To point of discomfort but not pain |
| Rogan et al. [249] | DS | — | Gluteus maximus  1 | 1  30 s (each side)  10 s | 60 s | 100 beats per minute  Unreported |
| Romero-Franco et al. [250] | DS SAS | No-stretching controls | Lower limbs  3 | 1  DS: 20 reps (each side) SAS: 20 s (each side)  10 s | DS: 120 reps SAS: 120 s | DS: 1 repetition per second SAS: unreported  Low-intensity, less than point of discomfort |
| Sagiroglu et al. [251] | SAS | No-stretching controls Self-myofascial release | Lower limbs  4 | 2  30 s (each side)  10 s between sides 30 s between exercises | 480 s | Unreported  To point just before discomfort |
| Sampaio-Jorge et al. [252] | SPS | No-stretching controls | Lower limbs  3 | 1  60 s (each side; 1 stretch was bilateral)  Unreported | 300 s | Unreported  Unreported |
| Sánchez-Sánchez et al. [253] | DS SAS | No-stretching controls | Lower limbs  DS: 10 SAS: 6 | DS: unreported SAS: 2  DS: unreported SAS: 15 s  DS: unreported SAS: 5 s | DS: insufficient information to assess SAS: 180 s | DS: unreported SAS: slowly  DS: progressive intensity SAS: to point of discomfort |
| Sands et al. [254] | SAS  Static stretching + vibration | — | Lower limbs  2 | 1  40 s  Unreported | 80 s | Unreported  Unreported |
| Sands et al. [255] | SAS  Static stretching + vibration | — | Lower limbs  2 | 4  10 s (each side)  5 s | 160 s | Unreported  To point of discomfort |
| Satkunskiene et al. [13] | Passive neurodynamic nerve gliding [a form of SPS] | Foam rolling | Lower limbs  1 | 6  45 s  15 s | 270 s | Unreported  Maximum ROM while avoiding pain |
| Sauers et al. [14] | Fauls routine (SPS combined with rolling and waving motions) | No-stretching on contralateral limb | Upper limbs  12 | 5  7 s + 10 circular arm motions ^a^  Unreported | Insufficient information to assess | Unreported  Feeling a stretch |
| Sayers et al. [256] | SAS | No-stretching controls | Lower limbs  3 | 3  30 s (each side)  10 to 20 s | 540 s | Unreported  85% of maximal capacity or until point of discomfort |
| Schmitt et al. [257]  Schmitt et al. [258] | PNF (CR; machine-aided; constant intensity of isometric contractions) PNF (CR; machine-aided; progressive intensity of isometric contractions) | PNF (CR; machine-aided) + resistance training | Lower limbs and trunk  2 | 4  ~10 s (each side; 1 exercise was unilateral)  5 s | 120 s | Slowly  Maximum ROM while avoiding pain or discomfort |
| Seçer, Kaya [259] | DS | DS followed by foam rolling | Lower limbs  4 | 1  15 reps (each side)  Unreported | 120 reps | 1 repetition every 2 seconds  Through active ROM |
| Sekir et al. [260]  Sekir et al. [261]  Sekir et al. [8] | DS SAS | No-stretching controls | Lower limbs  4 | 2  DS: 15 reps (each side) SAS: 20 s (each side)  15 s | DS: 240 reps SAS: 320 s | DS: slowly at first (5 reps), then 10x as quickly and powerfully as possible without bouncing SAS: unreported  To point of mild discomfort, but not pain |
| Selkar et al. [262] | SAS | SAS followed by low-intensity eccentric quadriceps exercise | Lower limbs  7 | 1  15 s (each side)  Unreported | Insufficient information to assess | Unreported  Unreported |
| Sermaxhaj et al. [263]  Sermaxhaj et al. [264]  Sermaxhaj et al. [265]  Sermaxhaj et al. [266]  Sermaxhaj et al. [267] | SAS | No-stretching controls | Full-body  17 | 1  20 s  Unreported | 340 s | Unreported  Unreported |
| Sheard, Paine [268] | PNF (CR) at 20% MVIC (hip extension)  PNF (CR) at 50% MVIC (hip extension) PNF (CR) at 100% MVIC (hip extension) | — | Lower limbs  1 | 3  ~25 s  Unreported | ~75 s | Unreported  Anchored straps with strain gauge in line at 20%, 50% and 100% MVIC of hip extension |
| Shekadar et al. [269] | SAS (cross-body stretch)  SAS (sleeper stretch) | — | Upper limbs  1 | 3  30 s  30 s | 90 s | Unreported  Unreported |
| Shitara et al. [270] | SAS | Isometric training | Upper limbs  1 | 5  60 s  Unreported | 300 s | Unreported  Unreported |
| Siatras et al. [271] | DS SAS | No-stretching controls | Lower limbs  2 | 2  30 s  30 s | 120 s | DS: as fast as possible SAS: unreported  DS: maximum ROM while avoiding pain SAS: point of limitation before pain |
| Silva et al. [272] | SAS + DS | No-stretching controls | Lower limbs  DS: 7 SAS: 9 | 1  DS: 10 reps SAS: 90 s  30 s | 70 reps + 810 s | Unreported  Maximum ROM |
| Sim et al. [273] | SAS after dynamic sports specific activities SAS before dynamic sports specific activities | No-stretching controls | Lower limbs  4 | 2 for hamstrings and quadriceps + 1 for soleus and gastrocnemius  20 s (each side)  Unreported | 240 s | Unreported  To point of light discomfort |
| Skarabot et al. [274] | SAS | Foam rolling SAS followed by foam rolling | Plantar flexors  1 | 3  30 s  15 s | 90 s | Unreported  To point of discomfort but not pain |
| Solon Júnior, Neto [275] | SPS | No-stretching controls Submaximal running | Lower limbs  4 | 1  60 s  Unreported | 240 s | Unreported  To point of mild discomfort |
| Song et al. [276] | SPS | — | Hamstrings  1 | 3  30 s (each side)  30 s | 180 s | Smoothly  To point just before discomfort |
| Stevanovic et al. [277] | DS  SAS | — | Lower limbs  DS: 8 SAS: 6 | DS: 1 SAS: 2  DS: unclear (across 1.5 to 3 basketball court lengths) SAS: 15 s (each side)  15 s | DS: insufficient information to assess SAS: 360 s | Unreported  DS: through active ROM SAS: maximum ROM |
| Stewart et al. [278] | SAS alone SAS after WU | No WU WU with no stretching | Lower limbs  3 | 1  45 s (each side)  Unreported | 270 s | Unreported  Feeling a stretch, but no pain |
| Stojanovic et al. [279] | DS  SAS | — | Lower limbs  DS: 7 SAS: 6 | 2  DS: 10 to 15 reps SAS: 15 s  10 s | DS: 140 reps SAS: 180 s | Unreported  DS: moderate to high intensity SAS: to point of mild discomfort |
| Su et al. [280] | DS DS + SAS SAS SAS + DS | No-stretching controls | Lower limbs  DS: 2 DS + SAS: 4 SAS: 2 SAS + DS: 4 | 1  30 s  10 s | DS: 60 s DS + SAS: 120 s SAS: 60 s SAS + DS: 120 s | Unreported  Unreported |
| Sudhakar, Padmasheela [281] | DS SAS | No-stretching controls | Lower limbs  4 | 15  30 s  10 s | 1800 s | DS: unreported SAS: slowly  Unreported |
| Taber et al. [282] | SAS | Body tempering | Lower limbs  2 | 3  30 s (each side)  10 s | 300 s | Unreported  To point of mild discomfort |
| Takeuchi, Tsukuda [283] | SAS  SAS (same protocol, different order) | — | Lower limbs  3 | 1  Unclear: confusing description  Unreported | Insufficient information to assess | Unreported  Maximum ROM while avoiding pain |
| Taleb-Beydokhti, Haghshenas [284] | DS  SAS | — | Lower limbs  6 | 1  15 s (each side)  Unreported | 180 s | DS: unreported SAS: slowly  To point of mild discomfort |
| Tammam, Hashem [285] | PNF (CR) | No-stretching controls Plyometrics PNF (CR) followed by plyometrics | Full-body  8 | Weeks 1-2: 3 Weeks 3-4: 4 Weeks 5-6: 5  Weeks 1-2: 24 s (each side) Weeks 3-4: 29 s (each side) Weeks 5-6: 36 s (each side)  0 s | Weeks 1-2: 1152 s Weeks 3-4: 1856 s Weeks 5-6: 2880 s | Unreported  Maximum ROM while avoiding pain |
| Taylor et al. [286] | SAS | Dynamic WU | Lower limbs and trunk  9 | 2 (stretches 1 to 6) or 1 (stretches 7 to 9)  30 s (each side)  Unreported | 900 s | Unreported  To point of light discomfort |
| Toft et al. [287] | PNF (CR) | — | Plantar flexors  1 | 5  18 s  Unreported | 90 s | Slowly  Unreported |
| Torres et al. [288] | DS SAS SAS + DS | No-stretching controls | Upper limbs and trunk  DS: 7 DS + SAS: 14 SAS: 7 | DS: 1 DS + SAS: 1+2 SAS: 2  DS: 30 reps (each side) DS + SAS: 15 s + 30 reps (each side) SAS: 15 s (each side)  Unreported | DS: 420 reps DS + SAS: 420 reps + 420 s SAS: 420 s | Unreported  Unreported |
| Tsolakis et al. [289] | BS  SAS | — | Lower limbs  3 | 3  BS: 3 reps (each side) SAS: 20 s (each side)  BS: 20 s SAS: unreported | BS: 54 reps SAS: 360 s | BS: in rapid fashion SAS: unreported  BS: unreported SAS: without feeling pain or discomfort |
| Turki et al. [290] | DS (1 set)  DS (2 sets) DS (3 sets) | — | Lower limbs  5 | LV: 1 IV: 2 HV: 3  14 reps  10 s | LV: 70 reps IV: 140 reps HV: 210 reps | Unreported  Unreported |
| Turki et al. [291] | DS | Walking | Lower limbs  5 | 1  14 reps  10 s | 70 reps | Unreported  Unreported |
| Turna et al. [292] | DS SAS | No-stretching controls | Upper limbs  5 | 3  15 s  10 s | 225 s | Unreported  Unreported |
| Unick et al. [293] | BS  SAS | — | Lower limbs  4 | 3  15 s  20 s | 180 s | BS: 1 repetition per second SAS: unreported  BS: unreported SAS: to point just before discomfort |
| Valdivia et al. [294] | PNF (unclear modality) + SPS | No-stretching controls | Lower limbs and trunk  Unreported | Week 1: 2 Week 2: 3 Week 3: 4 Week 4: 5  19 to 30 s  Unreported | Insufficient information to assess | Unreported  Moderated |
| Van Gelder, Bartz [295] | DS SAS | No-stretching controls | Full-body  DS: 14 SAS: 10 | 1  DS: 4 to 20 reps, depending on exercise (~173 total) SAS: 30 s (each side)  Unreported | DS: ~173 reps SAS: 510 s | Unreported  DS: through active ROM SAS: unreported |
| Van Zyl et al. [296] | SAS SAS + vibration | No-stretching controls SAS followed by vibration | Lower limbs  1 | 1  600 s  Not applicable | 600 s | Unreported  Unreported |
| Vasconcellos et al. [297] | BS | No-stretching controls | Lower limbs  6 | 3  10 reps  Unreported | 180 reps | With velocity  Measured through the Scale of Perceived Effort in Flexibility. However, no instructions were provided to achieve a specific intensity. |
| Vasconcellos et al. [298] | SAS (1x10s)  SAS (3x20s) | — | Lower limbs  4 | LV: 1 HV: 3  LV: 10 s (each side) HV: 20 s (each side)  Unreported | LV: 40 s HV: 240 s | Slowly  Maximum ROM |
| Veevo et al. [299] | DS  SPS | — | Upper limbs  1 | 1  DS: 8 x 4 s SPS: 30 s  Unreported | DS: 32 s SPS: 30 s | Unreported  Unreported |
| Velasque et al. [300] | SPS | No-stretching controls | Lower limbs  2 | 2  45 s (each side)  60 s | 180 s | Unreported  To point of discomfort |
| Walker et al. [301] | DS (self-selected speed) DS (maximal speed)  DS (self-selected speed) with additional forces ("active-assisted") | — | Trunk  2 | 3  5 s  10 s | 30 s | DS (self-selected speed): self-selected speed DS (maximal speed): maximal speed  Maximum ROM |
| Wallmann et al. [302] | PNF (CR) SAS | No-stretching controls | Quadriceps  1 | 3  30 s (each side)  Unreported | 180 s | Unreported  PNF: maximum ROM SAS: feeling of stretch |
| Walsh [303] | DS  SAS | — | Lower limbs  2 | DS: 3 SAS: 1  DS: 12 reps SAS: 90 s  30 s | DS: 36 reps SAS: 180 s | DS: a self-preferred velocity SAS: unreported  DS: maximum ROM SAS: to point of mild discomfort; maximum ROM |
| Werstein, Lund [304] | DS  SAS | No-stretching controls | Lower limbs  4 | 3  DS: 10 reps SAS: 30 s  10 s | DS: 120 reps SAS: 360 s | Unreported  DS: unreported SAS: to point of mild discomfort |
| West et al. [305] | SAS | Anti-Gravity Treadmill (G-trainer). Stationary Cycling (CompuTrainer) | Lower limbs  8 | 3  30 s (each side)  Unreported | 1440 s | Unreported  To point of mild discomfort, but not pain |
| Williams et al. [306] | SPS (focused stretch) SPS (gross stretch) | No-stretching controls | Pectoralis minor  1 | 2  30 s  30 s | 60 s | Unreported  Maximum ROM |
| Wilson et al. [307] | SPS | No-stretching controls | Lower limbs  5 | 4  30 s  Unreported | 600 s | Gently  To point of mild discomfort |
| Winchester et al. [308] | SPS | No-stretching controls | Lower limbs  4 | 3  30 s  10 to 20 s | 360 s | Unreported  To stretch-induced discomfort similar to that normally felt during their daily stretching activities |
| Wong et al. [309] | SAS *The authors initially termed passive stretching, but later corrected to unassisted stretching, and description of each stretch reveals these were active*. | No-stretching controls | Lower limbs  2 | 2  20 s (each side)  60 s | 160 s | Slowly  To point of mild discomfort |
| Yamaguchi et al. [310] | DS | No-stretching controls | Lower limbs  5 | 1  10 reps  0 s | 50 reps | Quickly and powerfully; as quickly as possible without bouncing  Unreported |
| Yamaguchi et al. [311] | DS | No-stretching controls | Lower limbs  5 | 1  10 reps (each side)  0 s | 100 reps | 1 repetition every 2 seconds  Unreported |
| Yamaguchi et al. [312] | DS | No-stretching controls | Lower limbs  5 | 1  10 reps (each side)  0 s | 100 reps | 1 repetition every 2 seconds  Unreported |
| Yaşli, Müniroğlu [313] | SAS | No-stretching controls | Full-body  10 | 2  15 s  15 s | 360 s | Unreported  Unreported |
| Yildiz [314] | DS SAS | Foam rolling No-stretching controls | Lower limbs  Unreported | 1  30 s  15 s between exercises 30 s between sides | Insufficient information to assess | Unreported  Unreported |
| Yıldırım et al. [315] | DS SAS | No-stretching controls | Full-body  5 | 2  15 s  5 s | 150 s | DS: 1 repetition every 2 seconds SAS: unreported  To point of maximum tolerance |
| Young et al. [316] | SAS + SPS | No-stretching controls | Lower limbs  3 | 3  30 s (each side)  Unreported | 540 s | Slowly  Maximum ROM |
| Zakas et al. [317] | SAS (1 set) SAS (2 sets) SAS (6 sets) | SAS (1 set) after a WU SAS (2 sets) after a WU SAS (6 sets) after a WU | Lower limbs  5 | Single set group: 1 Double set group: 2 Six-set group: 6  Single set: 30 s (each side) Double set: 15 s (each side) Six-set: 5 s (each side)  10 s | 300 s | Slowly  Maximum ROM while avoiding pain |
| Zakas [318] | SAS (1 set)  SAS (2 sets) SAS (6 sets) | — | Lower limbs  5 | Single set group: 1 Double set group: 2 Six-set group: 6  Single set: 30 s (each side) Double set: 15 s (each side) Six-set: 5 s (each side)  10 s | 300 s | Slowly  Maximum ROM while avoiding pain |
| Zakas et al. [319] | SAS (3 sets)  SAS + SPS (20 sets) | — | Quadriceps  1 | LV: 3 HV: 20  15 s  15 s | LV: 45 s HV: 300 s | Unreported  Maximum ROM while avoiding pain |
| Zakas et al. [320] | SAS (1 set)  SAS (2 sets) + SPS (8 sets) SAS (2 sets) + SPS (14 sets) | — | Quadriceps  LV: 1 IV: 2 HV: 2 | LV: 1 IV: 10 HV: 16  30 s  15 s | LV: 30 s IV: 300 s HV: 480 s | Unreported  Feeling a stretch, but no pain |
| Zakas et al. [321] | SAS (4 sets)  SAS + SPS (32 sets) | — | Quadriceps  1 | LV: 4 HV: 32  15 s  15 s | LV: 60 s HV: 480 s | Unreported  Feeling a stretch, but no pain |
| Zakas et al. [322] | SPS alone | SPS after WU  WU alone | Lower limbs and trunk  6 | 3  15 s  10 s | 45 s | Slowly  Maximum ROM while avoiding pain |
| Zmijewski et al. [323] | BS SAS | No-stretching controls | Lower limbs  2 | 3  20 s (each side)  10 s | 240 s | BS: unreported SAS: slowly and smoothly  BS: unreported SAS: to point of light discomfort, not pain |
| Zourdos et al. [324] | DS | No-stretching controls | Lower limbs  10 | 2  4 reps  Unreported | 80 reps | Unreported  Unreported |
| 孙勇 [325] | SAS | Cycling at 20% VO_2_max No-stretching controls | Lower limbs  5 | 3  20 to 30 s  Unreported | 300 s | Unreported  Unreported |

*Legend (ordered alphabetically)*: CMJ – Countermovement jump. BS – Ballistic stretching. CRAC – contract-relax agonist contract. CRAntC – contract-relax antagonist contract. CWI - Cold-water immersion. DS – Dynamic stretching. GAS – Global active stretching^®^. GPR – Global postural re-education. HR – Hold-relax. HV – High volume. IV – Intermediate volume. LV – Low volume. PNF – Proprioceptive neuromuscular facilitation. RS – Rhythmic stabilization. SAS – Static active stretching. SPS – Static passive stretching. WU – Warm-up. ^a^ Initially, the authors mentioned 3 to 7 s + 5 to 10 reps, but later they said they made it uniform and always used 7 s + 10 reps.

*Reporting details*: Stretching modalities are reported in alphabetical order. In the modalities’ column, dose-related information is only presented in case those were comparators (*e.g.*, LV versus HV stretching interventions). Whenever the signal + is used, it means a combined intervention (*e.g.*, the group performed SAS combined with SPS). Regarding stretching dose (*e.g.*, no. stretches, rest between sets), if a single number is provided it is implied that it was equated across the multiple groups or interventions. The minimum stretching volume per session is the multiplication of the minimum values for no. stretches, no. sets and no. reps/time, and excluding rest time.

***ESM 2.4. Outcome-level information***

Full reporting of outcome-level details is delivered in supplementary table 4.

**Supplementary table 4.** Outcome-level details

| **Trials** | **Physiological outcomes** | **Biomechanical outcomes** | **Neural /**  **psychological outcomes** | **Performance outcomes** | **Injury-related outcomes** |
| --- | --- | --- | --- | --- | --- |
| Abadi et al. [18] | — | — | — | Artistic gymnastics-specific test (balance in the vault) Balance (dynamic and static centre of pressure) | — |
| Agopyan et al. [19] | — | — | — | Swimming-specific tests (25-m and 50-m swim, flutter kicking time) | — |
| Ahmadabadi et al. [20] | — | — | — | Balance (dynamic and static centre of pressure) | — |
| Akarsu et al. [21] | — | — | — | Strength/power (vertical jump) | — |
| Akehurst et al. [22] | — | Muscle tightness | Pain | — | — |
| Alipasali et al. [23]  Alipasali et al. [24] | — | — | — | Speed (4.5-m and 9-m sprint) Strength/power (countermovement block jump) | — |
| Almeida Júnior et al. [25] | Inflammation | — | — | — | — |
| Almeida Júnior et al. [26] | — | — | — | Judo-specific test (Tokui Waza) ROM (sit and reach) Strength/power (CMJ, handgrip, isometric pull-up, medicine ball throwing, SJ) | — |
| Alp [27] | — | — | — | Strength/power (isokinetic hip flexion-extension) | — |
| Alp et al. [28] | — | — | — | Strength/power (isokinetic knee and ankle flexion-extension) | — |
| Amir Vazini, Parnow [29] | — | — | — | COD (Illinois Agility Test) ROM (knee extension) Speed (30-m sprint) Strength/power (vertical jump) | — |
| Amiri-Khorasani [30] | — | Kinematics during soccer instep kicking | — | — | — |
| Amiri-Khorasani [31] | — | — | — | Balance (one-legged stance test, Star excursion balance test) | — |
| Amiri-Khorasani, Ferdinands [32] | — | Kinematics during soccer instep kicking | — | — | — |
| Amiri-Khorasani, Kellis [33] | — | EMG (vastus lateralis) | — | Soccer-specific test (ball velocity during soccer instep kicking) | — |
| Amiri-Khorasani, Sotoodeh [34] | — | — | — | COD (shuttle run) ROM (v-sit) Speed (10-m and 20-m sprint) Strength/power (vertical jump) | — |
| Amiri-Khorasani et al. [35] | — | EMG (vastus lateralis) | — | Soccer-specific test (ball velocity during soccer instep kicking) | — |
| Amiri-Khorasani et al. [36] | — | — | — | Speed (10-m and 20-m sprints) | — |
| Amiri-Khorasani et al. [37]  Amiri-Khorasani et al. [38] | — | Kinematics during soccer instep kicking | — | — | — |
| Amiri-Khorasani et al. [39] | — | — | — | COD (Illinois Agility Test) | — |
| Andre et al. [40] | — | — | — | Strength/power (bench press) | — |
| Andrejić et al. [41] | — | — | — | ROM (stand and reach) Speed endurance (4x15-m sprint) Strength/power (long jump, vertical jump) | — |
| Annino et al. [42] | — | — | — | Strength/power (CMJ, CMJ with arm swing) | — |
| Ari [43] | — | — | — | ROM (sit and reach) Speed (10-m sprint) Strength/power (CMJ, medicine ball throw) | — |
| Arihiro et al. [44] | — | — | — | ROM (hip to floor distance during overhead squat) | — |
| Avaz et al. [45] | Serum CK Serum lactate dehydrogenase | — | — | — | — |
| Avedesian et al. [46] | — | Kinematics of landing after CMJ | — | — | — |
| Avloniti et al. [47] | — | — | — | COD (t-test) Speed (10-m and 20-m sprint) | — |
| Avloniti et al. [48] | — | — | — | COD (t-test) Speed (10-m and 20-m sprint) | — |
| Ayala, De Baranda [49]  De Baranda, Ayala [50] | — | — | — | COD (T test) ROM (sit and reach) Speed (10-m and 30-m sprint) | — |
| Ayala et al. [51] | — | — | — | ROM (passive straight leg raise) | — |
| Aydoǧ et al. [52] | — | — | — | Strength/power (knee and hip extension and flexion) | — |
| Azuma, Someya [53] | — | Muscle tightness | — | — | Number and rate of injuries, injury type, circumstances, severity, situation |
| Babbar et al. [54] | — | — | — | Strength/power (5 step jumps) | — |
| Balci et al. [11] | — | — | — | ROM (active knee extension, sit and reach) | — |
| Bali, Guru [55] | — | — | — | ROM (hip abduction) | — |
| Barbosa et al. [56] | — | EMG (biceps femoris, rectus femoris) | — | COD (T-drill) Speed (20-m sprint) Strength/power (CMJ) | — |
| Baumgart et al. [57] | — | Anterior tibial translation | — | — | — |
| Bazett-Jones et al. [58] | — | — | — | ROM (active knee extension) Speed (55-m sprint) Strength/power (vertical jump) | — |
| Bazett-Jones et al. [59] | — | — | — | Strength/power (isometric squat test, leg press 1 RM) ROM (active bent-leg hamstrings, active prone quadriceps, active straight leg raise, gravity hip flexor) | — |
| Beckett et al. [60] | — | — | — | COD (6x20-m sprints with 100° COD every 4 m) Speed endurance (6x20-m sprints) | — |
| Behara, Jacobson [61] | — | — | — | ROM (hip flexion) Strength/power (isometric leg extension, vertical jump) | — |
| Belkhiria-Turki et al. [62] | — | — | — | ROM (sit and reach) Speed (20-m sprint) Speed endurance (6x20-m sprints) Strength/power (CMJ, SJ) | — |
| Belkhiria-Turki et al. [63] | — | — | — | Balance (Star Excursion Balance Test) | — |
| Bello et al. [64] | — | — | Pain | ROM (hip flexion) | Number of injuries |
| Ben Maaouia et al. [65] | — | — | — | COD (T-test) | — |
| Bingul et al. [66] | — | — | — | COD (T-test) | — |
| Bishop, Middleton [67] | — | — | — | COD (Illinois Agility Test) Speed (20-m sprint) Strength/power (CMJ) | — |
| Bogdanis et al. [68] | — | — | — | ROM (hip extension, knee flexion) Strength/power (single-leg CMJ) | — |
| Boudenot et al. [69] | — | — | — | Strength/power (Wingate) | — |
| Bouthin, Edouard [70] | — | — | — | — | Injury incidence, characteristics and severity |
| Brodowicz et al. [71] | — | — | — | ROM (straight leg raise) | — |
| Burkett et al. [72] | — | — | — | Strength/power (vertical jump) | — |
| Burkett et al. [73] | — | — | — | Strength/power (vertical jump) | — |
| Buttifant, Hrysomallis [74] | — | — | — | Strength/power (20-kg weighted jump squat) | — |
| Caliskan et al. [75] | Blood flow | Muscle stiffness | — | — | — |
| Caplan et al. [76] | — | Kinematics during 30-m sprint | — | — | — |
| Carvalho et al. [77] | — | — | — | Strength/power (CMJ, SJ) | — |
| Carvalho et al. [78] | — | — | — | Strength/power (vertical jump) | — |
| Celik [79] | — | — | — | ROM (back scratch test) Strength/power (shoulder external and internal rotation) Volleyball-specific test (accuracy and speed of volleyball spike) | — |
| César et al. [80] | Blood lactate | — | — | Brazilian Jiu-Jitsu-specific test (kimono grip)  Strength endurance (handgrip) Strength/power (handgrip) | — |
| Cetin et al. [81] | — | — | — | COD (Illinois Agility Test) Speed (30-m sprint) Strength/power (CMJ) | — |
| Chaouachi et al. [82] | — | — | — | COD (T test) Speed (30-m sprint) Strength/power (CMJ, 5-Jump) | — |
| Chaouachi et al. [83] | — | — | — | ROM (hip flexion) Strength/power (isokinetic leg flexion) | — |
| Chatzopoulos et al. [84] | — | — | — | Proprioception (force sense matching during MIVC) ROM (active straight leg raise) Speed (reaction and movement time using reaction timer apparatus) | — |
| Chatzopoulos et al. [85] | — | — | — | Balance (swinging platform) COD (505 test) Speed (reaction and movement time using reaction timer apparatus) | — |
| Chatzopoulos et al. [86] | — | — | — | ROM (active straight leg raise) Speed (reaction and movement time using reaction timer apparatus) | — |
| Chen et al. [87] | — | Pennation angle Muscle stiffness Muscle thickness | — | ROM (passive hip flexion) Strength/power (isokinetic knee flexion) | — |
| Chen et al. [88] | — | Shank circumference | Perceived discomfort | — | — |
| Chinnavan et al. [89] | — | — | — | ROM (sit and reach) | — |
| Christensen et al. [91] | — | — | — | COD (t-test) Speed (10-m and 20-m sprint) Strength/power (medicine ball throw, vertical jump) | — |
| Christensen, Nordstrom [90] | — | — | — | Strength/power (vertical jump) | — |
| Chtourou et al. [92] | — | — | — | Strength/power (CMJ, SJ) | — |
| Church et al. [93] | — | — | — | ROM (sit and reach) Strength/power (CMJ) | — |
| Colak [94] | — | — | — | ROM (knee extension and flexion) Strength/power (isokinetic knee extension and flexion) | — |
| Coons et al. [95] | — | — | — | ROM (knee extension) | — |
| Correia et al. [96] | — | — | — | Balance (Star Excursion Balance test) | — |
| Cwirlej-Sozanska et al. [97] | — | — | — | ROM (heel-buttock test) Volleyball-specific test (vertical jump with spike attack) | — |
| da Silva et al. [98] | — | — | — | Female: strength/power (horizontal jump) Male: speed ( 30-m sprint) | — |
| Dalamitros et al. [99] | — | — | — | Swimming-specific test (50-m sprint) | — |
| Dallas et al. [100] | — | — | — | ROM (sit and reach)  Strength/power (CMJ, SJ) | — |
| Dalrymple et al. [101] | — | — | — | Strength/power (CMJ) | — |
| Damasceno et al. [102] | — | EMG (biceps femoris, gastrocnemius medialis, vastus medialis) Kinematics during 3-km running | — | Endurance (3-km running) ROM (sit and reach) Strength/power (drop jump) | — |
| Darcadia et al. [103] | — | — | — | Speed (30-m sprint) Strength/power (horizontal jump, vertical jump) | — |
| Dawson et al. [104] | — | — | Perceived soreness | ROM (sit and reach) Speed (6-s cycling sprint) Strength/power (vertical jump) | — |
| de Almeida Leme et al. [105] | — | — | TEF (Stress soccer test) | — | — |
| de Castro et al. [106] | — | — | — | ROM (hip rotation) | — |
| De Oliveira Júnior et al. [107] | — | — | — | Strength/power (CMJ) | — |
| de Oliveira, Pinto Lopes Rama [108] | — | — | — | Speed (20-m sprint) Strength/power (CMJ) | — |
| de Rezende et al. [109] | — | — | — | Strength/power (CMJ) | — |
| Di Cagno et al. [110] | — | — | — | Rhythmic gymnastics-specific tests (technical leaps) Strength/power (CMJ, hopping test, SJ) | — |
| Di Cagno et al. [111] | — | — | — | Strength endurance (push-ups) Strength/power (ball throwing, MVIC) | — |
| Donti et al. [112] | — | — | — | ROM (straight leg raise) | — |
| Donti et al. [113] | — | Fascicle length Muscle thickness Pennation angle | — | ROM (ankle dorsiflexion) | — |
| Donti et al. [114] | — | — | — | ROM (straight leg raise) | — |
| Drews, Goltz [115] | — | — | — | ROM (hip abduction) | — |
| Ebadi, Cetin [116] | — | — | — | Strength/power (isokinetic knee extension) | — |
| Egan et al. [7] | — | — | — | Strength/power (isokinetic knee extension) | — |
| Eken, Bayer [117] | — | — | — | ROM (sit and reach) Strength/power (CMJ, handgrip) | — |
| Espi-Lopez et al. [118] | — | — | — | Balance (Star Excursion Balance Test) ROM (ankle dorsiflexion, fingertip-to-floor test, hip external and internal rotation, hip flexion, knee flexion) | — |
| Evetovich et al. [119] | — | EMG (rectus femoris) | — | Strength/power (leg extension) | — |
| Faigenbaum et al. [120] | — | — | — | COD (pro-agility shuttle) Speed (10-yard sprint) Strength/power (medicine ball toss, vertical jump) | — |
| Faigenbaum et al. [121] | — | — | — | Strength/power (medicine ball toss, vertical jump) | — |
| Faigenbaum et al. [122] | — | — | — | Speed (10-yard sprint) Strength/power (long jump, medicine ball toss, vertical jump) | — |
| Famisis [123] | — | — | — | ROM (passive hip flexion, extension and abduction, passive knee flexion, passive ankle dorsiflexion) Speed (20-m sprint) | — |
| Farshidi et al. [124] | — | — | — | Proprioception (knee joint position sense) ROM (knee extension) | — |
| Fattahi-Bafghi, Amiri-Khorasani [125] | — | — | — | COD (Illinois Agility test) Strength/power (vertical jump) | — |
| Favero et al. [126] | — | — | — | ROM (sit and reach) Speed (40-m sprint) | — |
| Feitosa Junior et al. [127] | — | — | — | Strength/power (wrist MVIC) | — |
| Fernandes et al. [128] | — | EMG (vastus lateralis) Ground reaction forces during drop jump | — | Strength/power (drop jump) | — |
| Ferreira et al. [129] | — | — | — | ROM (sit and reach) Strength/power (CMJ) | — |
| Ferri-Caruana et al. [130] | — | — | — | Artistic Gymnastics-specific test (split leap) ROM (hip flexion and extension) Strength/power (hip isometric strength, SJ) | — |
| Fletcher, Anness [131] | — | — | — | Speed (50-m sprint) | — |
| Fletcher, Jones [132] | — | — | — | Speed (20-m sprint) | — |
| Fletcher, Monte-Colombo [133]  Fletcher, Monte-Colombo [134] | Core temperature Heart rate | EMG (biceps femoris, rectus femoris) Kinematics during isokinetic knee extension and flexion | — | COD (Balsom test) Speed (20-m sprint) Strength/power (CMJ, drop jump) | — |
| Forte et al. [135] | — | — | — | Strength/power (Seargent Jump test) | — |
| Frantz, Ruiz [136] | — | — | — | Strength/power (standing long jump, stationary vertical jump) | — |
| Fredericson et al. [137] | — | Ground reaction forces during gait Iliotibial band length and strength Kinematics during gait | — | — | — |
| Frikha et al. [138] | Heart rate | — | RPE | Coordination (global coordination test) Soccer-specific test (Instep kicking accuracy in free and time-pressure conditions) | — |
| Funk et al. [139] | — | — | — | ROM (active knee extension) | — |
| Funk et al. [140] | — | — | — | ROM (knee extension) | — |
| Gabbe et al. [141] | — | — | — | — | Compliance (only 46.8% of players completed at least 2 of the 5 sessions!) Risk ratio of hamstring injury |
| Galazoulas [142] | — | — | — | ROM (knee and hip flexion) Strength/power (isokinetic knee extension and flexion) | — |
| Galazoulas [143] | — | — | — | Speed (10-m sprint) Strength/power (CMJ) | — |
| Galetin et al. [144] | — | — | — | Strength/power (CMJ, High Jump with Arm Swing, SJ) | — |
| Gao et al. [145] | — | EMG (biceps femoris, gastrocnemius, rectus femoris) Ground reaction forces after vertical jump Kinematics during vertical jumping | — | — | — |
| Gelen [146] | — | — | — | Soccer-specific tests (slalom dribbling, penalty kick) Speed (30-m sprint) | — |
| Gelen et al. [147] | — | — | — | Strength/power (CMJ, SJ) | — |
| Gelen et al. [148] | — | — | — | Tennis-specific test (tennis serve speed) | — |
| Gergley [149]  Gergley [150] | — | Clubhead speed, distance, accuracy, and ball contact | — | — | — |
| Ghasemi et al. [151] | — | EMG (triceps surae) | Pain | Strength endurance (MVC of triceps surae to exhaustion) | — |
| Gonçalves et al. [152] | — | EMG (biceps femoris, semitendinosus) | — | COD (sinuous running test) ROM (sit and reach) Speed (50-m sprint) Strength/power (knee flexion, long jump, vertical jump) | — |
| Gürses, Akgül [153] | — | — | — | COD (Illinois Agility Test) Speed (20-m sprint) Strength/power (CMJ) Soccer-specific test (20-m sprint with ball) | — |
| Haag et al. [154] | — | — | — | Baseball-specific test (pitching velocity and accuracy) | — |
| Haddad et al. [155] | — | — | — | Speed (30-m sprint) Speed endurance (6x40-m sprints) Strength/power (5 jump test) | — |
| Haddad et al. [156] | — | EMG (rectus femoris, semimembranosus, semitendinosus, vastus lateralis, vastus medialis) | — | Strength/power (isokinetic knee extension) | — |
| Han et al. [157] | — | — | — | Speed (40-m sprint) Strength/power (Margaria-Kalamen Power Test) | — |
| Heisey, Kingsley [158] | — | — | Perceived fatigue | ROM (sit and reach) Strength/power (back squat) | — |
| Herman, Smith [159] | Dual-energy x-ray absorptiometry | — | — | COD (300-yard shuttle run) ROM (trunk extension, sit and reach) Strength/power (Army Physical Fitness, isokinetic knee flexion-extension, medicine ball throwing). | — |
| Higuchi et al. [160] | — | Muscle length (pectoralis minor) | — | ROM (shoulder external and internal rotation) | — |
| Holt, Lambourne [161]  *Erratum*: Holt, Lambourne [326] | — | — | — | Strength/power (vertical jump) | — |
| Hough et al. [162] | — | EMG (vastus medialis) | — | Strength/power (vertical jump) | — |
| Hsu et al. [163] | — | EMG (biceps femoris, gastrocnemius, rectus femoris) | — | COD (Edgren Sidestep Test) ROM (sit and reach) Strength/power (medicine ball chest throw, standing broad jump) Table tennis-specific test (ball speed) | — |
| Huang et al. [164] | — | EMG (biceps brachii, deltoid, triceps brachii) | — | Balance (heel raise test) ROM (ankle dorsiflexion and plantar flexion) Speed (linear and curved 55-m sprints) | — |
| Huang, Zhu [165] | — | EMG (biceps femoris, gastrocnemius, rectus femoris) | — | Strength/power (lower limbs MVC) | — |
| Ide et al. [166] | — | — | — | ROM (sit and reach) Speed (30-m sprint)  Strength/power (CMJ, SJ) | — |
| Ishak et al. [167]  Ishak et al. [168] | Blood lactate Heart rate | — | Perceived fatigue RPE | Speed endurance (6x20-m and 6x30-m sprints) | — |
| Ishak et al. [169] | Blood lactate Heart rate | — | Perceived fatigue RPE | Speed endurance (5x20-m sprints) | — |
| Jang et al. [170] | — | Kinematics during jump smash in badminton | — | — | — |
| Jemni et al. [171] | — | — | — | ROM (split test) Strength/power (isokinetic knee flexion and extension) | — |
| Jing, Yang [172] | Heart rate | Ground reaction forces after CMJ Kinematics during vertical jump landing Leg stiffness | — | — | — |
| Johnson et al. [173] | — | — | — | Artistic Gymnastics-specific test (dynamic ROM and jump height during split jumps) | — |
| Jordan et al. [174] | — | — | — | Soccer-specific test (Balsom agility test while dribbling a soccer ball) | — |
| Junqueira et al. [175] | — | — | — | ROM (dorsal Shober, lumbar Shober, sit and reach, third finger-floor) | — |
| Kafkas et al. [176] | — | — | — | Swimming-specific test (50-m crawl and 50-m breaststroke) | — |
| Karloh et al. [177]  Karloh et al. [178] | — | — | — | ROM (hip extension) | — |
| Kazemi et al. [179] | — | EMG (biceps brachii, deltoid, triceps brachii) | — | — | — |
| Kilit et al. [180] | — | — | — | COD (T test) Speed (20-m sprint) | — |
| Konrad et al. [181] | VO_2_ | Kinematics during 15-min running on treadmill | — | — | — |
| Kornberg et al. [182] | Skin temperature | — | — | — | — |
| Krčmár et al. [183] | — | — | — | Strength/power (CMJ) | — |
| Kruse et al. [184]  Kruse et al. [185] | — | Kinetics during vertical jump | — | Strength/power (CMJ) | — |
| Kurt [186] | — | — | — | ROM (stand and reach) | — |
| Kurt, Firtin [187] | — | — | — | COD (Illinois Agility test) ROM (stand and reach) Speed endurance (6x35 m sprints) | — |
| Kurtdere et al. [188] | — | Muscle stiffness | — | ROM (sit and reach) Strength/power (jump-plyometric test) | — |
| Kyranoudis et al. [189] | — | — | — | ROM (hip flexion) Strength/power (CMJ with arms akimbo and with free arms) | — |
| Kyranoudis et al. [190] | — | — | — | Speed (20-m sprint) | — |
| Laudner et al. [191] | — | — | — | ROM (shoulder external and internal rotation, horizontal adduction) | — |
| Li et al. [192] | — | — | — | ROM (Shoulder Rotation Index) Strength/power (Isokinetic shoulder rotation) Swimming-specific test (50m freestyle swimming) | — |
| Lin et al. [193] | — | Muscle stiffness | — | Badminton-specific test (badminton COD test)  ROM (Ely's test, popliteus angle test) Strength/power (CMJ) | — |
| Little, Williams [194] | — | — | — | COD (Zig-zag) Speed (10-m sprint and flying 20-m sprint) Strength/power (CMJ) | — |
| Lotfi et al. [195] | Blood Parameters (White Blood Cells, Red Blood Cells, Haemoglobin, Haematocrit, and Platelet) Enzymatic Activity of CK and LDH | — | — | Speed endurance (Running-based Anaerobic Sprint Test) | — |
| Loughran et al. [196] | — | — | — | Speed (10-m, 20-m, and 40-m sprints) Strength/power (CMJ) | — |
| Lowery et al. [197] | — | EMG (gastrocnemius lateralis) Ground contact time during 1-mile run | — | Endurance (1 mile uphill) ROM (sit and reach) | — |
| Makaruk et al. [198] | — | — | — | ROM (active knee extension) Strength/power (knee flexion) | — |
| Manzi et al. [199] | — | — | — | ROM (passive straight leg raise) | — |
| Mariscal et al. [200] | — | — | — | Speed (40-m sprint) Strength/power (Abalakov jump, CMJ) | — |
| Martin et al. [201] | — | — | — | Speed (reaction and movement time using reaction timer apparatus) Strength/power (CMJ, SJ) | — |
| Martinez-Chicote et al. [202] | — | — | — | Tennis-specific test (Sideward movement test) | — |
| Mascarin et al. [203] | — | — | — | Handball-specific test (ball throwing speed test) ROM (shoulder internal and external rotation) Strength/power (medicine ball throw) | — |
| McNeal, Sands [204] | — | — | — | Strength/power (drop jump) | — |
| McNeal et al. [205] | — | — | — | ROM (split test) | — |
| Meerits et al. [206] | — | Muscle elasticity Muscle tone | — | Strength/power (SJ) | — |
| Melocchi et al. [207] | — | — | — | Artistic Gymnastics-specific tests (acrobatic gymnastic jump, forward oversplit figure) Strength/power (CMJ, SJ) | — |
| Mendez-Sanchez et al. [12] | — | — | — | ROM (finger-to-floor, modified Schober tests, seated slump test, sit and reach, straight leg raise) | — |
| Merrigan et al. [208] | — | — | Mood state Perceived fatigue Perceived soreness RPE | — | — |
| Mikolajec et al. [209] | — | — | — | Speed (5-m and 20-m sprint) Strength/power (CMJ) | — |
| Miladi et al. [210] | Blood lactate Heart rate Oxygen uptake kinetics | — | — | Endurance (time to exhaustion in supramaximal cycling) | — |
| Mojock et al. [211] | Energy cost (running) Heart rate Relative VO_2_ | — | RPE | Endurance (distance and speed in treadmill running test) ROM (sit and reach) | — |
| Molacek et al. [212] | — | — | — | Strength/power (bench press 1RM) | — |
| Montalvo, Dorgo [213] | — | — | — | Strength/power (CMJ, depth jump, SJ) | — |
| Moore, Hutton [214] | — | EMG (rectus femoris, semitendinosus) | Pain | ROM (hip flexion) | — |
| Mor et al. [215] | — | — | — | Balance (flamingo test) COD (t-test) Speed (30-m sprint) Strength/power (isokinetic knee extension) Soccer-specific test (ball kicking speed) | — |
| Moran et al. [216] | — | Clubhead and ball speed Swing paths, central impact points and club face angle | — | — | — |
| Moran et al. [217] | — | — | — | Swimming-specific test (50-m freestyle) | — |
| Moreno-Perez et al. [218] | — | — | — | COD (505 test) ROM (hip flexion and extension) Speed (5-m and 10-m sprints) Strength/power (CMJ) | — |
| Needham et al. [219] | — | — | — | Speed (10-m and 20-m sprints) Strength/power (CMJ) | — |
| Nelson et al. [220] | — | — | — | Speed (20-m sprint) | — |
| Nobre et al. [221] | — | — | — | Handball-specific test (throwing speed and accuracy) ROM (shoulder internal and external rotation) Strength/power (Kendall's manual muscle test) | — |
| Notarnicola et al. [222] | — | — | — | ROM (forward trunk bending, straight leg raise) | — |
| Nuri et al. [223] | — | — | — | ROM (passive and active ankle dorsiflexion) | — |
| O'Sullivan et al. [224] | — | — | — | ROM (passive knee extension) | — |
| Ohshita, Mitsuzono [10] | Blood lactate VO_2_max | — | — | ROM (hip flexion, ankle dorsiflexion) | — |
| Olivares-Arancibia et al. [225] | — | — | — | ROM (modified sit and reach, passive straight leg raise) | — |
| Oliveira et al. [226] | — | — | RPE | ROM (sit and reach) Speed (10-m, 20-m, and 30-m sprints) Strength/power (CMJ, SJ) | — |
| Oña Tacan et al. [227] | — | — | — | ROM (lateral split test, frontal split test) | — |
| Oskouei et al. [228] | — | — | — | Proprioception (knee joint position sense) | — |
| Osternig et al. [229] | — | EMG (biceps femoris, vastus lateralis) | — | ROM (knee extension) | — |
| Oyama et al. [9] | — | — | — | ROM (shoulder internal rotation, internal rotation, and horizontal adduction) | — |
| Pagaduan et al. [230] | — | — | — | Strength/power (CMJ) | — |
| Panidi et al. [231] | — | Fascicle length Muscle cross-sectional area | — | ROM (ankle dorsiflexion) Strength/power (one-leg CMJ) | — |
| Papadimitriou et al. [232] | Blood lactate | — | — | ROM (hip flexion and extension, plantar flexion, and dorsiflexion) Swimming-specific test (10 s free kick, 2'10'' free kick) | — |
| Papia et al. [233] | — | — | — | ROM (hip and knee extension and flexion) Strength/power (one and two-leg CMJ) | — |
| Pellegrini et al. [234] | — | Kinematics of the scapula | — | — | — |
| Penichet-Tomas et al. [235] | — | — | — | ROM (sit and reach) | — |
| Pojskic et al. [236] | — | — | — | COD (T test) Speed (15-m sprint) Strength/power (CMJ) | — |
| Polat et al. [237] | — | — | — | Balance (Y balance) COD (Illinois Agility Test) ROM (sit and reach) Speed (20-m sprint) Strength/power (handgrip dynamometry; isokinetic knee extension) | — |
| Pooley et al. [238] | CK concentrations Muscle oedema | — | Perceived soreness | Strength/power (CMJ with arms) | — |
| Pooley et al. [239] | CK concentrations Muscle oedema | — | Perceived soreness | Strength/power (CMJ with arms) | — |
| Popelka, Pivovarniček [240]  Popelka et al. [241] | — | — | — | COD (4x10 m shuttle run) ROM (sit and reach) Speed (E-test, run to cones) Strength/power (medicine ball throw) Volleyball-specific tests (block jump, spike jump) | — |
| Popelka, Pivovarniček [242] | — | — | — | ROM (sit and reach) Speed (E-Test, run to cones) Strength/power (medicine ball throwing, sit-ups) Volleyball-specific tests (block jump, spike jump) | — |
| Portilla-Dorado et al. [243] | — | EMG (biceps femoris, semitendinosus) | — | ROM (sit and reach) Strength/power (Abalakov jump, CMJ, SJ) | — |
| Racil et al. [244] | — | — | — | Motor skills (running, hopping, leaping) ROM (hip flexion and extension) Strength/power (CMJ, SJ, stiffness jump) Track and Field-specific test (60-m hurdle sprint) | — |
| Reis et al. [245] | — | EMG (rectus femoris, vastus lateralis) | — | Strength/power (knee extension MVC) | — |
| Reuther et al. [246] | — | — | — | ROM (shoulder internal and external rotation) | — |
| Robey et al. [247] | Haemoglobin concentration and haematocrit Plasma volume Serum CK levels | — | Perceived soreness | Strength/power (isokinetic testing) Rowing-specific test (rowing ergometer) | — |
| Rodriguez-Marroyo et al. [248] | Heart rate | — | RPE | — | — |
| Rogan et al. [249] | — | — | — | Ice-hockey-specific test (20-m sprint on ice) Strength/power (drop jump) | — |
| Romero-Franco et al. [250] | — | — | — | Balance (unipodal static postural balance) Proprioception (knee joint position sense) | — |
| Sagiroglu et al. [251] | — | — | — | ROM (sit and reach) Strength/power (CMJ) | — |
| Sampaio-Jorge et al. [252] | — | — | Pain | Strength/power (CMJ) | — |
| Sánchez-Sánchez et al. [253] | — | — | — | Strength/power (horizontal jump) COD endurance (6 x 30 m with 180° COD) | — |
| Sands et al. [254] | — | — | — | ROM (passive and active forward split) | — |
| Sands et al. [255] | — | — | — | ROM (rear split) | — |
| Satkunskiene et al. [13] | — | Muscle stiffness Muscle viscoelasticity Passive-resistance torque | — | Proprioception (active knee-joint position sense) ROM (straight-leg raise) | — |
| Sauers et al. [14] | — | Muscle tightness | — | ROM (shoulder external and internal rotation) | — |
| Sayers et al. [256] | — | — | — | Speed (30-m sprint) | — |
| Schmitt et al. [257]  Schmitt et al. [258] | — | — | — | ROM (sit and reach - standard version plus two modified versions) | — |
| Seçer, Kaya [259] | — | — | — | Balance (Y balance test) COD (t test) ROM (sit and reach) | — |
| Sekir et al. [260]  Sekir et al. [261]  Sekir et al. [8] | — | EMG (rectus femoris, vastus lateralis) | — | Strength/power (isokinetic knee flexion and extension) | — |
| Selkar et al. [262] | — | — | Pain | Activities of Daily Living (Functional independence measure score) | — |
| Sermaxhaj et al. [263]  Sermaxhaj et al. [264]  Sermaxhaj et al. [265]  Sermaxhaj et al. [266]  Sermaxhaj et al. [267] | — | — | — | COD (20-m zig-zag running) ROM (Sit and reach) Soccer-specific test (20-m zig-zag running with ball) Speed (5-m, 10-m and 30-m sprints) Strength/power (CMJ, isokinetic knee extension and flexion) | — |
| Sheard, Paine [268] | — | — | — | ROM (straight leg raise) | — |
| Shekadar et al. [269] | — | — | — | ROM (shoulder internal rotation and horizontal adduction) | — |
| Shitara et al. [270] | — | — | — | ROM (elbow flexion and extension, shoulder horizontal abduction, external, and internal rotation) Strength/power (shoulder internal and external rotation) | Injury rate |
| Siatras et al. [271] | — | — | — | Artistic Gymnastics-specific test (speed on running for handspring vaulting) | — |
| Silva et al. [272] | — | — | — | ROM (hip flexion) Strength/power (CMJ) | — |
| Sim et al. [273] | Heart rate | — | RPE | Speed endurance (3x20-m sprints) | — |
| Skarabot et al. [274] | — | — | — | ROM (passive ankle dorsiflexion) | — |
| Solon Júnior, Neto [275] | — | — | — | Speed (10-m sprint) Strength/power (CMJ) | — |
| Song et al. [276] | — | Ground reaction forces during vertical jump Kinematics during vertical jump | — | — | — |
| Stevanovic et al. [277] | Spinal excitability | — | — | Strength/power (vertical jump) | — |
| Stewart et al. [278] | — | — | Perceived soreness | Speed (40-m sprint) | — |
| Stojanovic et al. [279] | — | — | — | Strength/power (CMJ) | — |
| Su et al. [280] | — | — | — | Speed (50-m sprint) Strength/power (CMJ, isokinetic knee flexion and extension) | — |
| Sudhakar, Padmasheela [281] | — | — | — | ROM (ankle dorsiflexion and plantar flexion) Strength/power (Sergeant jump test) | — |
| Taber et al. [282] | — | — | Perceived soreness | ROM (Eli's test, straight leg raise, Thomas test, 90-90 extension test)  Strength/power (CMJ, Isometric mid-thigh pull, SJ) | — |
| Takeuchi, Tsukuda [283] | — | — | — | ROM (ankle dorsiflexion, hip flexion, knee flexion) Strength/power (vertical jump) | — |
| Taleb-Beydokhti, Haghshenas [284] | — | — | — | COD (Illinois Agility Test) | — |
| Tammam, Hashem [285] | — | — | — | ROM (shoulder and wrist flexibility test, sit and reach, trunk rotation test) Strength/power (rotational power ball throw, seated medicine ball  throw, vertical jump) | — |
| Taylor et al. [286] | — | — | — | Speed (20-m sprint) Strength/power (CMJ) | — |
| Toft et al. [287] | — | Passive tension | — | ROM (ankle dorsiflexion and plantar flexion) | — |
| Torres et al. [288] | — | — | — | Strength/power (isometric bench press, lateral medicine ball throw, overhead medicine ball throw, 30% of 1RM bench throw) | — |
| Tsolakis et al. [289] | — | — | — | Fencing-specific test (Time and power of lunge and shuttle run test) ROM (sit and reach) Strength/power (CMJ, drop jump, SJ) | — |
| Turki et al. [290] | — | — | — | Speed (10-m and 20-m sprints) | — |
| Turki et al. [291] | — | — | — | COD (half-T-Test) | — |
| Turna et al. [292] | — | — | — | Archery-specific test (score in arrow shooting test) | — |
| Unick et al. [293] | — | — | — | Strength/power (vertical jump) | — |
| Valdivia et al. [294] | — | — | — | ROM (sit and reach, stand and reach, trunk extension) Strength/power (Squat 1RM, vertical jump) | — |
| Van Gelder, Bartz [295] | — | — | — | COD (505 test) | — |
| Van Zyl et al. [296] | — | — | — | ROM (distance from anterior-superior iliac spine of the rear leg to the floor) | — |
| Vasconcellos et al. [297] | — | — | — | Strength/power (vertical jump) | — |
| Vasconcellos et al. [298] | — | — | — | Strength/power (vertical jump) | — |
| Veevo et al. [299] | — | Muscle elasticity Muscle stiffness Muscle tone | — | Strength/power (elbow flexion MVC) | — |
| Velasque et al. [300] | — | — | — | Endurance (Yo-Yo IR2) Strength/power (Wingate) | — |
| Walker et al. [301] | — | — | — | ROM (Trunk lateral flexion and axial rotation) | — |
| Wallmann et al. [302] | — | — | — | COD (T-test) | — |
| Walsh [303] | — | — | — | Proprioception (knee joint position sense). Strength/power (isokinetic knee flexion and extension) | — |
| Werstein, Lund [304] | — | — | — | Strength/power (drop jump) | — |
| West et al. [305] | Heart rate Plasma lactate Serum cortisol, interleukin-6, tumour necrosis factor alpha VO_2_max | — | Mood state | Strength/power (Wingate) | — |
| Williams et al. [306] | — | Kinematics of the scapula Muscle length (pectoralis minor) | — | — | — |
| Wilson et al. [307] | Energy expenditure Heart rate | — | RPE | Endurance (distance in performance run) ROM (sit and reach) | — |
| Winchester et al. [308] | — | — | — | Speed (40-m sprint) | — |
| Wong et al. [309] | — | — | — | Speed endurance (9x30-m sprints) | — |
| Yamaguchi et al. [310] | Running economy | — | — | Endurance (time to exhaustion and total running distance) | — |
| Yamaguchi et al. [311] | Heart rate Oxygen uptake | — | RPE | Endurance (time to exhaustion at 90% VO_2_max) | — |
| Yamaguchi et al. [312] | Heart rate Oxygen uptake | — | — | Endurance (time to exhaustion at 90% VO_2_max) | — |
| Yaşli, Müniroğlu [313] | — | — | — | ROM (sit and reach) Strength/power (horizontal jump, vertical jump) | — |
| Yildiz [314] | — | — | — | COD (t test) ROM (sit and reach) Speed (10-m and 30-m sprint) Strength/power (CMJ, horizontal jump, SJ) | — |
| Yıldırım et al. [315] | Heart rate | — | — | — | — |
| Young et al. [316] | — | Kinematics during drop punt kicks into a net | — | ROM (modified Thomas test) | — |
| Zakas et al. [317] | — | — | — | ROM (ankle dorsiflexion, hip flexion, extension and abduction, knee flexion) | — |
| Zakas [318] | — | — | — | ROM (ankle dorsiflexion, hip flexion, extension and abduction, knee flexion) | — |
| Zakas et al. [319] | — | — | — | ROM (knee flexion) Strength/power (Isokinetic knee extension) | — |
| Zakas et al. [320] | — | — | — | ROM (knee flexion) Strength/power (Isokinetic knee extension) | — |
| Zakas et al. [321] | — | — | — | ROM (knee flexion) Strength/power (Isokinetic knee extension) | — |
| Zakas et al. [322] | — | — | — | ROM (ankle dorsiflexion, hip abduction, flexion and extension, knee flexion, trunk flexion) | — |
| Zmijewski et al. [323] | — | — | — | ROM (sit and reach) Speed endurance (5x6 s on cycle ergometer) | — |
| Zourdos et al. [324] | VO_2_ | — | — | Endurance (distance covered running) ROM (sit and reach) | — |
| 孙勇 [325] | Blood lactate VO_2_max | — | — | — | — |

*Legend (ordered alphabetically)*: CK – Creatine kinase. CMJ – Countermovement jump. COD – Change of direction. PNF – Proprioceptive neuromuscular facilitation. ROM – Range of motion. SJ – Squat jump. RPE – Rate of perceived exertion. VO_2_ – Volume of oxygen.

***ESM 2.5. Special analysis of trials reporting injury data***

The five trials assessing injury data (all with a randomized design) were published between 2006 [141] and 2022 [270], with sample sizes ranging from 14 [64] to 220 [141] (mean 102.6 ± 80.4, median 113, IQR 28 to 172). In four trials [53, 64, 141, 270], only male subjects were analyzed, while sex was unreported in the remaining trial [70]. Static active stretching [70, 141, 270], static passive stretching [53, 64] and PNF (rhythmic stabilization) [64] were implemented, and non-stretching comparators involved passive controls [53, 70], isometric training [270], and eccentric strength training [141]. Interventions were implemented across 8 [70] to ~21 weeks [270] (mean 13.8 ± 4.9, median 12, IQR 10 to 18.5), and stretched the lower limbs [53, 64, 70], lower limbs and trunk [141], or upper limbs [270]. Three trials provided sufficient information to assess the minimum stretching volume per session: 100 s [70], 270 s [141], and 300 s [270]. One trial applied 1200 s of PNF per session, but unclear volume was applied in the static passive stretching group [64]. Interestingly, all the trials implemented stretching post-exercise [64, 70, 141], post-exercise plus independent sessions (*i.e.*, outside of the main training sessions) [53], or at night, before going to bed [270]. Therefore, no evidence is available from trials implementing stretching during the warm-up or intra-session stretching in general. The interventions were implemented in the pre-season [270], competitive season [53, 70], or both [141] (one was unreported [64]).

In the trial by Azuma, Someya [53], the RR was not significantly different between the groups during the intervention period (12 weeks). There was a RR difference at a 40-week follow-up, but since this was observed 28 weeks post-intervention, it is unlikely that differences in the intervention could have explained it. Of note, the two groups were not volume-equated, as the intervention group performed the stretching protocol in addition to the regular training that both groups were performing. The trial by Bello et al. [64] compared PNF to static passive stretching and found no significant between-group differences in the number of muscular and/or joint injuries. The trial by Bouthin, Edouard [70] also showed no effects of post-exercise stretching in reducing injury risk in comparison with passive controls. Comparing stretching to eccentric strength training, Gabbe et al. [141] found no evidence that stretching was superior in reducing injury risk, despite having reported poorer compliance in the eccentric training group. Finally, Shitara et al. [270] compared a stretching group to an isometric training group, and the injury rate was significantly smaller in the isometric training group. Therefore, from the available research, there is no evidence to suggest that stretching reduces injury risk in athletes, but with only 5 trials available, this is a tentative statement.

**References**

1. Higgins JP, Thomas J, Chandler J, Cumpston M, Li T, Page MJ, Welch V. Cochrane Handbook for Systematic Reviews of Interventions. 2nd ed. Chichester (UK): John Wiley & Sons; 2019.

2. Gogovor A, Zomahoun HTV, Ekanmian G, Adisso ÉL, Deom Tardif A, Khadhraoui L, et al. Sex and gender considerations in reporting guidelines for health research: a systematic review. Biology of Sex Differences. 2021;12(1):62. doi: 10.1186/s13293-021-00404-0.

3. Clayton JA, Tannenbaum C. Reporting Sex, Gender, or Both in Clinical Research? JAMA. 2016;316(18):1863-4. doi: 10.1001/jama.2016.16405.

4. McKay AKA, Stellingwerff T, Smith ES, Martin DT, Mujika I, Goosey-Tolfrey VL, et al. Defining Training and Performance Caliber: A Participant Classification Framework. Int J Sports Physiol Perform. 2022;17(2):317-31. doi: <https://doi.org/10.1123/ijspp.2021-0451>.

5. Rambaud AJM, Ardern CL, Thoreux P, Regnaux J-P, Edouard P. Criteria for return to running after anterior cruciate ligament reconstruction: a scoping review. Br J Sports Med. 2018;52(22):1437. doi: <https://doi.org/10.1136/bjsports-2017-098602>.

6. Behm DG. The science and physiology of flexibility and stretching. Implications and applications in sport performance and health. Oxon: Routledge; 2019.

7. Egan AD, Cramer JT, Massey LL, Marek SM. Acute effects of static stretching on peak torque and mean power output in National Collegiate Athletic Association Division I Women's Basketball players. J Strength Cond Res. 2006;20(4):778-82. doi: <https://doi.org/10.1519/R-18575.1>.

8. Sekir U, Arabaci R, Akova B. Acute effects of static stretching on peak and end-range hamstring-to-quadriceps functional ratios. World J Orthop. 2015;6(9):719-26. doi: <https://doi.org/10.5312/wjo.v6.i9.719>.

9. Oyama S, Goerger CP, Goerger BM, Lephart SM, Joseph BM. Effects of Non-Assisted Posterior Shoulder Stretches on Shoulder Range of Motion Among Collegiate Baseball Pitchers. Athlet Train Sports Health Care: J Practicing Clin. 2010;2(4):163-70. doi: <https://doi.org/10.3928/19425864-20100524-01>.

10. Ohshita T, Mitsuzono R. Influence of different stretching on range of motion and running economy in long distance runners. Jpn J Phys Fit Sports Med. 2009;58(3):395-404. doi: <https://doi.org/10.7600/jspfsm.58.395>.

11. Balci A, Unuvar E, Akinoglu B, Kocahan T. The effect of different neural mobilization exercises on hamstring flexibility and functional flexibility in wrestlers. J Exerc Rehabil. 2020;16(6):503-9. doi: <https://doi.org/10.12965/jer.2040700.350>.

12. Mendez-Sanchez R, Alburquerque-Sendin F, Fernandez-de-las-Penas C, Barbero-Iglesias FJ, Sanchez-Sanchez C, Calvo-Arenillas JI, Huijbregts P. Immediate Effects of Adding a Sciatic Nerve Slider Technique on Lumbar and Lower Quadrant Mobility in Soccer Players: A Pilot Study. J Altern Complement Med. 2010;16(6):669-75. doi: <https://doi.org/10.1089/acm.2009.0403>.

13. Satkunskiene D, Ardekani MMZ, Khair RM, Kutraite G, Venckuniene K, Snieckus A, Kamandulis S. Warm-up and Hamstring Stiffness, Stress-Relaxation, Flexibility and Knee Proprioception in Young Soccer Players. J Athl Train. 2022;57(5):485-93. doi: <https://doi.org/10.4085/1062-6050-0416.20>.

14. Sauers E, August A, Snyder A. Fauls stretching routine produces acute gains in throwing shoulder mobility in collegiate baseball players. J Sport Rehabil. 2007;16(1):28-40. doi: <https://doi.org/10.1123/jsr.16.1.28>.

15. Sheppard JM, Young WB. Agility literature review: classifications, training and testing. J Sports Sci. 2006;24(9):919-32. doi: <https://doi.org/10.1080/02640410500457109>.

16. Mota T, Afonso J, Sá M, Clemente FM. An Agility Training Continuum for Team Sports: From Cones and Ladders to Small-Sided Games. Strength Cond J. 2022;44(1). doi: <https://doi.org/10.1519/SSC.0000000000000653>.

17. Jeffreys I. A Task-Based Approach to Developing Context-Specific Agility. Strength Cond J. 2011;33(4). doi: <https://doi.org/10.1519/SSC.0b013e318222932a>.

18. Abadi FA, Avandi SM, Aminian-Far A. Acute effect of different warm up protocols on static and dynamic balance indices and balance the vault in skilled female gymnast. Koomesh. 2015;17(1):99-110. doi: No DOI available.

19. Agopyan A, Bozdogan FS, Tekin D, Yetgin MK, Guler CG. Acute effects of static stretching exercises on short-distance flutter kicking time in child swimmers. Int J Perform Anal Sport. 2012;12(3):484-97. doi: <https://doi.org/10.1080/24748668.2012.11868613>.

20. Ahmadabadi F, Avandi SM, Aminian-Far A. Correlation between sway parameters of center of pressure in static and dynamic balance indices with eyes closed after four weeks static warm up in skilled gymnast athletes. Koomesh. 2017;19(1):102-12. doi: No DOI available.

21. Akarsu M, Kurhan CO, İlbak İ, Altuntop R, Gönç M, Stojanović S, Purenovic-Ivanović T. Acute Effects of Self-Myofascial Release Through Foam Roller and Static Stretching Methods on Vertical Jump Performance of Taekwondo Players. J Pharm Negat Results. 2022;13:1630-4. doi: <https://doi.org/10.47750/pnr.2022.13.S06.215>.

22. Akehurst H, Grice JE, Angioi M, Morrissey D, Migliorini F, Maffulli N. Whole-body vibration decreases delayed onset muscle soreness following eccentric exercise in elite hockey players: a randomised controlled trial. J Orthop Surg Res. 2021;16(1):Article 589. doi: <https://doi.org/10.1186/s13018-021-02760-4>.

23. Alipasali F, Papadopoulou SD, Gissis I, Komsis G, Komsis S, Kyranoudis A, et al. The Effect of Static and Dynamic Stretching Exercises on Sprint Ability of Recreational Male Volleyball Players. Int J Environ Res Public Health. 2019;16(16):Article 2835. doi: <https://doi.org/10.3390/ijerph16162835>.

24. Alipasali F, Papadopoulou SD, Kyranoudis AE, Gisis I. The effect of static and dynamic stretching exercises on the jumping ability of recreational male volleyball players. J Phys Educ Sport. 2022;22(4):1026-32. doi: <https://doi.org/10.7752/jpes.2022.04130>.

25. Almeida Júnior H, Bastos AA, Martins FJA, De Souza RF, Martins COD, Da Silva AG, et al. Effect of the global active stretching (SGA®) for restoring the normal values of thermal asymmetry. J Phys Educ Sport. 2019;19:1453-9. doi: <https://doi.org/10.7752/jpes.2019.s4211>.

26. Almeida Júnior H, De Souza RF, Aidar FJ, Da Silva AG, Regi RP, Bastos AA. Global Active Stretching (SGA®) Practice for Judo Practitioners' Physical Performance Enhancement. Int J Exerc Sci. 2018;11(6):364-74. doi: No DOI available.

27. Alp M. Acute effects of static and dynamic stretching exercises on isokinetic strength of hip flexion-extension in male handball players. Prog Nutr. 2020;22(1):161-6. doi: <https://doi.org/10.23751/pn.v22i1.9209>.

28. Alp M, Catikkas F, Kurt C. Acute effects of static and dynamic stretching exercises on lower extremity isokinetic strength in taekwondo athletes. Isokinet Exerc Sci. 2018;26(4):307-11. doi: <https://doi.org/10.3233/ies-183159>.

29. Amir Vazini T, Parnow A. Level of functional capacities following soccer-specific warm-up methods among elite collegiate soccer players. J Sports Med Phys Fit. 2017;57(5):537-42. doi: <https://doi.org/10.23736/s0022-4707.16.06236-8>.

30. Amiri-Khorasani M. Kinematics Analysis: The Acute Effect of Different Stretching Methods on Dynamic Range of Motion of Lower Extremity Joints during Soccer Instep Kicking. Int J Perform Anal Sport. 2013;13(1):190-9. doi: <https://doi.org/10.1080/24748668.2013.11868641>.

31. Amiri-Khorasani M. Acute effects of different stretching methods on static and dynamic balance in female football players. Int J Ther Rehabil. 2015;22(2):68-73. doi: <https://doi.org/10.12968/ijtr.2015.22.2.68>.

32. Amiri-Khorasani M, Ferdinands RED. The acute effect of stretching on the kinematics of instep kicking in soccer. Sports Technol. 2014;7(1-2):69-78. doi: <https://doi.org/10.1080/19346182.2014.893348>.

33. Amiri-Khorasani M, Kellis E. Static vs. Dynamic Acute Stretching Effect on Quadriceps Muscle Activity during Soccer Instep Kicking. J Hum Kinet. 2013;39(1):37-47. doi: <https://doi.org/10.2478/hukin-2013-0066>.

34. Amiri-Khorasani M, Sotoodeh V. The acute effects of combined static and dynamic stretch protocols on fitness performances in soccer players. J Sports Med Phys Fit. 2013;53(5):559-65. doi: No DOI available.

35. Amiri-Khorasani M, Abu Osman NA, Yusof A. Electromyography Assessments of the Vastus Medialis Muscle during Soccer Instep Kicking between Dynamic and Static Stretching. J Hum Kinet. 2010;24:35-41. doi: <https://doi.org/10.2478/v10078-010-0017-2>.

36. Amiri-Khorasani M, Calleja-Gonzalez J, Mogharabi-Manzari M. Acute Effect of Different Combined Stretching Methods on Acceleration and Speed in Soccer Players. J Hum Kinet. 2016;50(1):179-86. doi: <https://doi.org/10.1515/hukin-2015-0154>.

37. Amiri-Khorasani M, Abu Osman NA, Yusof A. Acute effect of static and dynamic stretching on hip dynamic range of motion during instep kicking in professional soccer players. J Strength Cond Res. 2011;25(6):1647-52. doi: <https://doi.org/10.1519/JSC.0b013e3181db9f41>.

38. Amiri-Khorasani M, Mohammadkazemi R, Sarafrazi S, Riyahi-Malayeri S, Sotoodeh V. Kinematics analyses related to stretch-shortening cycle during soccer instep kicking after different acute stretching. J Strength Cond Res. 2012;26(11):3010-7. doi: <https://doi.org/10.1519/JSC.0b013e3182443442>.

39. Amiri-Khorasani M, Sahebozamani M, Tabrizi KG, Yusof AB. Acute effect of different stretching methods on Illinois Agility Test in soccer players. J Strength Cond Res. 2010;24(10):2698-704. doi: <https://doi.org/10.1519/JSC.0b013e3181bf049c>.

40. Andre MJ, Fry AC, McLellan E, Weiss LW, Moore CM. Acute Effects of Static Stretching on Bench Press Power and Velocity in Adolescent Male Athletes. Int J Sports Sci Coach. 2014;9(5):1145-52. doi: <https://doi.org/10.1260/1747-9541.9.5.1145>.

41. Andrejić O, Tošić S, Knežević O. Acute effects of low- and high-volume stretching on fitness performance in young basketball players. Serb J Sports Sci. 2012(1):11-6. doi: No DOI available.

42. Annino G, Ruscello B, Lebone P, Palapalazzo F, Lombardo M, Paduapadua E, et al. Acute effects of static and dynamic stretching on jump performance after 15 min of reconditioning shooting phase in basketball players. J Sports Med Phys Fit. 2017;57(4):330-7. doi: <https://doi.org/10.23736/s0022-4707.16.06143-0>.

43. Ari Y. Effects of different stretching methods on speed, jump, flexibility and upper extremity performance in wrestlers. Kinesiol Slov. 2021;27(1):162-76. doi: <https://doi.org/10.52165/kinsi.27.1.162-176>.

44. Arihiro H, Miku O, Katsuaki S, Norikazu YAO, Kanae I, Hideyuki K. Pilates Exercise Improves Hip Joint Flexion Mobility in Rugby Players. Advances in Exercise & Sports Physiology. 2018;24(3):45-9. doi: No DOI available.

45. Avaz MA, Saghebjoo M, Zardast M, Ilbeigi S. Acute effects of proprioception, massage and dynamic stretching warm up protocols on serum CK and LDH activity levels after one session of plyometric training in male volleyball players. Koomesh. 2016;17(2):393-402. doi: No DOI available.

46. Avedesian JM, Judge LW, Wang H, Dickin DC. The biomechanical effect of warm-up stretching strategies on landing mechanics in female volleyball athletes. Sports Biomech. 2020;19(5):587-600. doi: <https://doi.org/10.1080/14763141.2018.1503322>.

47. Avloniti A, Chatzinikolaou A, Fatouros IG, Avloniti C, Protopapa M, Draganidis D, et al. The acute effects of static stretching on speed and agility performance depend on stretch duration and conditioning level. J Strength Cond Res. 2016;30(10):2767-73. doi: <https://doi.org/10.1519/jsc.0000000000000568>.

48. Avloniti A, Chatzinikolaou A, Fatouros IG, Protopapa M, Athanailidis I, Avloniti C, et al. The effects of static stretching on speed and agility: One or multiple repetition protocols? Eur J Sport Sci. 2016;16(4):402-8. doi: <https://doi.org/10.1080/17461391.2015.1028467>.

49. Ayala F, De Baranda PS. Acute effect of stretching on sprint in honour division soccer players. RICYDE. 2010;6(18):1-12. doi: <https://doi.org/10.11138/mltj/2018.8.1.037>.

50. De Baranda PS, Ayala F. Efecto agudo del estiramiento sobre la agilidad y coordinación de movimientos rápidos en jugadores de fútbol de División de Honor [The Acute effect of stretching on the agility and coordination in fast movements of first division football players]. Kronos. 2010;9(17):21-8. doi: No DOI available.

51. Ayala F, De Baranda PS, De Ste Croix M. Effect of active stretch on hip flexion range of motion in female professional futsal players. J Sports Med Phys Fit. 2010;50(4):428-35. doi: No DOI available.

52. Aydoǧ ST, Hazir M, Hasçelik Z, Çaǧlar A, Özdoǧan M. The effect of stretching exercise on maximum peak torque. J Rheumatol Med Rehabil. 2000;11(4):278-81. doi: No DOI available.

53. Azuma N, Someya F. Injury prevention effects of stretching exercise intervention by physical therapists in male high school soccer players. Scand J Med Sci Sports. 2020;30(11):2178-92. doi: <https://doi.org/10.1111/sms.13777>.

54. Babbar S, Zutshi K, Munjal J. Acute Effect of a Dynamic and Static Stretching Exercise Bout During Warm up on Power in Soccer Players. Indian J Physiother Occup Ther. 2011;5(2):141-3. doi: No DOI available.

55. Bali S, Guru K. Comparative effect of static stretching and mulligan stretching on hip adductor flexibility in footballers: a two group trial. JK Science. 2020;22(2):96-100. doi: No DOI available.

56. Barbosa GM, Dantas GAF, Pinheiro SM, Rego JTP, Oliveira TLC, Silva KKF, et al. Acute effects of stretching and/or warm-up on neuromuscular performance of volleyball athletes: a randomized cross-over clinical trial. Sport Sci Health. 2020;16(1):85-92. doi: <https://doi.org/10.1007/s11332-019-00576-8>.

57. Baumgart C, Gokeler A, Donath L, Hoppe MW, Freiwald J. Effects of Static Stretching and Playing Soccer on Knee Laxity. Clin J Sport Med. 2015;25(6):541-5. doi: <https://doi.org/10.1097/jsm.0000000000000174>.

58. Bazett-Jones DM, Gibson MH, McBride JM. Sprint and vertical jump performances are not affected by six weeks of static hamstring stretching. J Strength Cond Res. 2008;22(1):25-31. doi: <https://doi.org/10.1519/JSC.0b013e31815f99a4>.

59. Bazett-Jones DM, Winchester JB, McBride JM. Effect of potentiation and stretching on maximal force, rate of force development, and range of motion. J Strength Cond Res. 2005;19(2):421-6. doi: <https://doi.org/10.1519/14193.1>.

60. Beckett JRJ, Schneiker KT, Wallman KE, Dawson BT, Guelfi KJ. Effects of Static Stretching on Repeated Sprint and Change of Direction Performance. Med Sci Sports Exerc. 2009;41(2):444-50. doi: <https://doi.org/10.1249/MSS.0b013e3181867b95>.

61. Behara B, Jacobson BH. Acute Effects of Deep Tissue Foam Rolling and Dynamic Stretching on Muscular Strength, Power, and Flexibility in Division I Linemen. J Strength Cond Res. 2017;31(4):888-92. doi: <https://doi.org/10.1519/jsc.0000000000001051>.

62. Belkhiria-Turki L, Chaouachi A, Turki O, Chtourou H, Chtara M, Chamari K, et al. Eight weeks of dynamic stretching during warm-ups improves jump power but not repeated or single sprint performance. Eur J Sport Sci. 2014;14(1):19-27. doi: <https://doi.org/10.1080/17461391.2012.726651>.

63. Belkhiria-Turki L, Chaouachi A, Turki O, Hammami R, Chtara M, Amri M, et al. Greater volumes of static and dynamic stretching within a warm-up do not impair star excursion balance performance. J Sports Med Phys Fit. 2014;54(3):279-88. doi: No DOI available.

64. Bello M, Mesiano Maifrino LB, Gama EF, Rodrigues de Souza R. Rhythmic stabilization versus conventional passive stretching to prevent injuries in indoor soccer athletes: A controlled clinical trial. J Bodyw Mov Ther. 2011;15(3):380-3. doi: <https://doi.org/10.1016/j.jbmt.2010.11.002>.

65. Ben Maaouia G, Nassib S, Negra Y, Chammari K, Souissi N. Agility performance variation from morning to evening: dynamic stretching warm-up impacts performance and its diurnal amplitude. Biol Rhythm Res. 2020;51(4):509-21. doi: <https://doi.org/10.1080/09291016.2018.1537553>.

66. Bingul BM, Son M, Aydin M, Gelen E, Cinel Y, Bulgan C. The effects of static and dynamic stretching on agility performance. J Phys Educ Sports Sci. 2014;8(1):43-8. doi: No DOI available.

67. Bishop D, Middleton G. Effects of static stretching following a dynamic warm-up on speed, agility and power. J Hum Sport Exerc. 2013;8(2 Suppl):391-400. doi: <https://doi.org/10.4100/jhse.2012.82.07>.

68. Bogdanis GC, Donti O, Tsolakis C, Smilios I, Bishop DJ. Intermittent but not continuous static stretching improves subsequent vertical jump performance in flexibility-trained athletes. J Strength Cond Res. 2019;33(1):203-10. doi: <https://doi.org/10.1519/jsc.0000000000001870>.

69. Boudenot A, Jaffre C, Portier H. Comparaison de quatre méthodes d'échauffement sur la performance lors du Wingate test [Comparing four warm-up procedures on Wingate test performance]. Kinesitherapie. 2014;14(146):34-8. doi: <https://doi.org/10.1016/j.kine.2013.10.017>.

70. Bouthin B, Edouard P. Les étirements sont-ils un facteur préventif des lésions de l’appareil locomoteur ? Étude pilote prospective dans une population de footballeurs amateurs [Is stretching a good strategy to prevent musculo-skeletal injuries? A prospective pilot study on football amateur players]. J Traumatol du Sport. 2015;32(1):22-8. doi: <https://doi.org/10.1016/j.jts.2015.01.001>.

71. Brodowicz GR, Welsh R, Wallis J. Comparison of stretching with ice, stretching with heat, or stretching alone on hamstring flexibility. J Athl Train. 1996;31(4):324-7. doi: No DOI available.

72. Burkett LN, Phillips WT, Ziuraitis J. The best warm-up for the vertical jump in college-age athletic men. J Strength Cond Res. 2005;19(3):673-6. doi: <https://doi.org/10.1519/15204.1>.

73. Burkett LN, Ziuraitis J, Phillips WT. The effect of four different warm-ups on the maximum vertical jump test scores for female college athletes. Women Sport Phys Act J. 2001;10(2):83-93. doi: <https://doi.org/10.1123/wspaj.10.2.83>.

74. Buttifant D, Hrysomallis C. Effect of various practical warm-up protocols on acute lower-body power. J Strength Cond Res. 2015;29(3):656-60. doi: <https://doi.org/10.1519/jsc.0000000000000690>.

75. Caliskan E, Akkoc O, Bayramoglu Z, Gozubuyuk OB, Kural D, Azamat S, Adaletli I. Effects of static stretching duration on muscle stiffness and blood flow in the rectus femoris in adolescents. Med Ultrason. 2019;21(2):136-43. doi: <https://doi.org/10.11152/mu-1859>.

76. Caplan N, Rogers R, Parr MK, Hayes PR. The effect of proprioceptive neuromuscular facilitation and static stretch training on running mechanics. J Strength Cond Res. 2009;23(4):1175-80. doi: <https://doi.org/10.1519/JSC.0b013e318199d6f6>.

77. Carvalho FLP, Carvalho MC, Simão R, Gomes TM, Costa PB, Neto LB, et al. Acute effects of a warm-up including active, passive, and dynamic stretching on vertical jump performance. J Strength Cond Res. 2012;26(9):2447-52. doi: <https://doi.org/10.1519/JSC.0b013e31823f2b36>.

78. Carvalho FLP, Rayol Prati JEL, Alencar Carvalho MCG, Dantas EHM. Efectos agudos del estiramiento estático y de la facilitación neuromuscular proprioceptiva en el desempeño del salto vertical de tenistas adolescentes [Acute effects of static stretching and proprioceptive neuromuscular facilitation on the performance of vertical jump in adolescent tennis players]. Fit Perform J Online. 2009;8(4):264-8. doi: <https://doi.org/10.3900/fpj.8.4.264.e>.

79. Celik A. Acute effects of cyclic versus static stretching on shoulder flexibility, strength, and spike speed in volleyball players. Turk J Phys Med Rehabil. 2017;63(2):124-32. doi: <https://doi.org/10.5606/tftrd.2017.198>.

80. César EP, Junior CSR, Francisco RN. Effects of 2 Intersection Strategies for Physical Recovery in Jiu-Jitsu Athletes. Int J Sports Physiol Perform. 2021;16(4):585-90. doi: <https://doi.org/10.1123/ijspp.2019-0701>.

81. Cetin O, Isik O, Yasar MN. The acute effects of a dynamic warm-up including hip mobility exercises on sprint, agility and vertical jump performance. Eur J Hum Mov. 2020;45. doi: <https://doi.org/10.21134/eurjhm.2020.45.6>.

82. Chaouachi A, Castagna C, Chtara M, Brughelli M, Turki O, Galy O, et al. Effect of warm-ups involving static or dynamic stretching on agility, sprinting, and jumping performance in trained individuals. J Strength Cond Res. 2010;24(8):2001-11. doi: <https://doi.org/10.1519/JSC.0b013e3181aeb181>.

83. Chaouachi A, Padulo J, Kasmi S, Ben Othmen A, Chatra M, Behm DG. Unilateral static and dynamic hamstrings stretching increases contralateral hip flexion range of motion. Clin Physiol Funct Imaging. 2017;37(1):23-9. doi: <https://doi.org/10.1111/cpf.12263>.

84. Chatzopoulos D, Doganis G, Lykesas G, Koutlianos N, Galazoulas C, Bassa E. Effects of static and dynamic stretching on force sense, dynamic flexibility and reaction time of children. Open Sports Sci J. 2019;12(1):22-7. doi: <https://doi.org/10.2174/1875399X01912010022>.

85. Chatzopoulos D, Galazoulas C, Patikas D, Kotzamanidis C. Acute Effects of Static and Dynamic Stretching on Balance, Agility, Reaction Time and Movement Time. J Sports Sci Med. 2014;13(2):403-9. doi: No DOI available.

86. Chatzopoulos D, Kapodistria L, Doganis G, Messaritakis V, Lykesas G. Effects of varying volumes of dynamic stretching on active range of motion, reaction time, and movement time in female soccer players. J Exerc Physiol Online. 2019;22(5):147-56. doi: No DOI available.

87. Chen CH, Ye X, Wang YT, Chen YS, Tseng WC. Differential effects of different warm-up protocols on repeated sprints-induced muscle damage. J Strength Cond Res. 2019;32(11):3276-84. doi: <https://doi.org/10.1519/JSC.0000000000002310>.

88. Chen YL, Tsai FM, Hsu WC, Yang CJ, Yei TY. Exploring Shank Circumference by Stretching after Training among Volleyball Players. Int J Environ Res Public Health. 2021;18(16):Article 8849. doi: <https://doi.org/10.3390/ijerph18168849>.

89. Chinnavan E, Gopaladhas S, Kaikondan P. Effectiveness of Pilates training in improving hamstring flexibility of football players. Bangladesh J Med Sci. 2015;14(3):265-9. doi: <https://doi.org/10.3329/bjms.v14i3.16322>.

90. Christensen B, Nordstrom BJ. The effects of proprioceptive neuromuscular facilitation and dynamic stretching techniques on vertical jump performance. J Strength Cond Res. 2008;22(6):1826-31. doi: <https://doi.org/10.1519/JSC.0b013e31817ae316>.

91. Christensen B, Bond CW, Napoli R, Lopez K, Miller J, Hackney KJ. The effect of static stretching, mini-band warm-ups, medicine-ball warm-ups, and a light jogging warm-up on common athletic ability tests. Int J Exerc Sci. 2020;13(4):298-311. doi: No DOI available.

92. Chtourou H, Aloui A, Hammouda O, Chaouachi A, Chamari K, Souissi N. Effect of Static and Dynamic Stretching on the Diurnal Variations of Jump Performance in Soccer Players. PLoS One. 2013;8(8):Article e70534. doi: <https://doi.org/10.1371/journal.pone.0070534>.

93. Church JB, Wiggins MS, Moode FM, Crist R. Effect of warm-up and flexibility treatments on vertical jump performance. J Strength Cond Res. 2001;15(3):332-6. doi: No DOI available.

94. Colak S. Effects of dynamic stretches on isokinetic hamstring and quadriceps femoris muscle strength in elite female soccer players. S African J Res Sport Phys Ed Recreat. 2012;34(2):15-25. doi: No DOI available.

95. Coons JM, Gould CE, Kim JK, Farley RS, Caputo JL. Dynamic stretching is effective as static stretching at increasing flexibility. J Hum Sport Exerc. 2017;12(4):1153-61. doi: <https://doi.org/10.14198/jhse.2017.124.02>.

96. Correia JDB, Martins GA, da Silva LG, Silveira EM, Steffens T, Pietta-Dias C, Cadore EL. Comparação dos efeitos agudos dos métodos FNP e estabilização (FIFA 11+) no equilíbrio dinâmico de atletas de futebol universitário [Comparison of the acute effects of the PNF and stabilization methods (FIFA 11+) on the dynamic balance of university football athletes]. Rev Bras Futsal Futeb. 2021;13(53):234-43. doi: No DOI available.

97. Cwirlej-Sozanska AB, Wójcik O, Wójcik J, Mól M, Kolasa T. The influence of static stretching of specific lower limb muscle groups on the jump height parameter of volleyball players aged 16-17: A pilot study. Adv Rehabil. 2021;35(1). doi: <https://doi.org/10.5114/AREH.2021.102315>.

98. da Silva FF, de Souza RA, do Couto CLB, Magalhães RF, Kawatake JP. Efeitos agudos do alongamento estático no rendimento de testes funcionais em atletas de voleibol e futebol [Acute effects of static stretching on functional performance tests on volleyball and soccer athletes]. Rev Terapia Manual. 2011;9(42):138-42. doi: No DOI available.

99. Dalamitros AA, Vagios A, Toubekis AG, Tsalis G, Clemente-Suarez VJ, Manou V. The effect of two additional dry-land active warm-up protocols on the 50-m front-crawl swimming performance. Hum Mov. 2018;19(3):75-81. doi: <https://doi.org/10.5114/hm.2018.76082>.

100. Dallas G, Smirniotou A, Tsiganos G, Tsopani D, Di Cagno A, Tsolakis CH. Acute effect of different stretching methods on flexibility and jumping performance in competitive artistic gymnasts. J Sports Med Phys Fit. 2014;54(6):683-90. doi: No DOI available.

101. Dalrymple KJ, Davis SE, Dwyer GB, Moir GL. Effect of static and dynamic stretching on vertical jump performance in collegiate women volleyball players. J Strength Cond Res. 2010;24(1):149-55. doi: <https://doi.org/10.1519/JSC.0b013e3181b29614>.

102. Damasceno MV, Duarte M, Pasqua LA, Lima-Silva AE, MacIntosh BR, Bertuzzi R. Static Stretching Alters Neuromuscular Function and Pacing Strategy, but Not Performance during a 3-Km Running Time-Trial. PLoS One. 2014;9(6):Article e99238. doi: <https://doi.org/10.1371/journal.pone.0099238>.

103. Darcadia CAF, Silva MdJ, de Souza RA, de Carvalho WRG, da Silva FF. Efeitos agudos do alongamento estático no rendimento de testes funcionais em atletas de futebol [Acute effects of static stretching on functional performance tests on soccer athletes]. Rev Terapia Manual. 2011;9(45):503-7. doi: No DOI available.

104. Dawson B, Gow S, Modra S, Bishop D, Stewart C. Effects of immediate post-game recovery procedures on muscle soreness, power and flexibility levels over the next 48 hours. J Sci Med Sport. 2005;8(2):210-21. doi: <https://doi.org/10.1016/S1440-2440(05)80012-X>.

105. de Almeida Leme JAC, Barberi RA, Curiacos KJ, Rogatto PV. Stretch influence on pre-competitive stress on juvenile soccer players. Motricidade. 2008;4(3):57-60. doi: <https://doi.org/10.6063/motricidade.271>.

106. de Castro JV, Machado KC, Scaramussa K, Gomes JLE. Incidence of Decreased Hip Range of Motion in Youth Soccer Players and Response to a Stretching Program: A Randomized Clinical Trial. J Sport Rehabil. 2013;22(2):100-7. doi: <https://doi.org/10.1123/jsr.22.2.100>.

107. De Oliveira Júnior JD, De Lima Pinto JCB, De Caldas Honorato R, De Barros ACM, Da Silva Santos TR, Mortatti AL. The acute effect of proprioceptive neuromuscular facilitation in explosive force and jump resistance of basketball players. J Phys Educ Sport. 2018;18(2):632-6. doi: <https://doi.org/10.7752/jpes.2018.02092>.

108. de Oliveira FCL, Pinto Lopes Rama LM. Alongamento estático ativo no desempenho em provas de potência e velocidade. Braz J Biomotricity. 2013;7(2):128-38. doi: No DOI available.

109. de Rezende FN, da Mota GR, Lopes CR, da Silva BVC, Simim MAM, Marocolo M. Specific warm-up exercise is the best for vertical countermovement jump in young volleyball players. Motriz. 2016;22(4):299-303. doi: <https://doi.org/10.1590/S1980-6574201600040013>.

110. Di Cagno A, Baldari C, Battaglia C, Gallotta MC, Videira M, Piazza M, Guidetti L. Preexercise static stretching effect on leaping performance in elite rhythmic gymnasts. J Strength Cond Res. 2010;24(8):1995-2000. doi: <https://doi.org/10.1519/JSC.0b013e3181e34811>.

111. Di Cagno A, Calcagno G, Buonsenso A, Iuliano E, Innocenti G, Piazza M, Fiorilli G. Effects of static and dynamic stretching on upper limb explosive, isometric and endurance strength, in male volleyball players. Ital J Anat Embryol. 2019;124(1):113-21. doi: <https://doi.org/10.13128/IJAE-25475>.

112. Donti O, Gaspari V, Papia K, Panidi I, Donti A, Bogdanis GC. Acute Effects of Intermittent and Continuous Static Stretching on Hip Flexion Angle in Athletes with Varying Flexibility Training Background. Sports (Basel). 2020;8(3):Article 28. doi: <https://doi.org/10.3390/sports8030028>.

113. Donti O, Panidis I, Terzis G, Bogdanis GC. Gastrocnemius Medialis Architectural Properties at Rest and During Stretching in Female Athletes with Different Flexibility Training Background. Sports (Basel). 2019;7(2):Article 39. doi: <https://doi.org/10.3390/sports7020039>.

114. Donti O, Papia K, Toubekis A, Donti A, Sands WA, Bogdanis GC. Flexibility training in preadolescent female athletes: Acute and long-term effects of intermittent and continuous static stretching. J Sports Sci. 2018;36(13):1453-60. doi: <https://doi.org/10.1080/02640414.2017.1397309>.

115. Drews S, Goltz C. Lässt sich durch Querdehnung ein größerer Gewinn an Beweglichkeit erzielen als durch Längsdehnung? Randomisierte kontrollierte Studie am Beispiel der Hüftgelenkadduktoren von Fußballspielern und deren Beweglichkeit in die Abduktion [Can transversal stretching yield higher mobility than longitudinal stretching?]. Manuelle Ther. 2005;9(2):59-64. doi: <https://doi.org/10.1055/s-2005-858183>.

116. Ebadi LA, Cetin E. Duration Dependent Effect of Static Stretching on Quadriceps and Hamstring Muscle Force. Sports (Basel). 2018;6(1):Article 24. doi: <https://doi.org/10.3390/sports6010024>.

117. Eken Ö, Bayer R. Acute effects of proprioceptive neuromuscular facilitation stretching, massage and combine protocols on flexibility, vertical jump and hand grip strength performance in kickboxers. Pedagogy Phys Cult Sports. 2022;26(1):4-12. doi: <https://doi.org/10.15561/26649837.2022.0101>.

118. Espi-Lopez GV, Lopez-Martinez S, Ingles M, Serra-Ano P, Aguilar-Rodriguez M. Effect of manual therapy versus proprioceptive neuromuscular facilitation in dynamic balance, mobility and flexibility in field hockey players. A randomized controlled trial. Phys Ther Sport. 2018;32:173-9. doi: <https://doi.org/10.1016/j.ptsp.2018.04.017>.

119. Evetovich TK, Cain RM, Hinnerichs KR, Engebretsen BJ, Conley DS. Interpreting normalized and nonnormalized data after acute static stretching in athletes and nonathletes. J Strength Cond Res. 2010;24(8):1988-94. doi: <https://doi.org/10.1519/JSC.0b013e3181e3132a>.

120. Faigenbaum AD, Kang J, McFarland J, Bloom JM, Magnatta J, Ratamess NA, Hoffman JR. Acute effects of different warm-up protocols on anaerobic performance in teenage athletes. Pediatr Exerc Sci. 2006;18(1):64-75. doi: <https://doi.org/10.1123/pes.18.1.64>.

121. Faigenbaum AD, McFarland JE, Kelly NA, Ratamess NA, Kang J, Hoffman JR. Influence of Recovery Time on Warm-up Effects in Male Adolescent Athletes. Pediatr Exerc Sci. 2010;22(2):266-77. doi: <https://doi.org/10.1123/pes.22.2.266>.

122. Faigenbaum AD, McFarland JE, Schwerdtman JA, Ratamess NA, Kang J, Hoffman JR. Dynamic warm-up protocols, with and without a weighted vest, and fitness performance in high school female athletes. J Athl Train. 2006;41(4):357-63. doi: No DOI available.

123. Famisis K. Acute effect of static and dynamic stretching exercise on sprint and flexibility of amateur soccer players. Phys Train. 2015:1-12. doi: No DOI available.

124. Farshidi B, Daneshjoo A, Sahebozamani M, Konrad A. Effects of static and PNF stretching on joint position sense and range of motion after a fatigue protocol in professional male soccer players. Med Sport (Roma). 2022;75(2):206-17. doi: <https://doi.org/10.23736/S0025-7826.22.04148-5>.

125. Fattahi-Bafghi A, Amiri-Khorasani M. Sustaining effect of different stretching methods on power and agility after warm-up exercise in soccer players. World Appl Sci J. 2013;21(4):520-5. doi: <https://doi.org/10.5829/idosi.wasj.2013.21.4.2242>.

126. Favero JP, Midgley AW, Bentley DJ. Effects of an Acute Bout of Static Stretching on 40 m Sprint Performance: Influence of Baseline Flexibility. Res Sports Med. 2009;17(1):50-60. doi: <https://doi.org/10.1080/15438620802678529>.

127. Feitosa Junior JVA, Solon Junior LJF, da Silva Neto LV. Alongamento estático não reduz a força de preensão manual isométrica máxima em lutadores Brasileiros de Jiu-Jitsu [Static stretching does not reduce the maximum isometric handgrip strength in Brazilian Jiu-Jitsu fighters]. Rev Bras Prescrição Fisiol Exerc. 2019;13(86):1021-5. doi: No DOI available.

128. Fernandes IA, Kawchuk G, Bhambhani Y, Gomes PSC. Does vibration counteract the static stretch-induced deficit on muscle force development? J Sci Med Sport. 2013;16(5):472-6. doi: <https://doi.org/10.1016/j.jsams.2012.11.886>.

129. Ferreira VD, Muller BC, Achour A. Efeito agudo de exercícios de alongamento estático e dinâmico na impulsão vertical de jogadores de futebol [Acute effects of static versus dynamic stretching on the vertical jump performance of soccer players]. Motriz. 2013;19(2):450-9. doi: <https://doi.org/10.1590/S1980-65742013000200022>.

130. Ferri-Caruana A, Roig-Ballester N, Romagnoli M. Effect of dynamic range of motion and static stretching techniques on flexibility, strength and jump performance in female gymnasts. Sci Gymnast J. 2020;12(1):87-100. doi: <https://doi.org/10.52165/sgj.12.1.87-100>.

131. Fletcher IM, Anness R. The acute effects of combined static and dynamic stretch protocols on fifty-meter sprint performance in track-and-field athletes. J Strength Cond Res. 2007;21(3):784-7. doi: <https://doi.org/10.1519/R-19475.1>.

132. Fletcher IM, Jones B. The effect of different warm-up stretch protocols on 20 meter sprint performance in trained rugby union players. J Strength Cond Res. 2004;18(4):885-8. doi: <https://doi.org/10.1519/14493.1>.

133. Fletcher IM, Monte-Colombo MM. An investigation into the effects of different warm-up modalities on specific motor skills related to soccer performance. J Strength Cond Res. 2010;24(8):2096-101. doi: <https://doi.org/10.1519/JSC.0b013e3181e312db>.

134. Fletcher IM, Monte-Colombo MM. An investigation into the possible physiological mechanisms associated with changes in performance related to acute responses to different preactivity stretch modalities. Appl Physiol Nutr Metab. 2010;35(1):27-34. doi: <https://doi.org/10.1139/h09-125>.

135. Forte D, Ferrara F, Altavilla G. Relationship between types of stretching and jumping in volleyball. J Phys Educ Sport. 2019;19:1859-62. doi: <https://doi.org/10.7752/jpes.2019.s5275>.

136. Frantz TL, Ruiz MD. Effects of dynamic warm-up on lower body explosiveness among collegiate baseball players. J Strength Cond Res. 2011;25(11):2985-90. doi: <https://doi.org/10.1519/JSC.0b013e31820f509b>.

137. Fredericson M, White JJ, MacMahon JM, Andriacchi TP. Quantitative analysis of the relative effectiveness of 3 iliotibial band stretches. Arch Phys Med Rehabil. 2002;83(5):589-92. doi: <https://doi.org/10.1053/apmr.2002.31606>.

138. Frikha M, Derbel MS, Chaari N, Gharbi A, Chamari K. Acute effect of stretching modalities on global coordination and kicking accuracy in 12-13 year-old soccer players. Hum Mov Sci. 2017;54:63-72. doi: <https://doi.org/10.1016/j.humov.2017.03.008>.

139. Funk DC, Swank AM, Mikla BM, Fagan TA, Farr BK. Impact of prior exercise on hamstring flexibility: A comparison of proprioceptive neuromuscular facilitation and static stretching. J Strength Cond Res. 2003;17(3):489-92. doi: No DOI available.

140. Funk DC, Swank AM, Adams KJ, Treolo D. Efficacy of moist heat pack application over static stretching on hamstring flexibility. J Strength Cond Res. 2001;15(1):123-6. doi: No DOI available.

141. Gabbe BJ, Branson R, Bennell KL. A pilot randomised controlled trial of eccentric exercise to prevent hamstring injuries in community-level Australian Football. J Sci Med Sport. 2006;9(1-2):103-9. doi: <https://doi.org/10.1016/j.jsams.2006.02.001>.

142. Galazoulas C. Effects of static stretching duration on isokinetic peak torque in basketball players in semi-professional male basketball players. J Phys Educ Sport. 2016;16:1058-63. doi: <https://doi.org/10.7752/jpes.2016.s2168>.

143. Galazoulas C. Acute effects of static and dynamic stretching on the sprint and countermovement jump of basketball players. J Phys Educ Sport. 2017;17(1):219-23. doi: <https://doi.org/10.7752/jpes.2017.01033>.

144. Galetin N, Cvetković M, Ujsasi D, Čokorilo N, Andrašić S, Lazarević M. Effects of static stretching of various durations on the vertical jump among female volleyball players. FU Phys Educ Sport. 2017;15(1):207-17. doi: <https://doi.org/10.22190/FUPES1701207G>.

145. Gao ZX, Song Y, Yu PM, Zhang Y, Li SD. Acute Effects of Different Stretching Techniques on Lower Limb Kinematics, Kinetics and Muscle Activities during Vertical Jump. J Biomimetics, Biomater Biomed Eng. 2019;40:1-15. doi: <https://doi.org/10.4028/www.scientific.net/JBBBE.40.1>.

146. Gelen E. Acute effects of different warm-up methods on sprint, slalom dribbling, and penalty kick performance in soccer players. J Strength Cond Res. 2010;24(4):950-6. doi: <https://doi.org/10.1519/JSC.0b013e3181cb703f>.

147. Gelen E, Bakici D, Yasar MN, Turgut A. Does Static Stretching Compromise Jump Performance in Diurnal Variation? Eur J Hum Mov. 2021;47:23-31. doi: <https://doi.org/10.21134/eurjhm.2021.47.3>.

148. Gelen E, Dede M, Bingul BM, Bulgan C, Aydin M. Acute effects of static stretching, dynamic exercises, and high volume upper extremity plyometric activity on tennis serve performance. J Sports Sci Med. 2012;11(4):600-5. doi: No DOI available.

149. Gergley JC. Acute effects of passive static stretching during warm-up on driver clubhead speed, distance, accuracy, and consistent ball contact in young male competitive golfers. J Strength Cond Res. 2009;23(3):863-7. doi: <https://doi.org/10.1519/JSC.0b013e3181a00c67>.

150. Gergley JC. Latent effect of passive static stretching on driver clubhead speed, distance, accuracy, and consistent ball contact in young male competitive golfers. J Strength Cond Res. 2010;24(12):3326-33. doi: <https://doi.org/10.1519/JSC.0b013e3181e725e4>.

151. Ghasemi M, Bagheri H, Olyaei G, Talebian S, Shadmehr A, Jalaei S, Kalantari KK. Effects of cyclic static stretch on fatigue recovery of triceps surae in female basketball players. Biol Sport. 2013;30(2):97-102. doi: <https://doi.org/10.5604/20831862.1044224>.

152. Gonçalves DL, Pavão TS, Dohnert MB. Efeitos agudos e crônicos de um programa de alongamento estático e dinâmico no rendimento em jovens atletas do futebol [Acute and chronic effects of a static and dynamic stretching program in the performance of young soccer athletes]. Rev Bras Med Esporte. 2013;19(4):241-6. doi: <https://doi.org/10.1590/s1517-86922013000400003>.

153. Gürses VV, Akgül MŞ. Futbolcularin isinmada uyguladiklari farkli germe yöntemlerinin dikey siçrama, sürat ve çeviklik performansina akut etkisi [Acute effects of different stretching methods during warm-up on vertical jump, speed and agility of soccer players performance]. J Phys Educ Sports Sci. 2019;17(1):178-86. doi: <https://doi.org/10.33689/spormetre.520033>.

154. Haag SJ, Wright GA, Gillette CM, Greany JF. Effects of acute static stretching of the throwing shoulder on pitching performance of National Collegiate Athletic Association Division III Baseball Players. J Strength Cond Res. 2010;24(2):452-7. doi: <https://doi.org/10.1519/JSC.0b013e3181c06d9c>.

155. Haddad M, Dridi A, Chtara M, Chaouachi A, Wong DP, Behm D, Chamari K. Static stretching can impair explosive performance for at least 24 hours. J Strength Cond Res. 2014;28(1):140-6. doi: <https://doi.org/10.1519/JSC.0b013e3182964836>.

156. Haddad M, Prince MS, Zarrouk N, Tabben M, Behm DG, Chamari K. Dynamic stretching alone can impair slower velocity isokinetic performance of young male handball players for at least 24 hours. PLoS One. 2019;14(1):Article e0210318. doi: <https://doi.org/10.1371/journal.pone.0210318>.

157. Han G, Lee M, Cho B. Effects of Dynamic Stretch Training on Lower Extremity Power Performance of Young Sprinters. J Phys Ther Sci. 2011;23(3):401-4. doi: <https://doi.org/10.1589/jpts.23.401>.

158. Heisey CF, Kingsley JD. Effects of Static Stretching on Squat Performance in Division I Female Athletes. Int J Exerc Sci. 2016;9(3):359-67. doi: No DOI available.

159. Herman SL, Smith DT. Four-week dynamic stretching warm-up intervention elicits longer-term performance benefits. J Strength Cond Res. 2008;22(4):1286-97. doi: <https://doi.org/10.1519/JSC.0b013e318173da50>.

160. Higuchi T, Nakao Y, Tanaka Y, Sadakiyo M, Hamada K, Yokoyama S. Acute effects of doorway stretch on the glenohumeral rotational range of motion and scapular position in high-school baseball players. JSES Int. 2021;5(6):972-7. doi: <https://doi.org/10.1016/j.jseint.2021.07.002>.

161. Holt BW, Lambourne K. The impact of different warm-up protocols on vertical jump performance in male collegiate athletes. J Strength Cond Res. 2008;22(1):226-9. doi: <https://doi.org/10.1519/JSC.0b013e31815f9d6a>.

162. Hough PA, Ross EZ, Howatson G. Effects of dynamic and static stretching on vertical jump performance and electromyographic activity. J Strength Cond Res. 2009;23(2):507-12. doi: <https://doi.org/10.1519/JSC.0b013e31818cc65d>.

163. Hsu FY, Tsai KL, Lee CL, Chang WD, Chang NJ. Effects of Dynamic Stretching Combined With Static Stretching, Foam Rolling, or Vibration Rolling as a Warm-Up Exercise on Athletic Performance in Elite Table Tennis Players. J Sport Rehabil. 2021;30(2):198-205. doi: <https://doi.org/10.1123/jsr.2019-0442>.

164. Huang S, Zhang HJ, Wang X, Lee WCC, Lam WK. Acute Effects of Soleus Stretching on Ankle Flexibility, Dynamic Balance and Speed Performances in Soccer Players. Biology (Basel). 2022;11(3):Article 374. doi: <https://doi.org/10.3390/biology11030374>.

165. Huang Y, Zhu X-n. 男子跆拳道运动员下肢肌肉最大肌力肌电电压受PNF牵拉训练影响的研究 [Research on the Effects of PNF Stretching on Strength and EMG Parameters in Taekwondo Male Players]. J Beijing Sport University. 2011;34(10):56-68. doi: No DOI available.

166. Ide BN, Moreira A, Schoenfeld BJ, Lodo L, Santos AR, Barbosa WP, et al. Acute effect of different warm-up interventions on neuromuscular performance of recreational soccer players. Rev Bras Ciência Mov. 2017;25(3):34-43. doi: <https://doi.org/10.31501/rbcm.v25i3.7053>.

167. Ishak A, Ahmad H, Mohamed NI, Rosman NA. The effects of different volumes of dynamic stretching on 20-m repeated sprint ability performance. J Fundam Appl Sci. 2017;9:1041-9. doi: <https://doi.org/10.4314/jfas.v9i6s.76>.

168. Ishak A, Ahmad H, Wong FY, Rejeb A, Hashim HA, Pullinger SA. Two sets of dynamic stretching of the lower body musculature improves linear repeated-sprint performance in team-sports. Asian J Sports Med. 2019;10(3). doi: <https://doi.org/10.5812/asjsm.91775>.

169. Ishak A, Priestley J, Malik ZA, Osman N, Txi MRS, Rosman N, Ping FWC. Acute effect of different sets of ballistic stretching protocol on repeated sprint performance among football players. Eur J Mol Clin Med. 2020;7(2):5896-903. doi: No DOI available.

170. Jang HS, Kim D, Park J. Immediate effects of different types of stretching exercises on badminton jump smash. J Sports Med Phys Fit. 2018;58(7-8):1014-20. doi: <https://doi.org/10.23736/s0022-4707.17.06989-4>.

171. Jemni M, Mkaouer B, Marina M, Asllani A, Sands WA. Acute static vibration-induced stretching enhanced muscle viscoelasticity but did not affect maximal voluntary contractions in footballers. J Strength Cond Res. 2014;28(11):3105-14. doi: <https://doi.org/10.1519/jsc.0000000000000404>.

172. Jing L, Yang G. Effects of two kinds of stretch training on lower limb kinetics and energy absorption pattern during cushioning period. Chin J Appl Mech. 2021;38(6):2432-40. doi: <https://doi.org/10.11776/cjam.38.06.D040>.

173. Johnson AW, Warcup CN, Seeley MK, Eggett D, Feland JB. The acute effects of stretching with vibration on dynamic flexibility in young female gymnasts. J Sports Med Phys Fit. 2019;59(2):210-6. doi: <https://doi.org/10.23736/s0022-4707.18.08290-7>.

174. Jordan JB, Korgaokar AD, Farley RS, Caputo JL. Acute Effects of Static and Proprioceptive Neuromuscular Facilitation Stretching on Agility Performance in Elite Youth Soccer Players. Int J Exerc Sci. 2012;5(2):97-105. doi: No DOI available.

175. Junqueira C, Rudnick CT, Facci LM. Stretching Global Ativo comparado aos alongamentos convencionais em atletas de basquetebol [Active Global Stretching compared to conventional stretching in basketball athletes]. Rev Terapia Manual. 2011;9(45):612-8. doi: No DOI available.

176. Kafkas A, Eken Ö, Kurt C, Kafkas ME. The effects of different stretching and warm-up exercise protocols on 50-meter swimming performance in sub-elite women swimmers. Isokinet Exerc Sci. 2019;27(4):289-97. doi: <https://doi.org/10.3233/ies-193141>.

177. Karloh M, dos Santos RP, Kraeski MH, Matias TS, Frutuoso AS. Alongamento estático versus conceito Mulligan - efeitos crônicos no treino de flexibilidade em ginastas [Static stretch versus Mulligan Concept - long-term effects in gymnast's flexibility]. Rev Bras Cineantropometria Desempenho Hum. 2010;12(3):202-8. doi: <https://doi.org/10.5007/1980-0037.2010v12n3p202>.

178. Karloh M, dos Santos RP, Kraeski MH, Matias TS, Kraeski D, de Menezes FS. Alongamento estático versus conceito Mulligan: aplicações no treino de flexibilidade em ginastas [Static stretch versus Mulligan concept: flexibility training in gymnasts]. Fisioter Mov. 2010;23(4):523-33. doi: <https://doi.org/10.1590/S0103-51502010000400003>.

179. Kazemi O, Letafatkar A, Marchetti PH. Effect of Stretching Protocols on Glenohumeral-Joint Muscle Activation in Elite Table Tennis Players. Int J Sports Physiol Perform. 2021;16(1):110-6. doi: <https://doi.org/10.1123/ijspp.2019-0768>.

180. Kilit B, Arslan E, Soylu Y. Effects of different stretching methods on speed and agility performance in young tennis players. Sci Sports. 2019;34(5):313-20. doi: <https://doi.org/10.1016/j.scispo.2018.10.016>.

181. Konrad A, Tilp M, Stöcker F, Mehmeti L, Mahnič N, Seiberl W, et al. Quadriceps or triceps surae proprioceptive neuromuscular facilitation stretching with post-stretching dynamic activities does not induce acute changes in running economy. Front Physiol. 2022;13:981108. doi: <https://doi.org/10.3389/fphys.2022.981108>.

182. Kornberg C, Ther GDM, McCarthy T. The effect of neural stretching technique on sympathetic outflow to the lower limbs. J Orthop Sports Phys Ther. 1992;16(6):269-74. doi: <https://doi.org/10.2519/jospt.1992.16.6.269>.

183. Krčmár M, Šimonek J, Polačková B. Impact of different warm-up modalities on the height of countermovement vertical jump and its practical applicability. J Phys Educ Sport. 2016;16(2):481-8. doi: <https://doi.org/10.7752/jpes.2016.02074>.

184. Kruse NT, Barr MW, Gilders RM, Kushnick MR, Rana SR. Using a practical approach for determining the most effective stretching strategy in female college Division I volleyball players. J Strength Cond Res. 2013;27(11):3060-7. doi: <https://doi.org/10.1519/JSC.0b013e31828bf2b6>.

185. Kruse NT, Barr MW, Gilders RM, Kushnick MR, Rana SR. Effect of different stretching strategies on the kinetics of vertical jumping in female volleyball athletes. J Sport Health Sci. 2015;4(4):364-70. doi: <https://doi.org/10.1016/j.jshs.2014.06.003>.

186. Kurt C. Alternative to traditional stretching methods for flexibility enhancement in well-trained combat athletes: local vibration versus whole-body vibration. Biol Sport. 2015;32(3):225-33. doi: <https://doi.org/10.5604/20831862.1150305>.

187. Kurt C, Firtin I. Comparison of the acute effects of static and dynamic stretching exercises on flexibility, agility and anaerobic performance in professional football players. Turk J Phys Med Rehabil. 2016;62(3):206-13. doi: <https://doi.org/10.5606/tftrd.2016.32698>.

188. Kurtdere I, Kurt C, Nebioglu IO. Acute static stretching with different volumes improves hamstring flexibility but not reactive strength index and leg stiffness in well-trained judo athletes. J Hum Sport Exerc. 2021;16(4):760-71. doi: <https://doi.org/10.14198/jhse.2021.164.03>.

189. Kyranoudis Α, Arsenis S, Ispyrlidis I, Chatzinikolaou A, Gourgoulis V, Kyranoudis E, Metaxas T. The acute effects of combined foam rolling and static stretching program on hip flexion and jumping ability in soccer players. J Phys Educ Sport. 2019;19(2):1164-72. doi: <https://doi.org/10.7752/jpes.2019.02169>.

190. Kyranoudis Α, Nikolaidis V, Ispirlidis I, Galazoulas C, Alipasali F, Famisis K. Acute effect of specific warm-up exercises on sprint performance after static and dynamic stretching in amateur soccer players. J Phys Educ Sport. 2018;18(2):825-30. doi: <https://doi.org/10.7752/jpes.2018.02122>.

191. Laudner KG, Sipes RC, Wilson JT. The acute effects of sleeper stretches on shoulder range of motion. J Athl Train. 2008;43(4):359-63. doi: <https://doi.org/10.4085/1062-6050-43.4.359>.

192. Li B, Bai X, Zhu Y. Study on the effect of PNF method on the flexibility and strength quality of stretching muscles of shoulder joints of swimmers. MCB Mol Cell Biomech. 2021;18(2):99-105. doi: <https://doi.org/10.32604/MCB.2021.014748>.

193. Lin WC, Lee CL, Chang NJ. Acute Effects of Dynamic Stretching Followed by Vibration Foam Rolling on Sports Performance of Badminton Athletes. J Sports Sci Med. 2020;19(2):420-8. doi: No DOI available.

194. Little T, Williams AG. Effects of differential stretching protocols during warm-ups on high-speed motor capacities in professional soccer players. J Strength Cond Res. 2006;20(1):203-7. doi: <https://doi.org/10.1519/R-16944.1>.

195. Lotfi N, Mohamadi S, Mirzaei-Takmil M. Effects of stretching before intense exercise training on hematologic and cellular injury indices. Pedagog Psychol Med Biol Probl Phys Train Sports. 2018;22(6):301-5. doi: <https://doi.org/10.15561/18189172.2018.0604>.

196. Loughran M, Glasgow P, Bleakley C, McVeigh J. The effects of a combined static-dynamic stretching protocol on athletic performance in elite Gaelic footballers: A randomised controlled crossover trial. Phys Ther Sport. 2017;25:47-54. doi: <https://doi.org/10.1016/j.ptsp.2016.11.006>.

197. Lowery RP, Joy JM, Brown LE, de Souza EO, Wistocki DR, Davis GS, et al. Effects of static stretching on 1-mile uphill run performance. J Strength Cond Res. 2014;28(1):161-7. doi: <https://doi.org/10.1519/JSC.0b013e3182956461>.

198. Makaruk H, Makaruk B, Sacewicz T. The effects of static stretching and isometric strength on hamstring strength and flexibility asymmetry. Pol J Sport Tour. 2010;17(3):153-6. doi: No DOI available.

199. Manzi V, Iellamo F, Alashram AR, D'Onofrio R, Padua E, Casasco M, Annino G. Effects of three different stretching protocols on hamstring muscle flexibility in professional soccer players: a randomized study. J Sports Med Phys Fit. 2020;60(7):999-1004. doi: <https://doi.org/10.23736/s0022-4707.20.10562-0>.

200. Mariscal SL, Garcia VS, Fernandez-Garcia JC, de Villarreal ES. Acute effects of ballistic vs. passive static stretching involved in a prematch warm-up on vertical jump and linear sprint performance in soccer players. J Strength Cond Res. 2021;35(1):147-53. doi: <https://doi.org/10.1519/jsc.0000000000002477>.

201. Martin P, Pavol P, Zuzana P, Martina T, Juraji M. Effectiveness of static and dynamic stretching prior to speed and speed-strength load. J Phys Educ Sport. 2014;14(4):455-8. doi: <https://doi.org/10.7752/jpes.2014.04069>.

202. Martinez-Chicote R, Brizuela G, Perez-Soriano P, Llana-Belloch S. Acute effect of 3 stretching techniques in sideward movements in tennis. Eur J Hum Mov. 2016;36:48-56. doi: No DOI available.

203. Mascarin NC, Vancini RL, Lira CAB, Andrade MS. Stretch-induced reductions in throwing performance are attenuated by warm-up before exercise. J Strength Cond Res. 2015;29(5):1393-8. doi: <https://doi.org/10.1519/jsc.0000000000000752>.

204. McNeal JR, Sands WA. Acute static stretching reduces lower extremity power in trained children. Pediatr Exerc Sci. 2003;15(2):139-45. doi: <https://doi.org/10.1123/pes.15.2.139>.

205. McNeal JR, Edgerly S, Sands WA, Kawaguchi J. Acute effects of vibration-assisted stretching are more evident in the non-dominant limb. Eur J Sport Sci. 2011;11(1):45-50. doi: <https://doi.org/10.1080/17461391003774642>.

206. Meerits T, Bacchieri S, Pääsuke M, Ereline J, Cicchella A, Gapeyeva H. Acute effect of static and dynamic stretching on tone and elasticity of hamstring muscles and on vertical jump performance in track-and-field athletes. Acta Kinesiol Univ Tartu. 2014;20:48-59. doi: <https://doi.org/10.12697/akut.2014.20.05>.

207. Melocchi I, Filipas L, Lovecchio N, De Nardi M, La Torre A, Codella R. Effects of different stretching methods on vertical jump ability and range of motion in young female artistic gymnastics athletes. J Sports Med Phys Fit. 2021;61(4):527-33. doi: <https://doi.org/10.23736/s0022-4707.20.11386-0>.

208. Merrigan JJ, Tynan MN, Oliver JM, Jagim AR, Jones MT. Effect of Post-Exercise Whole Body Vibration with Stretching on Mood State, Fatigue, and Soreness in Collegiate Swimmers. Sports (Basel). 2017;5(1):Article 7. doi: <https://doi.org/10.3390/sports5010007>.

209. Mikolajec K, Waskiewicz Z, Maszczyk A, Bacik B, Kurek P, Zajac A. Effects of stretching and strength exercises on speed and power abilities in male basketball players. Isokinet Exerc Sci. 2012;20(1):61-9. doi: <https://doi.org/10.3233/ies-2012-0442>.

210. Miladi I, Temfemo A, Mandengue SH, Ahmaidi S. Effect of recovery mode on exercise time to exhaustion, cardiorespiratory responses, and blood lactate after prior, intermittent supramaximal exercise. J Strength Cond Res. 2011;25(1):205-10. doi: <https://doi.org/10.1519/JSC.0b013e3181af5152>.

211. Mojock CD, Kim JS, Eccles DW, Panton LB. The effects of static stretching on running economy and endurance performance in female distance runners during treadmill running. J Strength Cond Res. 2011;25(8):2170-6. doi: <https://doi.org/10.1519/JSC.0b013e3181e859db>.

212. Molacek ZD, Conley DS, Evetovich TK, Hinnerichs KR. Effects of low- and high-volume stretching on bench press performance in collegiate football players. J Strength Cond Res. 2010;24(3):711-6. doi: <https://doi.org/10.1519/JSC.0b013e3181c7c242>.

213. Montalvo S, Dorgo S. The effect of different stretching protocols on vertical jump measures in college age gymnasts. J Sports Med Phys Fit. 2019;59(12):1956-62. doi: <https://doi.org/10.23736/s0022-4707.19.09561-6>.

214. Moore MA, Hutton RS. Electromyographic investigation of muscle stretching techniques. Med Sci Sports Exerc. 1980;12(5):322-9. doi: <https://doi.org/10.1249/00005768-198025000-00004>.

215. Mor A, Yurtseven R, Mor H, Acar K. 11-12 yaş grubu futbolcularda farkli isinma protokollerinin bazi performans parametrelerine etkisi [The effects of different warm-up protocols on some performance parameters in 11-12 age group football players]. J Phys Educ Sports Sci. 2021;19(4):72-83. doi: <https://doi.org/10.33689/spormetre.907920>.

216. Moran KA, McGrath T, Marshall BM, Wallace ES. Dynamic Stretching and Golf Swing Performance. Int J Sports Med. 2009;30(2):113-8. doi: <https://doi.org/10.1055/s-0028-1103303>.

217. Moran MP, Whitehead JR, Guggenheimer JD, Brinkert RH. The Effects of Static Stretching Warm-up Versus Dynamic Warm-up on Sprint Swim Performance. J Swim Res. 2014;22(1):1-9. doi: No DOI available.

218. Moreno-Perez V, Hernandez-Davo JL, Nakamura F, Lopez-Samanes A, Jimenez-Reyes P, Fernandez-Fernandez J, Behm DG. Post-activation performance enhancement of dynamic stretching and heavy load warm-up strategies in elite tennis players. J Back Musculoskelet Rehabil. 2021;34(3):413-23. doi: <https://doi.org/10.3233/bmr-191710>.

219. Needham RA, Morse CI, Degens H. The acute effect of different warm-up protocols on anaerobic performance in elite youth soccer players. J Strength Cond Res. 2009;23(9):1614-20. doi: <https://doi.org/10.1519/JSC.0b013e3181b1f3ef>.

220. Nelson AG, Driscoll NM, Landin DK, Young MA, Schexnayder IC. Acute effects of passive muscle stretching on sprint performance. J Sports Sci. 2005;23(5):449-54. doi: <https://doi.org/10.1080/02640410410001730205>.

221. Nobre TL, Rocha LY, Ramos CC, Mazuchi F, Carbone PO, Madureira D, et al. The use of proprioceptive neuromuscular facilitation for increasing throwing performance. Rev Bras Med Esporte. 2020;26(4):332-6. doi: <https://doi.org/10.1590/1517-869220202604185184>.

222. Notarnicola A, Perroni F, Campese A, Maccagnano G, Monno A, Moretti B, Tafuri S. Flexibility responses to different stretching methods in young elite basketball players. Muscles Ligaments Tendons J. 2017;7(4):582-9. doi: <https://doi.org/10.11138/mltj/2017.7.4.582>.

223. Nuri L, Ghotbi N, Faghihzadeh S. Acute Effects of Static Stretching, Active Warm Up, or Passive Warm Up on Flexibility of the Plantar Flexor Muscles of Iranian Professional Female Taekwondo Athletes. J Musculoskelet Pain. 2013;21(3):263-8. doi: <https://doi.org/10.3109/10582452.2013.827771>.

224. O'Sullivan K, Murray E, Sainsbury D. The effect of warm-up, static stretching and dynamic stretching on hamstring flexibility in previously injured subjects. BMC Musculoskelet Disord. 2009;10. doi: <https://doi.org/10.1186/1471-2474-10-37>.

225. Olivares-Arancibia J, Solis-Urra P, Rodriguez-Rodriguez F, Santos-Lozano A, Sanchez-Martinez J, Martin-Hernandez J, et al. A single bout of whole-body vibration improves hamstring flexibility in university athletes: A randomized controlled trial. J Hum Sport Exerc. 2018;13(4):776-88. doi: <https://doi.org/10.14198/jhse.2018.134.06>.

226. Oliveira LP, Vieira LHP, Aquino R, Manechini JPV, Santiago PRP, Puggina EF. Acute effects of active, ballistic, passive, and proprioceptive neuromuscular facilitation stretching on sprint and vertical jump performance in trained young soccer players. J Strength Cond Res. 2018;32(8):2199-208. doi: <https://doi.org/10.1519/jsc.0000000000002298>.

227. Oña Tacan EJ, Chamorro Werz DN, Chávez Cevallos E. Insistencia pasiva dinámica y contracción maximal: Influencia en la flexibilidad del split en kárate [Dynamic passive insistence and maximal contraction: Flexibility influence on the karate split]. Podium. 2021;16(2):524-34. doi: No DOI available.

228. Oskouei ST, Abazari R, Kahjoogh MA, Goljaryan S, Zohrabi S. The effect of static stretching of agonist and antagonist muscles on knee joint position sense. Int J Ther Rehabil. 2021;28(10). doi: <https://doi.org/10.12968/ijtr.2020.0043>.

229. Osternig LR, Robertson RN, Troxel RK, Hansen P. Differential responses to proprioceptive neuromuscular facilitation (PNF) stretch techniques. Med Sci Sports Exerc. 1990;22(1):106-11. doi: <https://doi.org/10.1249/00005768-199002000-00017>.

230. Pagaduan JC, Pojskic H, Uzicanin E, Babajic F. Effect of Various Warm-Up Protocols on Jump Performance in College Football Players. J Hum Kinet. 2012;35:127-32. doi: <https://doi.org/10.2478/v10078-012-0086-5>.

231. Panidi I, Bogdanis GC, Terzis G, Donti A, Konrad A, Gaspari V, Donti O. Muscle Architectural and Functional Adaptations Following 12-Weeks of Stretching in Adolescent Female Athletes. Front Physiol. 2021;12:Article 701338. doi: <https://doi.org/10.3389/fphys.2021.701338>.

232. Papadimitriou K, Loupos D, Tsalis G, Manou B. Effects of proprioceptive neuromuscular facilitation (PNF) on swimmers leg mobility and performance. J Phys Educ Sport. 2017;17(2):663-8. doi: <https://doi.org/10.7752/jpes.2017.02099>.

233. Papia K, Bogdanis GC, Toubekis A, Donti A, Donti O. Acute effects of prolonged static stretching on jumping performance and range of motion in young female gymnasts. Sci Gymnast J. 2018;10(2):217-26. doi: No DOI available.

234. Pellegrini A, Tonino P, Salazar D, Hendrix K, Parel I, Cutti A, et al. Can posterior capsular stretching rehabilitation protocol change scapula kinematics in asymptomatic baseball pitchers? Musculoskelet Surg. 2016;100:39-43. doi: <https://doi.org/10.1007/s12306-016-0416-1>.

235. Penichet-Tomas A, Pueo B, Abad-Lopez M, Jimenez-Olmedo JM. Acute Comparative Effect of Foam Rolling and Static Stretching on Range of Motion in Rowers. Sustainability. 2021;13(7). doi: <https://doi.org/10.3390/su13073631>.

236. Pojskic H, Pagaduan JC, Babajic F, Uzicanin E, Muratovic M, Tomljanovic M. Acute effects of prolonged intermittent low-intensity isometric warm-up schemes on jump, sprint, and agility performance in collegiate soccer players. Biol Sport. 2015;32(2):129-34. doi: <https://doi.org/10.5604/20831862.1140427>.

237. Polat SC, Cetin E, Yarim I, Bulgay C, Cicioglu HI. Effect of ballistic warm-up on isokinetic strength, balance, agility, flexibility and speed in elite freestyle wrestlers. Sport Mont. 2018;16(3):85-9. doi: <https://doi.org/10.26773/smj.181015>.

238. Pooley S, Spendiff O, Allen M, Moir HJ. Static stretching does not enhance recovery in elite youth soccer players. BMJ Open Sport Exerc Med. 2017;3(1). doi: <https://doi.org/10.1136/bmjsem-2016-000202>.

239. Pooley S, Spendiff O, Allen M, Moir HJ. Comparative efficacy of active recovery and cold water immersion as post-match recovery interventions in elite youth soccer. J Sports Sci. 2020;38(11-12):1423-31. doi: <https://doi.org/10.1080/02640414.2019.1660448>.

240. Popelka J, Pivovarniček P. Comparison of the effects of static and dynamic stretching on the force-velocity capabilities of young volleyball players. J Phys Educ Sport. 2018;18(4):2314-8. doi: <https://doi.org/10.7752/jpes.2018.04349>.

241. Popelka J, Beťák B, Pivovarniček P. In which indicators can the difference between effectivity of static and dynamic stretching of young volleyball players be noticed? Sport Sci. 2020;14(1):21-6. doi: No DOI available.

242. Popelka J, Pivovarniček P. The effect comparison of foam rolling and dynamic stretching on performance in motion tests by young volleyball players: a pilot study. Phys Act Rev. 2022;10(2):140-9. doi: <https://doi.org/10.16926/par.2022.10.28>.

243. Portilla-Dorado E, Villaquiran-Hurtado A, Molano-Tobar N. Potencia del salto en jugadores de fútbol sala después de la utilización del rodillo de espuma y la facilitación neuromuscular propioceptiva en la musculatura isquiosural [Jump power in futsal players after foam roller use and proprioceptive neuromuscular facilitation in the ischiosural muscles]. Revista de la Academia Colombiana de Ciencias Exactas, Fisicas y Naturales. 2019;43(167):165-76. doi: <https://doi.org/10.18257/raccefyn.846>.

244. Racil G, Jlid MC, Bouzid MS, Sioud R, Khalifa R, Amri M, et al. Effects of flexibility combined with plyometric exercises vs. isolated plyometric or flexibility mode in adolescent male hurdlers. J Sports Med Phys Fit. 2020;60(1):45-52. doi: <https://doi.org/10.23736/s0022-4707.19.09906-7>.

245. Reis EDS, Pereira GB, de Sousa NMF, Tibana RA, Silva MF, Araujo M, et al. Acute effects of proprioceptive neuromuscular facilitation and static stretching on maximal voluntary contraction and muscle electromyographical activity in indoor soccer players. Clin Physiol Funct Imaging. 2013;33(6):418-22. doi: <https://doi.org/10.1111/cpf.12047>.

246. Reuther KE, Larsen R, Kuhn PD, Kelly JD, Thomas SJ. Sleeper stretch accelerates recovery of glenohumeral internal rotation after pitching. J Shoulder Elbow Surg. 2016;25(12):1925-9. doi: <https://doi.org/10.1016/j.jse.2016.07.075>.

247. Robey E, Dawson B, Goodman C, Beilby J. Effect of Postexercise Recovery Procedures Following Strenuous Stair-climb Running. Res Sports Med. 2009;17(4):245-59. doi: <https://doi.org/10.1080/15438620902901276>.

248. Rodriguez-Marroyo JA, Gonzalez B, Foster C, Carballo-Leyenda AB, Villa JG. Effect of the Cooldown Type on Session Rating of Perceived Exertion. Int J Sports Physiol Perform. 2021;16(4):573-7. doi: <https://doi.org/10.1123/ijspp.2020-0225>.

249. Rogan S, Blasimann A, Steiger M, Torre A, Radlinger L. Acute Effects of Fast Dynamic Stretching on Rate of Force Development in Ice Hockey Players: A Pilot Study. Sportverletzung-Sportschaden. 2012;26(4):207-11. doi: <https://doi.org/10.1055/s-0032-1325416>.

250. Romero-Franco N, Parraga-Montilla JA, Molina-Flores EM, Jimenez-Reyes P. Effects of Combining Running and Practical Duration Stretching on Proprioceptive Skills of National Sprinters. J Strength Cond Res. 2020;34(4):1158-65. doi: <https://doi.org/10.1519/jsc.0000000000002620>.

251. Sagiroglu I, Kurt C, Pekunlu E, Ozsu I. Residual effects of static stretching and self-myofascial-release exercises on flexibility and lower body explosive strength in well-trained combat athletes. Isokinet Exerc Sci. 2017;25(2):135-41. doi: <https://doi.org/10.3233/ies-160656>.

252. Sampaio-Jorge F, Rangel LFC, Mota HR, Morales AP, Costa L, Coelho GMO, Ribeiro BG. Acute effects of passive stretching on muscle power performance. J Exerc Physiol Online. 2014;17(6):81-9. doi: No DOI available.

253. Sánchez-Sánchez J, Rodríguez-Fernández A, Villa-Vicente G, Petisco-Rodríguez C, Ramírez-Campillo R, Gonzalo-Skok O. Efecto de un calentamiento con estiramientos estáticos y dinámicos sobre el salto horizontal y la capacidad para repetir esprint con cambio de dirección [Effect of warm-up with static and dynamic stretching on the horizontal jump and repeated sprint ability with changes of direction]. RICYDE. 2017;13(47):26-38. doi: <https://doi.org/10.5232/ricyde2017.04702>.

254. Sands WA, McNeal JR, Stone MH, Kimmel WL, Haff GG, Jemni M. The effect of vibration on active and passive range of motion in elite female synchronized swimmers. Eur J Sport Sci. 2008;8(4):217-23. doi: <https://doi.org/10.1080/17461390802116682>.

255. Sands WA, McNeal JR, Stone MH, Russell EM, Jemni M. Flexibility enhancement with vibration: Acute and long-term. Med Sci Sports Exerc. 2006;38(4):720-5. doi: <https://doi.org/10.1249/01.mss.0000210204.10200.dc>.

256. Sayers AL, Farley RS, Fuller DK, Jubenville CB, Caputo JL. The effect of static stretching on phases of sprint performance in elite soccer players. J Strength Cond Res. 2008;22(5):1416-21. doi: <https://doi.org/10.1519/JSC.0b013e318181a450>.

257. Schmitt GD, Pelham TW, Holt LE. Changes in flexibility of elite female soccer players resulting from a flexibility program or combined flexibility and strength program: A pilot study. Clin Kinesiol. 1998;52(3):64-7. doi: No DOI available.

258. Schmitt GD, Pelham TW, Holt LE. A comparison of selected protocols during proprioceptive neuromuscular facilitation stretching. Clin Kinesiol. 1999;53(1):16-21. doi: No DOI available.

259. Seçer E, Kaya DO. Comparison of Immediate Effects of Foam Rolling and Dynamic Stretching to Only Dynamic Stretching on Flexibility, Balance, and Agility in Male Soccer Players. J Sport Rehabil. 2022;31(1):10-6. doi: <https://doi.org/10.1123/jsr.2021-0017>.

260. Sekir U, Arabaci R, Akova B, Kadagan SM. Acute effects of static and dynamic stretching on leg flexor and extensor isokinetic strength in elite women athletes. Scand J Med Sci Sports. 2010;20(2):268-81. doi: <https://doi.org/10.1111/j.1600-0838.2009.00923.x>.

261. Sekir U, Arabaci R, Akova B. Acute Effects of Dynamic Stretching on Peak and End-Range Functional Hamstring/Quadriceps Strength Ratios. Turk Klin J Med Sci. 2010;30(1):164-73. doi: <https://doi.org/10.5336/medsci.2008-8752>.

262. Selkar SP, Ramteke GJ, Dongare AK. Effect of eccentric muscle training to reduce severity of delayed onset muscle soreness in athletic subjects. Eur J Gen Med. 2009;6(4):213-7. doi: <https://doi.org/10.29333/ejgm/82672>.

263. Sermaxhaj S, Arifi F, Bahtiri A. The effect of static stretching in agility and isokinetic force at football players. Sport Mont. 2017;15(3):29-33. doi: <https://doi.org/10.26773/smj.2017.10.005>.

264. Sermaxhaj S, Arifi F, Bahtiri A, Alaj I. The impact of recuperation with static stretching in flexibility and agility with and without ball of young soccer players. Acta Kinesiol. 2017;11(Suppl 1):33-8. doi: No DOI available.

265. Sermaxhaj S, Popovic S, Bjelica D, Gardasevic J, Arifi F. Effect of recuperation with static stretching in isokinetic force of young football players. J Phys Educ Sport. 2017;17(3):1948-53. doi: <https://doi.org/10.7752/jpes.2017.03191>.

266. Sermaxhaj S, Arifi F, Iber A, Bahtiri A, Havolli J, Sermaxhaj S. The effect of static stretching in agility and isokinetic force at football players. Sport Mont. 2018;16(2):45-9. doi: <https://doi.org/10.26773/smj.180608>.

267. Sermaxhaj S, Arifi F, Havolli J, Luta F, Isufi I. The effect of physical exercise according to a programme for the development of flexibility in the motor abilities of young football players. Sport Mont. 2021;19(1):25-9. doi: <https://doi.org/10.26773/SMJ.210209>.

268. Sheard PW, Paine TJ. Optimal contraction intensity during proprioceptive neuromuscular facilitation for maximal increase of range of motion. J Strength Cond Res. 2010;24(2):416-21. doi: <https://doi.org/10.1519/JSC.0b013e3181c50a0d>.

269. Shekadar M, Ganesh MSP, Mitra M. The Immediate Effects of Sleeper Stretch Versus Cross-body Stretch on Shoulder Range of Motion in Volleyball Players. Indian J Physiother Occup Ther. 2016;10(4):26-31. doi: <https://doi.org/10.5958/0973-5674.2016.00114.3>.

270. Shitara H, Tajika T, Kuboi T, Ichinose T, Sasaki T, Hamano N, et al. Shoulder stretching versus shoulder muscle strength training for the prevention of baseball-related arm injuries: a randomized, active-controlled, open-label, non-inferiority study. Sci Rep. 2022;12(1):22118. doi: <https://doi.org/10.1038/s41598-022-26682-1>.

271. Siatras T, Papadopoulos G, Mameletzi D, Gerodimos V, Kellis S. Static and dynamic acute stretching effect on gymnasts' speed in vaulting. Pediatr Exerc Sci. 2003;15(4):383-91. doi: <https://doi.org/10.1123/pes.15.4.383>.

272. Silva GF, Almeida AR, Rodrigues SA, Szmuchrowski LA, da Silva RAD, Drummond MDM. The Acute Effect of a Sport-Specific Stretching Routine on the Performance of Vertical Jumps in Rhythmic Gymnasts. J Exerc Physiol Online. 2018;21(2):30-9. doi: No DOI available.

273. Sim AY, Dawson BT, Guelfi KJ, Wallman KE, Young WB. Effects of static stretching in warm-up on repeated sprint performance. J Strength Cond Res. 2009;23(7):2155-62. doi: <https://doi.org/10.1519/JSC.0b013e3181b438f3>.

274. Skarabot J, Beardsley C, Stirn I. Comparing the effects of self-myofascial release with static stretching on ankle range-of-motion in adolescent athletes. Int J Sports Phys Ther. 2015;10(2):203-12. doi: No DOI available.

275. Solon Júnior LJF, Neto LVD. Efeito do alongamento estático e da corrida submáxima no desempenho do salto contramovimento e sprint em jogadores universitários de voleibol [Effect of Static Stretching and Submaximal Running on Countermovement Jump Performance and Sprint on College Volleyball Players]. Retos. 2021(39):325-9. doi: <https://doi.org/10.47197/retos.v0i39.79344>.

276. Song Y, Zhao XX, Finnie KP, Shao SR. Biomechanical Analysis of Vertical Jump Performance in Well-Trained Young Group before and after Passive Static Stretching of Knee Flexors Muscles. J Biomimetics, Biomater Biomed Eng. 2018;36:24-33. doi: <https://doi.org/10.4028/www.scientific.net/JBBBE.36.24>.

277. Stevanovic VB, Jelic MB, Milanovic SD, Filipovic SR, Mikic MJ, Stojanovic MDM. Sport-Specific Warm-Up Attenuates Static Stretching-Induced Negative Effects on Vertical Jump But Not Neuromuscular Excitability in Basketball Players. J Sports Sci Med. 2019;18(2):282-9. doi: No DOI available.

278. Stewart M, Adams R, Alonso A, Van Koesveld B, Campbell S. Warm-up or stretch as preparation for sprint performance? J Sci Med Sport. 2007;10(6):403-10. doi: <https://doi.org/10.1016/j.jsams.2006.10.001>.

279. Stojanovic MD, Mikic M, Vucetic V, Belegisanin B, Karac A, Bianco A, Drid P. Acute effects of static and dynamic stretching on vertical jump performance in adolescent basketball players. Gazz Med Ital. 2022;181(6):417-24. doi: <https://doi.org/10.23736/S0393-3660.20.04575-1>.

280. Su RH, Wei C, Hsu MC. Effects of Different Stretching Strategies on Soccer Players' Power, Speed, and Muscle Strength Performance. Rev de Cercet si Interv Soc. 2019;66:328-41. doi: <https://doi.org/10.33788/rcis.66.19>.

281. Sudhakar S, Padmasheela V. To investigate the effects of different warm- up protocols in vertical jump performance in male collegiate volleyball players. Int J Sports Sci Fit. 2012;2(1):142-53. doi: No DOI available.

282. Taber CB, Colter RJ, Davis JJ, Seweje PA, Wilson DP, Foster JZ, Merrigan JJ. The Effects of Body Tempering on Force Production, Flexibility and Muscle Soreness in Collegiate Football Athletes. J Funct Morphol Kinesiol. 2022;7(1). doi: <https://doi.org/10.3390/JFMK7010009>.

283. Takeuchi K, Tsukuda F. Comparison of the effects of static stretching on range of motion and jump height between quadriceps, hamstrings and triceps surae in collegiate basketball players. BMJ Open Sport Exerc Med. 2019;5(1). doi: <https://doi.org/10.1136/bmjsem-2019-000631>.

284. Taleb-Beydokhti I, Haghshenas R. Static versus dynamic stretching: Chronic and acute effects on Agility performance in male athletes. Int J Appl Exerc Physiol. 2015;4(1):1-8. doi: No DOI available.

285. Tammam AH, Hashem EM. Individual and Combined Effects of PNF Stretching and Plyometric Training on Muscular Power and Flexibility for Volleyball Players. Amazonia Investiga. 2020;9(36):73-82. doi: <https://doi.org/10.34069/ai/2020.36.12.6>.

286. Taylor KL, Sheppard JM, Lee H, Plummer N. Negative effect of static stretching restored when combined with a sport specific warm-up component. J Sci Med Sport. 2009;12(6):657-61. doi: <https://doi.org/10.1016/j.jsams.2008.04.004>.

287. Toft E, Espersen GT, Kålund S, Sinkjær T, Hornemann BC. Passive tension of the ankle before and after stretching. The American Journal of Sports Medicine. 1989;17(4):489-94. doi: <https://doi.org/10.1177/036354658901700407>.

288. Torres EM, Kraemer WJ, Vingren JL, Volek JS, Hatfield DL, Spiering BA, et al. Effects of stretching on upper-body muscular performance. J Strength Cond Res. 2008;22(4):1279-85. doi: <https://doi.org/10.1519/JSC.0b013e31816eb501>.

289. Tsolakis C, Douvis A, Tsigganos G, Zacharogiannis E, Smirniotou A. Acute Effects of Stretching on Flexibility, Power and Sport Specific Performance in Fencers. J Hum Kinet. 2010;26:105-14. doi: <https://doi.org/10.2478/v10078-010-0054-x>.

290. Turki O, Chaouachi A, Behm DG, Chtara H, Chtara M, Bishop D, et al. The effect of warm-ups incorporating different volumes of dynamic stretching on 10-and 20-m sprint performance in highly trained male athletes. J Strength Cond Res. 2012;26(1):63-72. doi: <https://doi.org/10.1519/JSC.0b013e31821ef846>.

291. Turki O, Dhahbi W, Padulo J, Khalifa R, Ridene S, Alamri K, et al. Warm-Up With Dynamic Stretching: Positive Effects on Match-Measured Change of Direction Performance in Young Elite Volleyball Players. Int J Sports Physiol Perform. 2020;15(4):528-33. doi: <https://doi.org/10.1123/ijspp.2019-0117>.

292. Turna B, Bayazit B, Eryucel ME, Yildiz M, Karademir MB. Acute effect of dynamic and static stretching exercises on targeting performance in archery. Prog Nutr. 2021;23. doi: <https://doi.org/10.23751/pn.v23iS1.11385>.

293. Unick J, Kieffer HS, Cheesman W, Feeney A. The acute effects of static and ballistic stretching on vertical jump performance in trained women. J Strength Cond Res. 2005;19(1):206-12. doi: <https://doi.org/10.1519/R-14843.1>.

294. Valdivia JED, Moreno PJF, Gonzalez JB, Pineda LTB, Valencia RTM, Gomez EG. Efectos de un programa de flexibilidad en el desarrollo de la fuerza muscular en jugadoras de futbol femenil [Effects of a program of stretching in the development of muscular strength in women's soccer players]. Educacion Fisica Y Ciencia. 2015;17(2). doi: No DOI available.

295. Van Gelder LH, Bartz SD. The effect of acute stretching on agility performance. J Strength Cond Res. 2011;25(11):3014-21. doi: <https://doi.org/10.1519/JSC.0b013e318212e42b>.

296. Van Zyl C, De Beer R, Bassett SH. The immediate effect of vibration therapy on flexibility in female junior elite gymnasts. Afr J Phys Health Educ Recreat Dance. 2011:20-8. doi: <https://doi.org/10.4314/ajpherd.v17i3.68069>.

297. Vasconcellos F, Salles PGdCMd, Cardozo GP, Achour Junior A, Mello DBd, Dantas EHM. Efeitos do flexionamento dinâmico agudo no impulsão vertical de jogadores de futebol. Fit Perform J Online. 2010;9(1):5-9. doi: <https://doi.org/10.3900/fpj.9.1.5.p>.

298. Vasconcellos F, Massaferri R, Reis M, Carnevale D, Salles PG, Brito J. Could the deleterious effect of stretching only influence soccer players with better performance in the vertical jump? Hum Mov. 2018;19(5 Special Issue):23-8. doi: <https://doi.org/10.5114/hm.2018.79736>.

299. Veevo M, Ereline J, Riso E-M, Gapeyeva H, Pääsuke M. The acute effects of warm-up, static and dynamic stretching exercises on biceps brachii muscle function in female basketball players. Acta Kinesiol Univ Tartu. 2012;18:39-46. doi: <https://doi.org/10.12697/akut.2012.18.05>.

300. Velasque R, Paulucio D, Alvarenga RL, Santos CG, Serpa TK, Machado M, et al. Could static stretching decrease anaerobic power in young soccer players? Med Sport (Roma). 2020;73(2):210-9. doi: <https://doi.org/10.23736/s0025-7826.20.03629-7>.

301. Walker MA, Li Y, Samson CO, Simpson KJ, Foutz T, Brown CN. Differences in trunk range of motion for various flexibility protocol types, particularly in quarterbacks wearing rib protectors. Sports Orthop Traumatol. 2021;37(1):41-50. doi: <https://doi.org/10.1016/j.orthtr.2020.11.001>.

302. Wallmann HW, Gillis CB, Martinez NJ. The effects of different stretching techniques of the quadriceps muscles on agility performance in female collegiate soccer athletes: a pilot study. N Am J Sports Phys Ther. 2008;3(1):41-7. doi: No DOI available.

303. Walsh GS. Effect of static and dynamic muscle stretching as part of warm up procedures on knee joint proprioception and strength. Hum Mov Sci. 2017;55:189-95. doi: <https://doi.org/10.1016/j.humov.2017.08.014>.

304. Werstein KM, Lund RJ. The effects of two stretching protocols on the reactive strength index in female soccer and rugby players. J Strength Cond Res. 2012;26(6):1564-7. doi: <https://doi.org/10.1519/JSC.0b013e318231ac09>.

305. West AD, Cooke MB, LaBounty PM, Byars AG, Greenwood M. Effects of G-trainer, cycle ergometry, and stretching on physiological and psychological recovery from endurance exercise. J Strength Cond Res. 2014;28(12):3453-61. doi: <https://doi.org/10.1519/jsc.0000000000000577>.

306. Williams JG, Laudner KG, McLoda T. The acute effects of two passive stretch maneuvers on pectoralis minor length and scapular kinematics among collegiate swimmers. Int J Sports Phys Ther. 2013;8(1):25-33. doi: No DOI available.

307. Wilson JM, Hornbuckle LM, Kim JS, Ugrinowitsch C, Lee SR, Zourdos MC, et al. Effects of static stretching on energy cost and running endurance performance. J Strength Cond Res. 2010;24(9):2274-9. doi: <https://doi.org/10.1519/JSC.0b013e3181b22ad6>.

308. Winchester JB, Nelson AG, Landin D, Young MA, Schexnayder IC. Static stretching impairs sprint performance in collegiate track and field athletes. J Strength Cond Res. 2008;22(1):13-8. doi: <https://doi.org/10.1519/JSC.0b013e31815ef202>.

309. Wong PL, Lau PWC, Mao DW, Wu YY, Behm DG, Wisloff U. Three days of static stretching within a warm-up does not affect repeated-sprint ability in youth soccer players. J Strength Cond Res. 2011;25(3):838-45. doi: <https://doi.org/10.1519/JSC.0b013e3181cc2266>.

310. Yamaguchi T, Takizawa K, Shibata K. Acute effect of dynamic stretching on endurance running performance in well-trained male runners. J Strength Cond Res. 2015;29(11):3045-52. doi: <https://doi.org/10.1519/jsc.0000000000000969>.

311. Yamaguchi T, Takizawa K, Shibata K, Tomabechi N, Samukawa M, Yamanaka M. Effect of General Warm-Up Plus Dynamic Stretching on Endurance Running Performance in Well-Trained Male Runners. Res Q Exerc Sport. 2019;90(4):527-33. doi: <https://doi.org/10.1080/02701367.2019.1630700>.

312. Yamaguchi T, Takizawa K, Shibata K, Tomabechi N, Samukawa M, Yamanaka M. Acute effect of dynamic stretching or running on endurance running performance in well-trained male runners. Gazz Med Ital. 2020;179(1-2):13-9. doi: <https://doi.org/10.23736/s0393-3660.18.03987-6>.

313. Yaşli BÇ, Müniroğlu RS. Futbolcularda 8 haftalik statik germe antrenmanlarinin siçrama performansina etkileri [The effects of 8 weeks static stretching traning on jumping in soccer players]. J Phys Educ Sports Sci. 2019;17(4):134-42. doi: <https://doi.org/10.33689/spormetre.562545>.

314. Yildiz M. An acute bout of self-myofascial release increases flexibility without a concomitant deficit in muscle performance in football players. Int J Physiother. 2018;5(3):92-7. doi: <https://doi.org/10.15621/ijphy/2018/v5i3/173932>.

315. Yıldırım Y, Arabacı R, Güngör AK, Görgülü R. The effects of dynamic and static stretching exercises performed to elite wrestlers after high intensity exercise on heart rate variability. Sci Sports. 2022. doi: <https://doi.org/10.1016/j.scispo.2022.03.008>.

316. Young W, Clothier P, Otago L, Bruce L, Liddell D. Acute effects of static stretching on hip flexor and quadriceps flexibility, range of motion and foot speed in kicking a football. J Sci Med Sport. 2004;7(1):23-31. doi: <https://doi.org/10.1016/s1440-2440(04)80040-9>.

317. Zakas A, Galazoulas C, Zakas N, Vamvakoudis E, Vergou A. The Effect of Stretching Duration on Flexibility During Warming Up in Adolescent Soccer Players. Phys Train. 2005:2-. doi: No DOI available.

318. Zakas A. The effect of stretching duration on the lower-extremity flexibility of adolescent soccer players. J Bodyw Mov Ther. 2005;9(3):220-5. doi: <https://doi.org/10.1016/j.jbmt.2004.07.002>.

319. Zakas A, Doganis G, Galazoulas C, Vamvakoudis E. Effect of acute static stretching duration on isokinetic peak torque in pubescent soccer players. Pediatr Exerc Sci. 2006;18(2):252-61. doi: <https://doi.org/10.1123/pes.18.2.252>.

320. Zakas A, Doganis G, Papakonstandinou V, Sentelidis T, Vamvakoudis E. Acute effects of static stretching duration on isokinetic peak torque production of soccer players. J Bodyw Mov Ther. 2006;10(2):89-95. doi: <https://doi.org/10.1016/j.jbmt.2005.04.007>.

321. Zakas A, Galazoulas C, Doganis G, Zakas N. Effect of two acute static stretching durations of the rectus femoris muscle on quadriceps isokinetic peak torque in professional soccer players. Isokinet Exerc Sci. 2006;14(4):357-62. doi: <https://doi.org/10.3233/ies-2006-0249>.

322. Zakas A, Grammatikopoulou MG, Zakas N, Zahariadis P, Vamvakoudis E. The effect of active warm-up and stretching on the flexibility of adolescent soccer players. J Sports Med Phys Fit. 2006;46(1):57-61. doi: No DOI available.

323. Zmijewski P, Lipinska P, Czajkowska A, Mroz A, Kapuscinski P, Mazurek K. Acute Effects of a Static vs. a Dynamic Stretching Warm-up on Repeated-Sprint Performance in Female Handball Players. J Hum Kinet. 2020;72(1):161-72. doi: <https://doi.org/10.2478/hukin-2019-0043>.

324. Zourdos MC, Wilson JM, Sommer BA, Lee SR, Park YM, Henning PC, et al. Effects of dynamic stretching on energy cost and running endurance performance in trained male runners. J Strength Cond Res. 2012;26(2):335-41. doi: <https://doi.org/10.1519/JSC.0b013e318225bbae>.

325. 孙勇. 不同恢复手段对青年男子篮球运动员高强度

间歇训练时运动能力和能量代谢的影响 [Effects of Distinct Recovery Methods on Exercise Performance and Energy Metabolism in High Intensity Interval Training of Young Male Basketball Athletes]. J Shenyang Sport University. 2017;36(4):106-12. doi: No DOI available.

326. Holt BW, Lambourne K. The impact of different warm-up protocols on vertical jump performance in male collegiate athletes [corrected] [published erratum appears in J STRENGTH CONDITION RES 2008 Sep;22(5):1720]. J Strength Cond Res. 2008;22(1):226-9. doi: <https://doi.org/10.1519/JSC.0b013e31815f9d6a>.

1. We understand that any number determined here would be arbitrary. Still, we believe this is a sufficiently large number (certainly, most systematic reviews do not even approach such large number), and it was determined *a priori*, i.e., before the searches were performed. This information is included in the pre-registered protocol (<https://osf.io/6auyj/>). [↑](#footnote-ref-1)
2. Despite “gender” being commonly used in the English language, this is a psychosocial construct of complex evaluation. In the case of our scoping review, what studies reported was sex in the simplest biological sense, and so that was the terminology we adopted in this review. This choice finds support in the literature [2, 3]. [↑](#footnote-ref-2)
3. Although we are aware that neural and psychological outcomes should probably be separated, there are outcomes that reside on the frontier between both (e.g., perception of pain). Therefore, we chose not to separate these two domains. [↑](#footnote-ref-3)
4. EndNote limits group citations to 250. [↑](#footnote-ref-4)
